# Quinolines from the cyclocondensation of isatoic anhydride with ethyl acetoacetate: preparation of ethyl 4-hydroxy-2-methylquinoline-3-carboxylate and derivatives

**DOI:** 10.3762/bjoc.14.229

**Published:** 2018-09-28

**Authors:** Nicholas G Jentsch, Jared D Hume, Emily B Crull, Samer M Beauti, Amy H Pham, Julie A Pigza, Jacques J Kessl, Matthew G Donahue

**Affiliations:** 1 Department of Chemistry and Biochemistry, University of Southern Mississippi, 118 College Drive #5043, Hattiesburg, MS 39406, USA

**Keywords:** cyclodehydration, HIV integrase, isatoic anhydride, masked acyl cyanide, quinoline

## Abstract

A convenient two-step synthesis of ethyl 4-hydroxy-2-methylquinoline-3-carboxylate derivatives has been developed starting from commercially available 2-aminobenzoic acids. In step 1, the anthranilic acids are smoothly converted to isatoic anhydrides using solid triphosgene in THF. In step 2, the anhydride electrophiles are reacted with the sodium enolate of ethyl acetoacetate, generated from sodium hydroxide, in warm *N,N*-dimethylacetamide resulting in the formation of substituted quinolines. A degradation–build-up strategy of the ethyl ester at the 3-position allowed for the construction of the α-hydroxyacetic acid residue required for the synthesis of key arylquinolines involved in an HIV integrase project.

## Introduction

**Figure 1:**
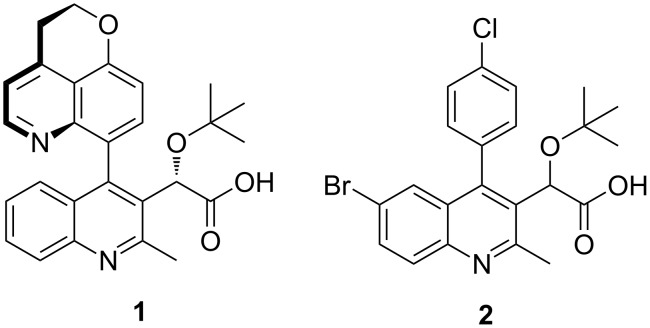
Investigational non-catalytic HIV-1 Integrase inhibitors.

In stark contrast to the prevalence of the quinoline heterocycle in natural products [1], quinolines are only present in approximately 2% of FDA approved prescription pharmaceuticals [2]. Recently, 2,3,4-trisubstituted arylquinolines such as BI 224436 **1** [3–4] and **2** [5–6] have been shown to exhibit inhibitory activity against HIV-1 integrase that is essential for viral replication through integration of viral DNA into host cell chromatin (Figure 1) [7–9]. In contrast to the FDA approved integrase strand transfer inhibitors (INSTIs) dolutegravir, elvitegravir, and raltegravir, arylquinolines **1** and **2** bind to a non-catalytic site of integrase (NCINI) via allosteric binding inhibition.

**Scheme 1:**
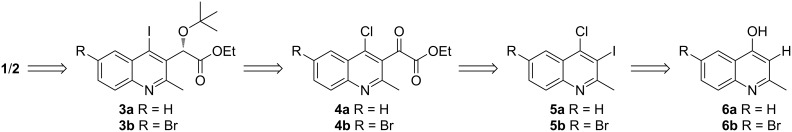
Boehringer Ingelheim retrosynthesis of quinoline **1**.

Structure–activity relationship studies have indicated that the 2-methyl and 3-acetic acid residues are crucial to maintaining the potency of this scaffold [10]. The Boehringer Ingelheim chemical development route toward the synthesis of quinoline **1** is shown in Scheme 1 [11]. The northern tricyclic heterocycle at position 4 is installed by Suzuki coupling with iodide **3a** that is synthesized in three steps from ethyl aryl oxalate **4a**. The α-ketoester side chain at position 3 was installed by selective halogen-metal exchange of iodide **5a** with isopropylmagnesium chloride lithium chloride complex followed by quenching with ethyl chlorooxolate furnishing ethyl oxalate **4a** in 29–79% yield [12]. This sequence works well with the unsubstituted benzene ring of the series **a** compounds where R = H. However, to access quinoline **2** with scaffolds such as **4b** with halogen substitution in the benzene ring at the 5-, 6-, 7- or 8-positions would require a different strategy due to regioselectivity issues encountered in the Grignard acylation step (**5b**→**4b**).

**Scheme 2:**
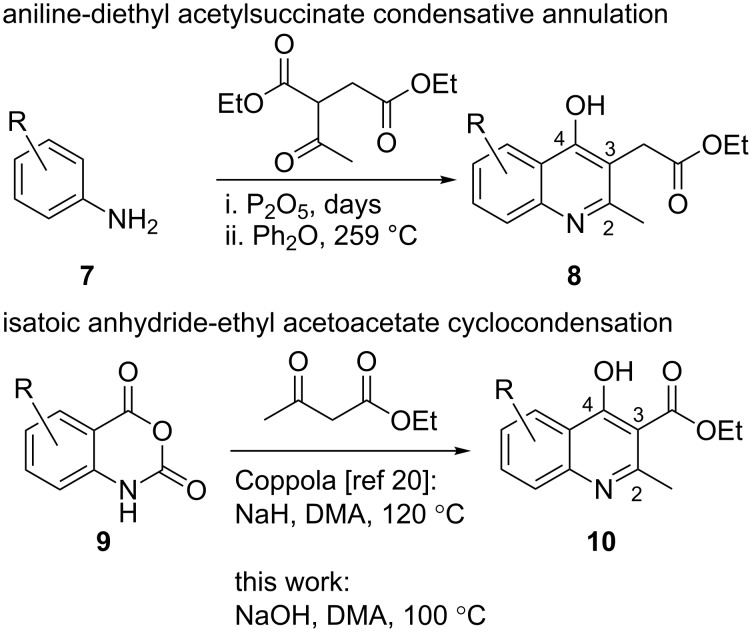
Quinoline ring condensation strategies.

As part of an ongoing research program investigating new assays toward integrase inhibition, we desired to synthesize quinoline derivatives such as **2** with substitution in the benzene ring of the quinolone [13]. The known synthesis of quinoline core **8** has been published in a one-pot, two-step reaction from 4-bromoaniline (**7**) and diethyl acetylsuccinate in 36% yield (Scheme 2) [14–15]. However, our attempts to repeat and scale-up this procedure beyond a few hundred milligrams were met with inconsistent results and variable yields. In our experience, this route is less advantageous due to: (1) the need for a prolonged room temperature condensation reaction time (>5 days in a desiccator with phosphorus pentoxide) to form the initial vinylogous amide; (2) the use of expensive diphenyl ether, which has a nauseating odor, at 0.01 molar dilution at reflux (259 °C) to effect the ring closure via electrophilic aromatic substitution; and (3) the aqueous work-up of this method which does not reliably produce a precipitate that can be filtered easily.

Given the regioselectivity issues and practical challenges associated with the aniline cyclocondensation (**7**→**8**), along with the scarcity of commercially available highly substituted quinolines, we sought to employ an entirely different tactic by utilizing 2*H*-3,1-benzoxazine-2,4(1*H*)-dione (isatoic anhydride) chemistry [16–17]. Isatoic anhydrides **9** are readily prepared from inexpensive, commercially available 2-aminobenzoic acid derivatives (anthranilic acids) with a variety of carbonyl transfer reagents such as phosgene, triphosgene, carbonyldiimidazole, or diethyl carbonate. We therefore employed a modified Coppola quinoline synthesis method through the one-pot acylation of ethyl acetoacetate with isatoic anhydrides followed by dehydrative intramolecular cyclization to access the desired quinoline scaffold **10** [18]. We replaced sodium hydride as the base required to generate the enolate of ethyl acetoacetate with sodium hydroxide [19–20]. The use of sodium hydride is of particular concern upon reaction scale-up due to limited solubility in organic solvents and the production of flammable hydrogen gas [21–22]. Sodium hydroxide avoids the off-gassing of hydrogen and produces water instead, thereby avoiding the use of any special safety precautions.

## Results and Discussion

**Scheme 3:**
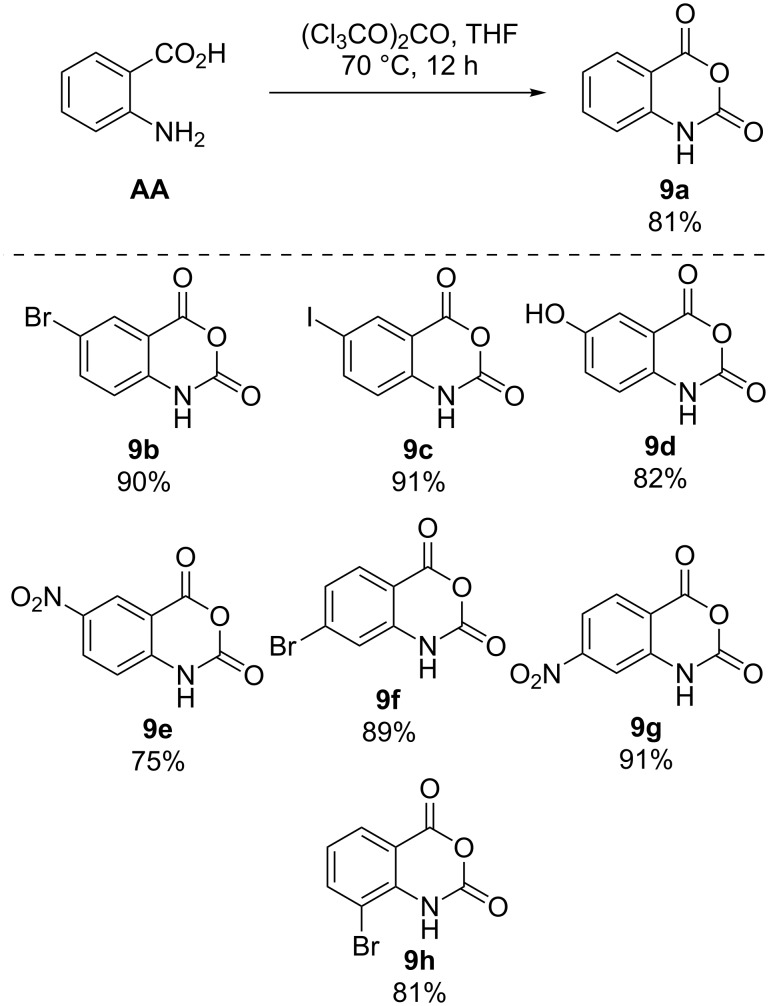
Isatoic anhydrides from anthranilic acids with triphosgene.

The anhydrides **9a**–**h** were readily accessible by treatment of anthranilic acids **AA** with one equivalent of triphosgene in refluxing tetrahydrofuran at 0.36 molar concentration (Scheme 3). This procedure is an alternative to the existing published protocol that relies on the use of toxic phosgene gas [23] as the acylating agent by replacing it with the non-volatile and weighable solid triphosgene [24–25]. After stirring for 12 hours, the reaction mixtures were quenched by pouring into approximately 30 volumes of water (1 volume = 10 mL of water per 1 gram of substrate) with subsequent stirring at room temperature for one hour. The isatoic anhydride products **9a**–**h** typically precipitate out of solution and are readily collected by vacuum filtration. This reverse aqueous quench provides sufficiently pure material without the need for extraction with organic solvent or additional purification. The reactions typically produced high purity products with some colored impurities that were removed by slurring the crude filter cake in methanol followed by vacuum filtration. HPLC analysis of the filtrate showed minuscule loss of product due to this trituration. The reaction is tolerant to halide substitution at the 6-, 7-, and 8-positions (**9b**, **9c**, **9f**, **9h**). The unprotected hydroxy group in **9d** gave reasonably high yield as did electron-withdrawing nitro groups in the 6- and 7-positions (**9e**, **9g**).

**Scheme 4:**
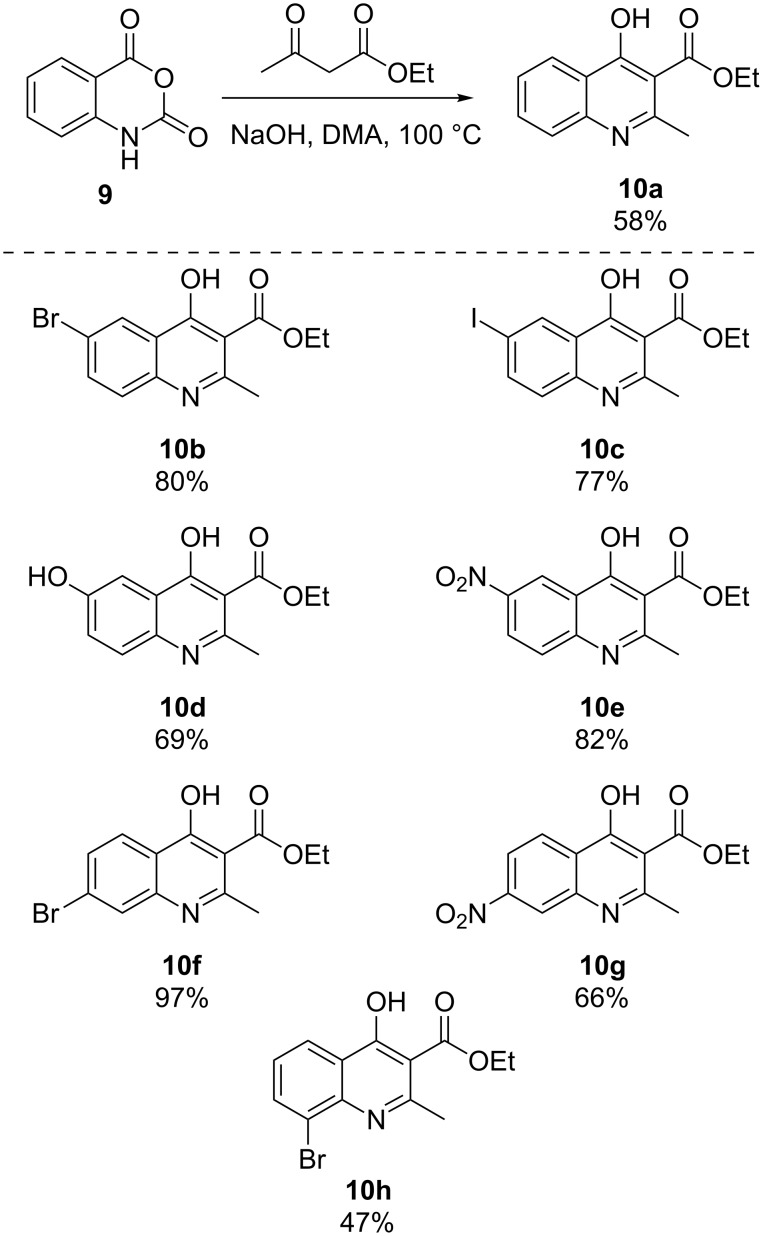
Substituted 2-methyl-4-hydroxyquinolines from isatoic anhydrides and ethyl acetoacetate.

The substituted isatoic anhydrides from Scheme 3 were then subjected to the modified Coppola conditions for the synthesis of 2-methylquinoline derivatives **10a**–**h** (Scheme 4). Most significantly, the investigation found that one equivalent of solid sodium hydroxide in 0.6 molar *N,N*-dimethylacetamide at 100 °C was able to achieve the same result as the Coppola protocol (NaH, DMA, 120 °C). While the use of sodium hydroxide results in the production of water in the reaction medium, we observed no evidence of hydrolysis of the ethyl esters even at elevated temperatures. The reaction is operationally robust and can be carried out without rigorous exclusion of moisture or degassing protocols. As with the isatoic anhydride step, the crude reaction mixtures were poured directly into approximately 30 volumes of water and produced solid material that was readily collected via vacuum filtration. It should be noted that addition of water directly to the reaction vessel did not have the same outcome, typically resulting in oiling out of the product, and requiring organic solvent extraction. The yields ranged from modest (47%) to excellent (97%) on scales from 1 gram up to 25 gram batches with spectral data matching known quinolones [26].

**Scheme 5:**
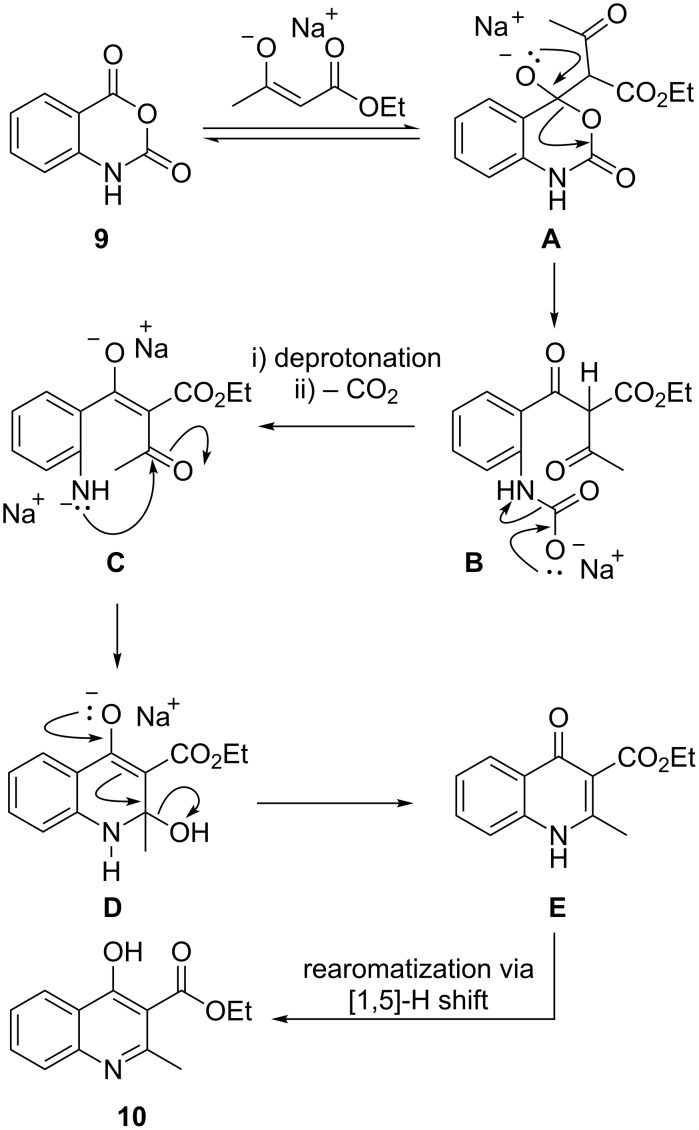
Mechanistic hypothesis for the cyclocondensation reaction.

A plausible mechanism for the formation of the quinoline is shown in Scheme 5 [27]. After initial formation of the enolate of ethyl acetoacetate with sodium hydroxide, water is generated in the reaction mixture, which then serves as a proton transfer agent. The resulting sodium enolate regioselectively attacks the more electrophilic ester carbonyl of the isatoic anhydride forming tetrahedral intermediate **A**. Subsequent collapse of the sp^3^-hybridized carbon to the ketone **B** with concomitant expulsion of carbon dioxide and enolization affords the ketone **C**. The anion of the aniline nitrogen then attacks the ketone carbonyl via intramolecular 6-*exo-trig* cyclization and subsequent proton transfer to the aminal oxygen **D**. Elimination of the 2-hydroxy group from **D** then affords the 4-quinolone **E** that tautomerizes via [1,5]-hydride shift to form quinoline **10**.

**Scheme 6:**
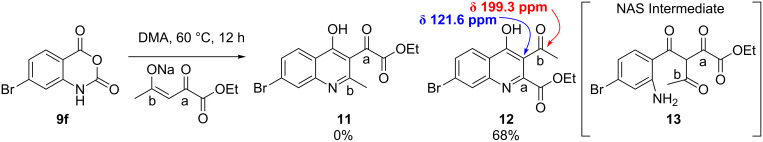
Quinoline synthesis with ethyl acetylpyruvate.

Given the success of employing ethyl acetoacetate in the quinoline cyclocondensation reaction (Scheme 4, **9**→**10**), we hypothesized that ethyl acetopyruvate could install the desired ethyl 2-oxoacetate residue at the 3-position as in **11** that is required for the synthesis of quinolines **1** and **2** (Scheme 6). Whereas with ethyl acetoacetate as shown in Scheme 5 can only condense to form quinolines of type **10** via **C**→**D**, the ethyl acetopyruvate has two carbonyls a and b as depicted in **13** that result in regioisomeric products upon ring closure. The desired quinoline **11** requires ring closure onto carbonyl b.

To that end, ethyl sodioacetopyruvate was prepared via Claisen condensation of acetone with diethyl oxalate and sodium ethoxide in ethanol [28]. Isatoic anhydride **9f** was then added to the solid enolate and both were dissolved in DMA and warmed to 60 °C for 12 hours. Following the general work-up protocol as described for Scheme 4, a light tan solid was isolated in modest yield after trituration with methanol that was determined to be exclusively compound **12** by NMR. The formation of either regioisomer **11** (via b) or **12** (via a) results from 6-*exo-trig* cyclization of the common nucleophilic acyl substitution intermediate **13**. The ^13^C NMR data of the product has a singlet at δ 199 ppm that shows a correlation by HMBC to the singlet integrating to three protons in the ^1^H NMR at δ 2.52 ppm. That same proton singlet in the HMBC only shows one other correlation to a carbon singlet at δ 121.6 ppm. These methyl group correlations can only be observed in compound **12** as the HMBC experiment detects two and three-bond correlations. In **11**, correlation of the methyl singlet to the ketone is ^4^*J*_CH_ and would not be detected. The α-keto carbonyl group a in compound **11** would be expected to have a chemical shift more upfield than δ 199.3 ppm. Therefore, the product was determined to unequivocally be the undesired quinoline **12**.

**Scheme 7:**
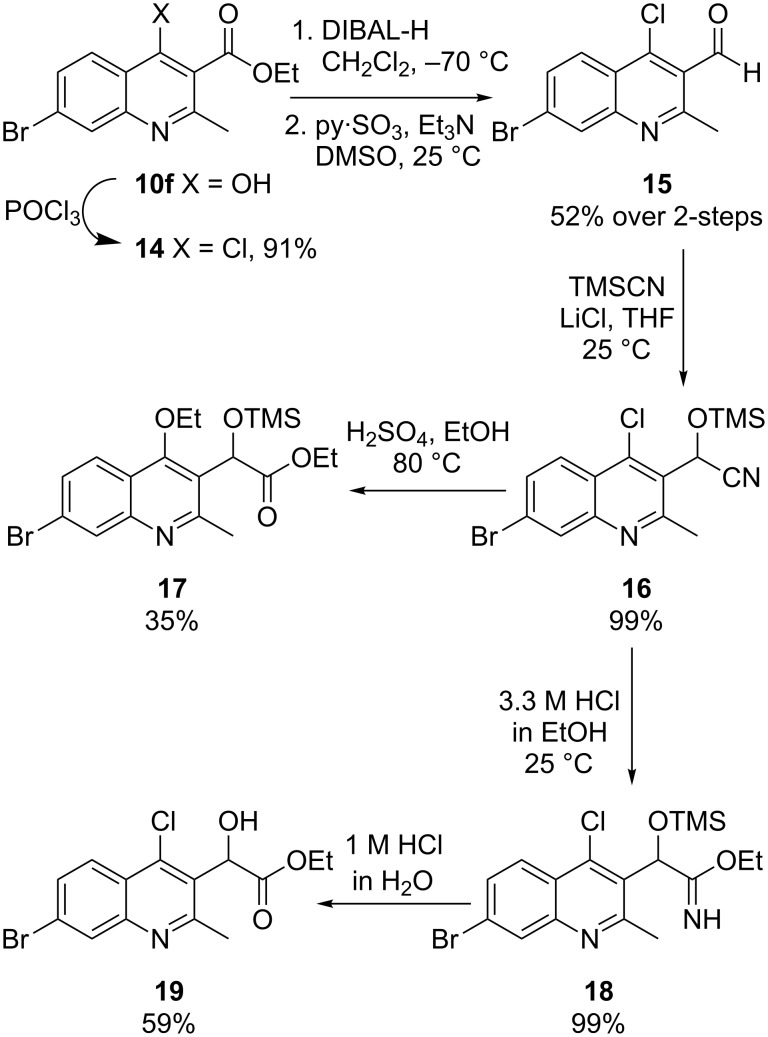
Elaboration of the benzoic acid ethyl ester to the acetic acid residue.

We then turned to a functional group transformation of the ethyl ester at the 3-position to address the acetic acid sidechain problem (Scheme 7). First, the 4-hydroxy group in quinoline **10f** was substituted for chlorine with neat phosphorus oxychloride to afford chloride **14** in 91% yield. The ethyl ester was then smoothly reduced with DIBAL to the benzyl alcohol. While the in-process analysis (TLC and HPLC) indicated quantitative reduction, the isolated yields from the reductions were only poor to modest despite utilizing standard workup conditions with sodium potassium tartrate. Subsequent oxidation of the primary alcohol to the aldehyde **15** was accomplished with the pyridine sulfur trioxide complex in 52% yield over two-steps [29].

The carbon atom at the acid oxidation state was installed by addition of trimethylsilyl cyanide to the aldehyde **15** in the presence of lithium chloride in THF [30]. Initially, the trimethylsilyl cyanohydrin **16** was subjected to solvolysis in ethanol with aqueous sulfuric acid. Unfortunately, those conditions resulted in displacement of the 4-chloro substituent with ethanol giving the 4-ethyl ether **17** in 35% yield. To circumvent this undesired substitution at the 4-position, the cyanohydrin **16** was hydrolyzed by a two-step process. First, hydrogen chloride in ethanol (3.3 molar) was used to produce the imidate **18** in quantitative yield. The structure of **18** was verified by NMR to prove that the 4-position had not suffered displacement by ethanol. Subsequent hydrolysis of **18** with dilute aqueous hydrochloric acid afforded the desired ethyl ester **19** in 59% yield. Given the three steps required to convert the aldehyde **15** into the desired α-hydroxy acetic acid ethyl ester **19**, we decided to pursue a more direct one-carbon homologation procedure.

**Scheme 8:**
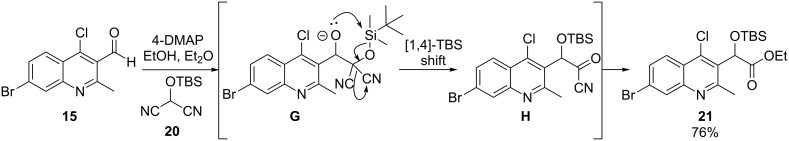
Umpolung addition of ethoxycarbonyl via a MAC strategy.

We envisioned a milder alternative to the acidic conditions required for cyanide hydrolysis that would provide the side chain at the correct oxidation in one pot. To that end, we turned to masked acyl cyanide (MAC) chemistry [31] in which the reagent **20** acts as an acyl anion via umpolung reactivity [32]. With the aldehyde **15** in hand, homologation involving the addition of the MAC reagent **20** afforded the TBS protected α-hydroxyacetic acid ethyl ester **21** in in 76% yield (Scheme 8).

Based on the purported mechanistic reasoning of Nemoto, addition of the methine anion of **20** to **15** proceeds to the intermediate alkoxide **G** that undergoes a [1,4]-shift of the silicon group with concomitant ejection of cyanide anion to form acyl cyanide **H** [33]. As the reaction is run in the presence of ethanol, nucleophilic acyl substitution of cyanide H for the ethoxy group furnishes the ethyl ester **21** in one pot. We were therefore able to construct the desired benzene substituted quinoline **21** in six steps from 2-amino-4-bromobenzoic acid in 31% overall yield.

## Conclusion

In conclusion, an efficient route for the synthesis of substituted quinolines **10a**–**h** has been demonstrated from commercially available anthranilic acids **AA** (Scheme 4). This strategy achieved the replacement of sodium hydride with sodium hydroxide thereby obviating any special equipment requiring the capture or scrubbing of hydrogen gas evolved. The extension of the 3-position ethyl ester into the α-*tert*-butoxy acetic acid residue was also demonstrated via a cyanohydrin-hydrolysis route (**10f**→**19**) and an umpolung acyl addition strategy (**10f**→**21**). The development of a library of quinoline scaffolds is currently underway within our lab utilizing this synthetic process [34–35].

## Experimental

### General procedure for isatoic anhydride synthesis


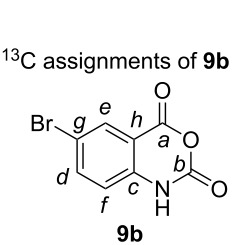


**6-Bromo-2*H*-benzo[*d*][1,3]oxazine-2,4(1*H*)-dione (9b).** A 500 mL single neck round-bottomed flask equipped with a football-shaped PTFE stirring bar (16 mm × 37 mm) was charged with 2-amino-5-bromobenzoic acid (10.0 g, 46.3 mmol, 1.0 equiv) followed by the addition of tetrahydrofuran (230 mL, 0.2 molar) and solid triphosgene (13.7 g, 46.3 mmol, 1.0 equiv) resulting in a suspension. The reaction vessel was placed into a fitted metal heating mantle and the neck was equipped with a 24/40 Liebig condenser. The suspension was stirred (500 rpm) and the heating mantle set to 70 °C. The suspension became homogenous before a white solid precipitated out after about 30 minutes at 70 °C. The heterogeneous reaction mixture was aged for 12 hours then cooled to room temperature (25 °C). The slurry was poured into a 600 mL beaker equipped with overhead mechanical stirrer (PTFE 75 mm paddle) containing 250 mL of deionized water. With vigorous stirring, the mixture became homogenous followed by precipitation of a pale white solid. The solid was collected by vacuum filtration on a Büchner funnel (7.6 cm diameter) with Whatman #1 filter paper (70 mm) and air pulled through for 5 minutes. The material was transferred to a 250 mL Erlenmeyer flask equipped with cylindrical stir bar and 50 mL of methanol was added. The slurry was stirred for 10 minutes and then collected by vacuum filtration. The filter cake was dried under vacuum (0.1 mmHg at 25 °C) for 12 hours to afford **9b** as a white powder (90% yield). Physical characteristics of **9b**: white powder with 95.4% purity as determined by quantitative ^1^H NMR using maleic acid as the internal standard; mp > 270 °C; IR (solid) cm^−1^: 3170, 1751; ^1^H NMR (DMSO-*d*_6_, 400 MHz) δ 11.86 (s, 1H, N-H), 7.98 (d, *J* = 2.3 Hz, 1H, C*e*-H), 7.88 (dd, *J* = 8.7, 2.3 Hz, 1H, C*d*-H), 7.19 (d, *J* = 8.7 Hz, 1H, C*f*-H); ^13^C NMR (DMSO-*d*_6_, 100 MHz) δ 158.8 (s, C*a*), 146.7 (s, C*b*), 140.6 (s, C*c*), 139.3 (d, C*d*), 130.5 (d, C*e*), 117.6 (d, C*f*), 114.5 (s, C*g*), 112.4 (s, C*h*); HRMS (ESI): calcd for [C_8_H_4_BrNO_3_ + Na]^+^ 263.926677; found: 263.926475; anal. calcd for C_8_H_4_BrNO_3_: C, 50.34; H, 3.90; found: C, 49.98; H, 3.80.

### General procedure for quinoline synthesis


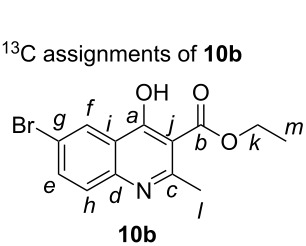


**Ethyl 6-bromo-4-hydroxy-2-methyl-3-carboxylate (10b).** To a 250 mL single-necked round bottom flask equipped with football-shaped PTFE stir bar (16 mm × 37 mm) was added isatoic anhydride **9b** (10.85 g, 44.8 mmol, 1.0 equiv), ethyl acetoacetate (11.3 mL, 89.7 mmol, 2.0 equiv), and *N,N*-dimethylacetamide (75 mL, 0.6 molar) resulting in a clear yellow solution. To the reaction solution was then added solid sodium hydroxide (1.79 g, 44.8 mmol, 1.0 equiv) that dissolved over time. The reaction vessel was placed in a fitted metal heating mantle and heated at 100 °C for 12 hours. The reaction solution was cooled to room temperature (25 °C) and poured into a 500 mL beaker equipped with cylindrical PTFE stir bar (16 mm × 37 mm) containing 250 mL of deionized water causing a beige solid to precipitate out of solution. The solid was collected on a Büchner funnel (7.6 cm diameter) with Whatman #1 filter paper (70 mm) and air dried by pulling a vacuum through for 10 minutes. The material was further dried in a vacuum oven (0.1 mmHg at 25 °C) for 24 hours to afford 11.1g of **10b** as a white powder (80% yield). Physical and spectroscopic characteristics of **10b**: white powder with 97.9% purity as determined by quantitative ^1^H NMR using maleic acid as the internal standard; mp 270–272 °C; IR (solid) cm^−1^ 1705; ^1^H NMR (DMSO-*d*_6_, 400 MHz) δ 12.03 (s, 1H, O-H), 8.13 (d, *J* = 2.3 Hz, 1H, C*f*-H), 7.82 (dd, *J* = 8.8, 2.4 Hz, 1H, C*e*-H), 7.51 (d, *J* = 8.8 Hz, 1H, C*h*-H), 4.24 (q, *J* = 7.1 Hz, 2H, C*k*-H), 2.39 (s, 3H, C*l*-H), 1.27 (t, *J* = 7.1 Hz, 1H, C*m*-H); ^13^C NMR (DMSO-*d*_6_, 100 MHz) δ 172.0 (s, C*a*), 166.4 (s, C*b*), 149.4 (s, C*c*), 138.0 (s, C*d*), 134.9 (d, C*e*), 127.1 (d, C*f*), 126.0 (s, C*g*), 120.6 (d, C*h*), 116.3 (s, C*i*), 115.0 (s, C*j*), 60.4 (t, C*k*), 18.2 (q, C*l*), 14.1 (q, C*m*); HRMS (ESI): calcd for [C_13_H_12_BrNO_3_ + Na]^+^ 331.989277; found: 331.988788; anal. calcd for C_13_H_12_BrNO_3_: C, 39.70; H, 1.67; found C, 39.51; H, 1.74.

## Supporting Information

File 1 Experimental procedures and analytical data.

File 2 IR, NMR and mass spectra, as well as elemental analyses.
