# Supplementary material for: Quinolines from the cyclocondensation of isatoic anhydride with ethyl acetoacetate: preparation of ethyl 4-hydroxy-2-methylquinoline-3-carboxylate and derivatives
Source: Beilstein J Org Chem. 2018 Sep 28;14:2529–36. doi: 10.3762/bjoc.14.229 (PMC6178280; doi:10.3762/bjoc.14.229)
Supplement: File 1 — Experimental procedures and analytical data. [file Beilstein_J_Org_Chem-14-2529-s001.pdf]

Supporting Information

for

Quinolines from the cyclocondensation of isatoic anhydride with ethyl acetoacetate: preparation of ethyl 4-hydroxy-2-methylquinoline-3-carboxylate and derivatives

Nicholas G. Jentsch, Jared D. Hume, Emily B. Crull, Samer M. Beauti, Amy H. Pham, Julie A. Pigza, Jacques J. Kessl and Matthew G. Donahue*

Address: [1]Department of Chemistry and Biochemistry, University of Southern Mississippi, 118 College Drive #5043, Hattiesburg, MS 39406

Email: Matthew G. Donahue - matthew.donahue@usm.edu
*Corresponding author

Experimental procedures and analytical data

Table of contents

General Procedures S3

1*H*-Benzo[*d*][1,3]oxazine-2,4-dione (**9a**): S7

6-Bromo-1*H*-benzo[*d*][1,3]oxazine-2,4-dione (**9b**): S8

6-Iodo-1*H*-benzo[*d*][1,3]oxazine-2,4-dione (**9c**): S8

6-Hydroxy-1*H*-benzo[*d*][1,3]oxazine-2,4-dione (**9d**): S9

6-Nitro-1*H*-benzo[*d*][1,3]oxazine-2,4-dione(**9e**): S9

7-Bromo-1*H*-benzo[*d*][1,3]oxazine-2,4-dione (**9f**): S9

7-Nitro-1*H*-benzo[*d*][1,3]oxazine-2,4-dione (**9g**): S10

8-Bromo-1*H*-benzo[*d*][1,3]oxazine-2,4-dione (**9h**): S10

Ethyl 4-hydroxy-2-methylquinoline-3-carboxylate (**10a**): S12

Ethyl 6-bromo-4-hydroxy-2-methylquinoline-3-carboxylate (**10b**): S13

Ethyl 4-hydroxy-6-iodo-2-methylquinoline-3-carboxylate (**10c**): S14

Ethyl 4,6-dihydroxy-2-methylquinoline-3-carboxylate (**10d**): S14

Ethyl 4-hydroxy-2-methyl-6-nitroquinoline-3-carboxylate (**10e**): S15

Ethyl 7-bromo-4-hydroxy-2-methylquinoline-3-carboxylate (**10f**): S15

Ethyl 4-hydroxy-2-methyl-7-nitroquinoline-3-carboxylate (**10g**): S15

Ethyl 8-bromo-4-hydroxy-2-methylquinoline-3-carboxylate (**10h**): S16

Ethyl 3-acetyl-7-bromo-4-hydroxyquinoline-2-carboxylate (**12**): S16

Ethyl 7-bromo-4-chloro-2-methylquinoline-3-carboxylate (**14**): S17

(7-Bromo-4-chloro-2-methylquinolin-3-yl)methanol (**SI-1**): S18

7-Bromo-4-chloro-2-methylquinoline-3-carbaldehyde (**15**): S19

2-(7-Bromo-4-chloro-2-methylquinolin-3-yl)-2-((trimethylsilyl)oxy)acetonitrile (**16**): S20

Ethyl 2-(7-bromo-4-chloro-2-methylquinolin-3-yl)-2-hydroxyacetate (**19**): S21

2-(7-Bromo-4-chloro-2-methylquinolin-3-yl)-2-((*tert*-butyldimethylsilyl)oxy)acetonitrile (**21**): S22

General procedures

Materials: Common abbreviations [1]. Starting materials were purchased from commercial vendors and used without purification unless noted [2].

Chemical Abstracts Service Registry Numbers:

Compound Number	CAS #
**9a**	118-48-9
**9b**	4692-98-2
**9c**	116027-10-2
**9d**	195986-91-5
**9e**	4693-02-1
**9f**	76561-16-5
**9g**	63480-10-4
**9h**	331646-98-1
**10a**	73987-39-0
**10b**	88960-40-1
**10c**	400825-48-1
**10d**	No CAS# as if 25-July-2018
**10e**	1397218-36-8 [3]
**10f**	1374869-12-1
**10g**	No CAS# as if 25-July-2018
**10h**	No CAS# as if 25-July-2018
**12**	No CAS# as if 25-July-2018
**14**	No CAS# as if 25-July-2018
**SI-1**	No CAS# as if 25-July-2018
**15**	No CAS# as if 25-July-2018
**16**	No CAS# as if 25-July-2018
**17**	No CAS# as if 25-July-2018
**18**	No CAS# as if 25-July-2018
**19**	No CAS# as if 25-July-2018
**21**	No CAS# as if 25-July-2018

[1] For a general overview of organic chemistry acronyms see: Daub, G. H.; Leon, A. A.; Silverman, I. R.; Daub, G. W.; Walker, S. B. "The Use of Acronyms in Organic Chemistry." *Aldrichimica Acta* **1984**, 17(*1*), 13-23.
[2] For general purification procedures see: Armarego, W. L. F.; Perrin, D. D. "Purification of Laboratory Chemicals." 4th Ed. 1996, Butterworth-Heinemann.
[3] CAS# registered but no journals or patents

General experimental techniques: Unless otherwise noted, all reactions were carried out using flame-dried glassware and standard syringe, cannula, and septa techniques, when necessary [4]. Tetrahydrofuran (THF), diethyl ether (Et_2O), hexanes, dichloromethane (CH_2Cl_2), and toluene ($PhCH_3$) were dried by passage through a column of activated alumina on an mBraun SPS [5]. Triethylamine (Et_3N), *N,N*-dimethylformamide (DMF), and acetonitrile (ACN) were dried by passage through a column of activated alumina on an Innovative Technologies system. Trimethylsilyl chloride and Hünig's base were distilled from calcium hydride under argon. Pyridine was distilled from potassium hydroxide under nitrogen. Analytical thin layer chromatography was performed using Sorbent Technologies 250 μm glass-backed UV254 silica gel plates. The plates were first visualized by fluorescence upon 254 nm irradiation then by iodine chamber. The plates were then dipped in one of the following stains followed by heating: *p*-anisaldehyde, phosphomolybdic acid, vanillin, ceric ammonium molybdate, potassium iodoplatinate, ninhydrin or bromocresol green. Flash column chromatography was performed using Sorbent Technologies 40–63 μm, pore size 60 Å silica gel with solvent systems indicated. Solvent removal was effected using a Buchi R3 rotary evaporator with a V900 diaphragm pump (≈10 mmHg). All yields refer to isolated material that is chromatographically (TLC or HPLC) and spectroscopically (^{1}H NMR) homogenous.

---

[4] For general laboratory techniques see: (a) Furniss, B. S.; Hannaford, A. J.; Smith, P. W. G.; Tatchell, A. R. "Vogel's, Textbook of Practical Organic Chemistry." 5th Ed. **1989**, Longman. (b) "Handling Air-Sensitive Reagents" Aldrich Technical Bulletin AL-134, revised 12/94; (c) "Handling Pyrophoric Reagents" Aldrich Technical Bulletin, revised 06/95.

[5] Pangborn, A. B.; Giardello, M. A.; Grubbs, R. H.; Rosen, R. K.; Timmers, F. J. *Organometallics* **1996**, *15*, 1518.

Characterization: All melting points were taken with a Thomas Hoover melting point apparatus and are uncorrected. Infrared spectra were recorded on a Nicolet Nexus 470 FTIR spectrometer as neat liquids, oils, solids or as thin films formed from evaporation of NMR solvent over the ATR plate. HPLC were recorded on an Agilent 1260 Infinity using a Poroshell 120 EC-C18 (3.0 × 50 mm, 2.7 micron) column with a binary gradient of 0.1% TFA in H_2O (**A**) and 0.1% TFA in CH_3CN (**B**): [0 min: **A** (95%), **B** (5%); 7 min: **A** (5%), **B** (95%); 8 min: **A** (5%), **B** (95%)] monitoring 214, 254, and 280 nm. Low-resolution mass spectra were recorded on a ThermoFinnigan LXQ ESI-LCMS by use of chemical ionization (CI). High-resolution mass spectra were recorded either at the Old Dominion University College of Science Major Instrumentation Center (COSMIC) on a Bruker 12 Tesla APEX-Qe FTICR-MS with an Apollo II ion source or The Ohio State University Campus Chemical Instrumentation Center (CCIC). Combustion analysis was performed at Atlanitic Microlabs on samples taken from the bulk of the material.

NMR Parameters: Proton nuclear magnetic resonance spectra were recorded on a Bruker UltraShield Plus 400 MHz spectrometer and are recorded in parts per million from internal chloroform (7.26 ppm), benzene, or dimethylsulfoxide on the δ scale and are reported as follows: chemical shift [multiplicity (s = singlet, d = doublet, t = triplet, q = quartet, qu = quintet, m = multiplet), coupling constant(s) in hertz, integration, interpretation] [6]. ^{13}C NMR data were recorded on a Bruker UltraShield Plus 400 MHz spectrometer and are reported as follows: chemical shift (multiplicity as determined from DEPT (CH, CH_3 up and CH_2 down) and/or HSQC experiments.

[6] For an overview of NMR analysis see : (a) Hoye, T. R.; Hanson, P. R. ; Vyvyan, J. R. « A Practical Guide to First-Order Multiplet Analysis in ^1H NMR Spectroscop. « *J. Org. Chem.* **1994**, *59*, 4096-4103.

Table 1: ^{1}H NMR data (DMSO-d_6) for isatoic anhydrides x

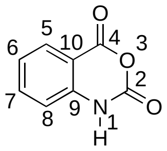

Entry	Isatoic Anhydride	^{1}H Position δ ppm (J Hz)				
		1	5	6	7	8
1	**9a**	11.75	7.92 (7.9)	7.26 (8.1, 0.9)	7.75 (8.0, 7.2, 1.5)	7.17 (8.0)
2	**9b**	11.86	7.10 (8.7)	–	7.89 (8.7, 2.3)	7.99 (2.3)
3	**9c**	–	8.13 (1.6)	–	8.01 (8.5, 1.8)	6.96 (8.5)
4	**9d**	–	8.13 (1.6)	–	8.01 (8.5, 1.8)	6.96 (8.5)
5	**9e**	12.35	8.56 (2.6)	–	8.52 (9.0, 2.6)	7.30 (9.1)
6	**9f**	11.83	7.83 (8.4)	7.42 (8.4, 1.6)	–	7.30 (1.6)
7	**9g**	12.09	8.16 (8.6)	7.96 (8.6, 2.2)	–	7.86 (9.1)
8	**9h**	11.08	7.95 (7.8, 1.4)	7.19 (7.8, 7.8)	8.02 (7.9, 1.4)	–

Table 2: ^{13}C NMR data (DMSO- d_6) for isatoic anhydrides x

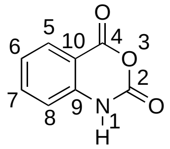

Entry	Isatoic Anhydride	^{13}C Position δ ppm							
		2	4	5	6	7	8	9	10
1	**9a**	147.5	160.3	129.4	124.0	137.4	115.8	141.9	110.7
2	**9b**	146.7	158.8	117.6	114.5	139.3	130.5	140.6	112.4
3	**9c**	147.2	159.1	136.9	86.5	145.3	118.1	141.4	113.1
4	**9d**	147.4	160.4	112.8	153.8	126.1	117.2	134.4	111.2
5	**9e**	147.0	159.1	125.0	142.9	131.7	117.1	146.5	111.6
6	**9f**	147.3	159.8	131.2	126.9	130.7	118.2	142.9	110.2
7	**9g**	147.1	159.2	131.4	117.8	152.4	110.7	142.5	115.9
8	**9h**	147.0	159.6	129.1	125.0	140.6	113.4	140.0	108.4

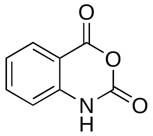

9a
2*H*-benzo[*d*][1,3]oxazine-2,4(1*H*)-dione

1*H*-Benzo[*d*][1,3]oxazine-2,4-dione (9a): To a single neck 500 mL round bottom flask was added anthranilic acid (10.0 g, 72.9 mmol) and tetrahydrofuran (200 mL, 2.4 mol) giving a dark brown solution. Triphosgene (21.64 g, 72.9 mmol) was added to the mixture that resulted in a tan color suspension. The flask was equipped with a reflux condenser and the reaction was heated to 70 °C and stirred at 800 rpm for 12 hours. The reaction was cooled to room temperature and was poured into a 600 mL beaker containing 300 mL of distilled water and was stirred vigorously for one hour using an overhead mechanical stirrer. A white precipitate formed and was vacuum filtered through a Büchner funnel. The solid was collected and stirred in 100 mL of methanol for

30 minutes. The slurry was then collected via vacuum filtration through a Büchner funnel to afford 2*H*-benzo[*d*][1,3]oxazine-2,4(1*H*)-dione (9.61 g, 81%) as a white solid. mp 249-250ºC; IR (solid) cm^{-1} 1724; ^{1}H NMR (DMSO- d_6, 400 MHz) δ: 11.75 (s, 1H, N-H), 7.92 (d, J = 7.9 Hz, 1H), 7.75 (dd, J = 8.0, 7.2, 1.5 Hz, 1H), 7.26 (d, J = 7.9 Hz, 1H), 7.17 (dd, J = 8.1, 0.9 Hz, 1H); ^{13}C NMR (DMSO- d_6, 100 MHz) δ: 160.3 (s), 147.5 (s), 141.9 (s), 137.4 (d), 129.4 (d), 124.0 (d), 115.8 (d), 110.7 (s).

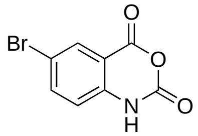

9b
6-bromo-2*H*-benzo[*d*][1,3]oxazine-2,4(1*H*)-dione

6-Bromo-1*H*-benzo[*d*][1,3]oxazine-2,4-dione [7] **(9b):** mp >260 ºC; IR (solid) cm^{-1} 3169, 1754, 1691; ^{1}H NMR (DMSO- d_6, 400 MHz) δ: 11.86 (s, 1H, N-H), 7.99 (d, J = 2.3 Hz, 1H), 7.89 (dd, J = 8.7, 2.3 Hz, 1H), 7.10 (d, J = 8.7 Hz, 1H); ^{13}C NMR (DMSO- d_6, 100 MHz) δ: 158.8 (s), 146.7 (s), 140.6 (s), 139.3 (d), 130.5 (d), 117.6 (d), 114.5 (s), 112.4 (s).

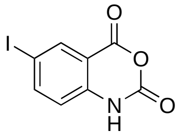

9c
6-iodo-2*H*-benzo[*d*][1,3]oxazine-2,4(1*H*)-dione

6-Iodo-1*H*-benzo[*d*][1,3]oxazine-2,4-dione (9c): mp >260 ºC; IR (solid) cm^{-1} 3169, 1751, 1695; ^{1}H NMR (DMSO- d_6, 400 MHz) δ: 11.82 (s, 1H, N-H), 8.13 (d, J = 1.6 Hz, 1H), 8.01 (dd, J = 8.5, 1.8 Hz, 1H), 6.96 (d, J = 8.5 Hz, 1H); ^{13}C NMR (DMSO- d_6, 100 MHz) δ: 159.1 (s), 147.2 (s), 145.3 (s), 141.4 (d), 136.9 (d), 118.1 (d), 113.1 (s), 86.5 (s).

---

[7] Zhang, W.-Z.; Zhang, N.; Sun, Y.-Q.; Ding, Y.-W.; Lu, X.-B. "Palladium-Catalyzed Cyclization Reaction of *o*-Iodoanilines, CO_2 and CO: Access to Isatoic Anhydrides." *ACS Catal.* **2017**, *7*, 8072-8076.

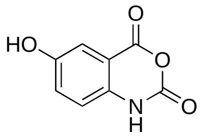

9d
6-hydroxy-2*H*-benzo[*d*][1,3]oxazine-2,4(1*H*)-dione

6-Hydroxy-1*H*-benzo[*d*][1,3]oxazine-2,4-dione [8] (9d): mp >260 ºC; IR (solid) cm^{-1} 3269, 1771, 1695; ^{1}H NMR (DMSO-d_6, 400 MHz) δ: 11.51 (s, 1H, N-H), 9.87(s, 1H, O-H), 7.22 (s, 1H), 7.20 (m, 1H) 7.03 (d, J = 8.4 Hz, 1H); ^{13}C NMR (DMSO- d_6, 100 MHz) δ: 160.4 (s), 146.7 (s), 140.6 (s), 139.3 (d), 130.5 (d), 117.6 (d), 114.5 (s), 112.4 (s) .

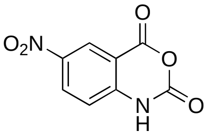

9e
6-nitro-2*H*-benzo[*d*][1,3]oxazine-2,4(1*H*)-dione

6-Nitro-1*H*-benzo[*d*][1,3]oxazine-2,4-dione (9e): mp >260 ºC; IR (solid) cm^{-1} 3169, 1747, 1695; ^{1}H NMR (DMSO-d6, 400 MHz) δ: 12.35 (s, 1H, N-H), 8.56 (d, J = 2.6 Hz, 1H), 8.52 (dd, J = 9.0, 2.6 Hz, 1H), 7.30 (d, J = 9.1 Hz, 1H); ^{13}C NMR (DMSO-d_6, 100 MHz) δ: 159.1 (s), 147.0 (s), 146.5 (s), 142.9 (s), 131.7 (d), 125.0 (d), 117.1 (d), 111.6 (s).

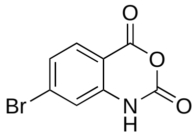

9f
7-bromo-2*H*-benzo[*d*][1,3]oxazine-2,4(1*H*)-dione

7-Bromo-1*H*-benzo[*d*][1,3]oxazine-2,4-dione [9] (9f): mp >260 ºC; IR (solid) cm^{-1} 3169, 1772, 1700; ^{1}H NMR (DMSO- d_6, 400 MHz) δ: 11.83 (s, 1H, N-H), 7.83 (d, J = 8.4 Hz, 1H), 7.42 (dd, J

---

[8] Sawatzky, E.; Wehle, S.; Kling, B.; Wendrich, J.; Bringmann, G.; Sotriffer, C. A.; Heilmann, J.; Decker, M. "Discovery of Highly Selective and Nanomolar Carbamate-Based Butyrylcholinesterase Inhibitors by Rational Investigation into Their Inhibition Mode." *J. Med. Chem.* **2016**, *59*, 2067-2082.

[9] Verma, C.; Sharma, S.; Pathak, A. "A Phosgene and Peroxide-Free One-Pot Tandem Synthesis of Isatoic Anhydrides Involving Anthranilic Acid, Boc Anhydride, and 2-Chloro-*N*-Methyl Pyridinium Iodide." *Tet. Lett.* **2013**, *54*, 6897-6899.

= 8.4, 1.6 Hz, 1H), 7.30 (d, J = 1.6 Hz, 1H); ^{13}C NMR (DMSO- d_6, 100 MHz) δ: 159.8 (s), 147.3 (s), 142.9 (s), 131.2 (d), 130.7 (s), 126.9 (d), 118.2 (d), 110.2 (s).

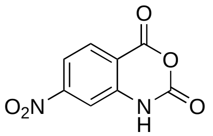

9g
7-nitro-2*H*-benzo[*d*][1,3]oxazine-2,4(1*H*)-dione

7-Nitro-1*H*-benzo[*d*][1,3]oxazine-2,4-dione [10] (9g): mp >260 ºC; IR (solid) cm^{-1} 3173, 1781, 1695; ^{1}H NMR (DMSO- d_6, 400 MHz) δ: 12.09 (s, 1H, N-H), 8.16 (d, J = 8.6 Hz, 1H), 7.96 (dd, J = 8.6, 2.2 Hz, 1H), 7.86 (d, J = 2.2 Hz, 1H); ^{13}C NMR (DMSO- d_6, 100 MHz) δ: 159.2 (s), 152.4 (s), 147.1 (s), 142.5 (s), 131.4 (d), 117.8 (d), 115.9 (s), 110.7 (d).

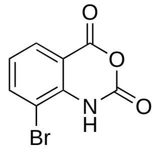

9h
8-bromo-2*H*-benzo[*d*][1,3]oxazine-2,4(1*H*)-dione

8-Bromo-1*H*-benzo[*d*][1,3]oxazine-2,4-dione [10**] (9h):** mp 99-201 ºC; IR (solid) cm^{-1} 1716; ^{1}H NMR (DMSO-d_6, 400 MHz) δ: 11.08 (s, 1H, N-H), 8.02 (dd, J = 7.9, 1.4 Hz, 1H), 7.95 (dd, J = 7.8, 1.4 Hz, 1H), 7.91 (dd, J = 7.8 Hz, 1H); ^{13}C NMR (DMSO-d_6, 100 MHz) δ: 159.6 (s), 147.0 (s), 140.6 (d), 140.0 (s), 129.1 (d), 125.0 (d), 113.4 (s), 108.4 (s).

[10]Wang, G.; Chen, X.; Deng, Y.; Li, Z.; Xu, X. "Synthesis and Nematicidal Activities of 1,2,3-Benzotriazin-4-one Derivatives against *Meloidogyne incognita*." *J. Agric. Food Chem.* **2015**, *63*, 6883-6889.

Table 3: ^{1}H NMR data (DMSO-d_6) for quinolines x

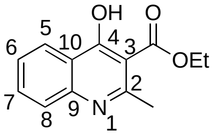

Entry	Quinoline	^{1}H Position δ ppm (*J* Hz)			
		5	6	7	8
1	**10a**	8.06 (8.1, 1.4)	7.35 (8.0, 7.1, 1.0)	7.68 (8.4, 7.0, 1.5)	7.54 (8.0)
2	**10b**	8.13 (2.3)	–	7.82 (8.8, 2.3)	7.51 (8.8, 1.0)
3	**10c**	8.34 (2.0)	–	7.93 (8.7, 2.1)	7.36 (8.7)
4	**10d**	7.38 (2.8)	–	7.16 (8.8, 2.8)	7.42 (8.8)
5	**10e**	8.79 (2.6)	–	8.44 (9.1, 2.7)	7.71 (9.2)
6	**10f**	7.97 (8.6)	7.48 (8.6, 1.8)	–	7.71 (1.8)
7	**10g**	8.28 (8.8)	8.08 (8.9, 2.2)	–	8.39 (2.0)
8	**10h**	8.12 (8.0, 1.4)	7.31 (7.8, 7.8)	8.02(7.8, 1.4)	–

Table 4: ^{13}C NMR data (DMSO-d_6) for quinolines x

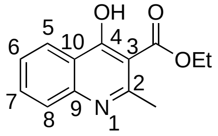

Entry	Quinoline	^{13}C Position δ ppm									
		1	2	3	4	5	6	7	8	9	10
1	**10a**	–	149.3	115.3	173.8	125.5	124.2	132.7	118.4	141.9	110.7
2	**10b**	–	149.9	115.5	172.5	127.6	116.8	135.4	121.1	138.5	126.5
3	**10c**	–	150.1	115.5	172.3	133.9	88.6	140.6	121.3	139.3	126.9
4	**10d**	–	147.9	113.7	173.3	108.2	154.5	122.6	120.0	133.0	126.5
5	**10e**	–	151.3	116.7	172.9	127.9	117.8	139.4	114.7	149.8	128.4
6	**10f**	–	150.2	115.7	173.3	127.8	127.1	124.0	121.0	141.0	125.9
7	**10g**	–	151.0	116.5	173.3	122.0	143.5	126.9	120.4	143.4	124.2
8	**10h**	–	150.4	116.5	173.3	125.5	125.2	136.7	126.6	137.5	111.3

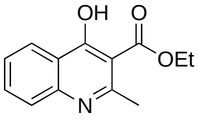

10a
ethyl 4-hydroxy-2-methylquinoline-3-carboxylate

Ethyl 4-hydroxy-2-methylquinoline-3-carboxylate [11] (10a): To a 100 mL single-necked round bottom flask was added 2*H*-benzo[*d*][1,3]oxazine-2,4(1*H*)-dione (5.00 g, 30.6 mmol), ethyl 3-oxobutanoate (7.70 mL, 61.3 mmol), and *N*,*N*-dimethylacetamide (50 mL). Sodium hydroxide (1.20 g, 30.6 mmol) was added to the flask, which resulted to an olive-green color. The reaction was equipped with a reflux condenser and stirred at 100 °C for 12 hours. The reaction was cooled to ambient temperature then was poured into a 600 mL beaker containing 200 mL of distilled water

[11] Staiger, R. P.; Miller, E. B. "Isatoic Anhydride. IV. Reactions with Various Nucleophiles" *J. Org. Chem.* **1959**, *24*, 1214-1219.

and was vigorously stirred for 30 min using an overhead mechanical stirrer. The white precipitate was vacuum filtered through a Büchner funnel and was rinsed with 50 mL of distilled water. The solid was collected and dried overnight under high vacuum to afford ethyl 4-hydroxy-2-methylquinoline-3-carboxylate (4.1 g, 58%) as a white solid. mp 224-228 ºC; IR (solid) cm^{-1} 2873, 1717; ^{1}H NMR (DMSO-d_6, 400 MHz) δ: 8.06 (dd, J = 8.1, 1.4 Hz, 1H), 7.68 (ddd, J = 8.4, 7.0, 1.5 Hz, 1H), 7.54 (d, J = 8.0 Hz, 1H), 7.35 (ddd, J = 8.0, 7.1, 1.0 Hz, 1H), 4.24 (q, J = 7.1, 2H), 2.34 (s, 3H), 1.28 (t, J = 7.1, 3H); ^{13}C NMR (DMSO-d_6, 100 MHz) δ: 173.8 (s), 167.2 (s), 149.3 (s), 139.6 (s), 132.7 (d), 125.5 (d), 125.0 (s), 124.2 (d), 118.4 (d), 115.3 (s), 60.7 (t), 18.6 (q), 14.6 (q); Anal. Calcd. for $C_8H_5NO_3$: C, 67.52; H, 5.67. Found C, 66.68; H, 5.73.

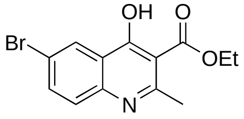

10b
ethyl 6-bromo-4-hydroxy-2-methylquinoline-3-carboxylate

Ethyl 6-bromo-4-hydroxy-2-methylquinoline-3-carboxylate (10b): White Solid; mp >260ºC; IR (solid) cm^{-1} 2904, 1704; ^{1}H NMR (DMSO-d_6, 400 MHz) δ: 12.04 (s, 1H, O-H), 8.13 (d, J = 2.3 Hz, 1H), 7.82 (dd, J = 8.8, 2.3 Hz, 1H), 7.51 (dd, J = 8.0, 1.0 Hz, 1H), 4.25 (q, J = 7.1, 2H), 2.39 (s, 3H), 1.28 (t, J = 7.1, 3H); ^{13}C NMR (DMSO-d_6, 100 MHz) δ: 172.5 (s), 166.9 (s), 149.9 (s), 138.5 (s), 135.4 (d), 127.6 (d), 126.5 (s), 121.1 (d), 116.8 (s), 115.5 (s), 60.9 (t), 18.7 (q), 14.6 (q); HRMS (ESI+): Exact mass calcd for $C_{13}H_{12}BrNO_3$ $[M+Na]^+$, 331.989277. Found 331.988788. Anal. Calcd. for $C_{13}H_{12}BrNO_3$: C, 50.34; H, 3.90. Found C, 50.10; H, 3.88.

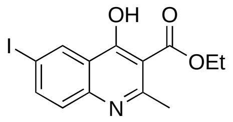

10c
ethyl 4-hydroxy-6-iodo-2-methylquinoline-3-carboxylate

Ethyl 4-hydroxy-6-iodo-2-methylquinoline-3-carboxylate [12] (10c): White Solid; mp >260ºC; IR (solid) cm^{-1} 2904, 1704; ^1H NMR (DMSO- d_6, 400 MHz) δ: 12.04 (s, 1H, O-H), 8.13 (d, J = 2.3 Hz, 1H), 7.82 (dd, J = 8.8, 2.3 Hz, 1H), 7.51 (dd, J = 8.0, 1.0 Hz, 1H), 4.25 (q, J = 7.1, 2H), 2.39 (s, 3H), 1.28 (t, J = 7.1, 3H); ^{13}C NMR (DMSO-d_6, 100 MHz) δ: 172.5 (s), 166.9 (s), 149.9 (s), 138.5 (s), 135.4 (d), 127.6 (d), 126.5 (s), 121.1 (d), 116.8 (s), 115.5 (s), 60.9 (t), 18.7 (q), 14.6 (q); Anal. Calcd. for $C_{13}H_{12}INO_3$: C, 43.72; H, 3.39. Found C, 43.43; H, 3.29.

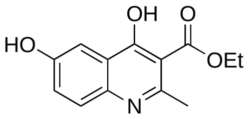

10d
ethyl 4,6-dihydroxy-2-methylquinoline-3-carboxylate

Ethyl 4,6-dihydroxy-2-methylquinoline-3-carboxylate (10d): White Solid; mp >260ºC; IR (solid) cm^{-1} 3365, 1705; ^1H NMR (DMSO-d_6, 400 MHz) δ: 11.74 (s, 1H, O-H), 9.74 (s, 1H, O-H) 7.42 (d, J = 8.8 Hz, 1H), 7.38 (d, J = 2.8 Hz, 1H), 7.16 (dd, J = 8.8, 2.8 Hz, 1H), 4.22 (q, J = 7.1, 2H), 2.36 (s, 3H), 1.27 (t, J = 7.1, 3H); ^{13}C NMR (DMSO-d_6, 100 MHz) δ: 173.3 (s), 167.6 (s), 154.5 (s), 147.9 (s), 133.0 (s), 126.5 (s), 122.6 (d), 120.0 (d), 113.7 (s), 108.2 (d), 60.6 (t), 18.5 (q), 14.6 (q); Anal. Calcd. for $C_{13}H_{13}NO_4$: C, 63.15; H, 5.30. Found C, 62.89; H, 5.44.

[12] Ghorab, M. M.; Abdel-Hamide, S. G.; Farrag, H. A. "Synthesis of Novel Quinolines, Pyranoquinolines, Furanoquinolines, Thienoquinolines and Their Effect on the Ultrastructure of Some Pathogenic Microorganisms." *Acta. Poloniae Pharmaceutica* **2001**, *58*, 175-184

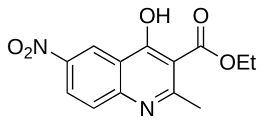

10e
ethyl 4-hydroxy-2-methyl-6-nitroquinoline-3-carboxylate

Ethyl 4-hydroxy-2-methyl-6-nitroquinoline-3-carboxylate (10e): Yellow Solid; mp >260ºC; IR (solid) cm^{-1} 1708; ^{1}H NMR (DMSO-d_6, 400 MHz) δ: 8.79 (d, J = 2.6 Hz, 1H), 8.44 (d, J = 9.1, 2.7 Hz, 1H), 7.71 (d, J = 9.2 Hz, 1H), 4.28 (q, J = 7.1, 2H), 2.43 (s, 3H), 1.29 (t, J = 7.1, 3H); ^{13}C NMR (DMSO-d_6, 100 MHz) δ: 173.3 (s), 166.4 (s), 151.0 (s), 143.5 (s), 143.4 (s), 126.9 (d), 124.2 (s), 122.0 (d), 120.4 (d), 116.5 (s), 61.1 (t), 18.7 (q), 14.6 (q); Anal. Calcd. for $C_{13}H_{12}N_2O_5$: C, 56.52; H, 4.38. Found C, 56.26; H, 4.43.

.

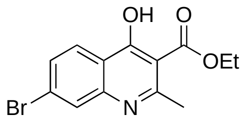

10f
ethyl 7-bromo-4-hydroxy-2-methylquinoline-3-carboxylate

Ethyl 7-bromo-4-hydroxy-2-methylquinoline-3-carboxylate (10f): White Solid; mp >260ºC; IR (solid) cm^{-1} 3073, 1711; ^{1}H NMR (DMSO-d_6, 400 MHz) δ: 7.97 (d, J = 8.6 Hz, 1H), 7.71 (d, J = 1.8 Hz, 1H), 7.48 (dd, J = 8.6, 1.8 Hz, 1H), 4.24 (q, J = 7.1, 2H), 2.38 (s, 3H), 1.27 (t, J = 7.1, 3H); ^{13}C NMR (DMSO-d_6, 100 MHz) δ: 173.3 (s), 167.0 (s), 150.2 (s), 141.0 (s), 127.8 (d), 127.1 (d), 125.9 (s), 124.0 (s), 121.0 (d), 115.7 (s), 60.8 (t), 18.9 (q), 14.6 (q); Anal. Calcd. for $C_{13}H_{12}BrNO_3$: C, 50.34; H, 3.90. Found C, 49.85; H, 3.88.

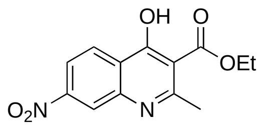

10g
ethyl 4-hydroxy-2-methyl-7-nitroquinoline-3-carboxylate

Ethyl 4-hydroxy-2-methyl-7-nitroquinoline-3-carboxylate (10g): Yellow Solid; mp >260ºC; IR (solid) cm^{-1} 1722; ^{1}H NMR (DMSO-d_6, 400 MHz) δ: 12.32 (s, 1H, O-H), 8.39 (d, J = 2.0 Hz, 1H), 8.28 (d, J = 8.8 Hz, 1H), 8.08 (dd,

J = 8.9, 2.2 Hz, 1H), 4.27 (q, J = 7.1, 2H), 2.44 (s, 3H), 1.29 (t, J = 7.1, 3H); ^{13}C NMR (DMSO-d_6, 100 MHz) δ: 172.9 (s), 166.6 (s), 151.3 (s), 149.8 (s), 139.4 (s), 128.4 (s) 127.9 (d), 117.8 (d), 116.7 (s), 114.7 (d), 61.1 (t), 18.9 (q), 14.6 (q); Anal. Calcd. for $C_{13}H_{12}N_2O_5$: C, 56.52; H, 4.38. Found C, 56.23; H, 4.40.

.

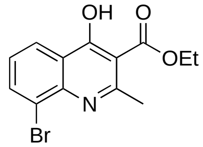

10h
ethyl 8-bromo-4-hydroxy-2-methylquinoline-3-carboxylate

Ethyl 8-bromo-4-hydroxy-2-methylquinoline-3-carboxylate (10h): White Solid; mp 133-135ºC; IR (solid) cm^{-1} 3526, 3400, 1715; ^{1}H NMR (DMSO-d_6, 400 MHz) δ: 10.56 (s, 1H, O-H), 8.12 (dd, J = 8.0, 1.4 Hz, 1H), 8.02 (dd, J = 7.8, 1.4 Hz, 1H), 7.31 (dd, J = 7.8 Hz, 1H), 4.27 (q, J = 7.1, 2H), 2.51 (s, 3H), 1.29 (t, J = 7.1, 3H); ^{13}C NMR (DMSO-d_6, 100 MHz) δ: 173.3 (s), 166.8 (s), 150.4 (s), 137.5 (s), 136.7 (d) 126.6 (s), 125.5 (d), 125.2 (d), 116.5 (s), 111.3 (s), 61.1 (t), 18.7 (q), 14.5 (q); Anal. Calcd. for $C_{13}H_{12}BrNO_3$: C, 50.34; H, 3.90. Found C, 47.63; H, 4.24. The observed elemental analysis is accurate if the compound is calculated as the monohydrate: Anal. Calcd. for $C_{13}H_{12}BrNO_3 \cdot H_2O$: C, 47.58; H, 4.30. Found C, 47.63; H, 4.24.

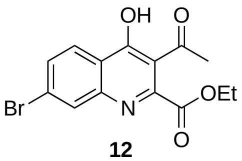

12
ethyl 3-acetyl-7-bromo-4-hydroxyquinoline-2-carboxylate

Eethyl 3-acetyl-7-bromo-4-hydroxyquinoline-2-carboxylate (**12**): A suspension of 7-bromo-1H-benzo[d][1,3]oxazine-2,4-dione (610 mg, 2.5 mmol, 1 equiv) and ethyl sodioacetopyruvate (680 mg, 3.8 mmol, 1.5 equiv) in *N,N*-

dimethylacetamide (5 mL, 0.5 M) was warmed to 60°C for 12 hours. The reaction turned a dark purple during the course of heating. After cooling to ambient temperature the reaction mixture was poured into water (5 mL) and stirred vigorously for 60 minutes. The solid that precipitated was collected by vacuum filtration on a Hirsch funnel then triturated with methanol at ambient temperature. The resulting light tan colored solid was collected by vacuum filtration to afford 755 mg (68%) of the title compound as a tan solid. mp 198-201ºC; IR (solid) cm^{-1} 3500, 1760, 1720; ^{1}H NMR (DMSO-d_6, 400 MHz) δ: 12.63 (br s, 1H, O-H), 8.06 (d, J = 8.6, 1H), 7.98 (d, J = 1.7 Hz, 1H), 7.60 (dd, J = 8.6, 1.7 Hz, 1H), 4.38 (q, J = 7.1, 2H), 2.52 (s, 3H), 1.32 (t, J = 7.2, 3H); ^{13}C NMR (DMSO-d_6, 100 MHz) δ: 199.3 (s), 175.4 (s), 162.9 (s), 141.2 (s), 140.1 (s), 128.6 (d), 127.8 (d), 127.1 (s), 125.6 (s), 122.0 (d), 121.6 (s), 63.4 (t), 31.7 (q), 14.0 (q); Anal. Calcd. for $C_{14}H_{12}BrNO_4$: C, 49.73; H, 3.58. Found C, 49.81; H, 3.47.

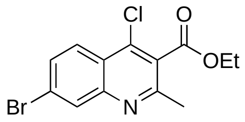

14
ethyl 7-bromo-4-chloro-2-methylquinoline-3-carboxylate

Ethyl 7-bromo-4-chloro-2-methylquinoline-3-carboxylate (14): To a 50 mL single neck round bottom flask charged with ethyl 7-bromo-4-hydroxy-2-methylquinoline-3-carboxylate (1.00 g, 3.2 mmol, 1 equiv) was added phosphorus oxychloride (2.2 mL, 24 mmol, 7 equiv) to give a slurry that rapidly became a homogenous yellow solution. The solution was aged at room temperature for 3 hours then poured into a beaker with ice-water (50 mL) with rapid stirring. The beaker was chilled in an ice-water bath and the pH of the mixture was adjusted to neutral by the addition of solid sodium hydroxide pellets. The aqueous phase was extracted with

dichloromethane (three 25 mL portions). The combined organic phases were washed with saturated sodium chloride (50 mL), dried over anhydrous sodium sulfate, and gravity filtered. The filtrate was concentrated in vacuo and further dried under high vacuum to afford 0.966 g (91%) of the title compound as a pink-white solid. mp >260ºC; IR (solid) cm^{-1} 1720; ^{1}H NMR (DMSO-d_6, 400 MHz) δ: 8.14 (d, J = 1.9 Hz, 1H), 7.94 (d, J = 8.9 Hz, 1H), 7.61 (dd, J = 8.9, 1.9 Hz, 1H), 4.52 (q, J = 7.2, 2H), 2.71 (s, 3H), 1.46 (t, J = 7.2, 3H); ^{13}C NMR (DMSO-d_6, 100 MHz) δ: 165.9 (s), 156.2 (s), 148.0 (s), 139.6 (s), 131.1 (d), 130.9 (d), 127.6 (s), 125.7 (s), 125.6 (s), 122.7 (s), 62.4 (t), 23.6 (q), 14.1 (q); Anal. Calcd. for $C_{13}H_{11}BrClNO_2$: C, 47.52; H, 3.37. Found C, 44.86; H, 3.23. The observed elemental analysis is accurate if the compound is calculated as the monohydrate: Anal. Calcd. for $C_{13}H_{11}BrClNO_2 \cdot H_2O$: C, 45.05; H, 3.78. Found C, 44.86; H, 3.23.

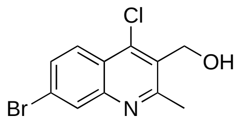

SI-1
(7-bromo-4-chloro-2-methylquinolin-3-yl)methanol

(7-Bromo-4-chloro-2-methylquinolin-3-yl)methanol (SI-1): A solution of the ethyl 7-bromo-4-chloro-2-methylquinoline-3-carboxylate (1.27 g, 3.86 mmol, 1 equiv) in dichloromethane (13 mL, 0.3 M) was cooled to an internal temperature of −70 °C in a dry ice-acetone bath. Diisobutylaluminum hydride (1.6 mL of 1M in CH_2Cl_2, 11.6 mmol, 3 equiv) was added dropwise via syringe. The cooling bath was removed and the reaction mixture warmed to room temperature. After 2 hours, the reaction was quenched by the sequential addition of water (10 mL), aqueous sodium hydroxide (20 mL of 1 M), and then water (10 mL) with stirring overnight until clear

phase separation was observed. The phases were cut and the aqueous phase extracted with dichloromethane (three 25 mL portions). The combined chlorocarbon phases were dried over anhydrous magnesium sulfate, gravity filtered and concentrated in vacuo. The crude residue was purified by flash chromatography over an ISCO CombiFlash 12 gram normal phase silica gel column eluted with a gradient of hexanes-ethyl acetate. The product rich fractions were pooled and concentrated in vacuo to afford 776 mg (70%) the title compound as a white solid. mp 161-162ºC; IR (solid) cm^{-1} 3050; ^{1}H NMR (DMSO-d_6, 400 MHz) δ: 8.17 (d, J = 1.8 Hz, 1H), 8.06 (d, J = 8.9 Hz, 1H), 7.66 (dd, J = 8.9, 1.8 Hz, 1H), 5.02 (s, 2H), 2.87 (s, 3H), 2.30 (br s, 1H); ^{13}C NMR (DMSO-d_6, 100 MHz) δ: 161.0 (s), 147.9 (s), 142.7 (s), 130.7 (d), 130.5 (d), 130.1 (s), 126.1 (d), 124.8 (s), 124.1 (s), 59.2 (t), 23.4 (q).

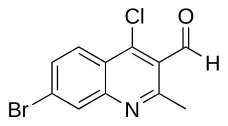

15
7-bromo-4-chloro-2-methylquinoline-3-carbaldehyde

7-Bromo-4-chloro-2-methylquinoline-3-carbaldehyde (15): A solution of the (7-bromo-4-chloro-2-methylquinolin-3-yl)methanol (1.67 g, 5.86 mmol, 1 equiv) in dimethyl sulfoxide (20 mL, 0.3 M) chilled in an ice-water bath was added triethylamine (4.89 mL, 35.1 mmol, 6 equiv) dropwise via syringe. After 15 minutes, pyridine sulfur trioxide complex (6.52 g, 41 mmol, 7 equiv) was added in small portions and the cooling bath removed. After stirring at room temperature for 1 hour, the reaction was deemed to be complete. The reaction mixture was partitioned between with water (50 mL) and dichloromethane (50 mL). The phases were split and the aqueous phase extracted with dichloromethane (two 25 mL portions). The combined dichloromethane extracts were washed with brine (five 50 mL portions), dried over

anhydrous sodium sulfate, and gravity filtered. The filtrate was concentrated in vacuo to an oil that was purified by automated flash chromatography on the ISCO CombiFlash over a 40 gram column eluted with a gradient of hexanes ethyl acetate. The product rich fractions were pooled and concentrated in vacuo to afford 1.232 g (74%) of the title compound as an off-white solid. mp 141-145ºC (decomposed); IR (solid) cm^{-1} 1736; ^{1}H NMR (DMSO-d_6, 400 MHz) δ: 10.81 (s, 1H), 8.22 (d, J = 1.8 Hz, 1H), 8.18 (d, J = 8.9, 1H), 7.73 (dd, J = 9.0, 1.9, 1H), 2.92 (s, 3H); ^{13}C NMR (DMSO-d_6, 100 MHz) δ: 190.9 (d), 159.9 (s), 149.3 (s), 149.2 (s), 131.7 (d), 131.3 (d), 128.0 (s), 125.9 (d), 124.6 (s), 123.6 (s), 25.4 (q); Anal. Calcd. for $C_{11}H_7ClBrNO$: C, 46.43; H, 2.48. Found C, 45.72; H, 2.41.

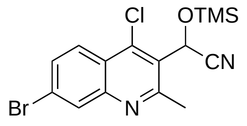

16
2-(7-bromo-4-chloro-2-methylquinolin-3-yl)-2-((trimethylsilyl)oxy)acetonitrile

2-(7-Bromo-4-chloro-2-methylquinolin-3-yl)-2-((trimethylsilyl)oxy)acetonitrile (16): A 25 mL single neck round bottom flask with stirbar was charged with anhydrous lithium chloride (30 mg, .0708 mmol, 1 equiv). The vessel and contents were flame dried under vacuum and cooled under nitrogen atmosphere. A solution of the 7-bromo-4-chloro-2-methylquinoline-3-carbaldehyde (200 mg, 0.70 mmol, 1 equiv) in tetrahydrofuran (3.5 mL, 0.2 M) was added and stirring commenced for 15 minutes at ambient temperature prior to the introduction of trimethylsilyl cyanide (106 μL, 0.848 mmol, 1.2 equiv) via Hamilton gas-tight syringe. The reaction mixture was stirred at ambient temperature for 6 hours before the aldehyde was fully consumed. The reaction was quenched by the addition of

saturated aqueous sodium bicarbonate (10 mL) and diluted with dichloromethane (10 mL). The phases were split and the aqueous phase extracted with dichloromethane (three 10 mL portions). The combined organic phases were washed with brine (25 mL), dried over anhydrous magnesium sulfate, and gravity filtered. The filtrate was concentrated in vacuo leaving 267 mg (99%) of title compound as a colorless residue that was sufficiently pure to carry forward without purification. IR (solid) cm^{-1} 2255; ^{1}H NMR (DMSO-d_6, 400 MHz) δ: 8.22 (s, 1H), 8.05 (d, J = 8.9 Hz, 1H), 7.73 (d, J = 8.9 Hz, 1H), 6.45 (s, 1H), 3.05 (s, 3H), 0.25 (s, 9H); ^{13}C NMR (DMSO-d_6, 100 MHz) δ: 160.4 (s), 148.7 (s), 141.7 (s), 131.8 (d), 131.4 (d), 126.7 (d), 126.4 (s), 126.2 (s), 123.5 (s), 118.0 (s), 59.8 (d), 24.7 (q), 0.0 (q).

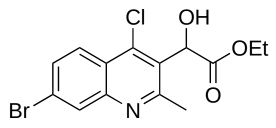

19
ethyl 2-(7-bromo-4-chloro-2-methylquinolin-3-yl)-2-hydroxyacetate

Ethyl 2-(7-bromo-4-chloro-2-methylquinolin-3-yl)-2-hydroxyacetate (19): To a solution of 2-(7-bromo-4-chloro-2-methylquinolin-3-yl)-2-((trimethylsilyl)oxy)acetonitrile (137 mg, 0.476 mmol, 1 equiv) in ethanol (240 μL) cooled in an ice-water bath was added hydrogen chloride (1.5 mL of 3.3 M in ethanol, 4.95 mmol, 10.4 equiv). The solution was warmed to ambient temperature and stirred for 2 hours at which time the cyanide had been consumed. The solvent was evaporated and the crude residue shown by ^{1}H NMR to be the ethyl 2-(7-bromo-4-chloro-2-methylquinolin-3-yl)-2-hydroxyacetimidate that was immediately subjected to hydrolysis with aqueous hydrochloric acid (5 mL of 1 M, 5 mmol, 10.5 equiv) at ambient temperature. After 1 hour, the vessel was cooled in an ice-water bath and the pH of the reaction mixture adjusted to 7 by the dropwise addition of saturated

aqueous sodium bicarbonate. The aqueous phase was extracted with dichloromethane (three 15 mL portions). The combined organic phases were washed with brine (20 mL), dried over anhydrous sodium sulfate, and gravity filtered. The filtrate was concentrated in vacuo leaving 77 mg (59%) of the title compound as a colorless residue. The material was sufficiently pure to not require further purification. IR (solid) cm^{-1} 3400, 1756; ^{1}H NMR (DMSO-d_6, 400 MHz) δ: 7.98 (d, J = 8.7 Hz, 1H), 7.70 (d, J = 1.7 Hz, 1H), 7.46 (dd, J = 8.6, 1.9 Hz, 1H), 5.45 (s, 1H), 4.07 (q, J = 7.2, 2H), 2.41 (s, 3H), 1.11 (t, J = 7.1, 3H); ^{13}C NMR (DMSO-d_6, 100 MHz) δ: 174.9 (s), 173.8 (s), 149.5 (s), 140.4 (s), 128.0 (d), 126.7 (d), 125.5 (s), 123.1 (s), 120.3 (d), 119.0 (s), 65.4 (d), 60.6 (t), 17.8 (q), 14.6 (q).

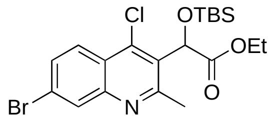

21

ethyl 2-(7-bromo-4-chloro-2-methylquinolin-3-yl)-2-((*tert*-butyldimethylsilyl)oxy)acetate

2-(7-Bromo-4-chloro-2-methylquinolin-3-yl)-2-((*tert*-butyldimethylsilyl)oxy)acetonitrile (21): To a 5 mL conical vial was added 7-bromo-4-chloro-2-methylquinoline-3-carbaldehyde (28 mg, 0.1 mmol), 2-((tert-butyldimethylsilyl)oxy)malononitrile (84 mg, 0.2 mmol), diethyl ether (2.0 mL), and ethanol (17 µL, 0.3 mmol). The solution was cooled to 0ºC in an ice bath. *N*,*N*-dimethylaminopyridine (12 mg, 0.1 mmol) was then added to the solution and the reaction was stirred at 0 ºC for one hour. The reaction was warmed to ambient temperature and concentrated under house air to afford a yellow oil/solid mixture (24 mg). The residue was chromatographed over 4 g of silica gel eluted with hexanes-acetone (25 mL 100% hexanes→25 mL 9:1) in 3 mL fractions. Product

rich fractions were pooled and evaporated to afford ethyl 2-(7-bromo-4-chloro-2-methylquinolin-3-yl)-2-((tert-butyldimethylsilyl)oxy)acetate (36 mg, 76 %) as a clear, colorless oil. IR (Thin Film) cm^{-1} 2921, 1755, 1734; ^{1}H NMR ($CDCl_3$, 400 MHz) δ: 8.19 (d, J = 1.8 Hz, 1H), 8.09 (dd, J = 8.9, 1.9 Hz, 1H), 7.67 (d, J = 8.9 Hz, 1H), 6.09 (s, 1H), 4.20 (q, J = 7.1 Hz, 2H), 2.75 (s, 3H), 1.20 (t, J = 7.1 Hz, 3H), 0.88 (s, 9H), 0.21 (s, 3H), -0.01 (s, 3H); ^{13}C NMR ($CDCl_3$, 100 MHz) δ: 171.4 (s), 160.7 (s), 148.0 (s), 141.7 (s), 131.3 (d) 130.6 (d), 130.1 (s), 126.5 (d), 124.9 (s), 123.7 (s), 70.6 (d), 61.9 (t), 25.8 (q), 24.2 (q), 18.3 (s), 14.2 (q), -4.8 (q), -5.0 (q); HRMS (ESI+): Exact mass calcd for $C_{20}H_{27}BrClNO_3Si$ $[M+H]^+$, 472.070487. Found 472.070663.
