# Supplementary material for: Quinolines from the cyclocondensation of isatoic anhydride with ethyl acetoacetate: preparation of ethyl 4-hydroxy-2-methylquinoline-3-carboxylate and derivatives
Source: Beilstein J Org Chem. 2018 Sep 28;14:2529–36. doi: 10.3762/bjoc.14.229 (PMC6178280; doi:10.3762/bjoc.14.229)
Supplement: File 2 — IR, NMR and mass spectra, as well as elemental analyses. [file Beilstein_J_Org_Chem-14-2529-s002.pdf]

Supporting Information

for

Quinolines from the cyclocondensation of isatoic anhydride with ethyl acetoacetate: preparation of ethyl 4-hydroxy-2-methylquinoline-3-carboxylate and derivatives

Nicholas G. Jentsch, Jared D. Hume, Emily B. Crull, Samer M. Beauti, Amy H. Pham, Julie A. Pigza, Jacques J. Kessl and Matthew G. Donahue*

Address: Department of Chemistry and Biochemistry, University of Southern Mississippi, 118 College Drive #5043, Hattiesburg, MS 39406

Email: Matthew G. Donahue – matthew.donahue@usm.edu

* Corresponding author

IR, NMR and mass spectra, as well as elemental analyses

AHP-1-017

Collection time: Fri Jul 28 14:23:05 2017 (GMT-06:00)

Number of sample scans: 8
Number of background scans: 8
Resolution: 2.000
Sample gain: 8.0
Mirror velocity: 0.6329
Aperture: 100.00

9a

2*H*-benzo[*d*][1,3]oxazine-2,4(1*H*)-dione

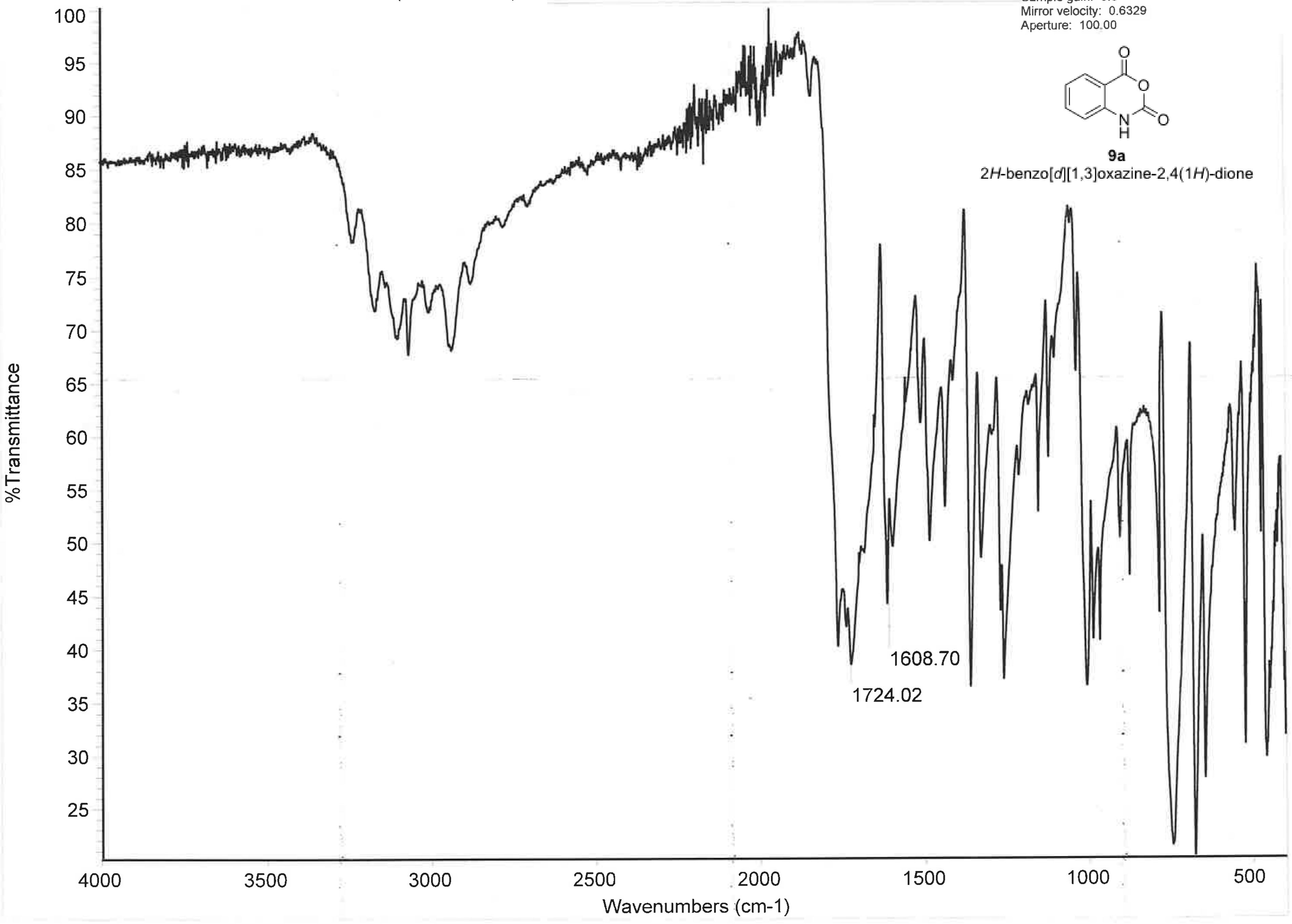

AHP-1-017 (DMSO, 400 MHz) Pure White Solid- Isatoic anhydride synthesis

NAME	AHP-1-017
EXPNO	20
PROCNO	1
Date_	20161202
Time	19.50 h
INSTRUM	spect
PROBHD	Z108618_0161 (
PULPROG	zg30
TD	65536
SOLVENT	DMSO
NS	16
DS	2
SWH	8012.820 Hz
FIDRES	0.244532 Hz
AQ	4.0894966 sec
RG	128
DW	62.400 usec
DE	6.50 usec
TE	298.2 K
D1	1.00000000 sec
TD0	1
SFO1	400.1724710 MHz
NUC1	1H
P1	29.07 usec
SI	65536
SF	400.1699945 MHz
WDW	EM
SSB	0
LB	0.30 Hz
GB	0
PC	1.00

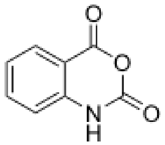

9a

2*H*-benzo[*d*][1,3]oxazine-2,4(1*H*)-dione

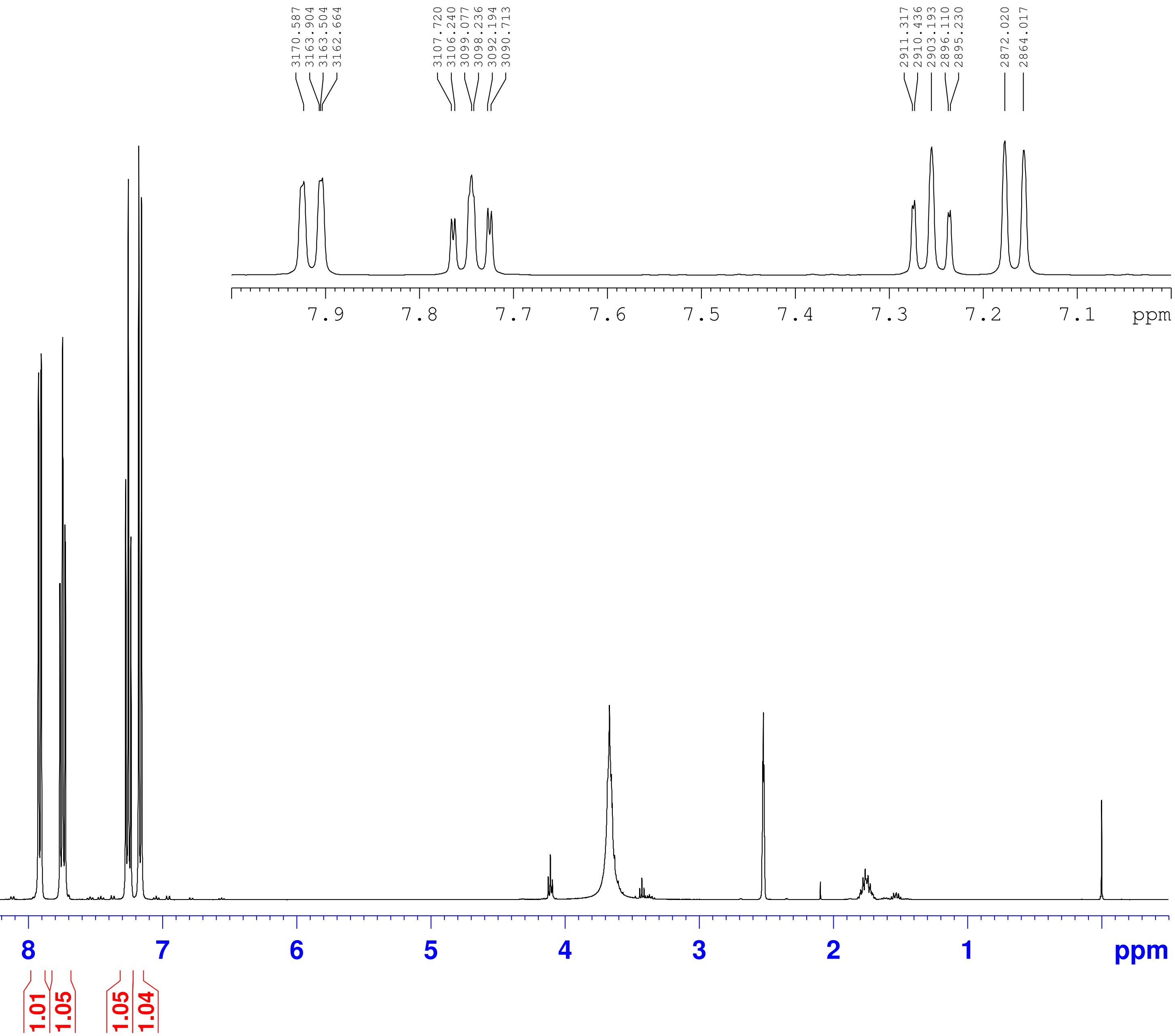

AHP-1-017 (DMSO, 400 MHz) Pure White Solid- Isatoic anhydride

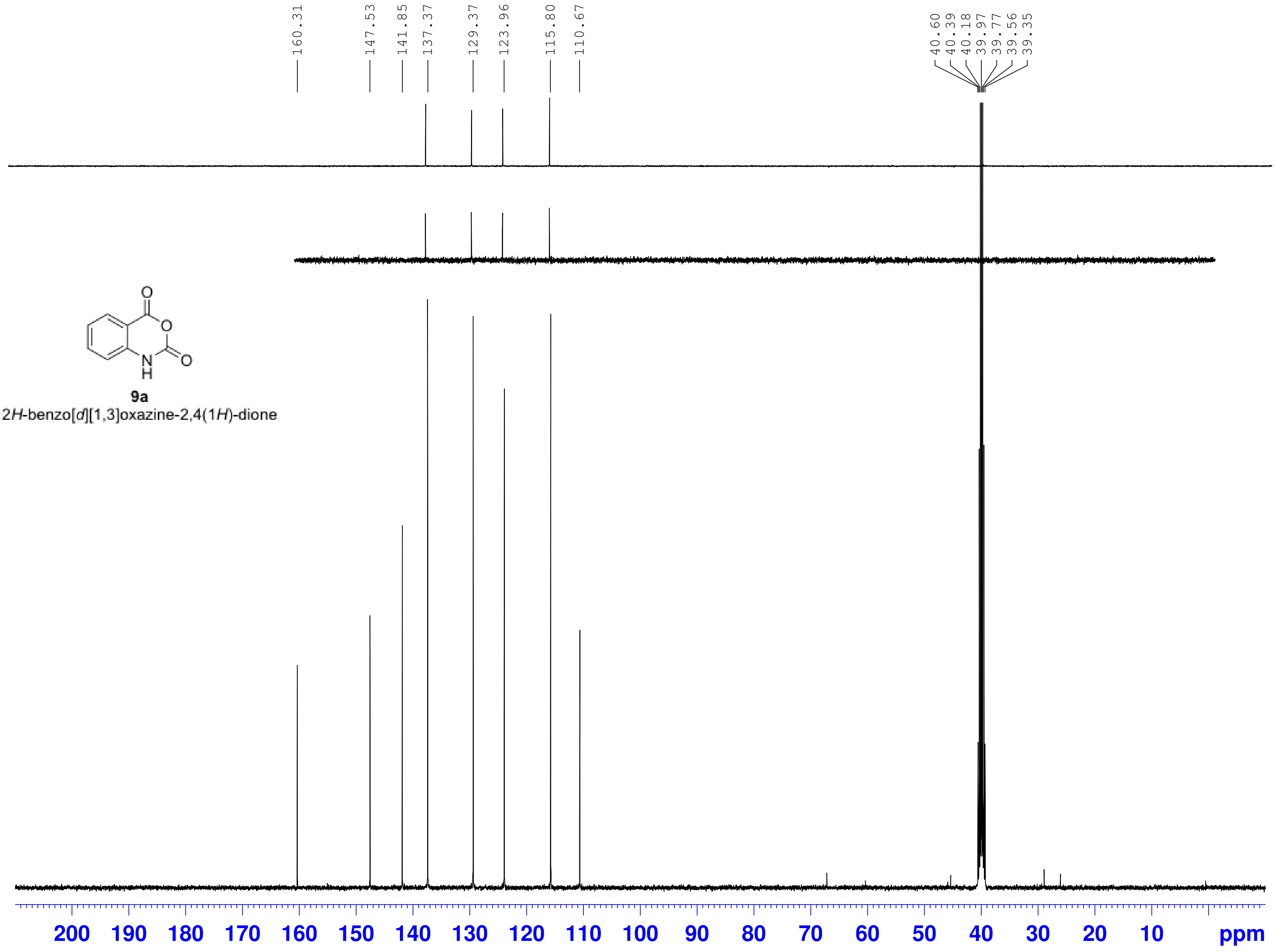

9a

2*H*-benzo[*d*][1,3]oxazine-2,4(1*H*)-dione

AHP-1-017 (DMSO, 400 MHz) Pure White Solid- Isatoic anhydride synthesis

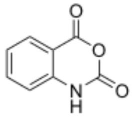

9a

2*H*-benzo[*d*][1,3]oxazine-2,4(1*H*)-dione

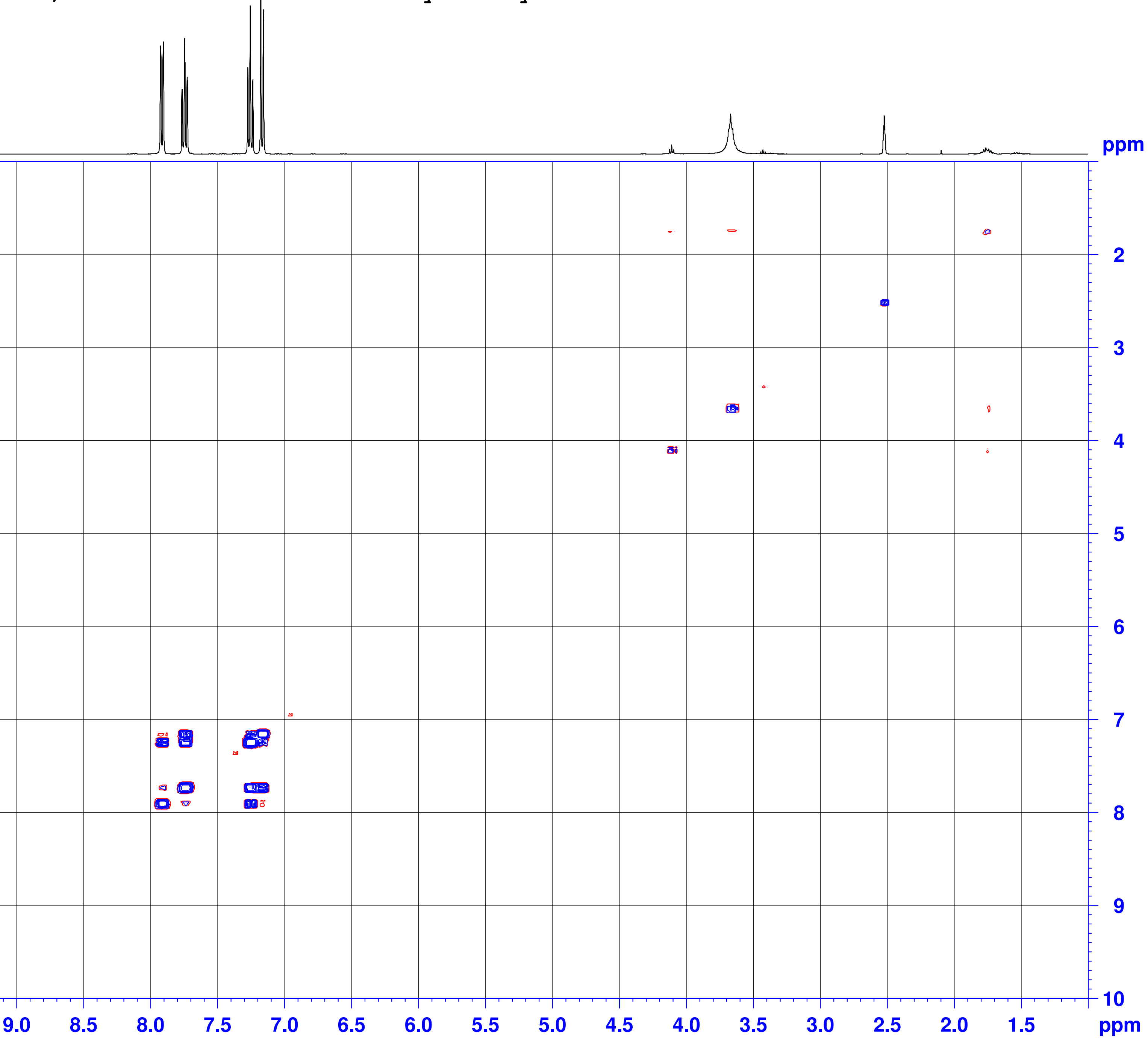

AHP-1-017 (DMSO, 400 MHz) Pure White Solid- Isatoic anhydride synthesis

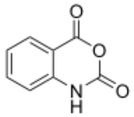

9a

2*H*-benzo[*d*][1,3]oxazine-2,4(1*H*)-dione

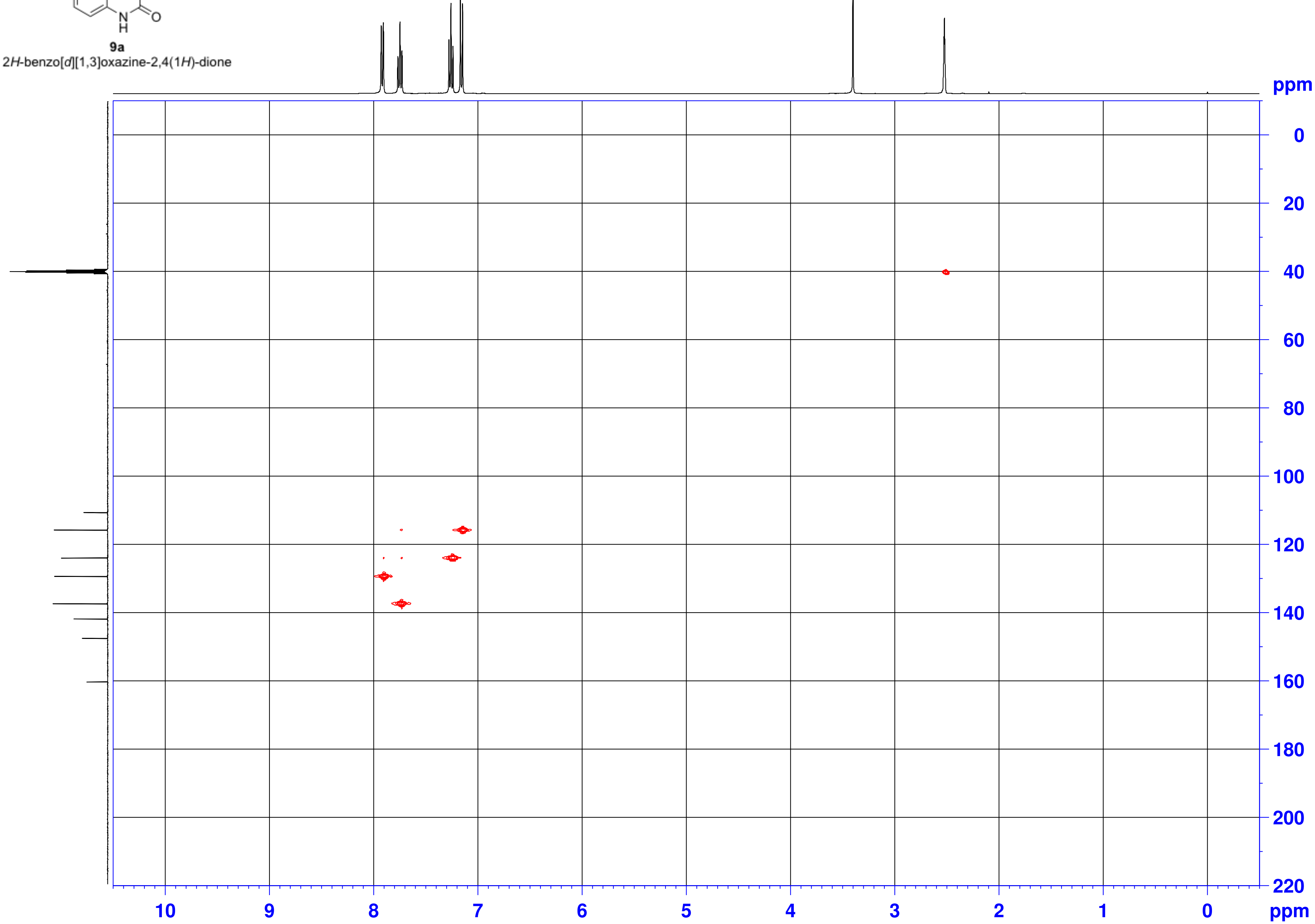

AHP-1-017 (DMSO, 400 MHz) Pure White Solid- Isatoic anhydride synthesis

9a

2*H*-benzo[*d*][1,3]oxazine-2,4(1*H*)-dione

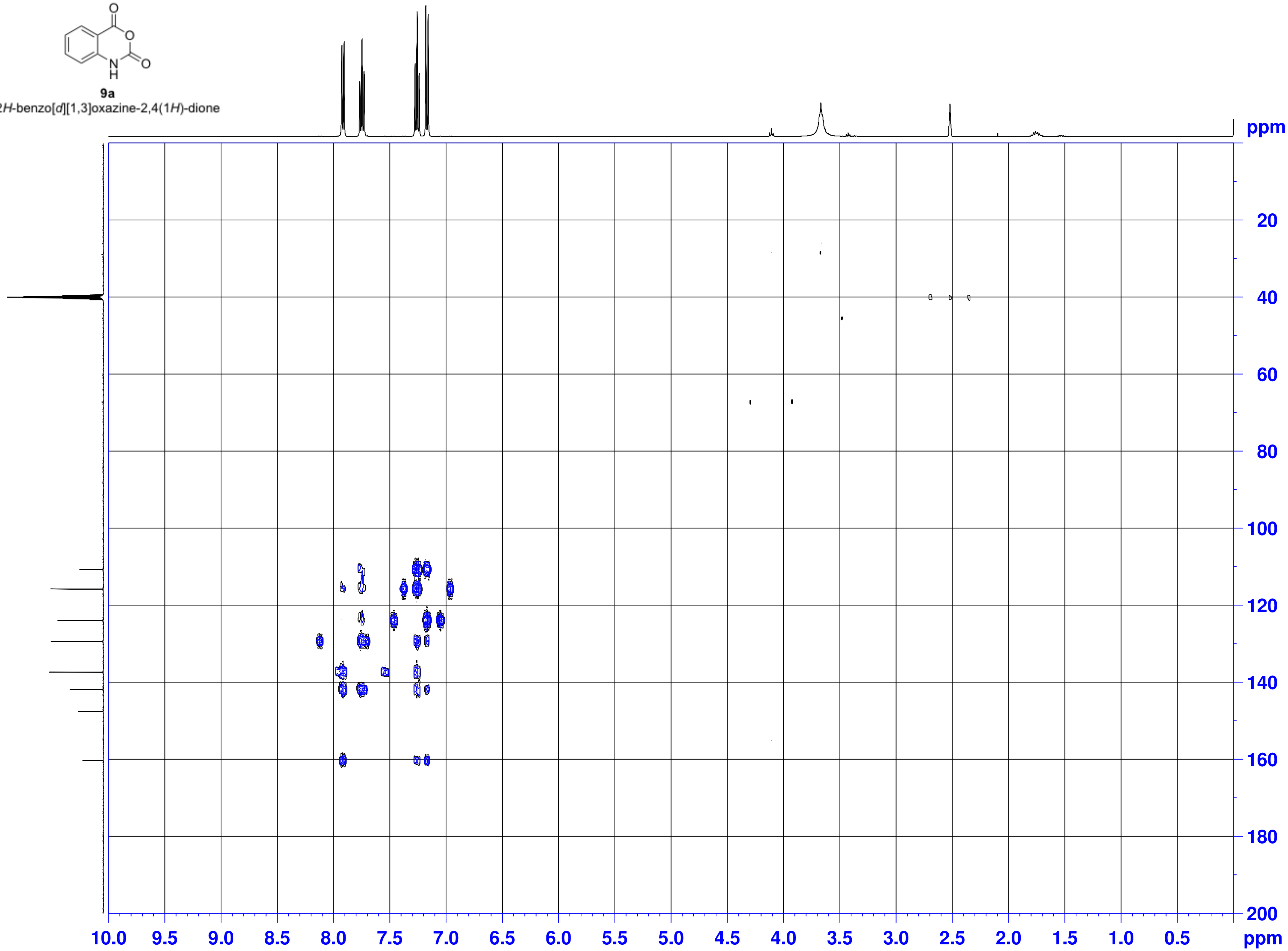

SMB-1-012

Collection time: Fri Jul 28 14:29:49 2017 (GMT-06:00)

Number of sample scans: 8
Number of background scans: 8
Resolution: 2.000
Sample gain: 8.0
Mirror velocity: 0.6329
Aperture: 100.00

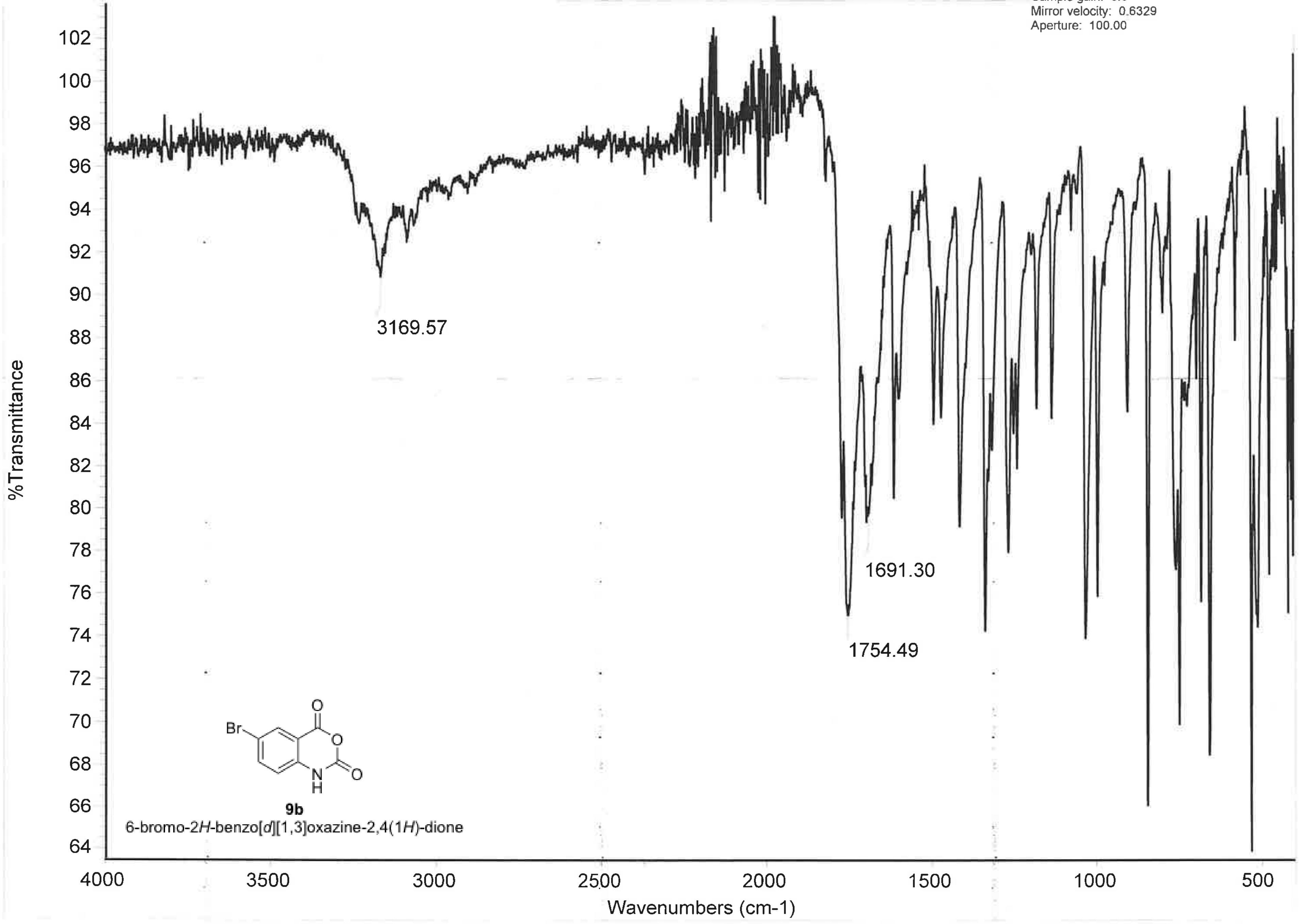

SMB-1-012 (DMSO, 400 MHz) Isotoic anhydride
PROTON DMSO {C:\Bruker\TOPSPIN} Donahue 1

NAME	SMB-1-012
EXPNO	50
PROCNO	1
Date_	20161022
Time	12.55
INSTRUM	spect
PROBHD	5 mm PABBO BB-
PULPROG	zg30
TD	65536
SOLVENT	DMSO
NS	16
DS	2
SWH	8223.685 Hz
FIDRES	0.125483 Hz
AQ	3.9846387 sec
RG	203
DW	60.800 usec
DE	6.50 usec
TE	299.1 K
D1	1.00000000 sec
TD0	1

======== CHANNEL f1 ========

NUC1	1H
P1	10.20 usec
PL1	-5.00 dB
PL1W	25.65680122 W
SFO1	400.1724712 MHz
SI	32768
SF	400.1699980 MHz
WDW	EM
SSB	0
LB	0.30 Hz
GB	0
PC	1.00

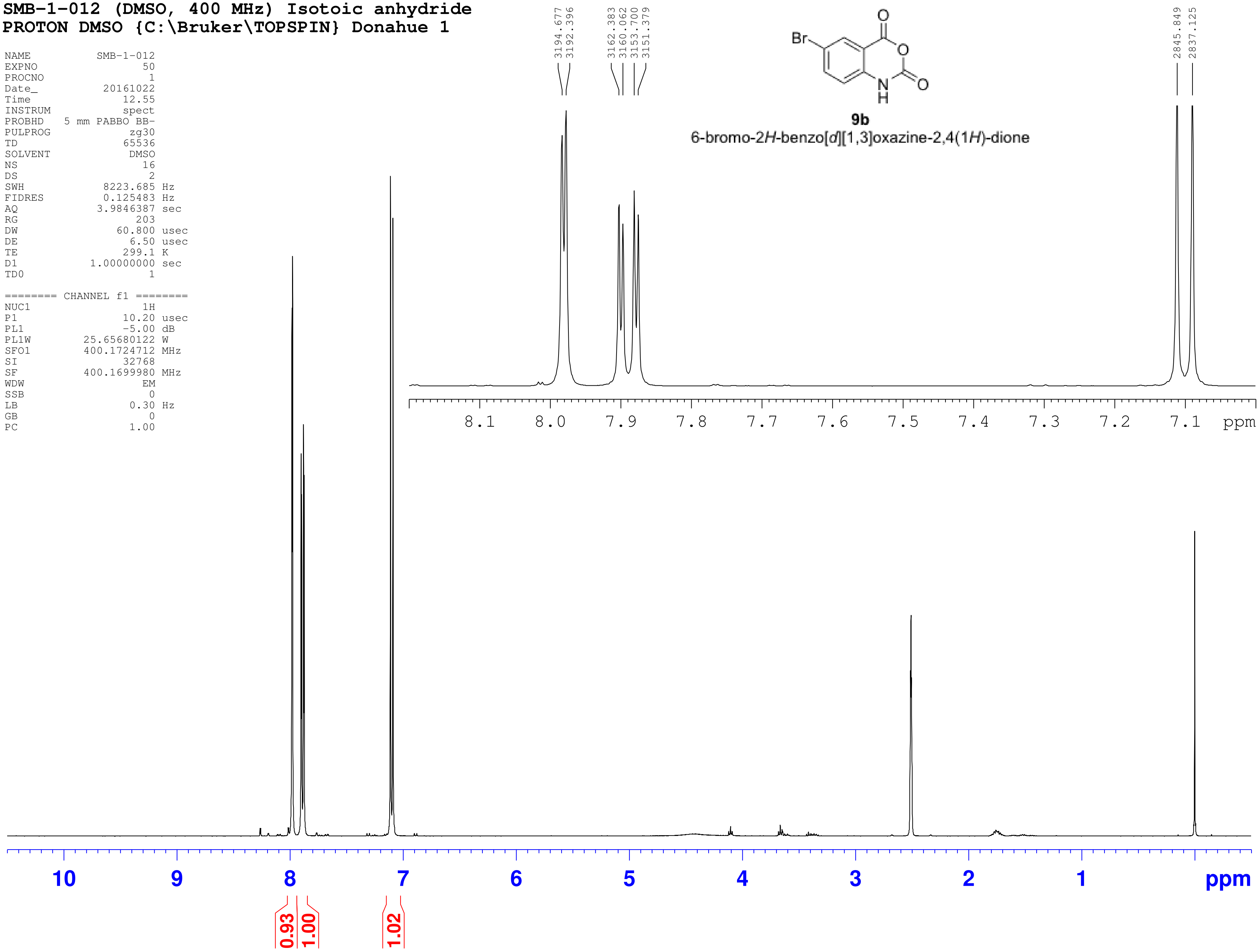

9b
6-bromo-2*H*-benzo[*d*][1,3]oxazine-2,4(1*H*)-dione

SMB-1-012 (DMSO, 400 MHz) Isotoic anhydride
C13CPD DMSO {C:\Bruker\TOPSPIN} Donahue 1

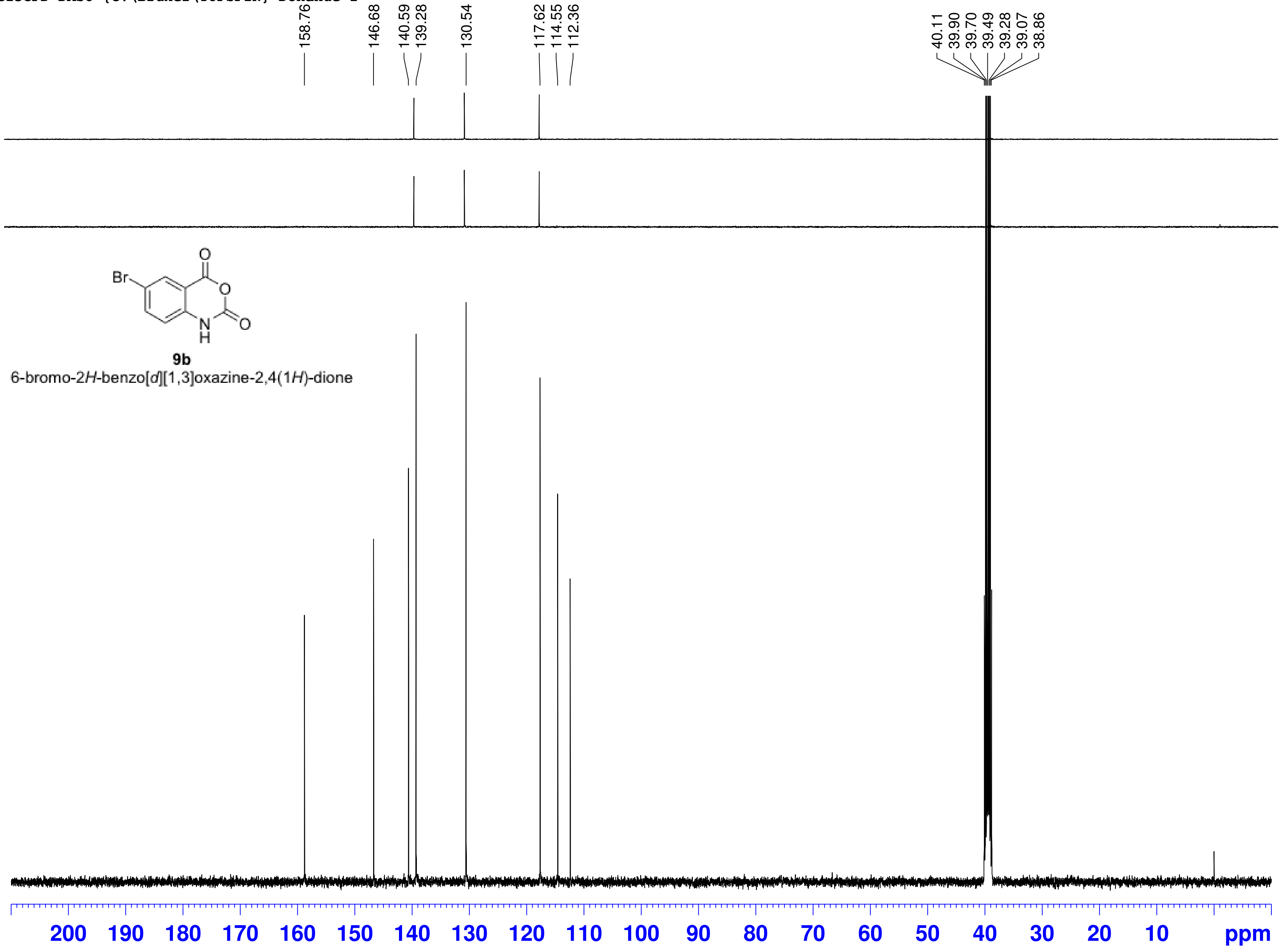

SMB-1-012 (DMSO, 400 MHz)

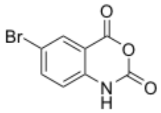

9b

6-bromo-2*H*-benzo[*d*][1,3]oxazine-2,4(1*H*)-dione

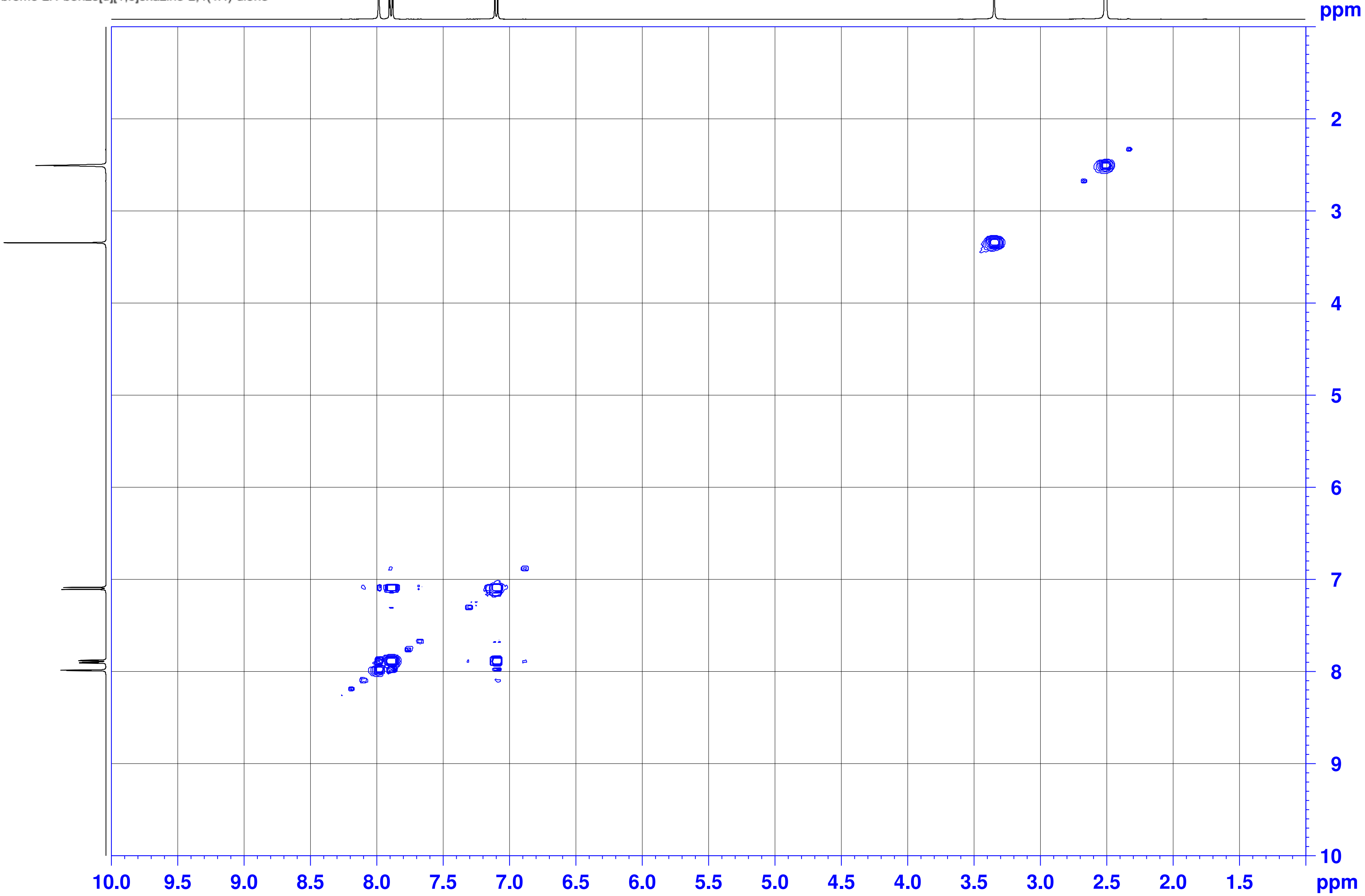

SMB-1-012 (DMSO, 400 MHz)

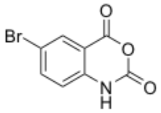

9b

6-bromo-2*H*-benzo[*d*][1,3]oxazine-2,4(1*H*)-dione

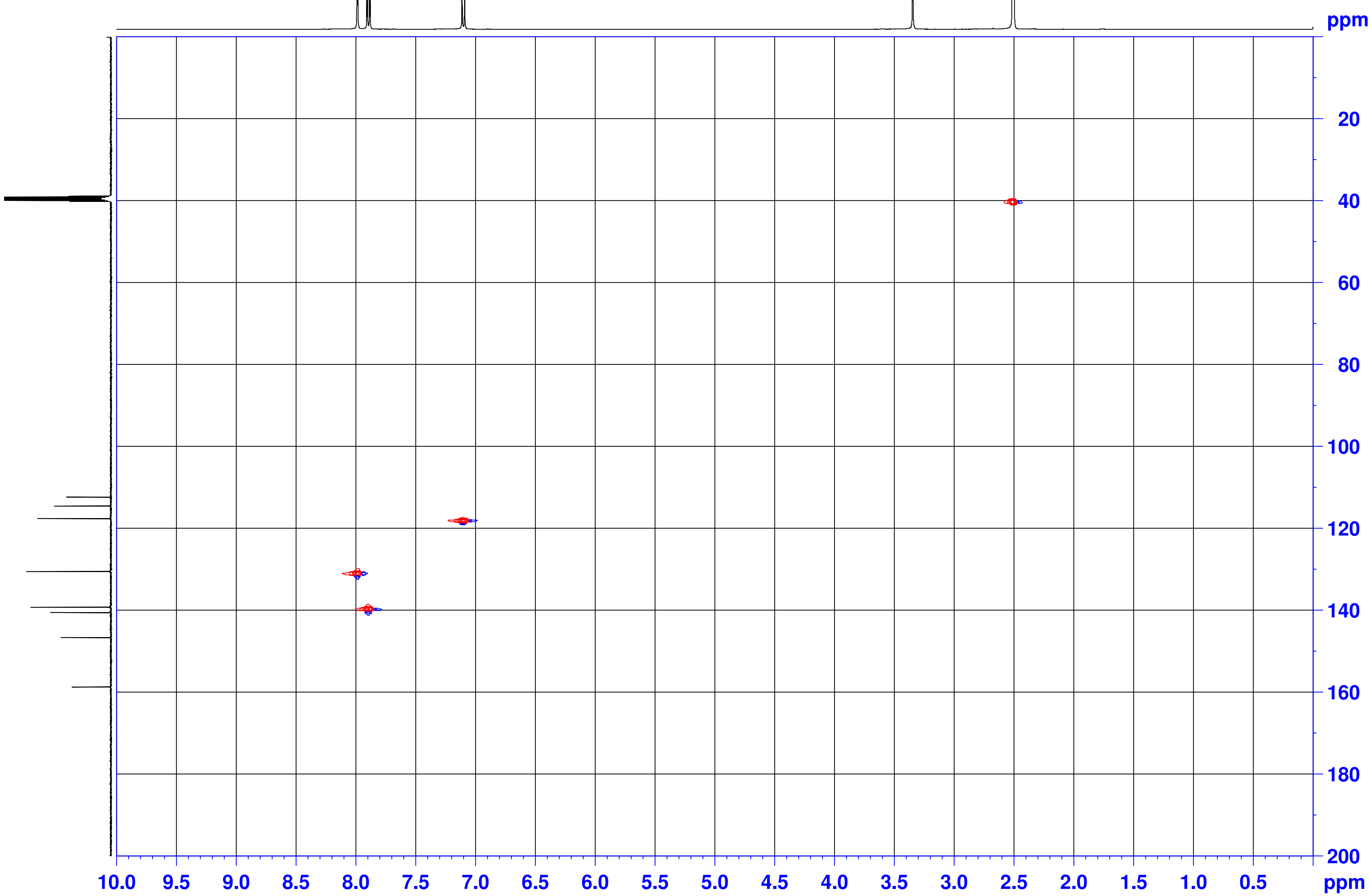

SMB-1-012 (DMSO, 400 MHz)

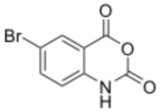

9b

6-bromo-2*H*-benzo[*d*][1,3]oxazine-2,4(1*H*)-dione

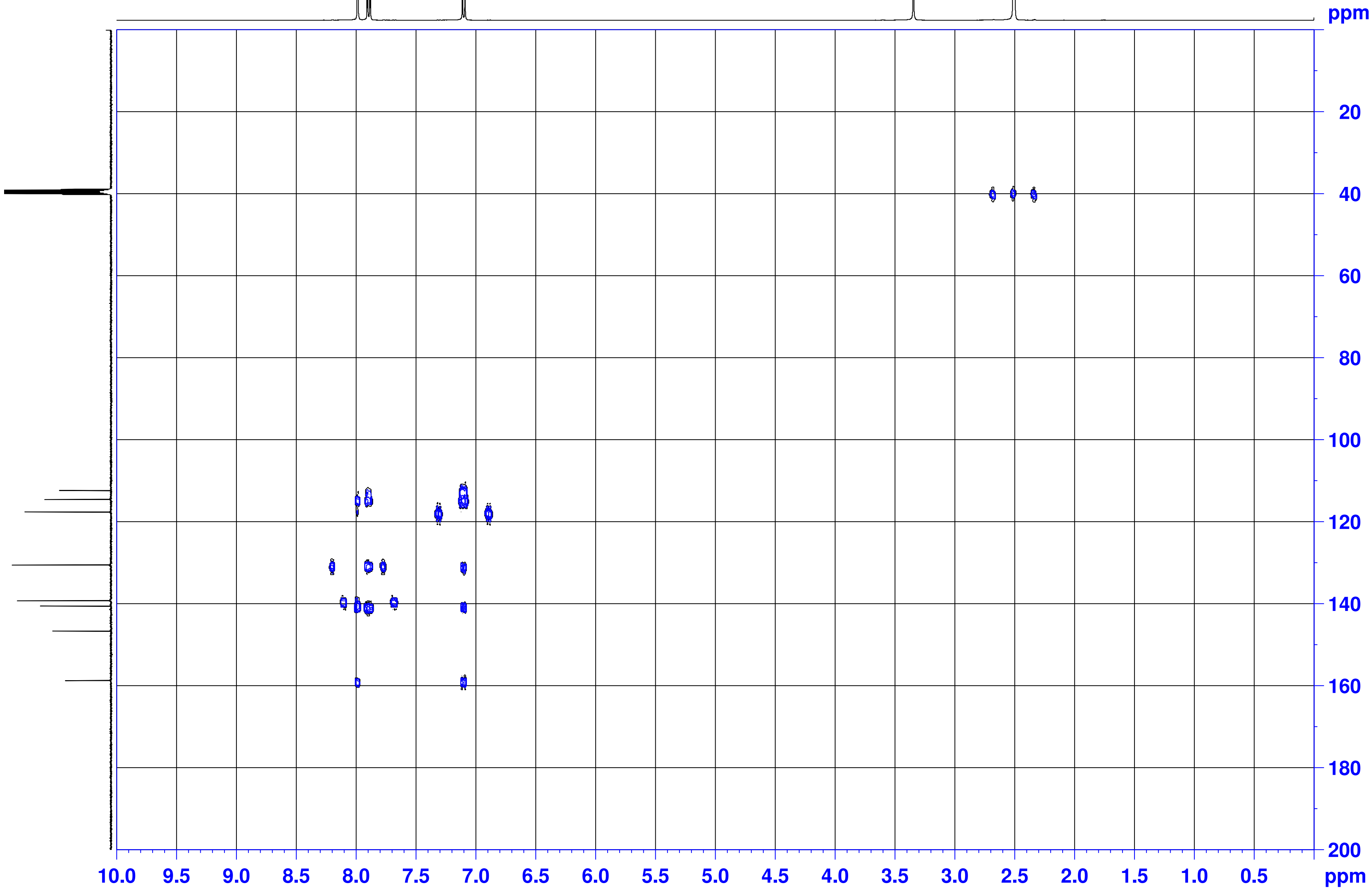

Mass Spectrum List Report

Analysis Info

Analysis Name	G:\Data\apexdata111116\SMB-1-012_pos_000006.d	Acquisition Date	11/11/2016 3:45:13 PM
Method	Neg_DOM_032112	Operator	FTMS_USER
Sample Name	SMB-1-012	Instrument	apex-Qe
Comment	SMB-1-012 in MeOH:H2O with NaCl added		

Sample Name SMB-1-012

Exact Mass of C8H4BrNO3Na+ = 263.926677 m/z

Mass Observed with signature = 263.926475 m/z

Difference < -1.0 ppm

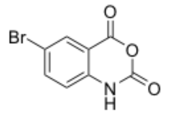

9b

6-bromo-2*H*-benzo[*d*][1,3]oxazine-2,4(1*H*)-dione

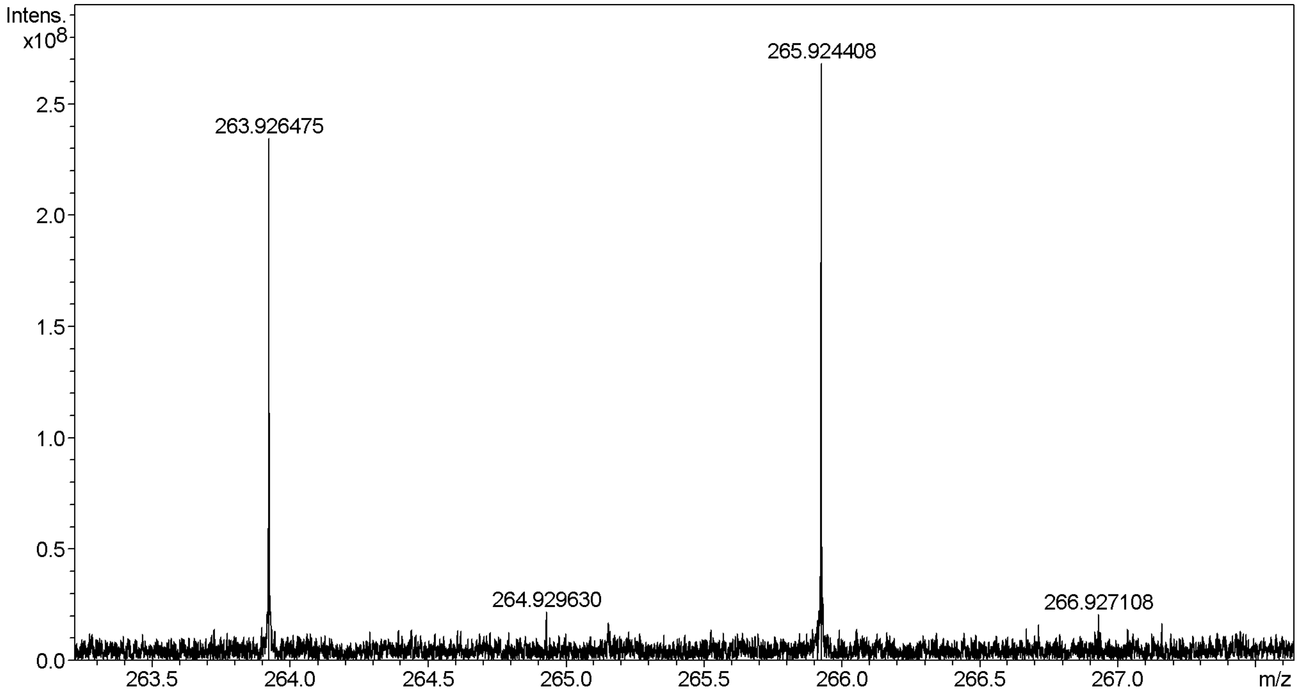

#	m/z	I
1	184.248996	29083902
2	184.318093	42775166
3	184.319018	39766398
4	209.440134	27024405
5	223.933903	27067184
6	241.944033	33785451
7	263.926475	234262671
8	265.924408	267869301
9	281.172242	25188311
10	296.955403	37178465
11	297.954288	59439899
12	298.952932	26643926
13	301.140721	34136216
14	304.961966	41642600
15	305.961179	27247913
16	309.203187	39712525
17	313.967500	36685598
18	317.910440	33984901
19	317.945622	27168194
20	321.956222	34530426

Mass Spectrum List Report

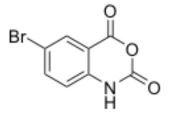

9b
6-bromo-2*H*-benzo[*d*][1,3]oxazine-2,4(1*H*)-dione

#	m/z	I
21	322.271327	41936530
22	323.182675	26556575
23	333.987027	32572363
24	339.177670	32285085
25	350.302566	26442052
26	351.214016	27704086
27	351.250310	25128785
28	351.966230	83996642
29	353.265909	52176936
30	353.964224	73075865
31	365.135518	34454264
32	365.229512	32318350
33	367.943724	34958455
34	368.804750	45575632
35	368.812119	28999644
36	368.819573	57075176
37	368.823127	50369005
38	368.826517	48318963
39	368.834408	184769024
40	368.837952	199592453
41	368.841638	82524683
42	368.845408	104487441
43	368.856624	44488227
44	368.867210	38727731
45	369.413982	36109720
46	369.941703	25207005
47	369.976812	67948821
48	371.974779	92032369
49	379.245097	29819410
50	381.297204	86464145
51	383.402055	43903831
52	384.401102	40925539
53	393.260560	26089097
54	393.297105	233443009
55	394.300451	51389624
56	395.240082	36698700
57	398.241098	68809740
58	407.276348	29357280
59	413.265844	81097572
60	422.008061	29705479
61	425.286961	32267113
62	494.865483	103637377
63	494.885708	56770967
64	495.367255	39707545
65	495.864406	170575274
66	496.366617	58460094
67	496.863385	92670411
68	497.365017	34173915
69	497.862464	29160933
70	498.880227	36538891
71	500.878633	28109338
72	502.854185	50151924
73	503.853222	84403680
74	504.354939	38408150
75	504.852157	51472837
76	506.861490	32530819
77	512.896085	65742849
78	514.894159	34371377
79	520.828697	62702532
80	522.826694	112134765
81	522.861755	143198858
82	523.830060	31401399
83	524.824427	58081522
84	524.859833	304681232

Mass Spectrum List Report

#	m/z	I
85	525.287556	25661040
86	525.862437	49669191
87	526.857636	152803184
88	527.860661	32619158
89	540.872291	70995998
90	542.870335	161711445
91	543.872967	35436517
92	544.868162	90540647
93	569.313656	27266344
94	594.901605	25363706
95	609.308266	26271211
96	655.351381	26094454
97	718.384106	26188354
98	747.786383	57826085
99	749.783764	61325943
100	1675.785499	34323639

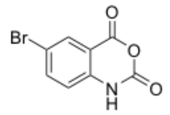

9b
6-bromo-2*H*-benzo[*d*][1,3]oxazine-2,4(1*H*)-dione

Atlantic Microlab, Inc.

Sample No. SMB-1-012

6180 Atlantic Blvd. Suite M
Norcross, GA 30071
www.atlanticmicrolab.com

Company/School University of Southern Mississippi
Dept. Chemistry and Biochemistry
Address 118 College Dr. #5043
City, State, Zip Hattiesburg, MS 39406
Name Matthew Donahue Date 10/25/16
Phone 614-203-1123

Professor/Supervisor: Matthew G. Donahue
PO# / CC# Mastercard 9359 (On file)

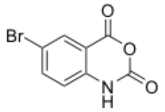

9b

6-bromo-2*H*-benzo[*d*][1,3]oxazine-2,4(1*H*)-dione

Element	Theory	Found	
C	39.70	39.51	
H	1.67	1.74	

Single ☒ Duplicate ☐

Elements Present:

Analyze for: C, H

Hygroscopic ☐ Explosive ☐

M.P. ______ B.P. ______

To be dried: Yes ☐ No ☒

Temp. ______ Vac. ______ Time ______

Rush Service ☐ Rush service guarantees analyses will be completed and results available by 5 PM EST on the day the sample is received by 11 AM.

Include Email Address or FAX # Below

matthew.donahue@usm.edu

Date Received NOV 03 P.M. Date Completed NOV [illegible]

Remarks:

AHP-1-018

Collection time: Fri Jul 28 14:34:36 2017 (GMT-06:00)

Number of sample scans: 8
Number of background scans: 8
Resolution: 2.000
Sample gain: 8.0
Mirror velocity: 0.6329
Aperture: 100.00

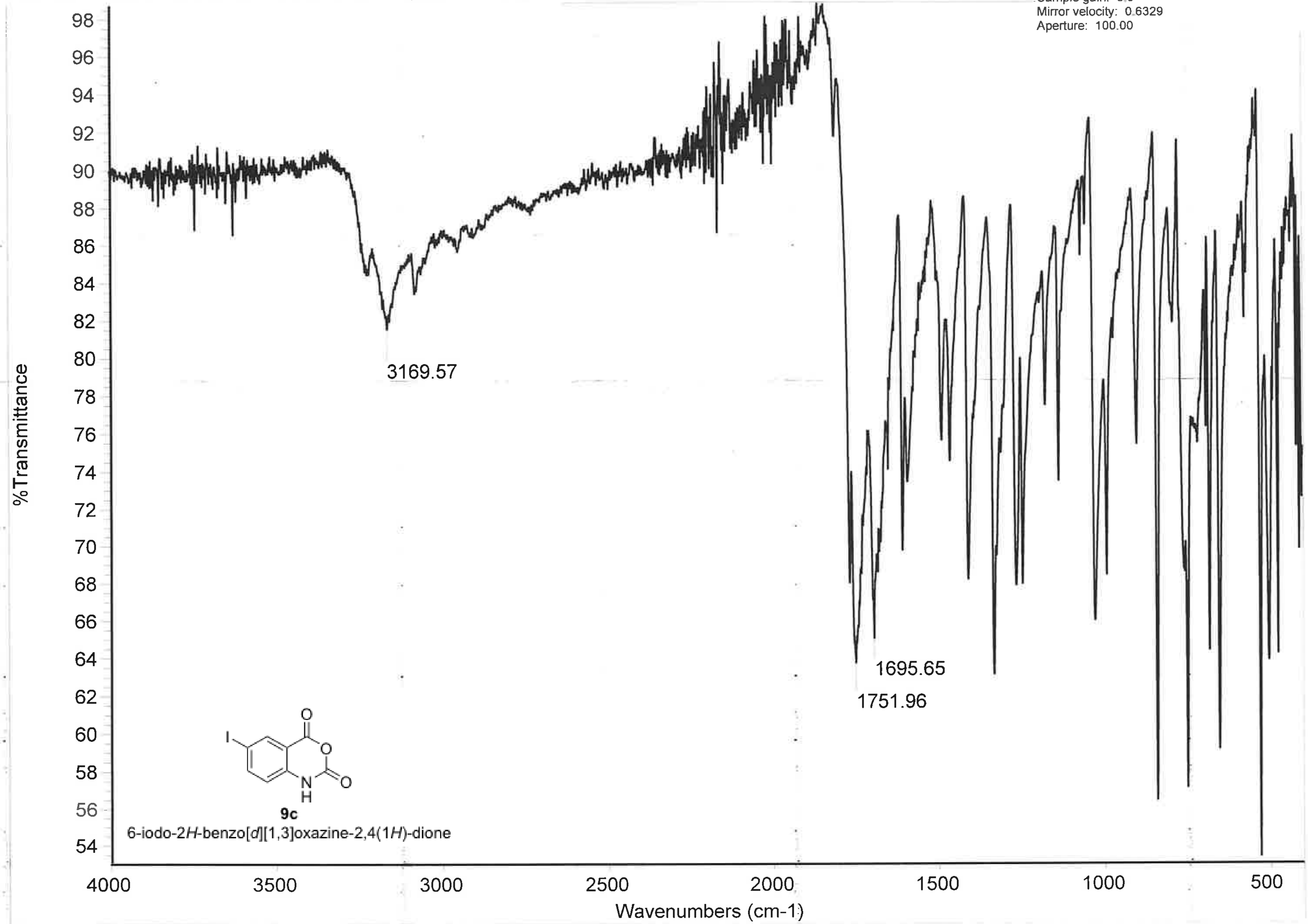

AHP-1-018 (DMSO, 400 MHz) Pure Purple Solid - Synthesis of 4-idod isatoic anhydride

NAME	AHP-1-018	
EXPNO	10	
PROCNO	1	
Date_	20170223	
Time	21.41	h
INSTRUM	spect	
PROBHD	Z108618_0161 (	
PULPROG	zg30	
TD	65536	
SOLVENT	DMSO	
NS	16	
DS	2	
SWH	8012.820	Hz
FIDRES	0.244532	Hz
AQ	4.0894966	sec
RG	203	
DW	62.400	usec
DE	6.50	usec
TE	297.9	K
D1	1.00000000	sec
TD0	1	
SFO1	400.1724710	MHz
NUC1	1H	
P1	9.88	usec
SI	65536	
SF	400.1700000	MHz
WDW	EM	
SSB	0	
LB	0.30	Hz
GB	0	
PC	1.00	

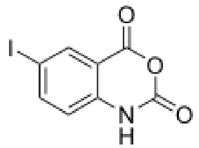

9c

6-iodo-2*H*-benzo[*d*][1,3]oxazine-2,4(1*H*)-dione

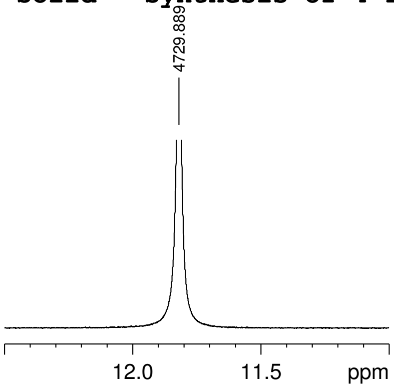

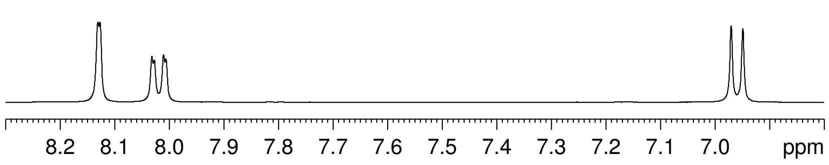

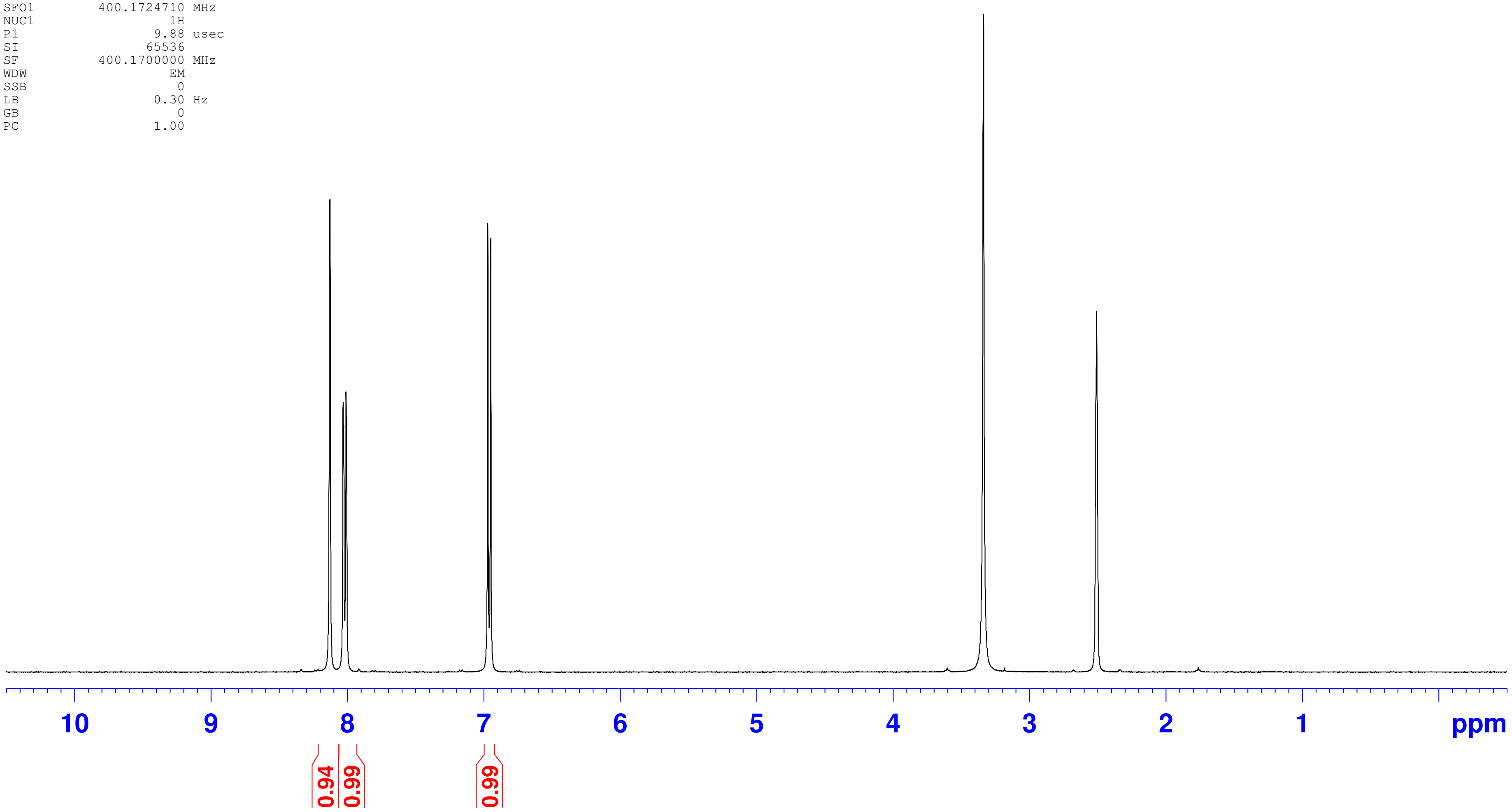

AHP-1-018 (DMSO, 400 MHz) Pure Purple Solid - Synthesis of 4-i

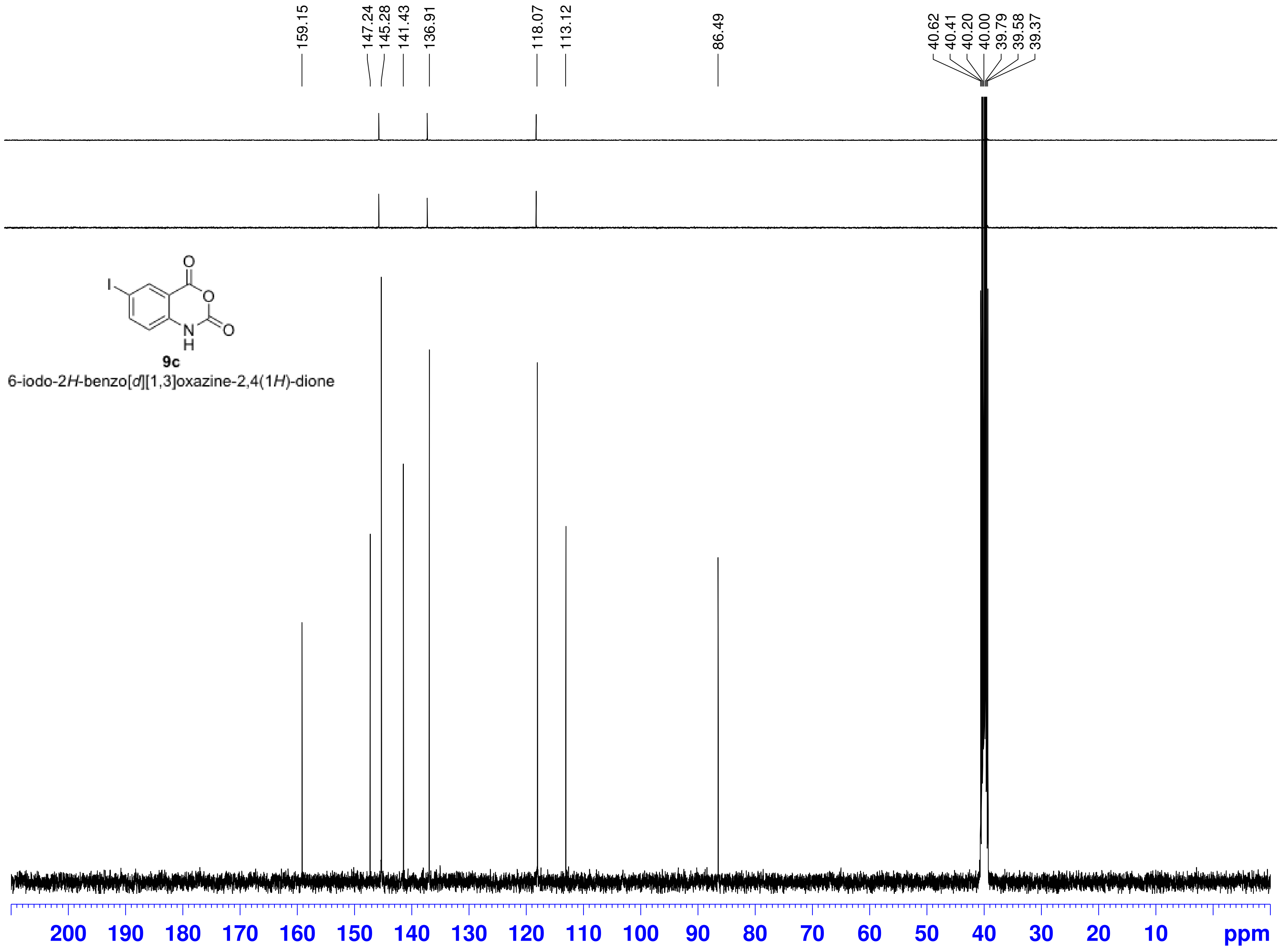

AHP-1-018 (DMSO, 400 MHz)

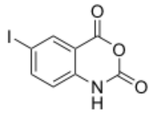

9c

6-iodo-2*H*-benzo[*d*][1,3]oxazine-2,4(1*H*)-dione

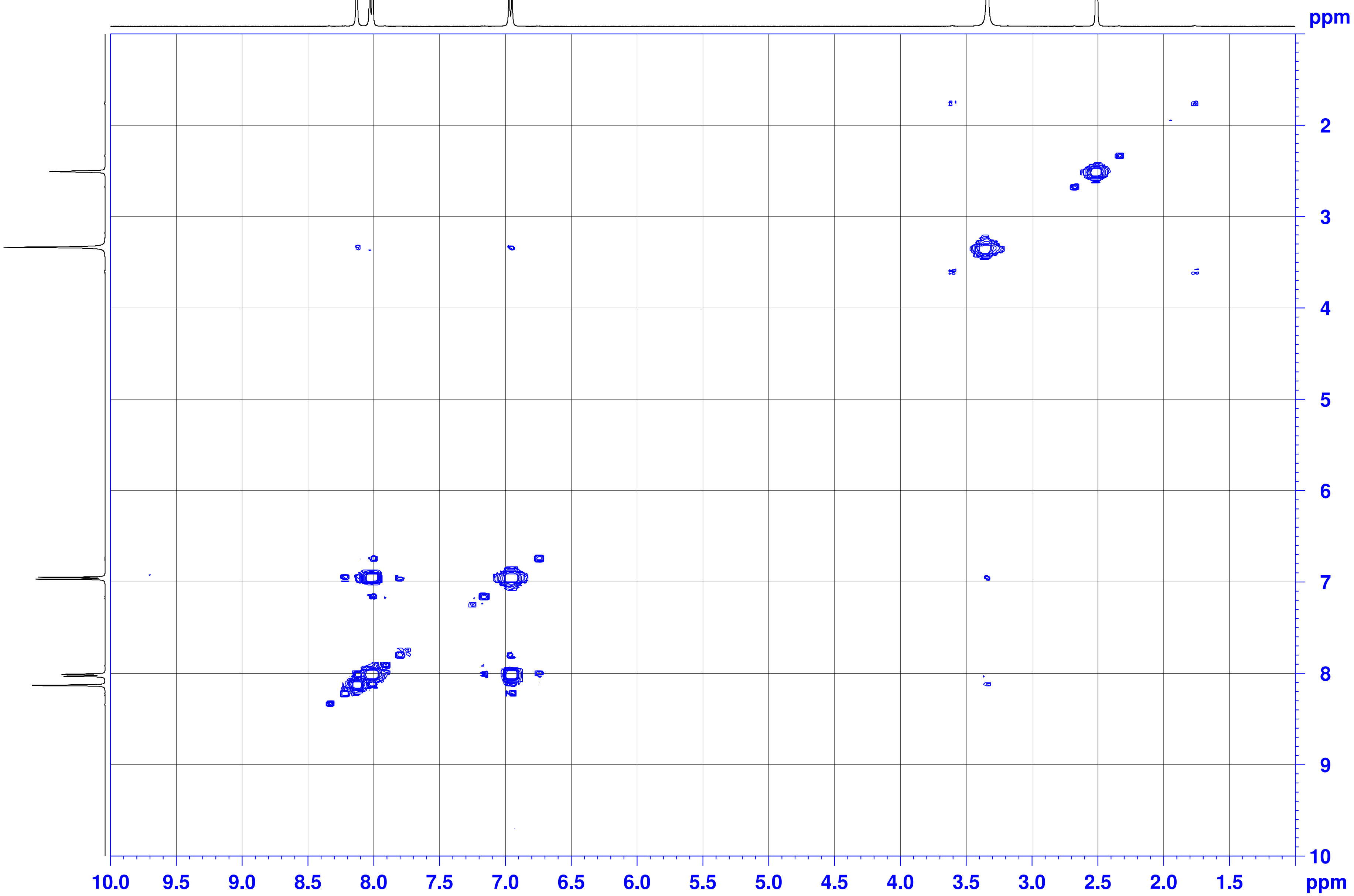

AHP-1-018 (DMSO, 400 MHz)

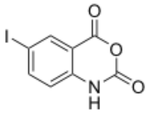

9c

6-iodo-2*H*-benzo[*d*][1,3]oxazine-2,4(1*H*)-dione

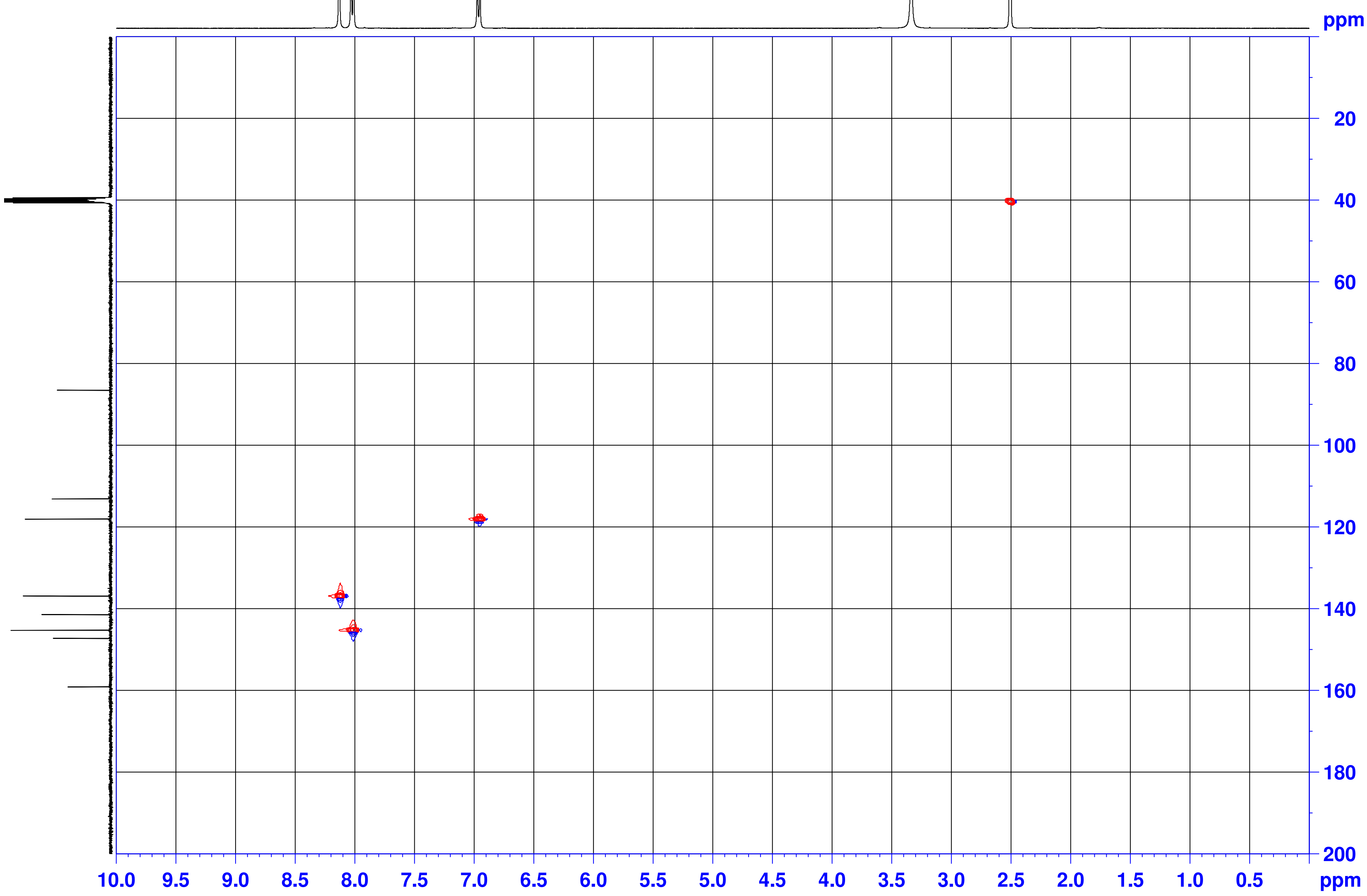

AHP-1-018 (DMSO, 400 MHz)

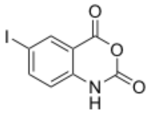

9c

6-iodo-2*H*-benzo[*d*][1,3]oxazine-2,4(1*H*)-dione

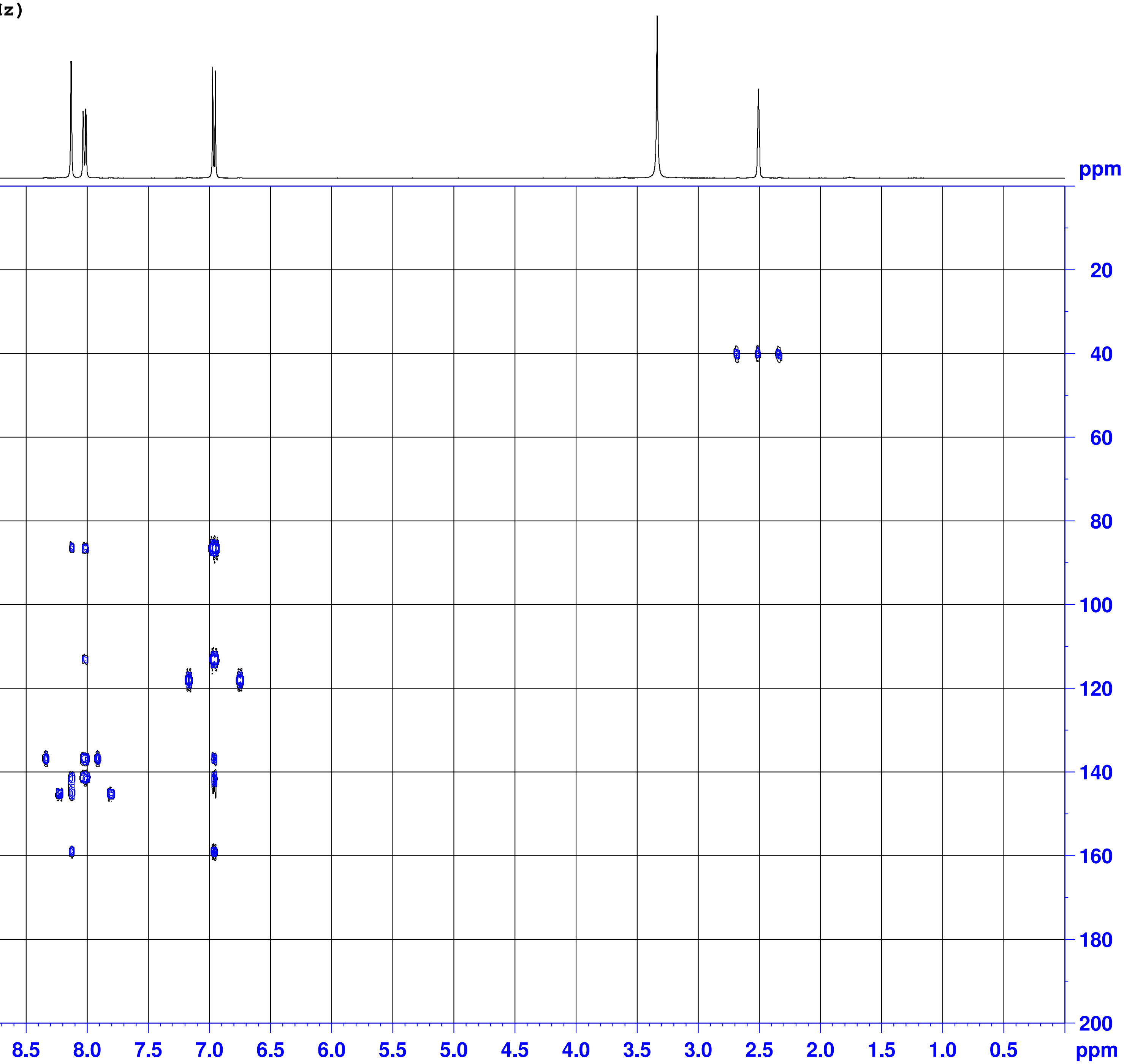

SMB-1-027

Collection time: Fri Jul 28 14:38:09 2017 (GMT-06:00)

Number of sample scans: 8
Number of background scans: 8
Resolution: 2.000
Sample gain: 8.0
Mirror velocity: 0.6329
Aperture: 100.00

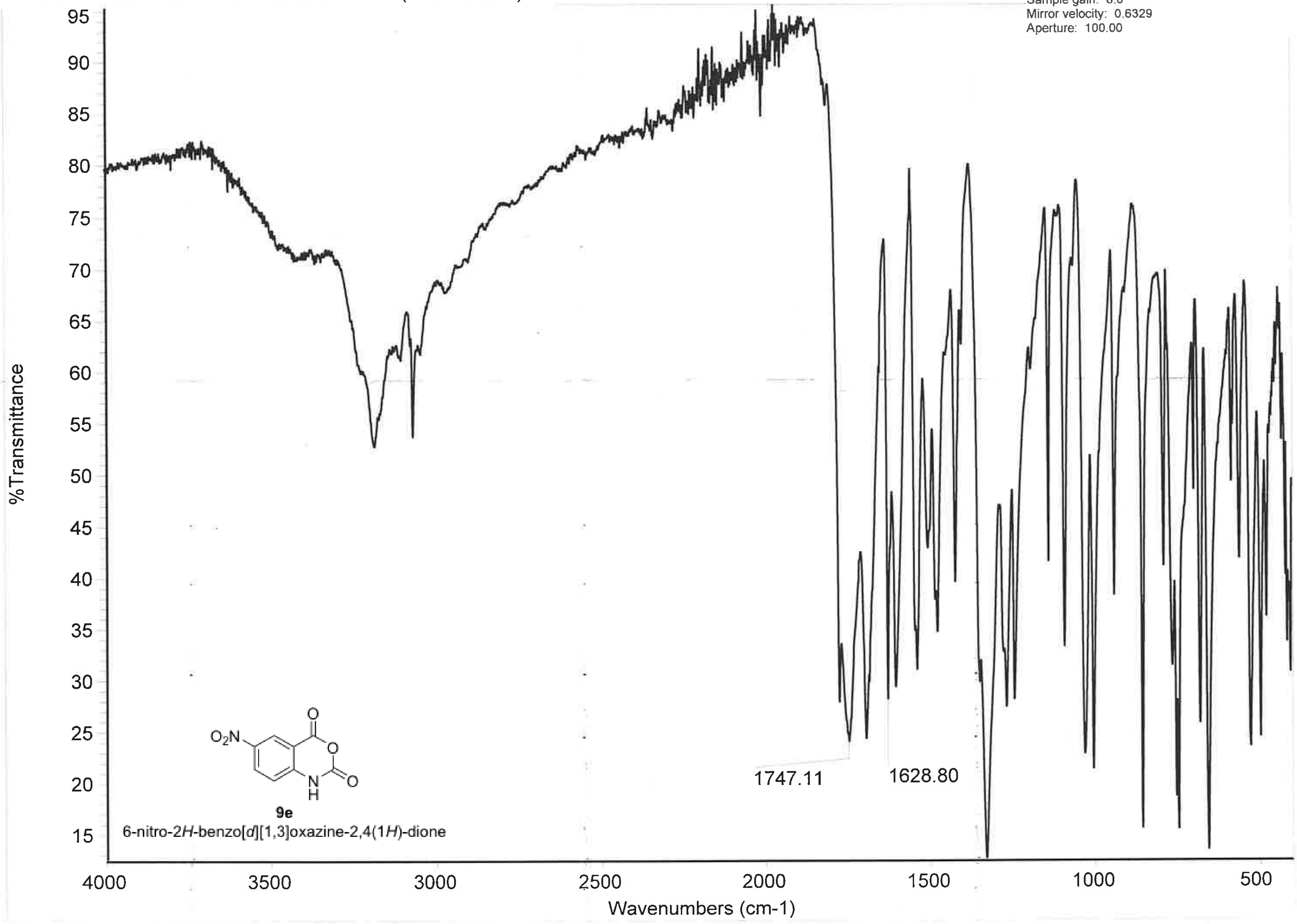

SMB-1-027 (DMSO, 400 MHz) Pure Tan Solid- 4-nitro isatoic anhydride synthesis

NAME	SMB-1-027
EXPNO	20
PROCNO	1
Date_	20161203
Time	15.13 h
INSTRUM	spect
PROBHD	Z108618_0161 (
PULPROG	zg30
TD	65536
SOLVENT	DMSO
NS	16
DS	2
SWH	8012.820 Hz
FIDRES	0.244532 Hz
AQ	4.0894966 sec
RG	203
DW	62.400 usec
DE	6.50 usec
TE	296.7 K
D1	1.00000000 sec
TD0	1
SFO1	400.1724710 MHz
NUC1	1H
P1	29.07 usec
SI	65536
SF	400.1699998 MHz
WDW	EM
SSB	0
LB	0.30 Hz
GB	0
PC	1.00

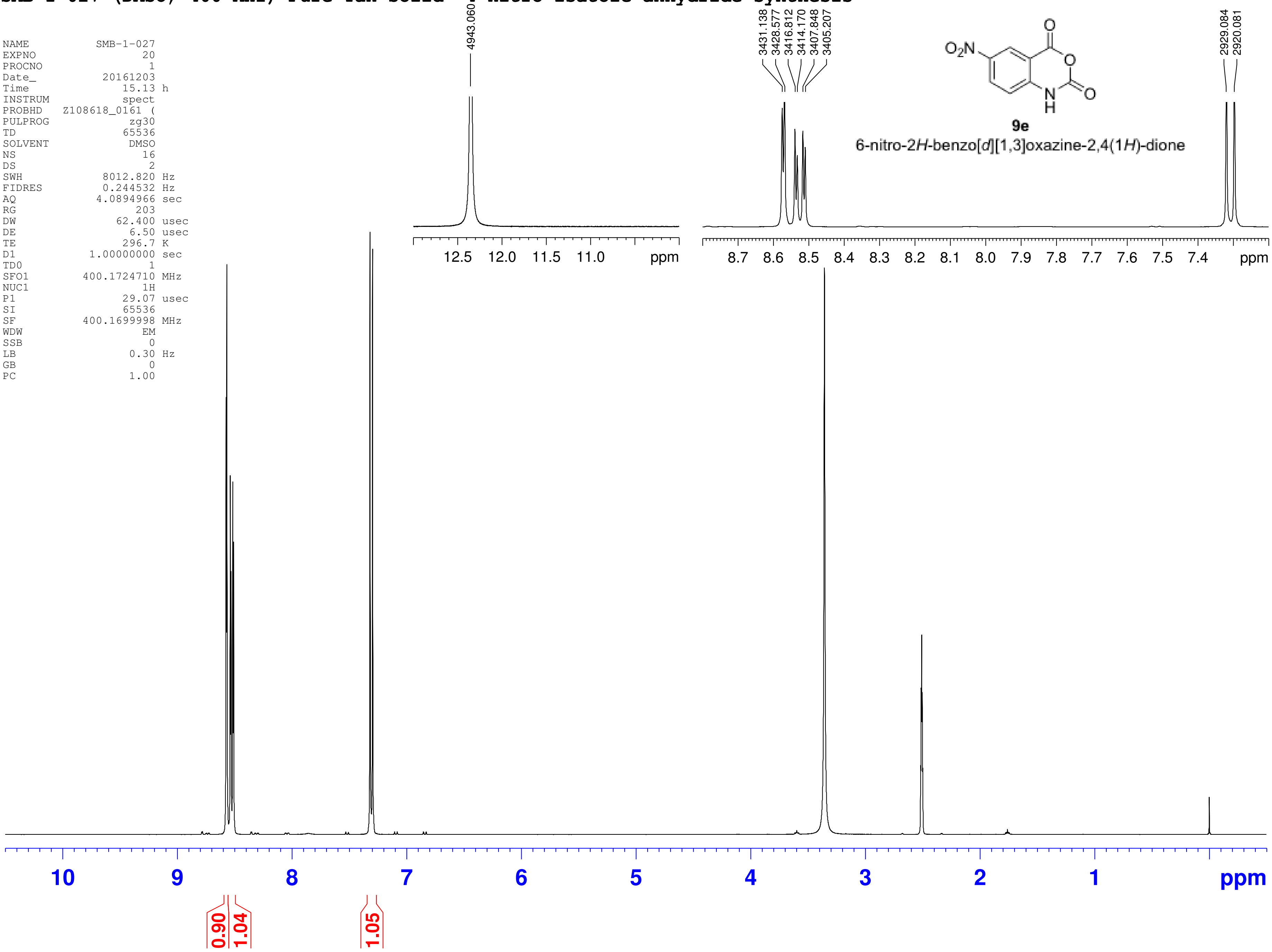

9e

6-nitro-2*H*-benzo[*d*][1,3]oxazine-2,4(1*H*)-dione

SMB-1-027 (DMSO, 400 MHz) Pure Tan Solid- 4-nitro isatoic anhy

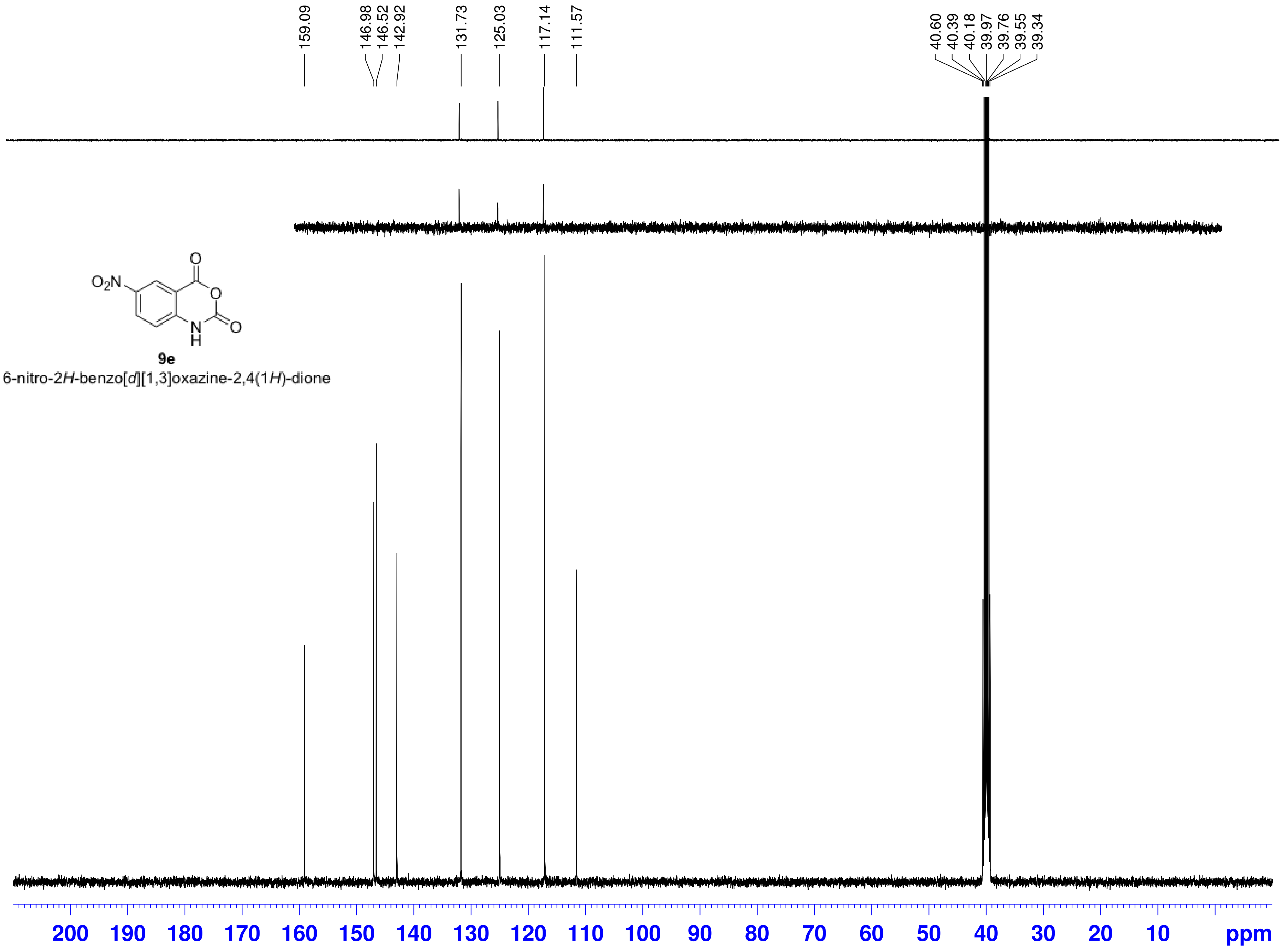

9e

6-nitro-2*H*-benzo[*d*][1,3]oxazine-2,4(1*H*)-dione

SMB-1-027 (DMSO, 400 MHz) Pure Tan Solid- 4-nitro isatoic anhydride synthesis

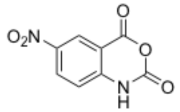

9e

6-nitro-2*H*-benzo[*d*][1,3]oxazine-2,4(1*H*)-dione

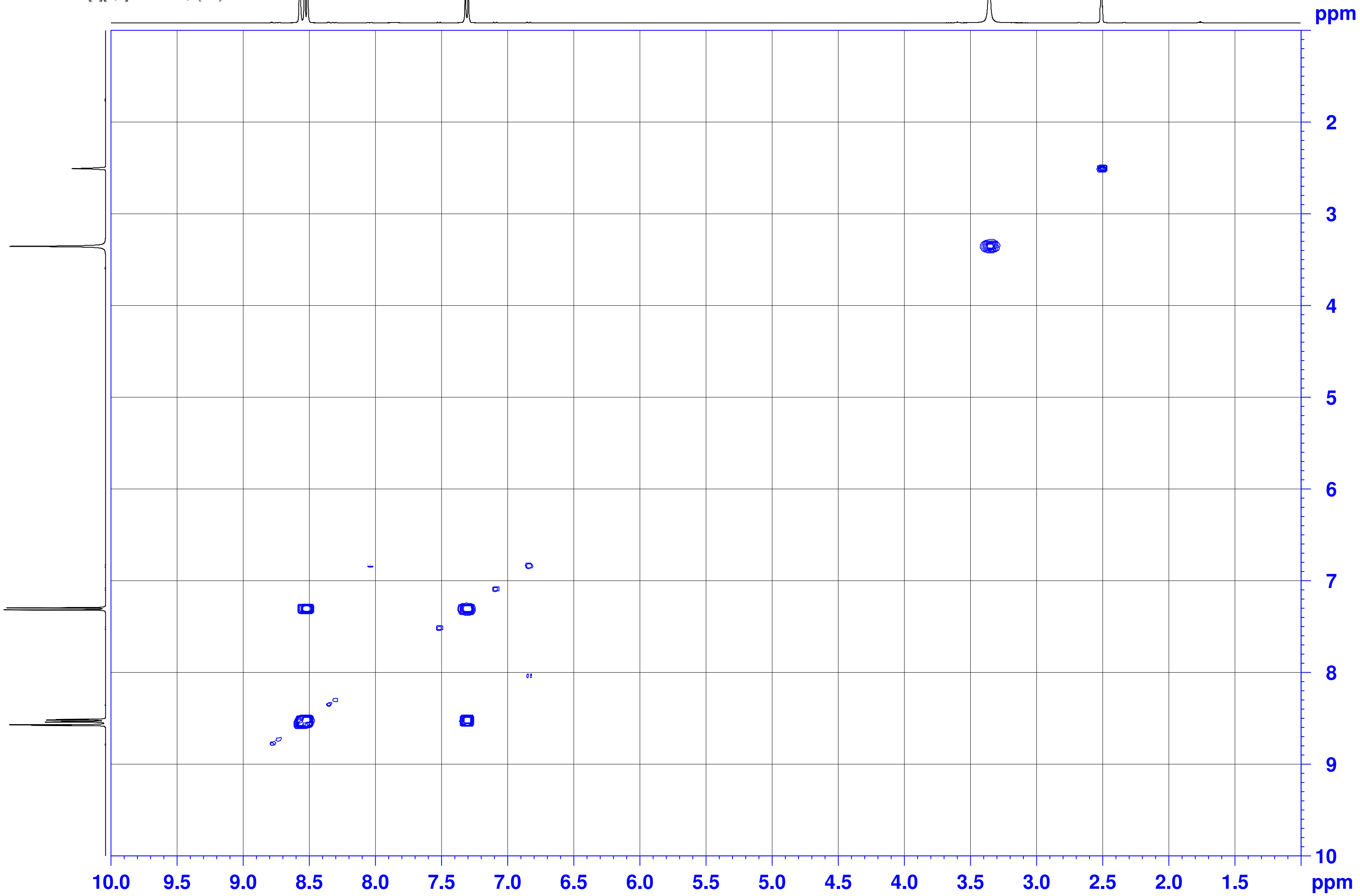

SMB-1-027 (DMSO, 400 MHz)

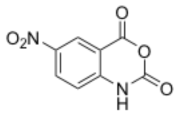

9e

6-nitro-2*H*-benzo[*d*][1,3]oxazine-2,4(1*H*)-dione

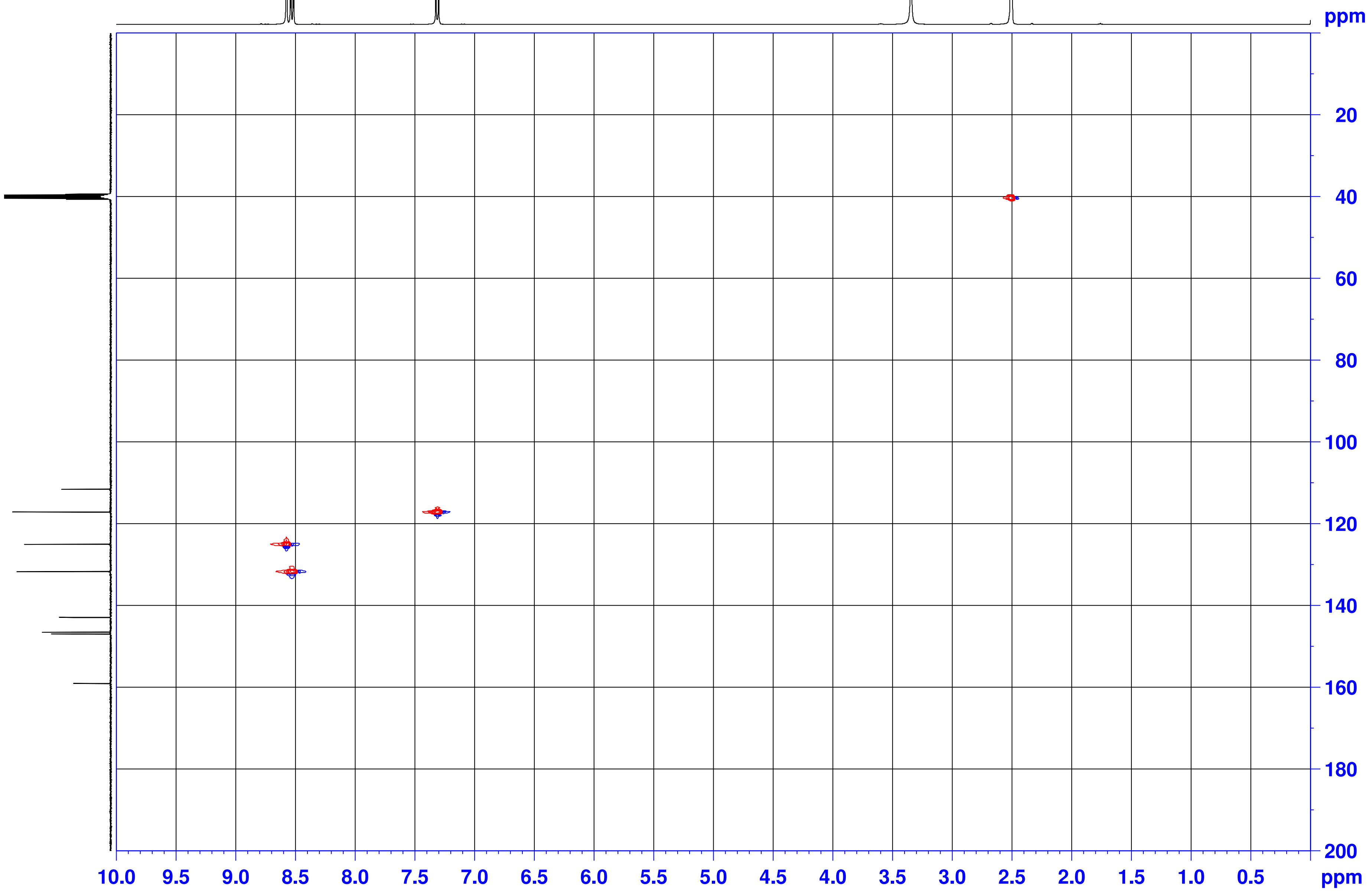

SMB-1-027 (DMSO, 400 MHz) Pure Tan Solid- 4-nitro isatoic anhydride synthesis

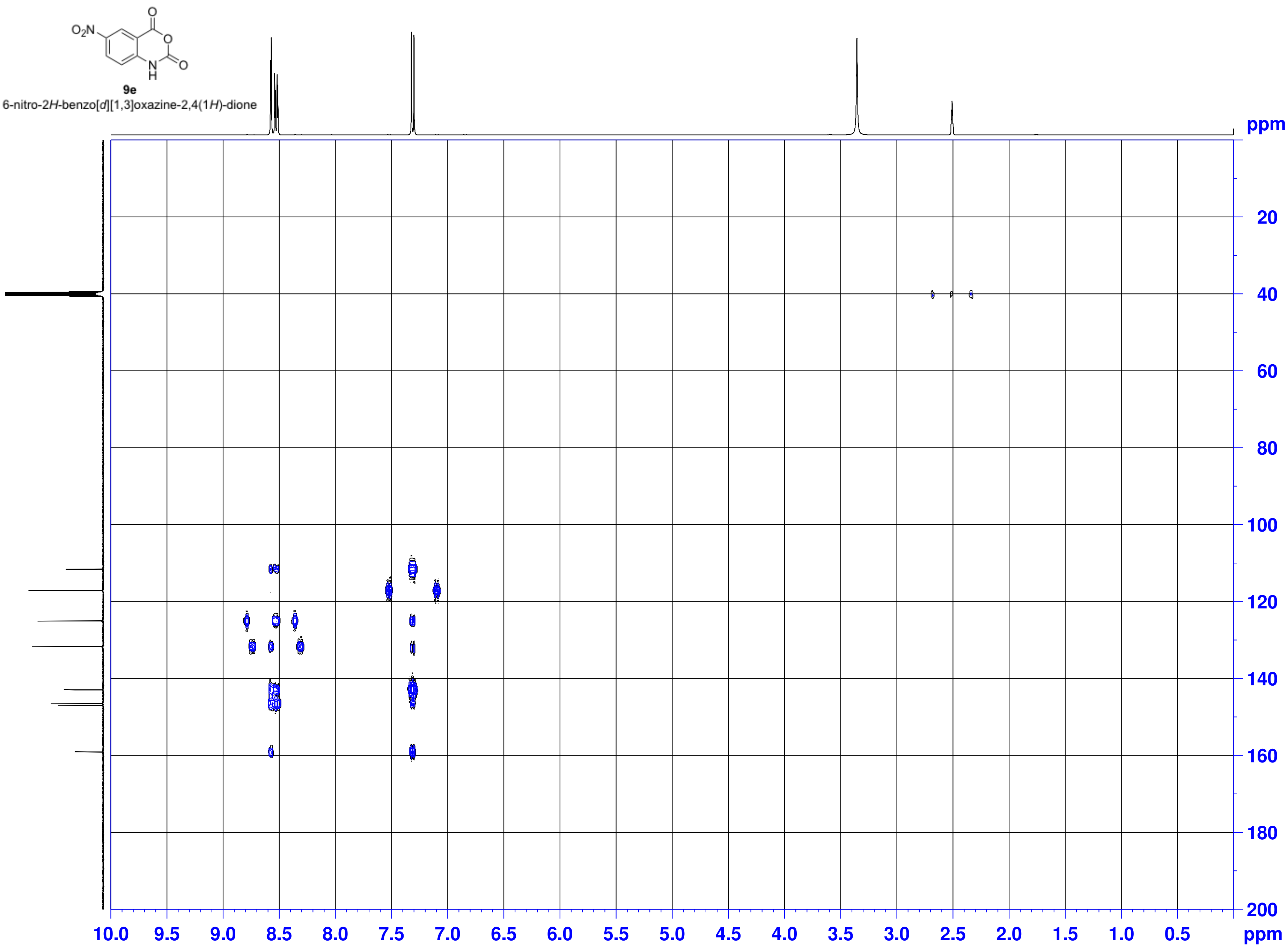

SMB-1-029
Collection time: Fri Jul 28 14:41:33 2017 (GMT-06:00)

Number of sample scans: 8
Number of background scans: 8
Resolution: 2.000
Sample gain: 8.0
Mirror velocity: 0.6329
Aperture: 100.00

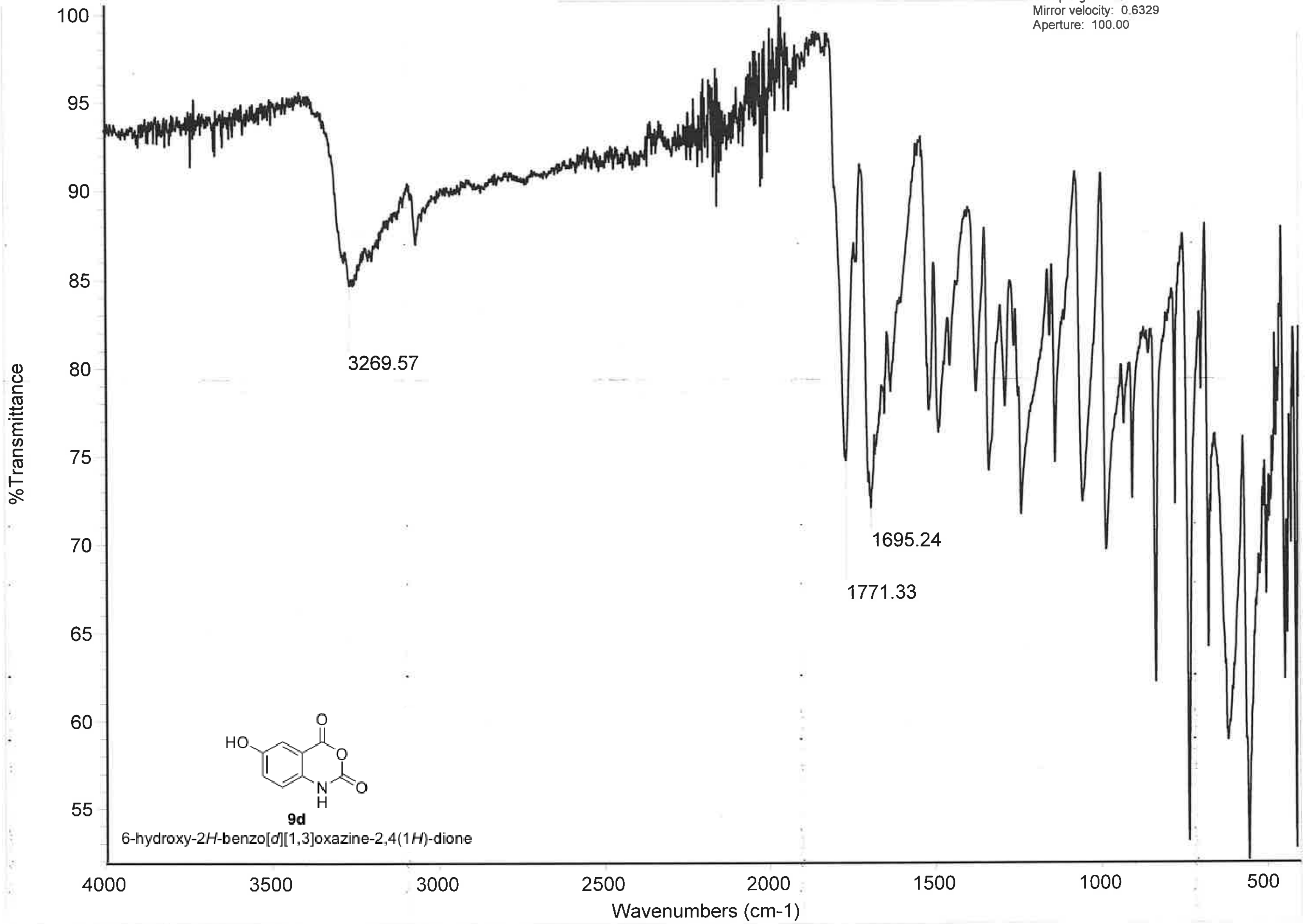

SMB-1-029 (DMSO, 400 MHz) Pure Brown Solid- hydroxy isatoic anhydride

NAME	SMB-1-029
EXPNO	20
PROCNO	1
Date_	20161130
Time	20.42 h
INSTRUM	spect
PROBHD	Z108618_0161 (
PULPROG	zg30
TD	65536
SOLVENT	DMSO
NS	16
DS	2
SWH	8012.820 Hz
FIDRES	0.244532 Hz
AQ	4.0894966 sec
RG	128
DW	62.400 usec
DE	6.50 usec
TE	297.2 K
D1	1.00000000 sec
TD0	1
SFO1	400.1724710 MHz
NUC1	1H
P1	29.07 usec
SI	65536
SF	400.1699913 MHz
WDW	EM
SSB	0
LB	0.30 Hz
GB	0
PC	1.00

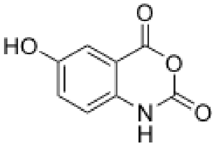

9d

6-hydroxy-2*H*-benzo[*d*][1,3]oxazine-2,4(1*H*)-dione

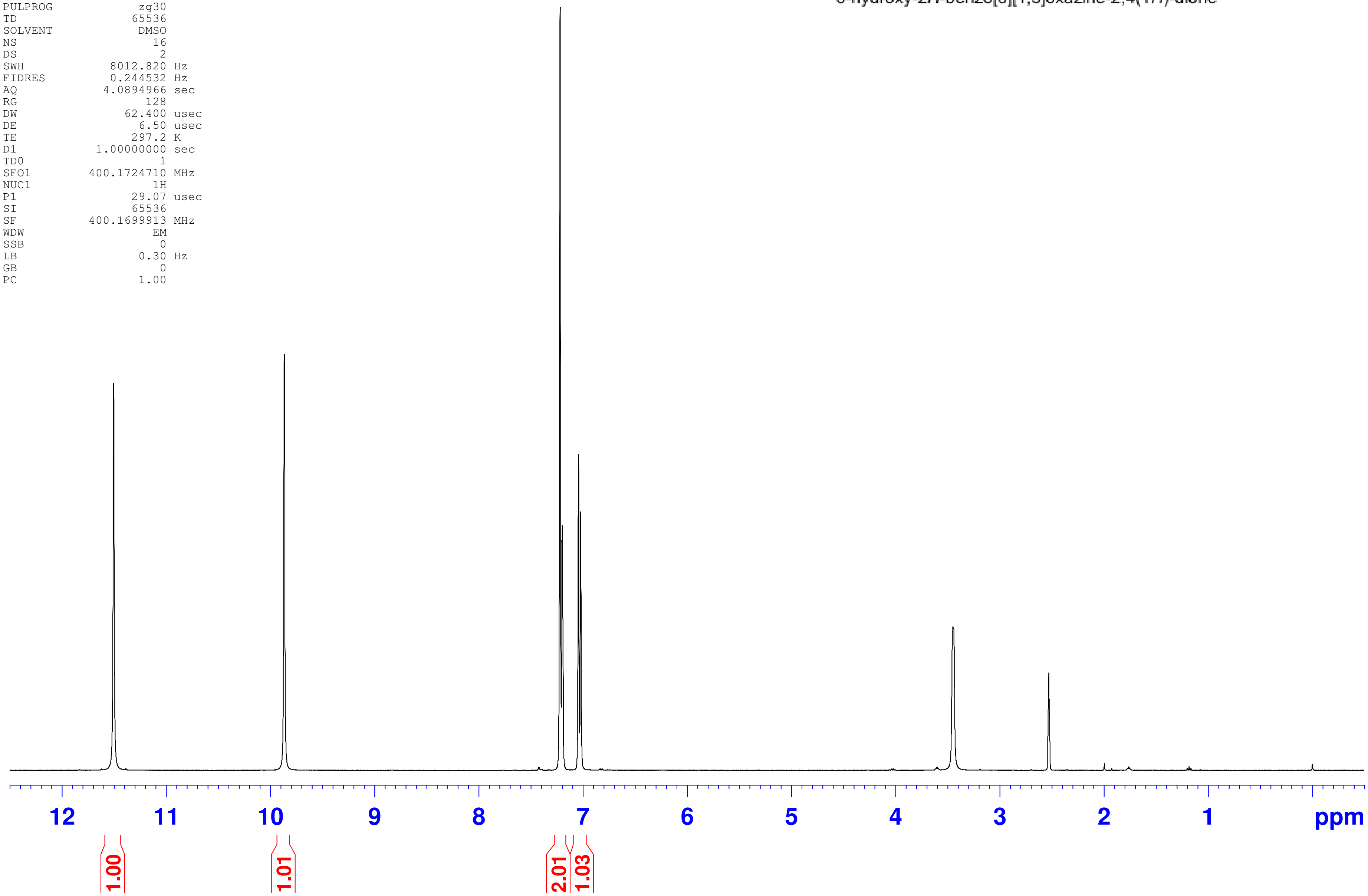

SMB-1-029 (DMSO, 400 MHz) Pure Brown Solid- hydroxy isatoic ar

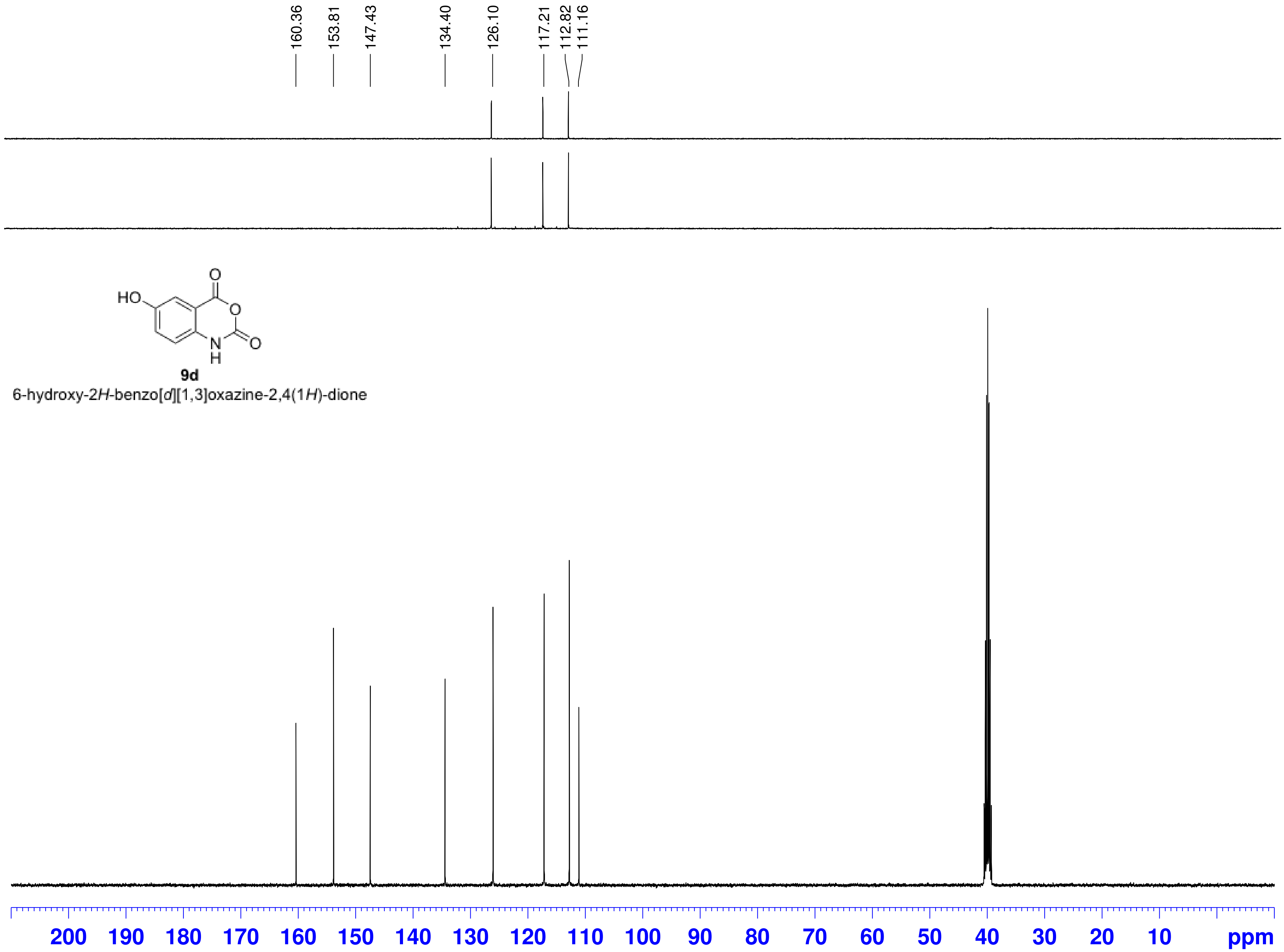

9d

6-hydroxy-2*H*-benzo[*d*][1,3]oxazine-2,4(1*H*)-dione

SMB-1-029 (DMSO, 400 MHz) Pure Brown Solid- hydroxy isatoic anhydride

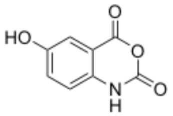

9d

6-hydroxy-2*H*-benzo[*d*][1,3]oxazine-2,4(1*H*)-dione

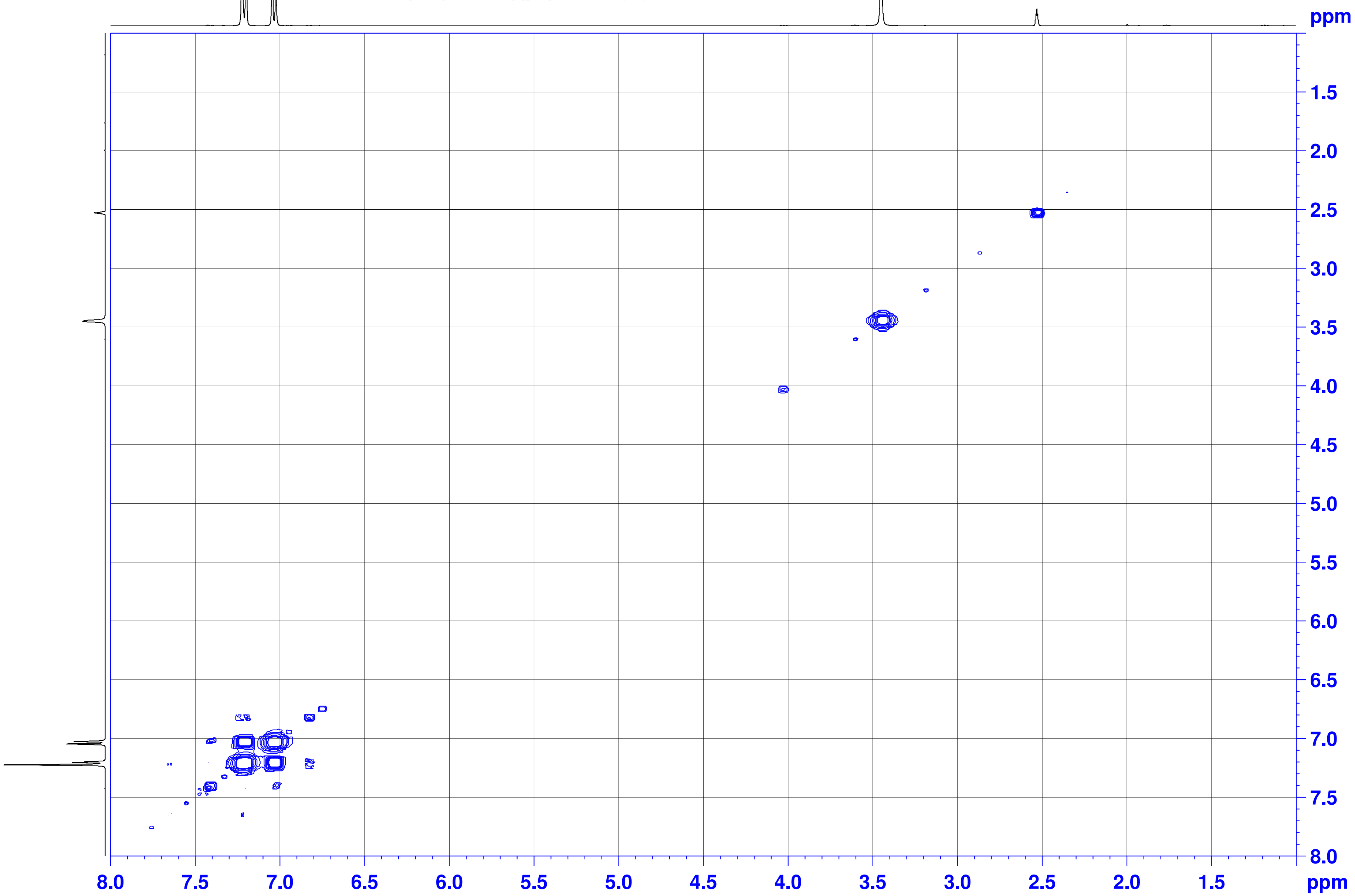

SMB-1-029 (DMSO, 400 MHz) Pure Brown Solid- hydroxy isatoic anhydride

9d

6-hydroxy-2*H*-benzo[*d*][1,3]oxazine-2,4(1*H*)-dione

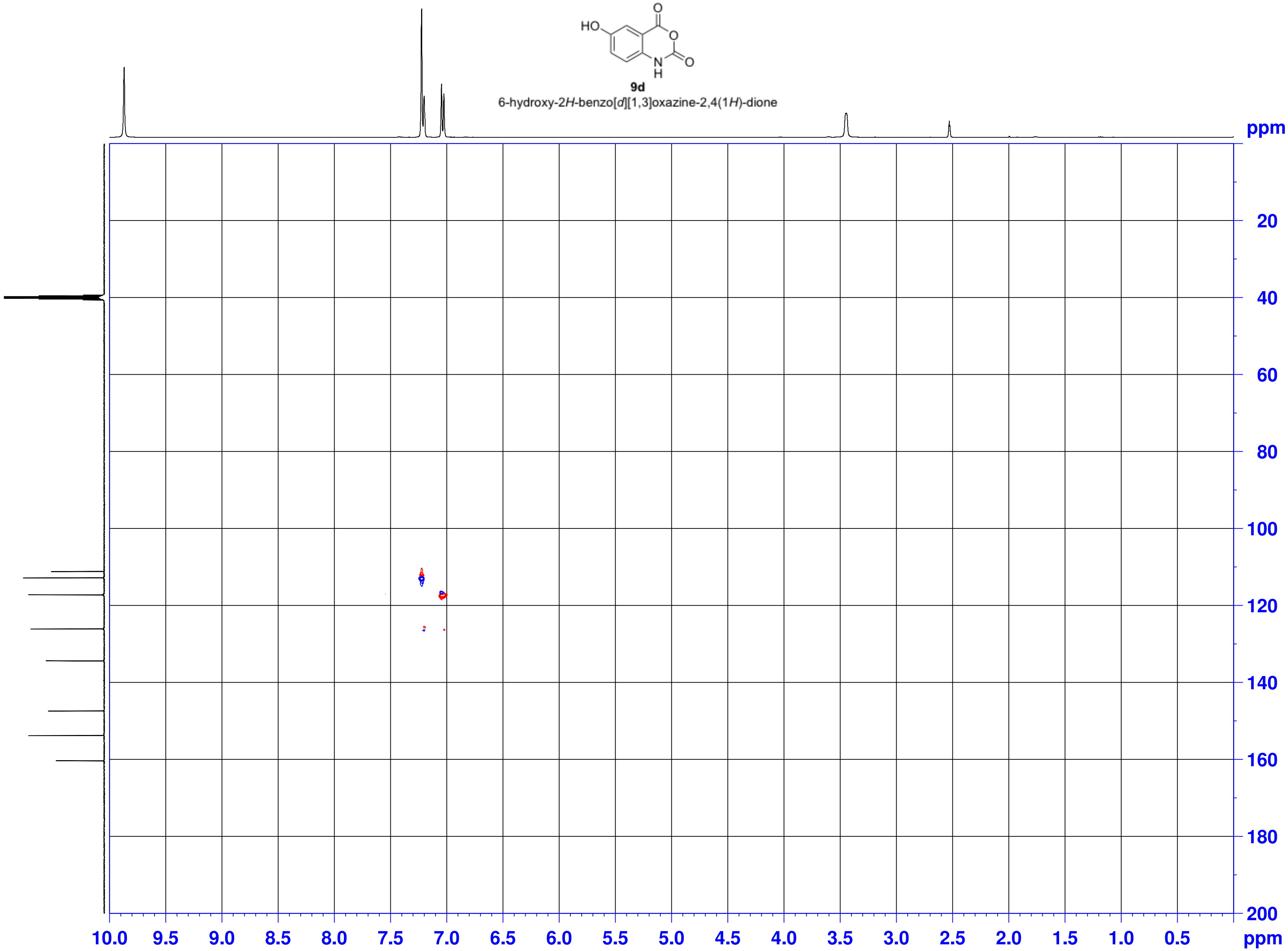

SMB-1-029 (DMSO, 400 MHz) Pure Brown Solid- hydroxy isatoic anhydride

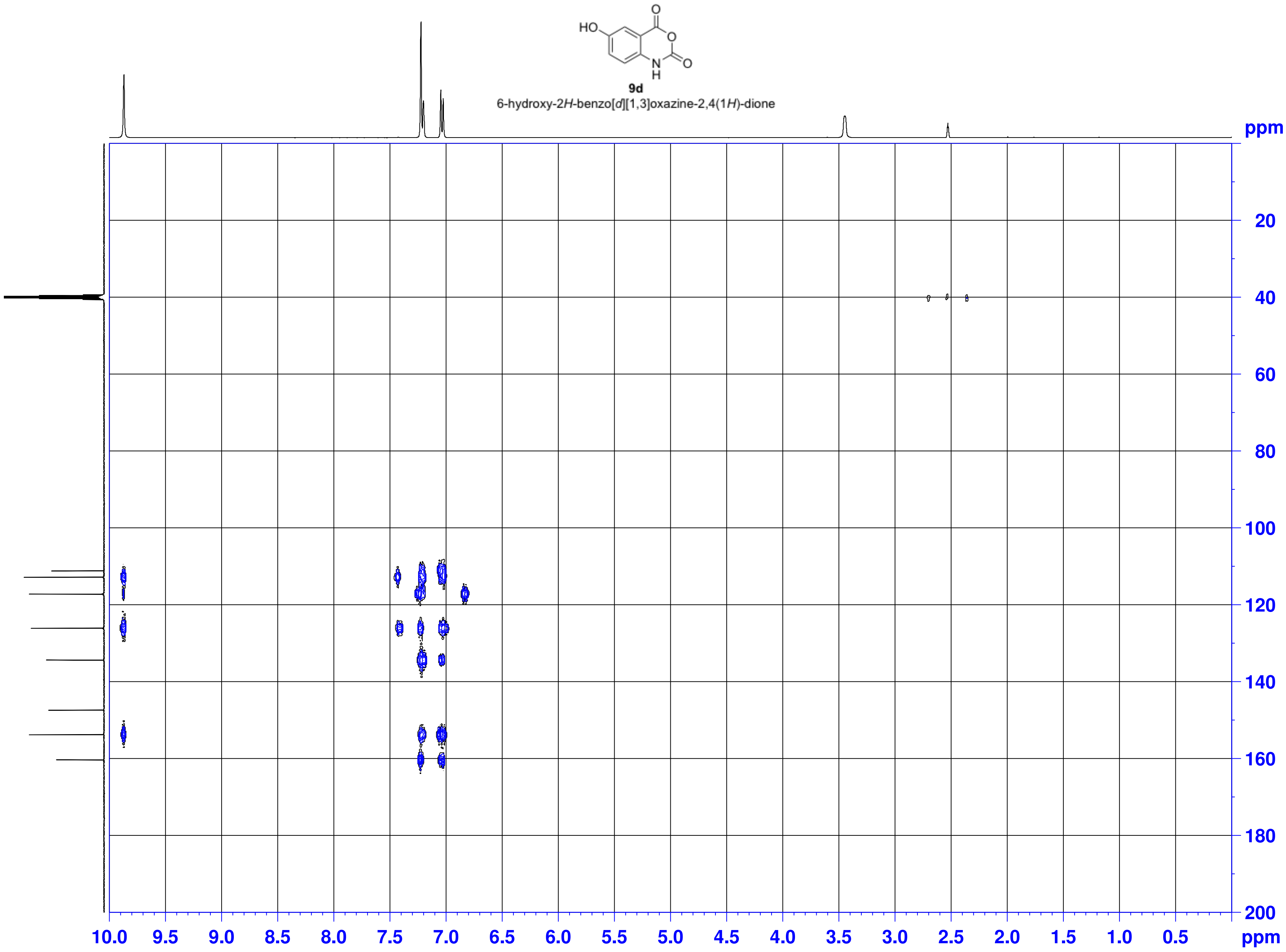

smb-1-028
Collection time: Fri Jul 28 14:44:38 2017 (GMT-06:00)

Number of sample scans: 8
Number of background scans: 8
Resolution: 2.000
Sample gain: 8.0
Mirror velocity: 0.6329
Aperture: 100.00

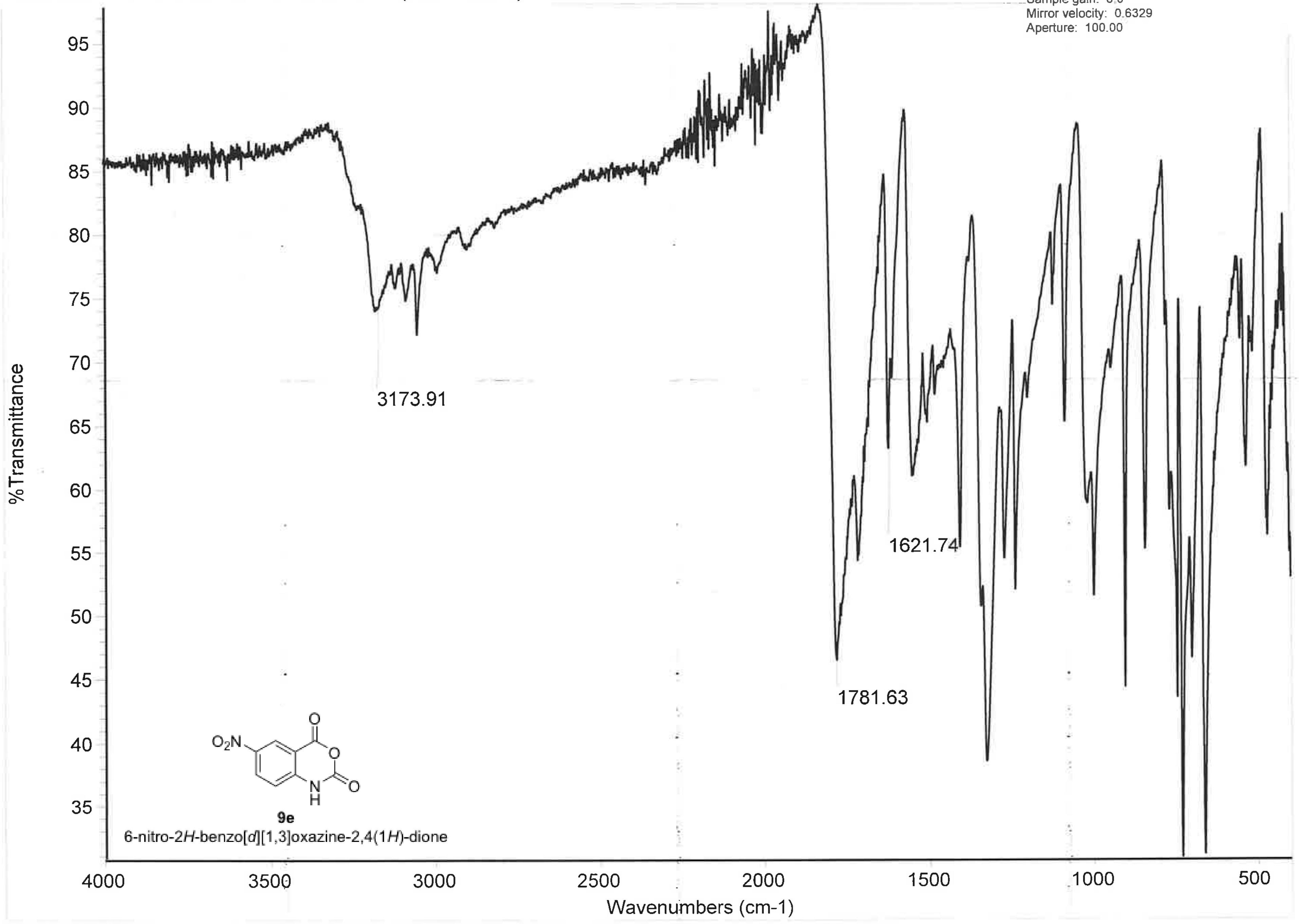

SMB-1-028 (DMSO, 400 MHz) Pure Yellow Solid - Synthesis of 4-nitro isatoic anhydride

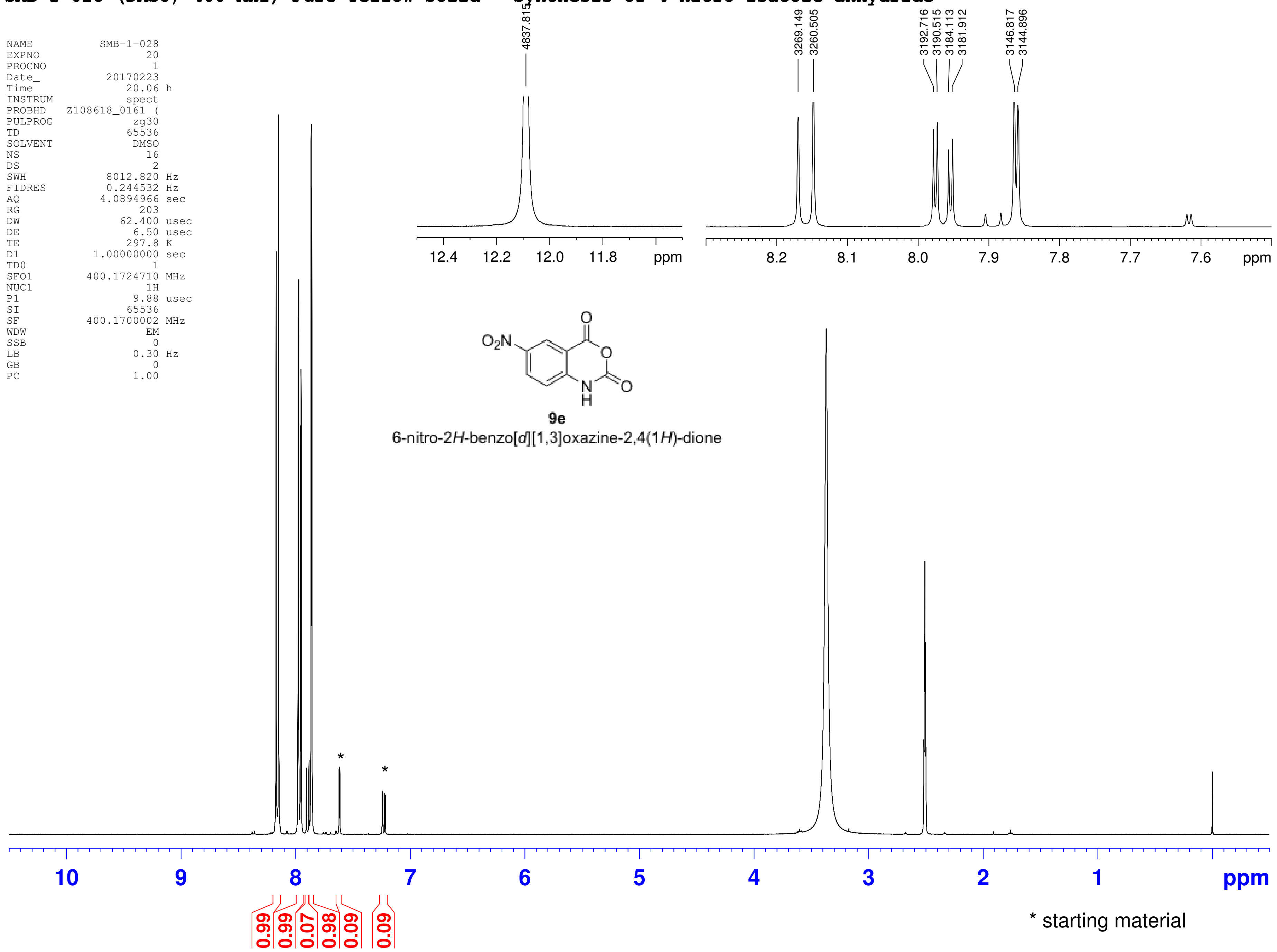

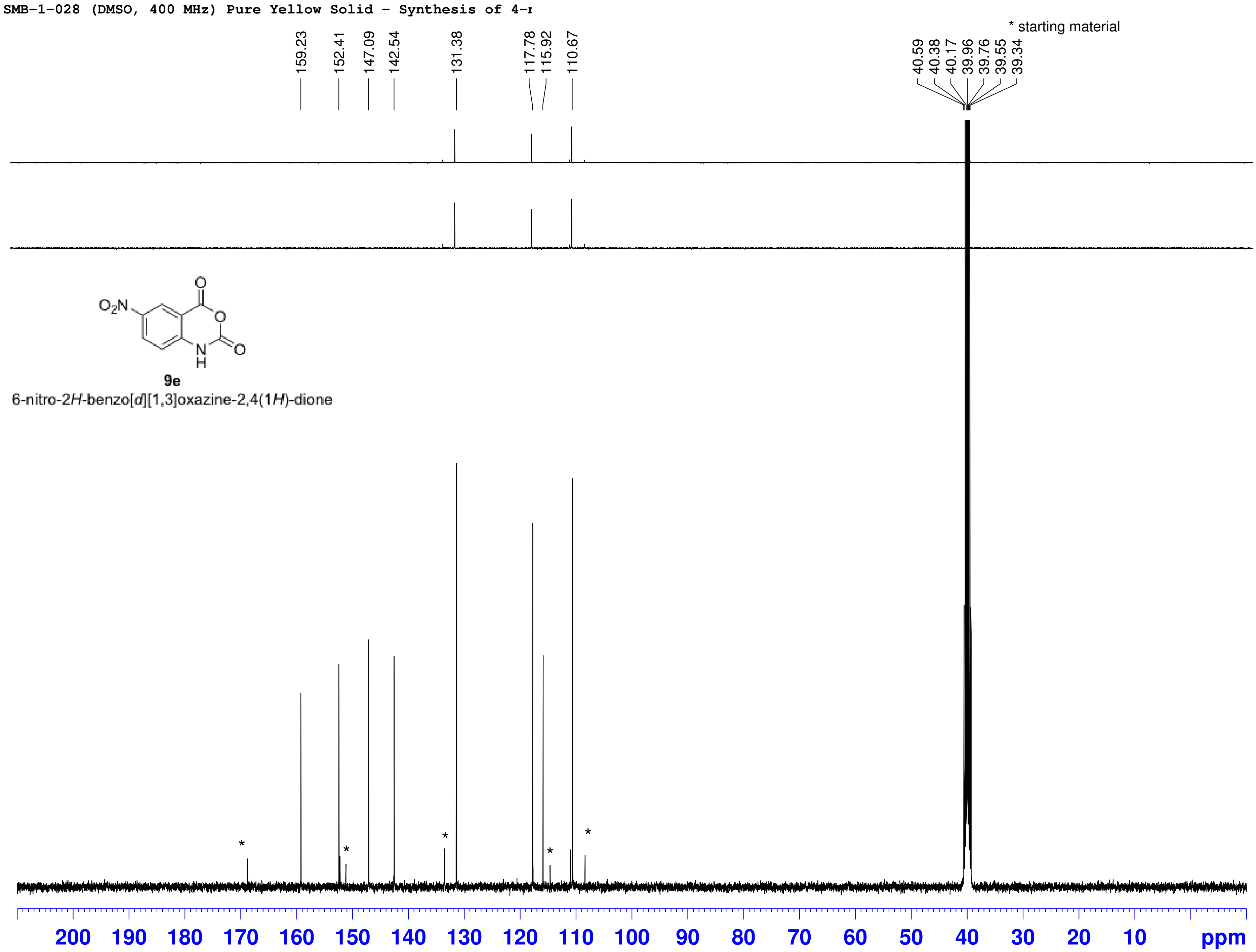
SMB-1-028 (DMSO, 400 MHz) Pure Yellow Solid - Synthesis of 4-r
* starting material
159.23
152.41
147.09
142.54
131.38
117.78
115.92
110.67
40.59
40.38
40.17
39.96
39.76
39.55
39.34
O2N
O
O
N
H
O
9e
6-nitro-2H-benzo[d][1,3]oxazine-2,4(1H)-dione
200 190 180 170 160 150 140 130 120 110 100 90 80 70 60 50 40 30 20 10 ppm

SMB-1-028 (DMSO, 400 MHz)

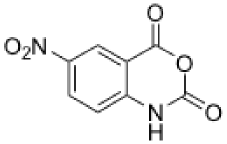

9e

6-nitro-2*H*-benzo[*d*][1,3]oxazine-2,4(1*H*)-dione

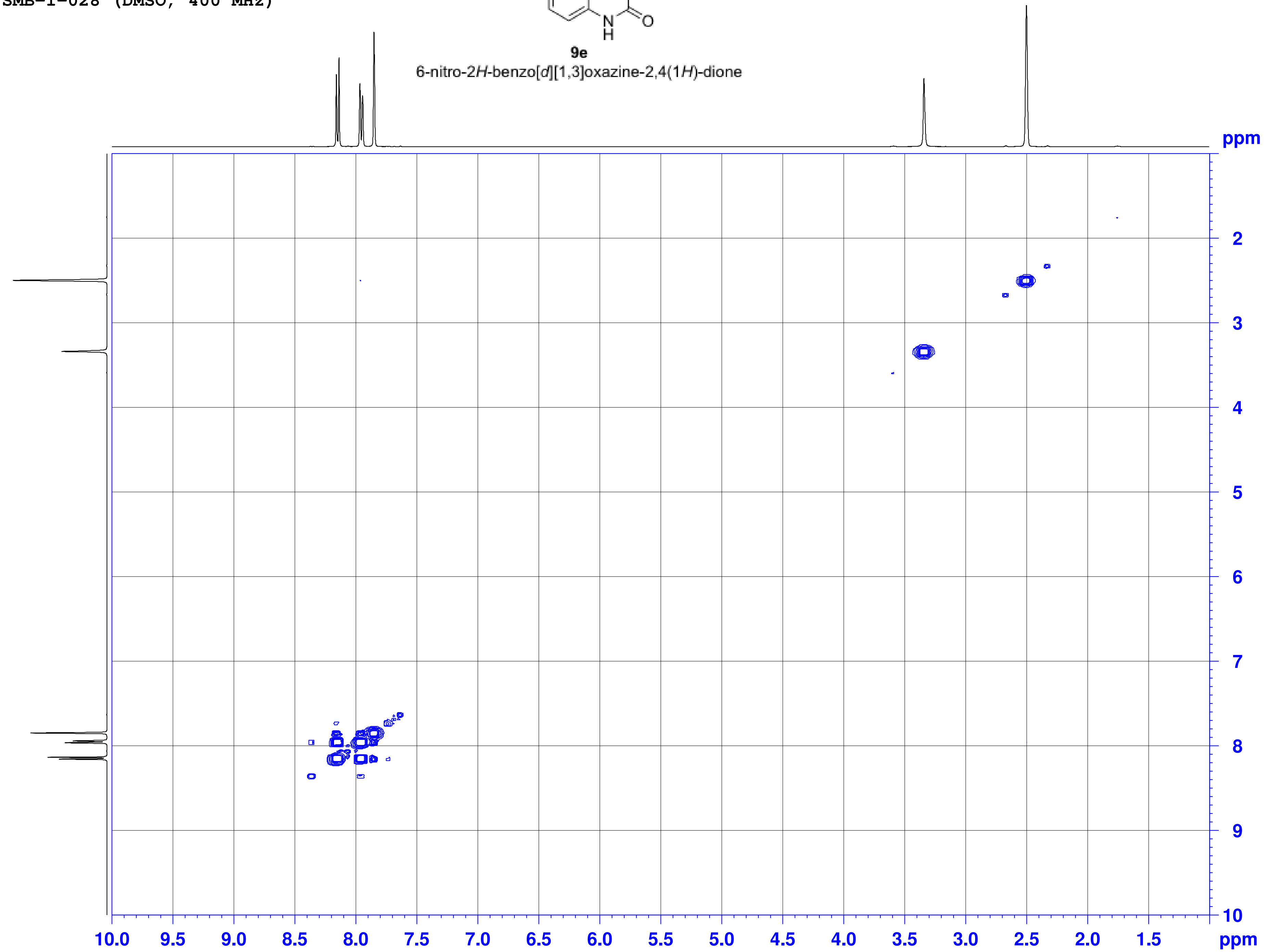

SMB-1-028 (DMSO, 400 MHz)

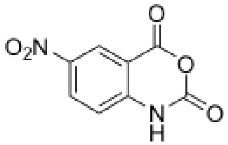

9e

6-nitro-2*H*-benzo[*d*][1,3]oxazine-2,4(1*H*)-dione

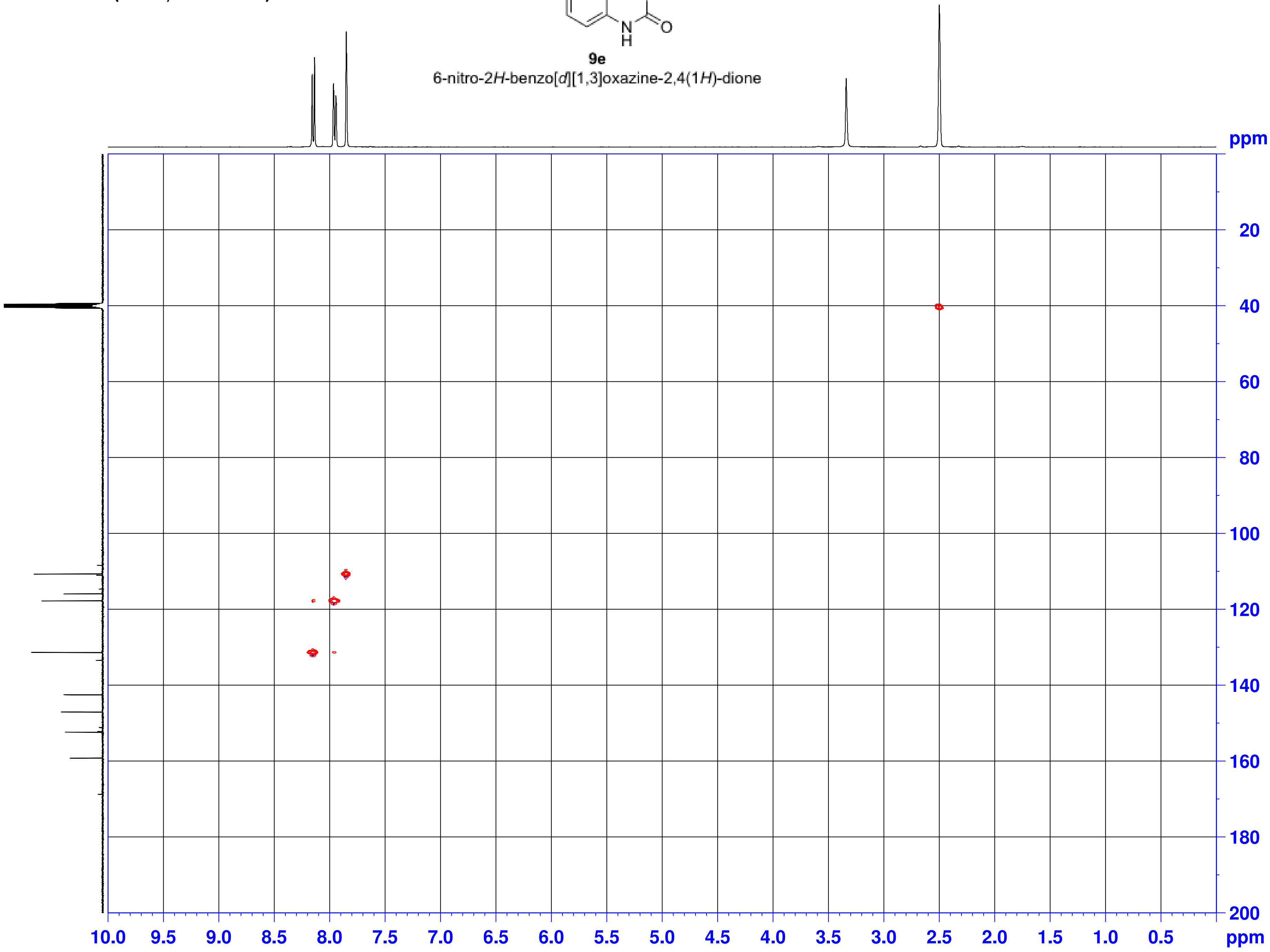

SMB-1-028 (DMSO, 400 MHz)

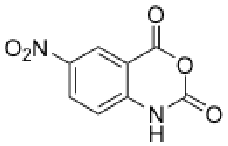

9e

6-nitro-2*H*-benzo[*d*][1,3]oxazine-2,4(1*H*)-dione

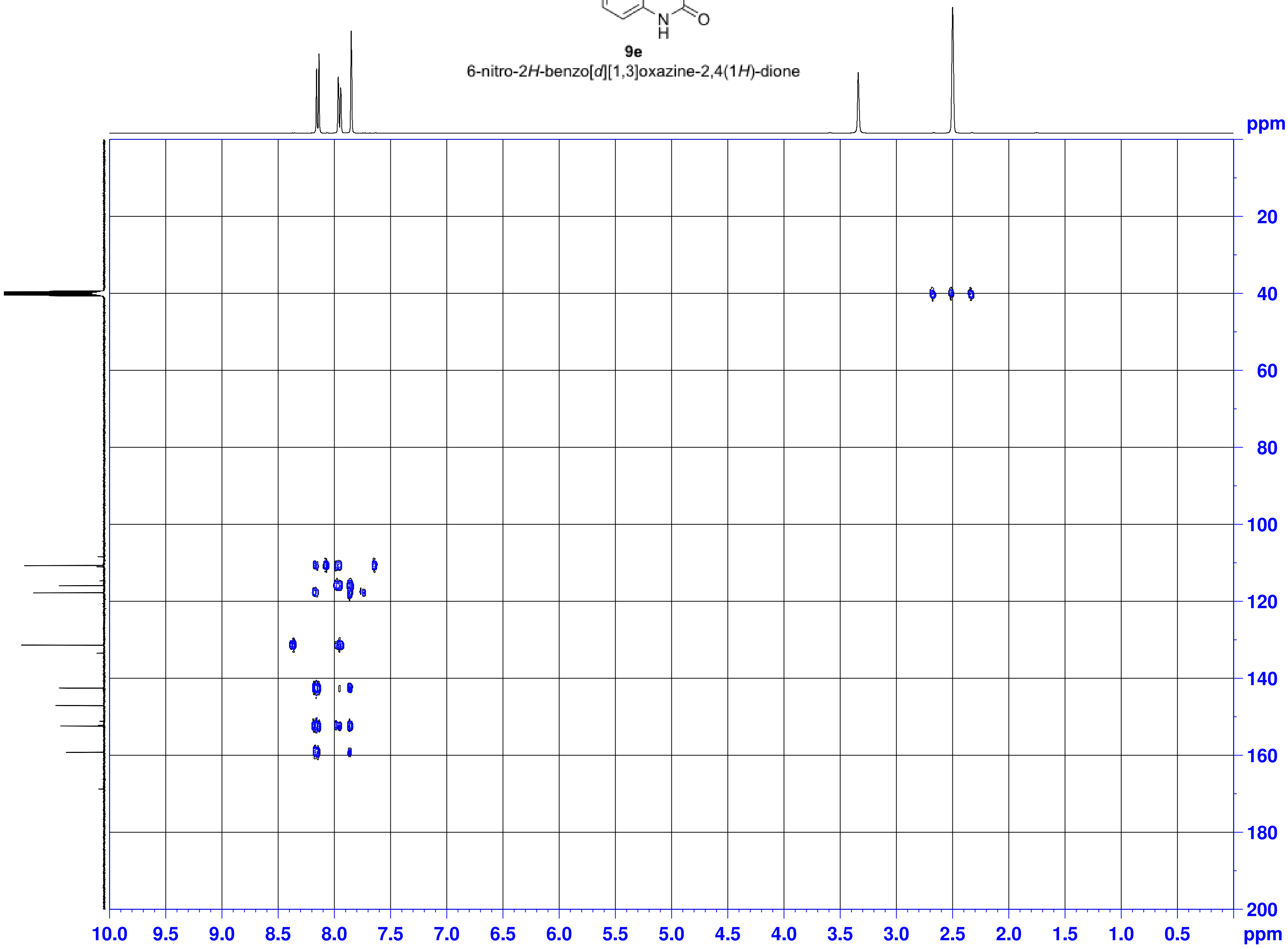

NGJ-8-029 (DMSO, 400 MHz) Crude White Solid - 7-bromoisatoic anhydride

NAME	NGJ-8-029	
EXPNO	20	
PROCNO	1	
Date_	20170816	
Time	18.00	h
INSTRUM	spect	
PROBHD	Z108618_0161 (	
PULPROG	zg30	
TD	65536	
SOLVENT	DMSO	
NS	16	
DS	2	
SWH	8012.820	Hz
FIDRES	0.244532	Hz
AQ	4.0894966	sec
RG	203	
DW	62.400	usec
DE	6.50	usec
TE	298.2	K
D1	1.00000000	sec
TD0	1	
SFO1	400.1724710	MHz
NUC1	1H	
P1	9.88	usec
SI	65536	
SF	400.1699994	MHz
WDW	EM	
SSB	0	
LB	0.30	Hz
GB	0	
PC	1.00	

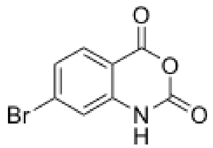

9f

7-bromo-2*H*-benzo[*d*][1,3]oxazine-2,4(1*H*)-dione

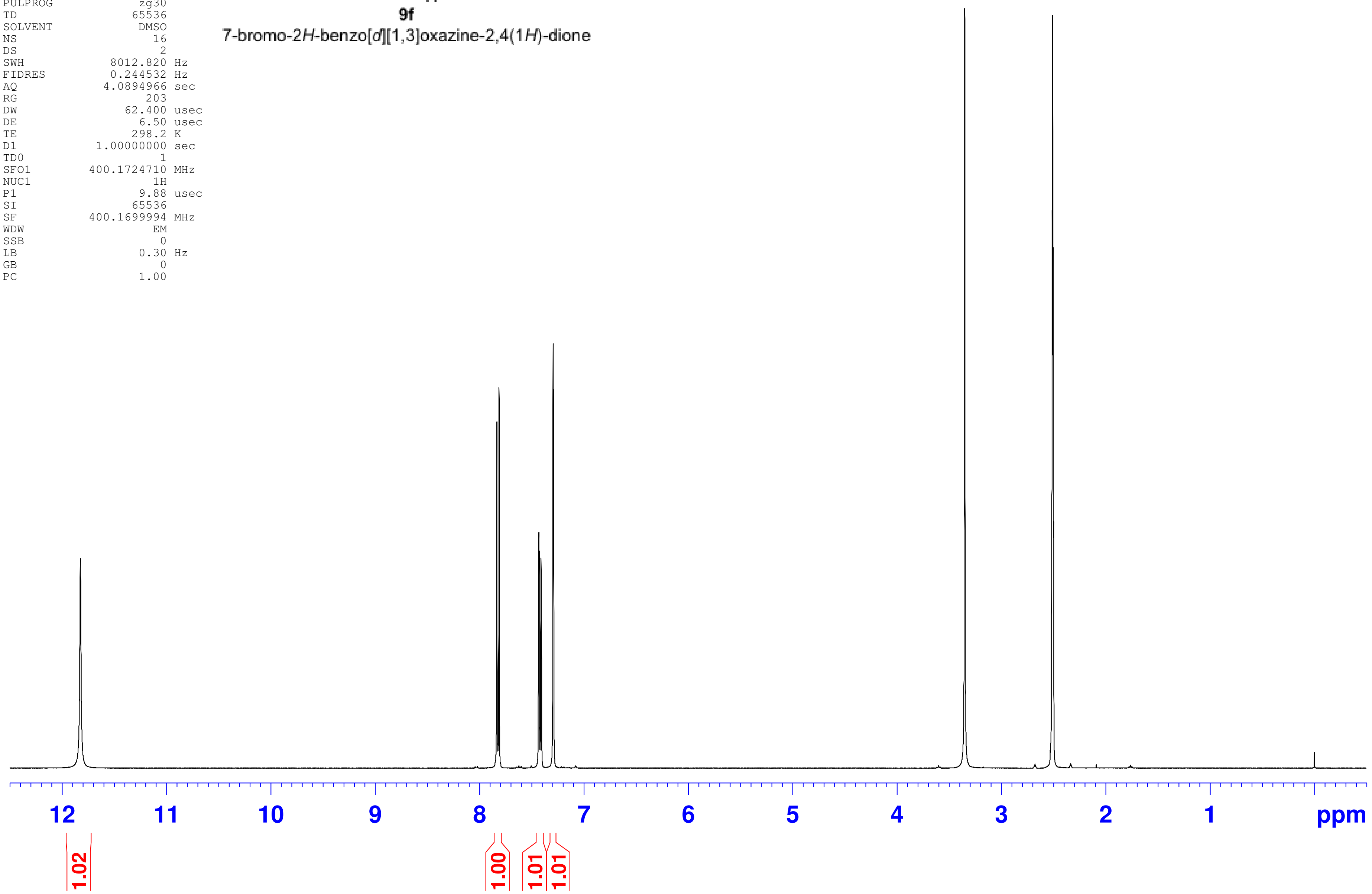

NGJ-8-029 (DMSO, 400 MHz) Crude White Solid - 7-bromoisatoic a

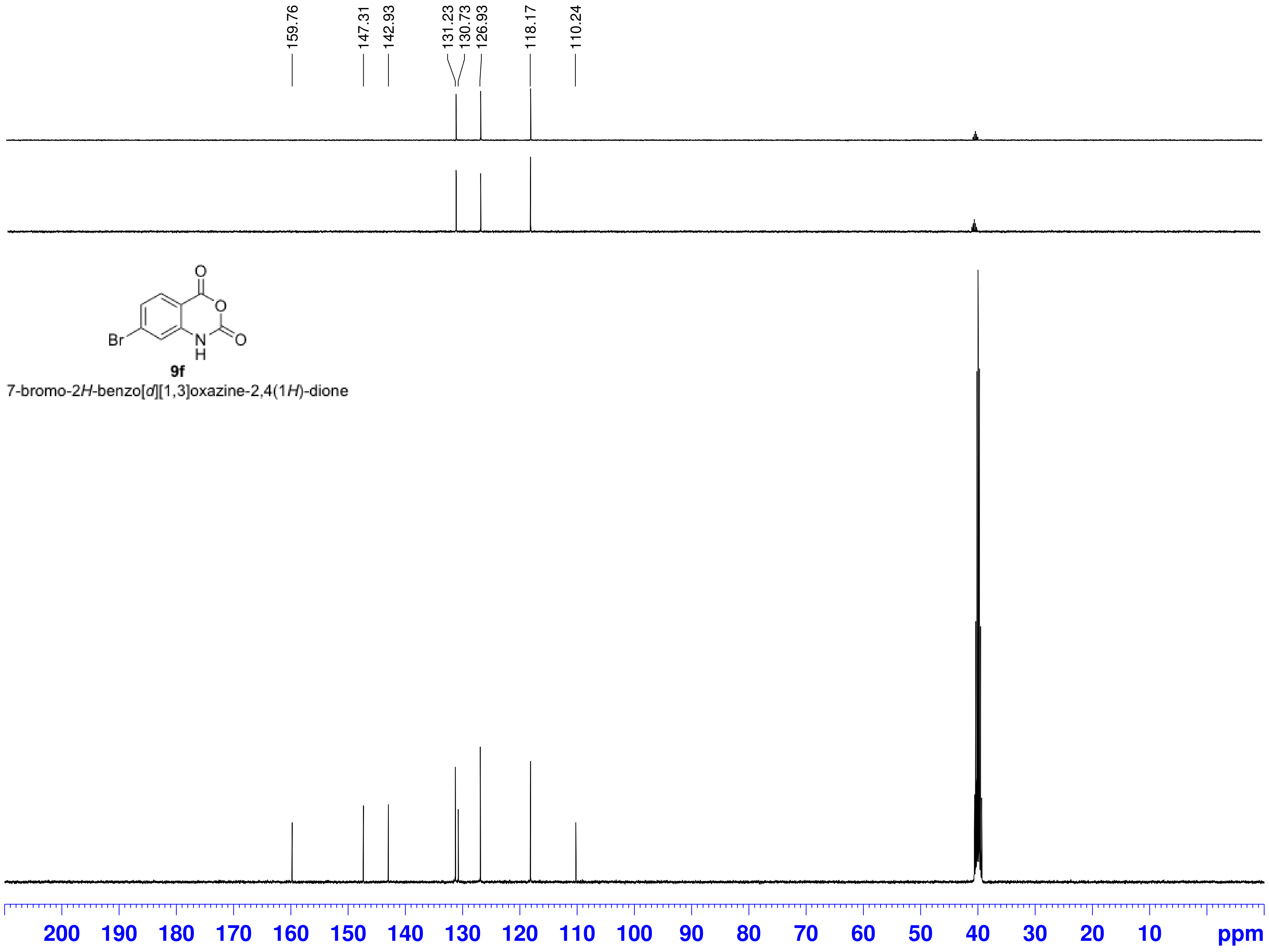

NGJ-8-029 (DMSO, 400 MHz) Crude White Solid - 7-bromoisatoic anhydride

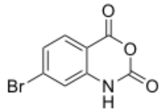

9f

7-bromo-2*H*-benzo[*d*][1,3]oxazine-2,4(1*H*)-dione

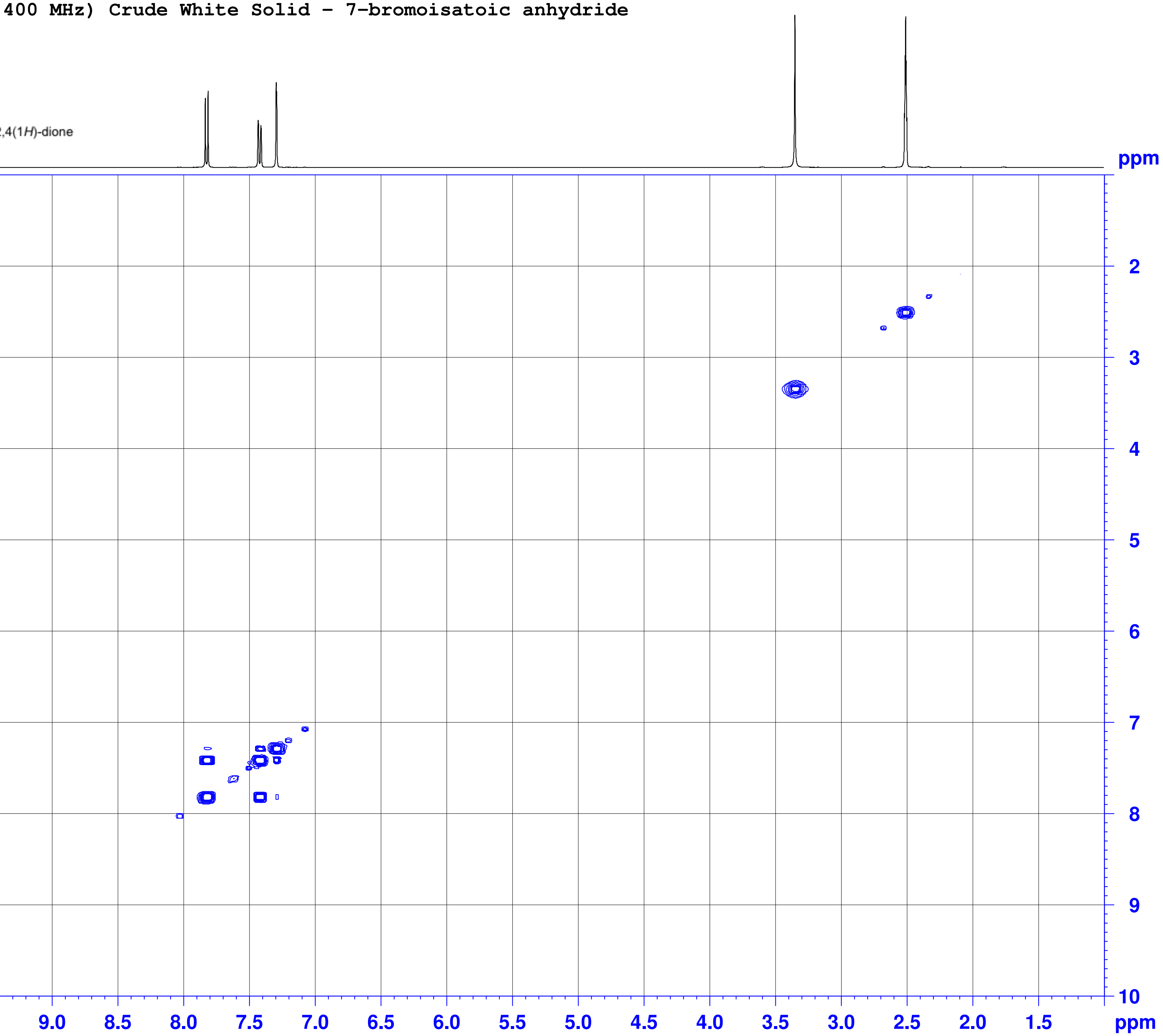

NGJ-8-029 (DMSO, 400 MHz) Crude White Solid - 7-bromoisatoic anhydride

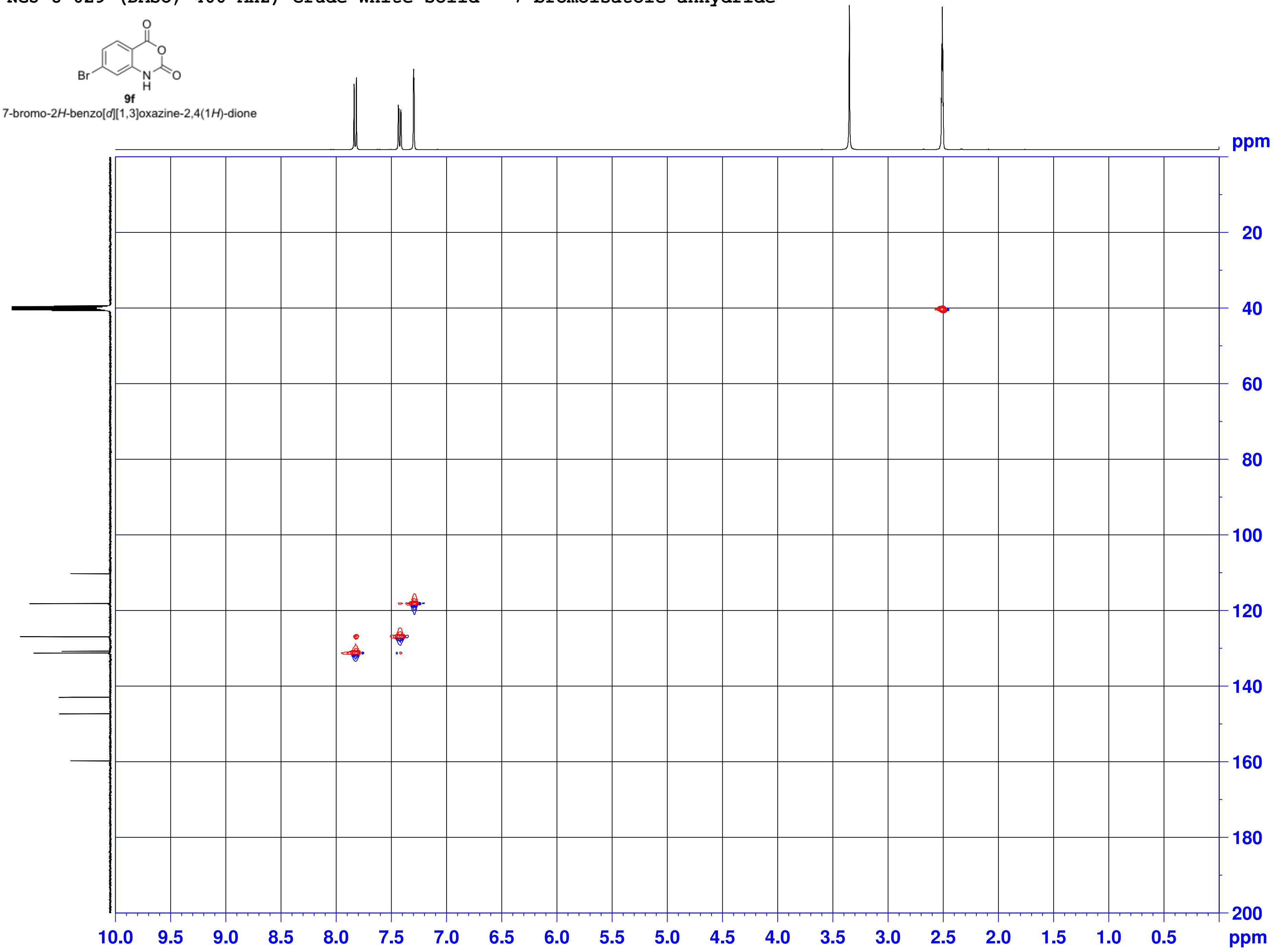

NGJ-8-029 (DMSO, 400 MHz) Crude White Solid - 7-bromoisatoic anhydride

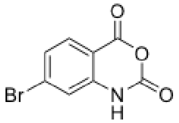

9f

7-bromo-2*H*-benzo[*d*][1,3]oxazine-2,4(1*H*)-dione

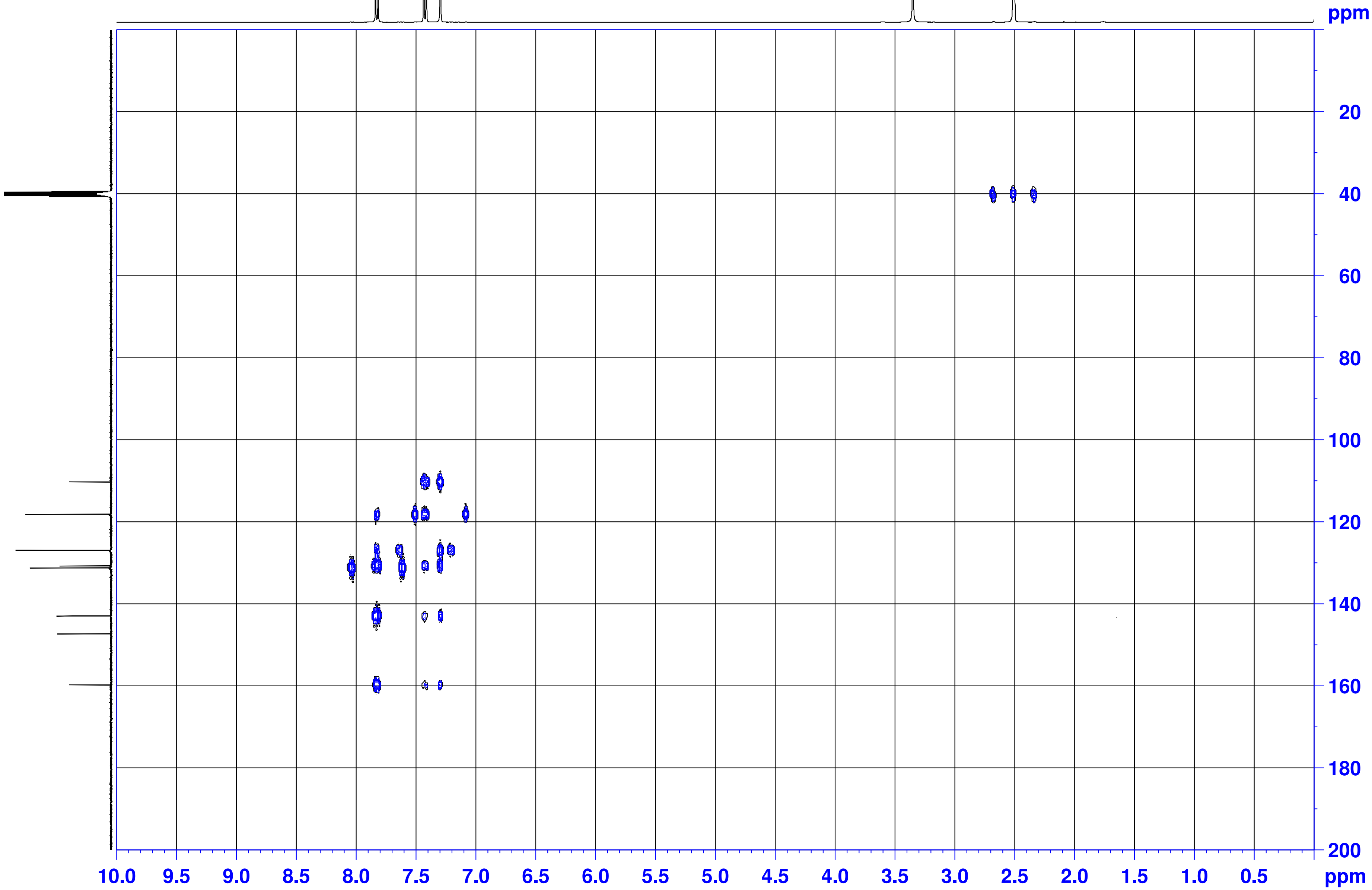

NGJ-8-034 (DMSO, 400 MHz) Pure Tan Solid - synthesis of 8-bromo isatoic anhydride

NAME	NGJ-8-034	
EXPNO	20	
PROCNO	1	
Date_	20170823	
Time	21.16	h
INSTRUM	spect	
PROBHD	Z108618_0161 (	
PULPROG	zg30	
TD	65536	
SOLVENT	DMSO	
NS	16	
DS	2	
SWH	8012.820	Hz
FIDRES	0.244532	Hz
AQ	4.0894966	sec
RG	203	
DW	62.400	usec
DE	6.50	usec
TE	297.6	K
D1	1.00000000	sec
TD0	1	
SFO1	400.1724710	MHz
NUC1	1H	
P1	9.88	usec
SI	65536	
SF	400.1699999	MHz
WDW	EM	
SSB	0	
LB	0.30	Hz
GB	0	
PC	1.00	

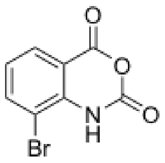

9h

8-bromo-2*H*-benzo[*d*][1,3]oxazine-2,4(1*H*)-dione

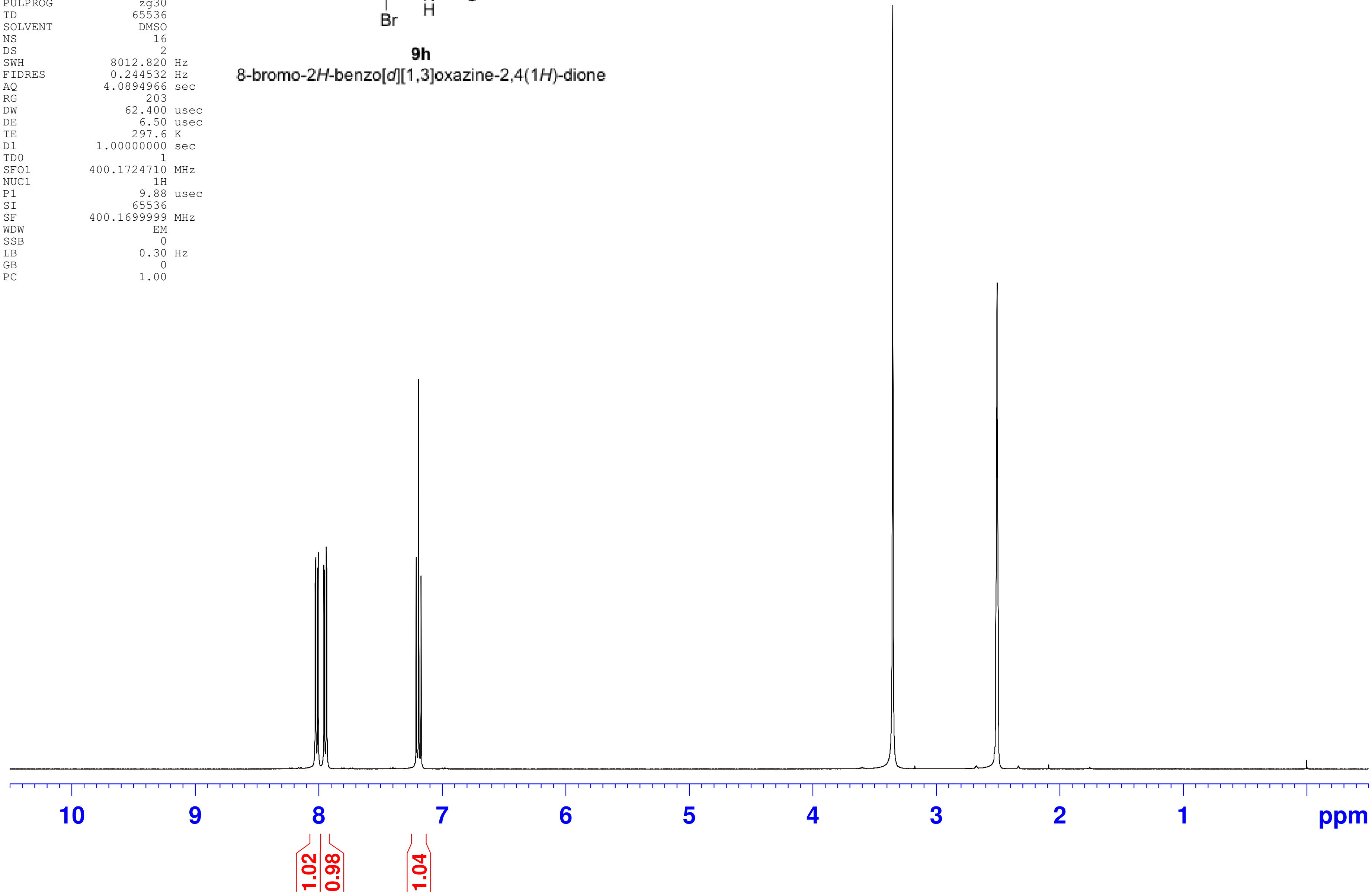

NGJ-8-034 (DMSO, 400 MHz) Pure Tan Solid - synthesis of 8-brom

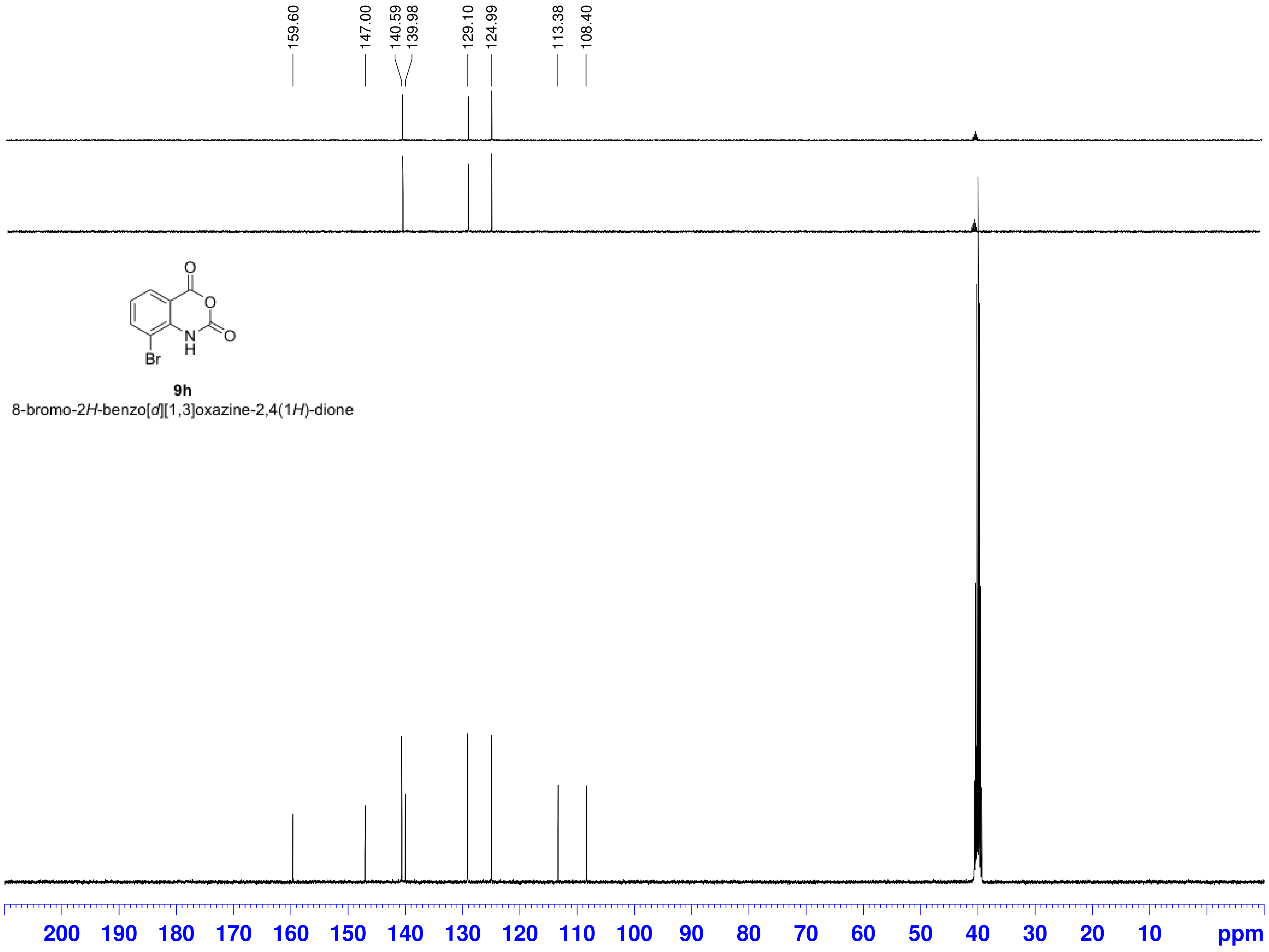

9h

8-bromo-2*H*-benzo[*d*][1,3]oxazine-2,4(1*H*)-dione

NGJ-8-034 (DMSO, 400 MHz) Pure Tan Solid - synthesis of 8-bromo isatoic anhydride

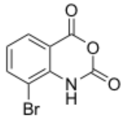

9h

8-bromo-2*H*-benzo[*d*][1,3]oxazine-2,4(1*H*)-dione

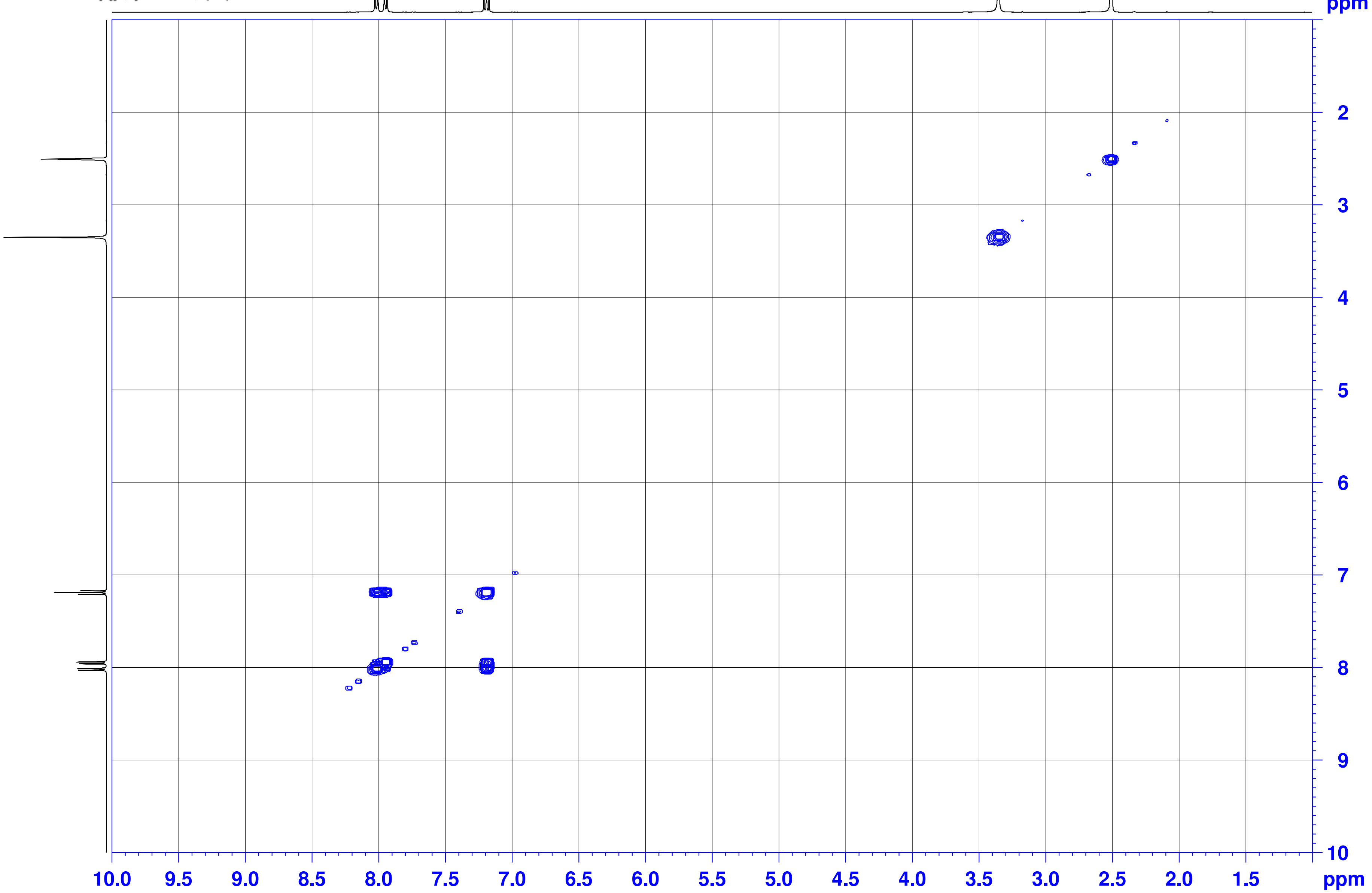

NGJ-8-034 (DMSO, 400 MHz) Pure Tan Solid - synthesis of 8-bromo isatoic anhydride

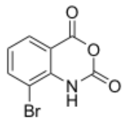

9h

8-bromo-2*H*-benzo[*d*][1,3]oxazine-2,4(1*H*)-dione

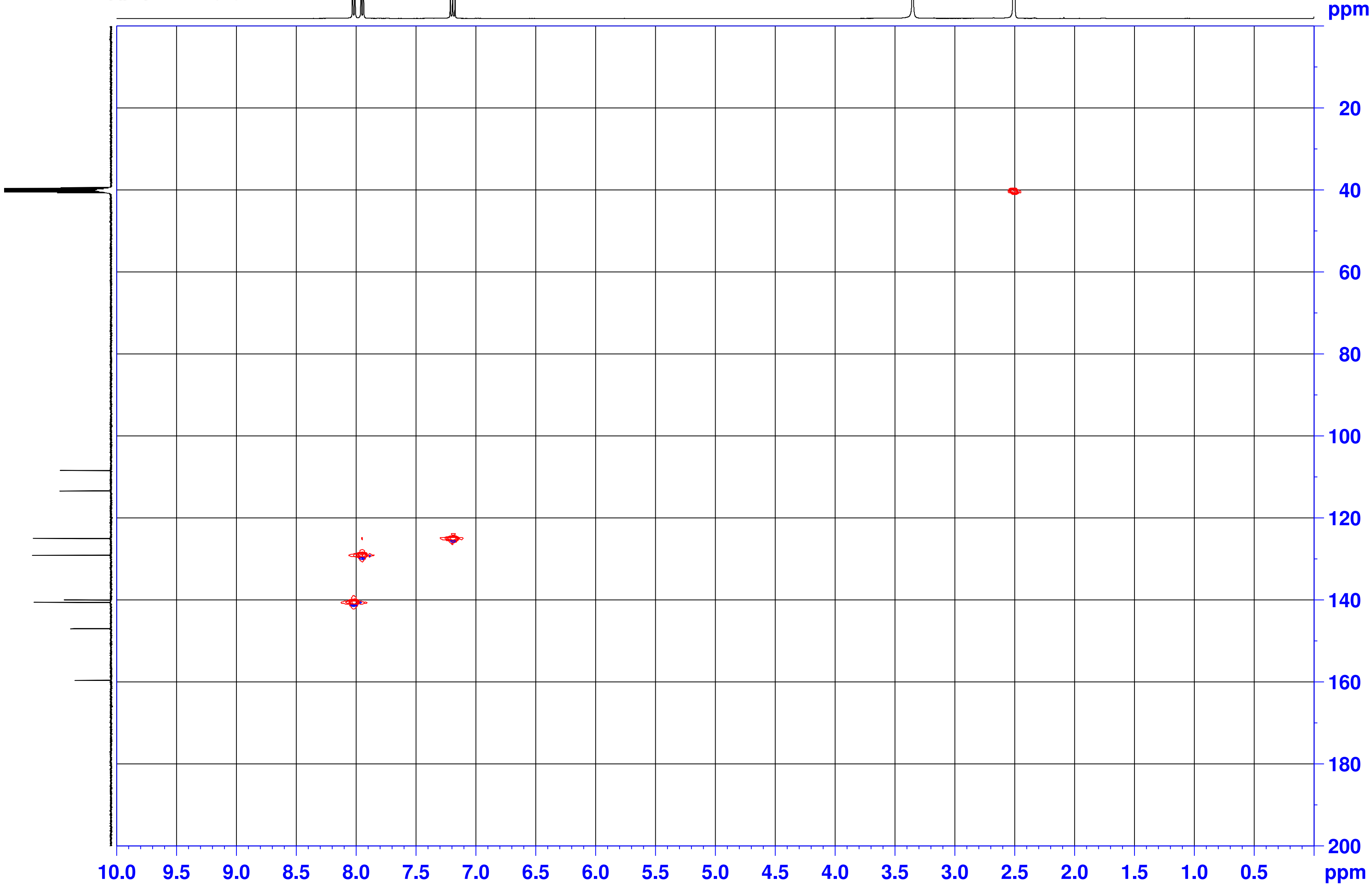

NGJ-8-034 (DMSO, 400 MHz) Pure Tan Solid - synthesis of 8-bromo isatoic anhydride

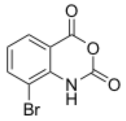

9h

8-bromo-2*H*-benzo[*d*][1,3]oxazine-2,4(1*H*)-dione

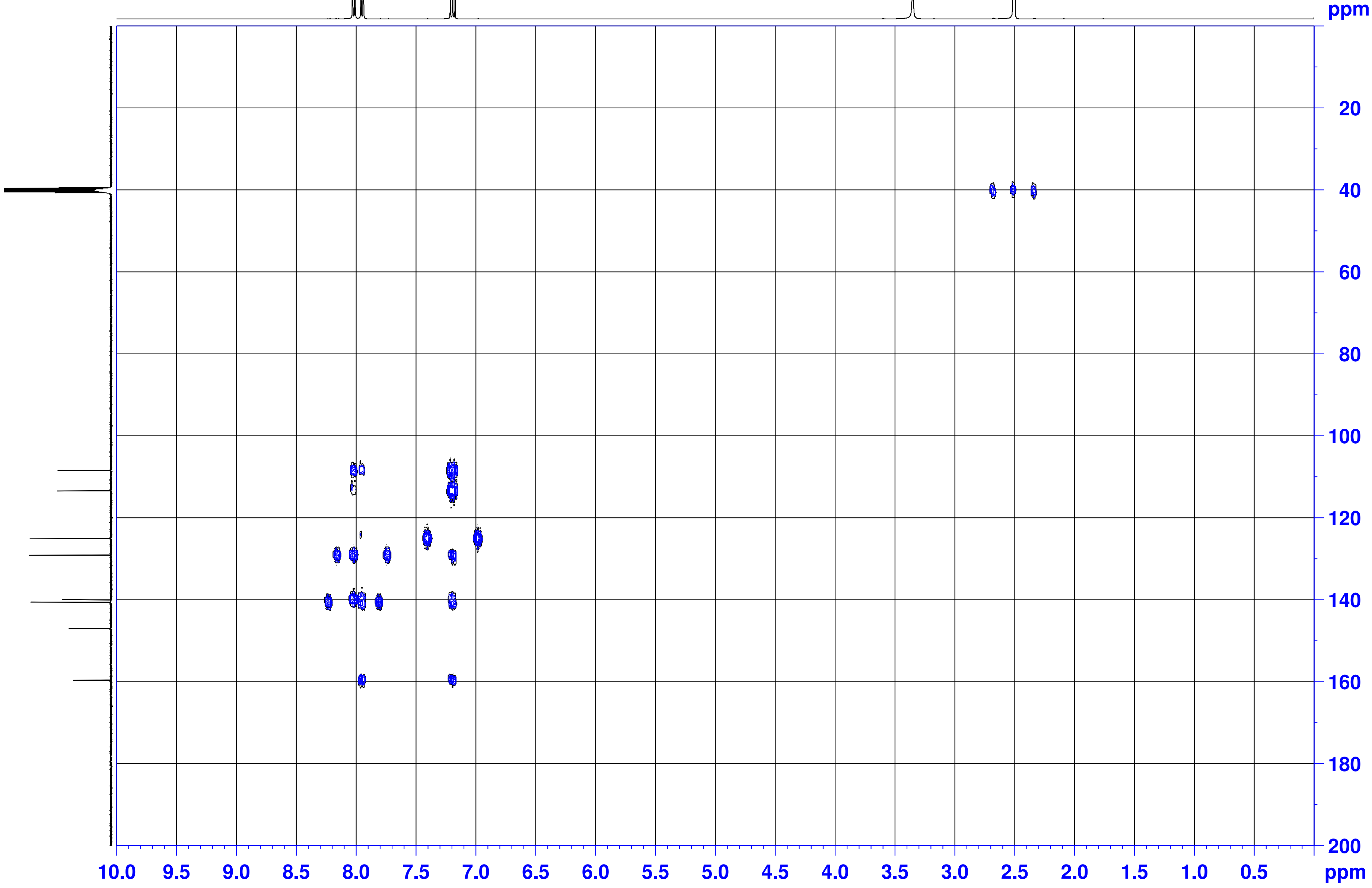

AHP-1-020

Collection time: Tue Dec 20 09:33:07 2016 (GMT-06:00)

Number of sample scans: 32
Number of background scans: 16
Resolution: 4.000
Sample gain: 8.0
Mirror velocity: 0.6329
Aperture: 100.00

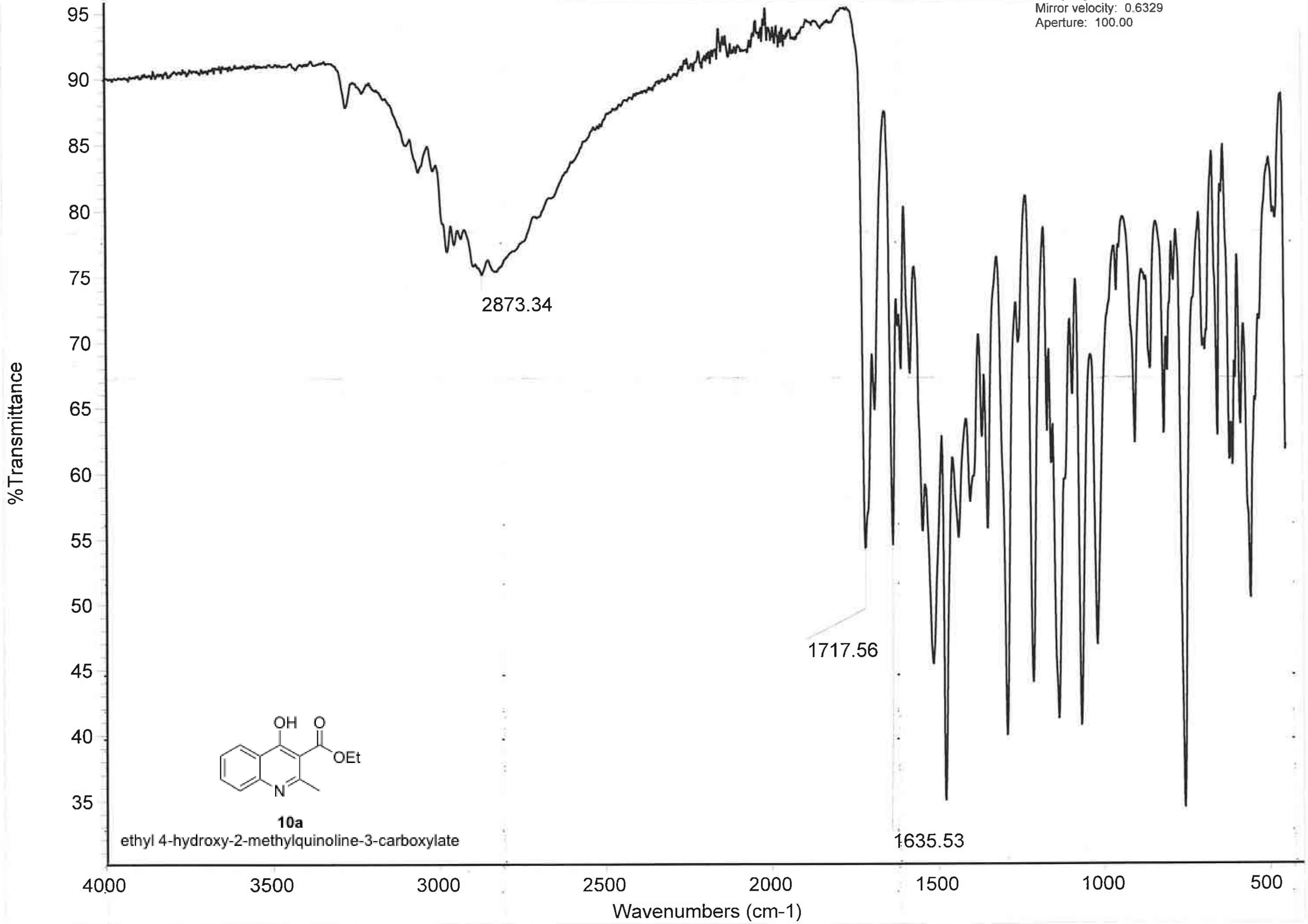

AHP-1-020 (DMSO, 400 MHz) Pure White Solid- 4-hydroxy quinoline

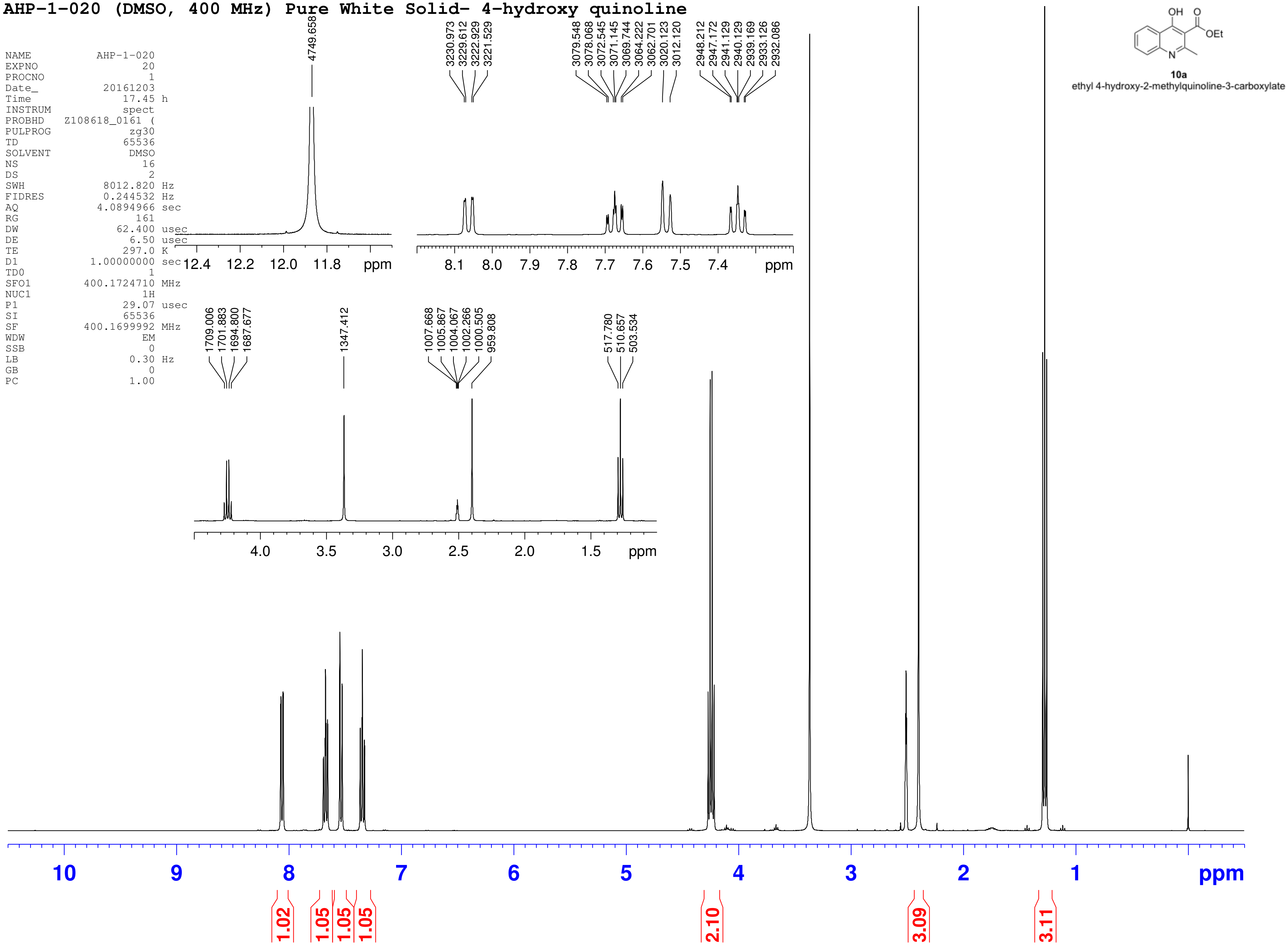

10a
ethyl 4-hydroxy-2-methylquinoline-3-carboxylate

AHP-1-020 (DMSO, 400 MHz)

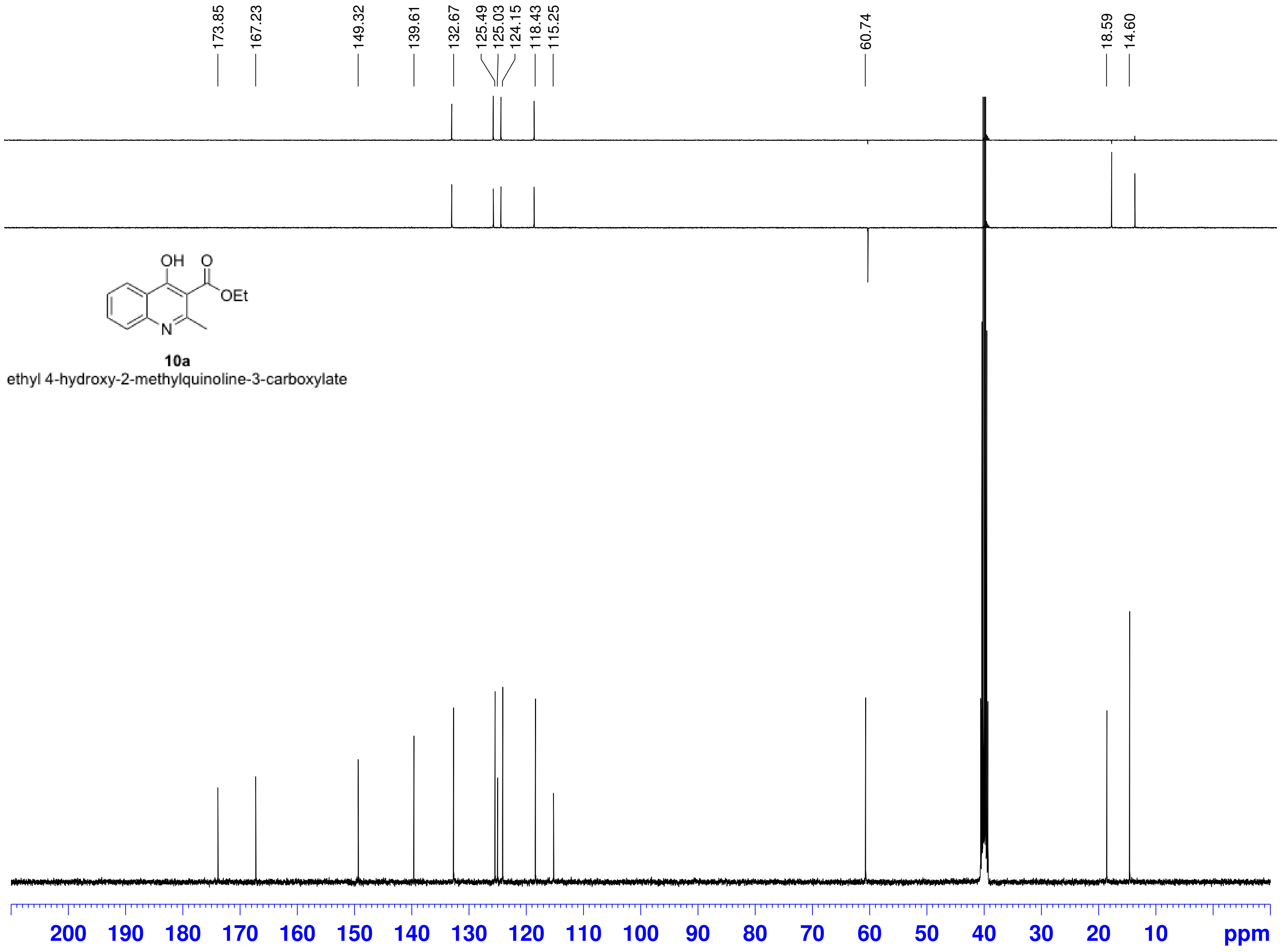

AHP-1-020 (DMSO, 400 MHz)

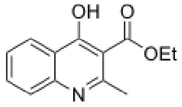

10a

ethyl 4-hydroxy-2-methylquinoline-3-carboxylate

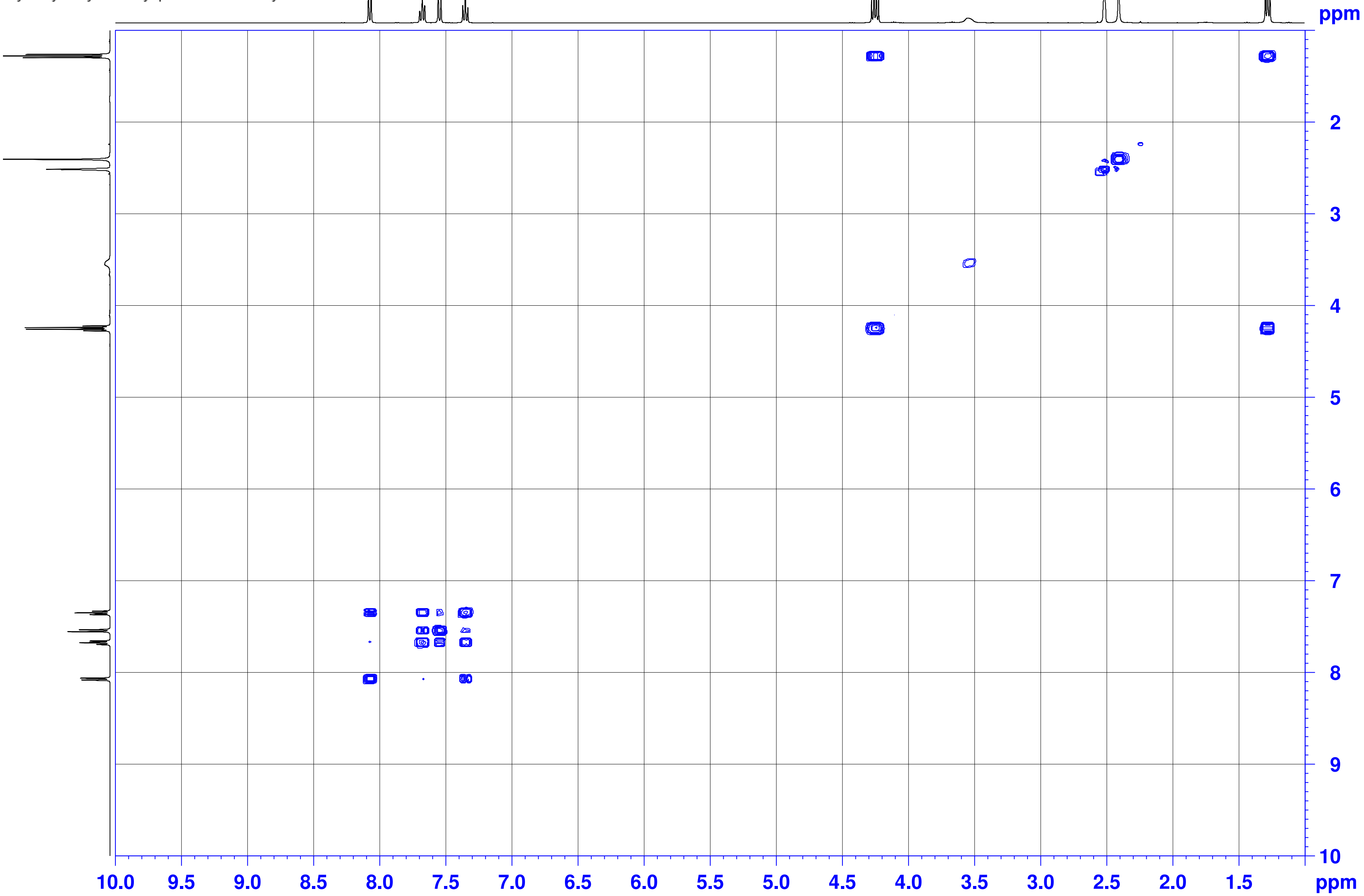

AHP-1-020 (DMSO, 400 MHz)

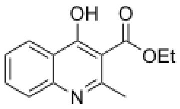

10a

ethyl 4-hydroxy-2-methylquinoline-3-carboxylate

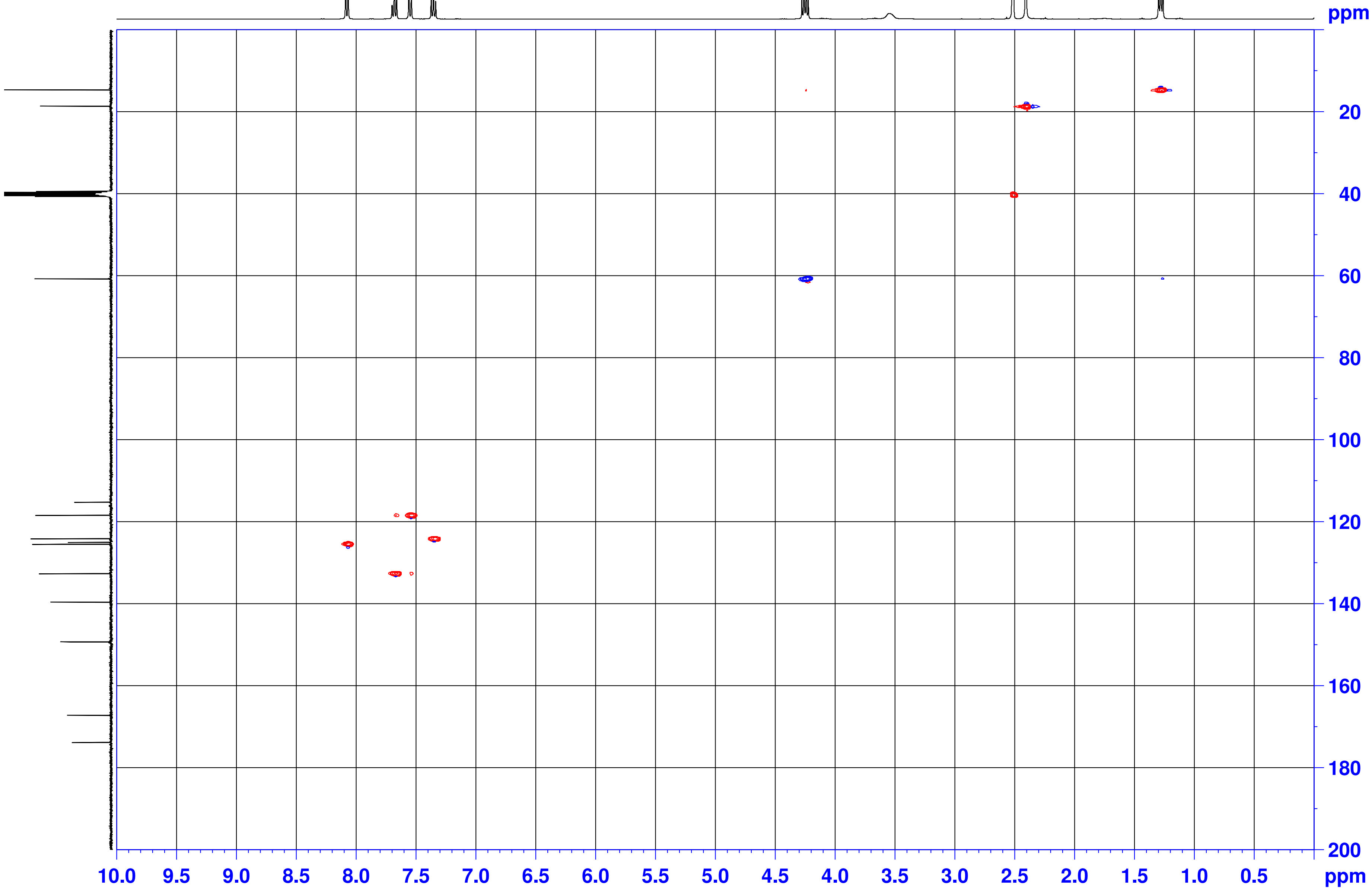

AHP-1-020 (DMSO, 400 MHz) Pure White Solid- 4-hydroxy quinoline

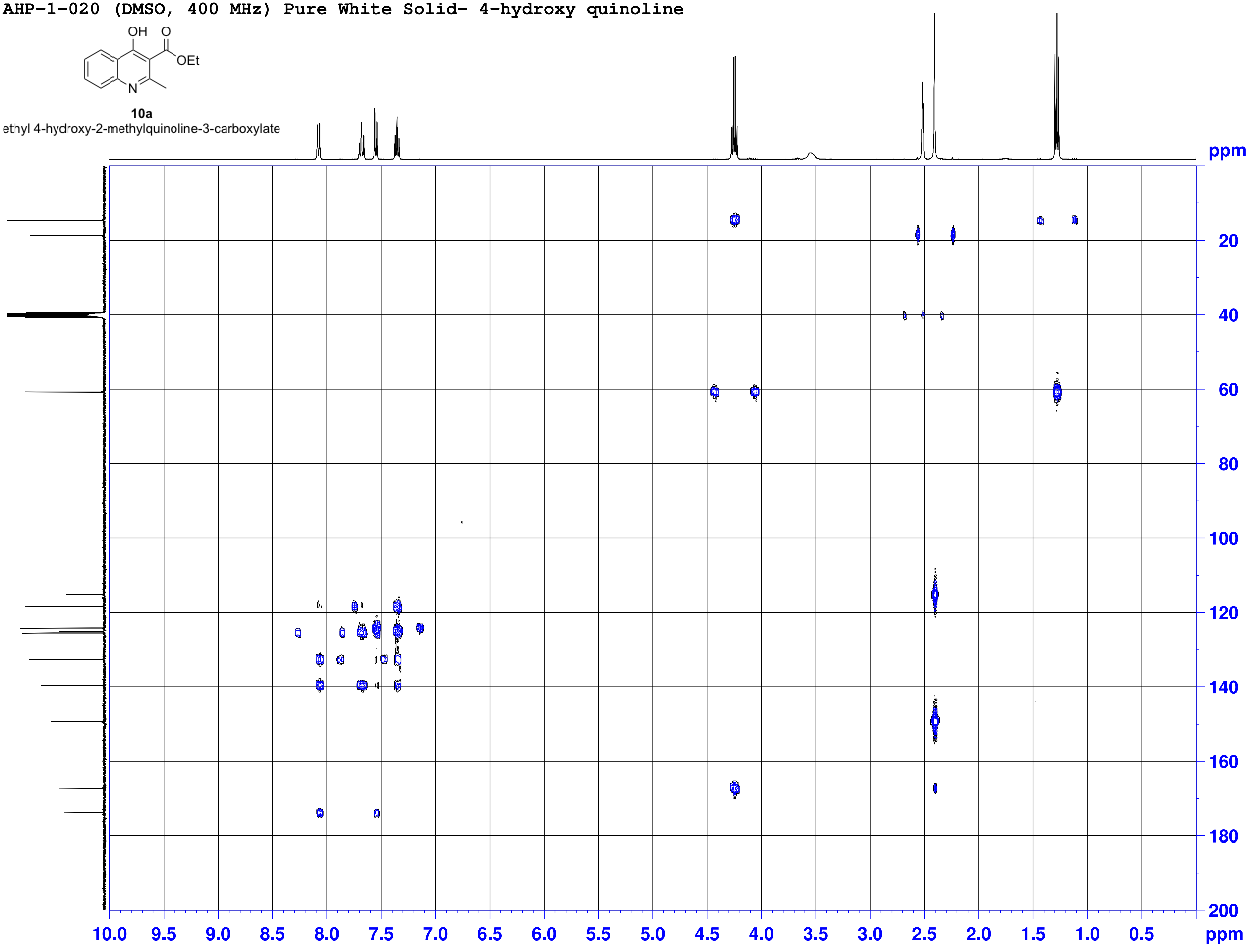

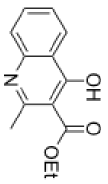

10a

ethyl 4-hydroxy-2-methylquinoline-3-carboxylate

Atlantic Microlab, Inc.

Sample No. AHP-1-020

6180 Atlantic Blvd. Suite M
Norcross, GA 30071
www.atlanticmicrolab.com

Company/School University of Southern Mississippi
Dept. Chemistry and Biochemistry
Address 118 College Dr. #5043
City, State, Zip Hattiesburg, MS 39406
Name Matthew G. Donahue Date 2/25/17
Phone 614-203-1123

Professor/Supervisor: Matthew G. Donahue
PO# / CC# Visa 5794

Element	Theory	Found	
C	67.52	66.52	66.68
H	5.67	5.76	5.73
		NO CHARGE FOR DUPLICATES	

Single ☒ Duplicate ☐

Elements Present: $C_{13}H_{13}NO_3$

Analyze for: C, H

Hygroscopic ☐ Explosive ☐
M.P. ______ B.P. ______

To be dried: Yes ☐ No ☒
Temp. ______ Vac. ______ Time ______

Rush Service ☐ Rush service guarantees analyses will be completed and results available by 5 PM EST on the day the sample is received by 11 AM.

Include Email Address or FAX # Below

matthew.donahue@usm.edu

Date Received MAR 13 P.M. Date Completed MAR 14 2017

Remarks:

Mass Spectrum List Report

Analysis Info

Analysis Name	G:\Data\apexdata111116\EBC-1-009_pos_000001.d
Method	Neg_DOM_032112
Sample Name	EBC-1-009
Comment	EBC-1-009 in MeOH:THF with NaCl added

Acquisition Date	11/11/2016 3:33:29 PM
Operator	FTMS_USER
Instrument	apex-Qe

Sample Name EBC-1-009

Exact Mass of $C_{12}H_{12}BrNO_3Na^+$ = 331.989277 m/z

Mass Observed with signature = 331.988788 m/z

Difference < -1.5 ppm

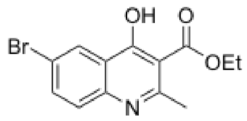

10b

ethyl 6-bromo-4-hydroxy-2-methylquinoline-3-carboxylate

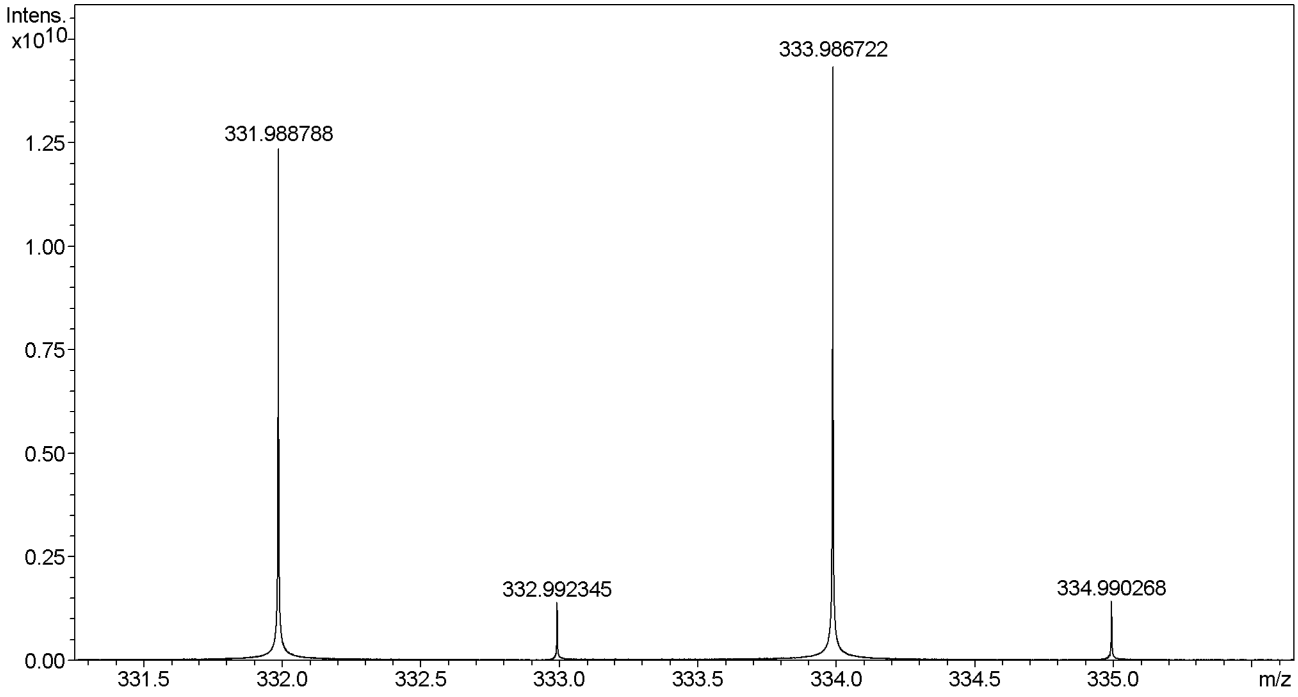

#	m/z	I
1	184.283963	24768120
2	184.318078	28267768
3	184.319972	28174904
4	215.088747	72876717
5	216.933685	53887112
6	218.207464	589511162
7	219.492165	49374700
8	220.812057	469930650
9	263.965289	65200142
10	265.963231	78783632
11	281.975883	59984049
12	283.973876	50207343
13	310.007021	204095469
14	312.004973	247188977
15	314.341485	40748245
16	331.988788	12405816634
17	332.989875	344055448
18	332.992345	1399439001
19	333.203861	27184092
20	333.597258	31554641

Mass Spectrum List Report

#	m/z	I
21	333.984417	3546070972
22	333.986722	14441103293
23	334.224004	38123002
24	334.987734	422723756
25	334.990268	1437450412
26	335.993673	65921381
27	337.965737	27704887
28	347.962923	103942939
29	349.182847	33175826
30	349.960911	125478823
31	351.213893	29969709
32	360.323427	110611657
33	367.208688	25808934
34	368.823514	53026242
35	368.834576	90639799
36	368.841783	77305264
37	370.404041	32155472
38	393.209189	27007712
39	393.260477	28799651
40	393.297140	167194231
41	394.300536	24820169
42	398.240992	121313202
43	399.244370	26374958
44	413.265883	81332931
45	425.287009	51091939
46	525.375814	53423870
47	550.628349	34942131
48	552.607372	41453689
49	567.386212	36247364
50	569.401918	138683102
51	570.405462	44318879
52	611.412388	63896188
53	612.415953	25293512
54	613.428127	203252495
55	614.431386	75319104
56	640.988804	254165193
57	641.992203	68068163
58	642.629037	52688075
59	642.986753	529171877
60	643.632136	26001190
61	643.989893	145917945
62	644.984851	249907766
63	645.987989	81747044
64	655.438894	64261521
65	656.441816	30328588
66	657.454308	221831290
67	658.457608	87695830
68	668.644704	29338704
69	672.926102	60968722
70	674.924044	132829198
71	675.927732	26824827
72	676.921573	64012507
73	685.435271	61993567
74	686.438601	27312733
75	699.464883	48537422
76	700.468086	29910751
77	701.480628	176123497
78	702.483870	66498031
79	711.506912	42365115
80	717.454283	27186339
81	722.265597	26638614
82	724.263715	29411219
83	743.491343	34842998
84	744.344042	106729704

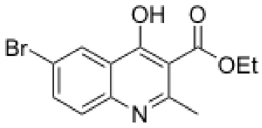

10b
ethyl 6-bromo-4-hydroxy-2-methylquinoline-3-carboxylate

Mass Spectrum List Report

#	m/z	I
85	745.347253	38457413
86	745.506598	111489068
87	746.341851	98832296
88	746.510448	42680205
89	747.345277	44797709
90	789.532665	42125751
91	877.534532	27960792
92	877.673538	30318135
93	919.683717	36163175
94	921.699388	34788645
95	963.710361	26770656
96	965.725532	28994480
97	994.434676	35133536
98	996.433484	27383399
99	997.436067	25610600
100	1009.752101	29787331

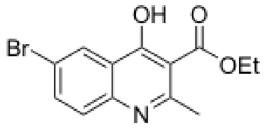

10b
ethyl 6-bromo-4-hydroxy-2-methylquinoline-3-carboxylate

SMB-1-019 (DMSO, 400 MHz)

NAME	SMB-1-019	
EXPNO	60	
PROCNO	1	
Date_	20170720	
Time	20.24	h
INSTRUM	spect	
PROBHD	Z108618_0161 (	
PULPROG	zg30	
TD	65536	
SOLVENT	DMSO	
NS	16	
DS	2	
SWH	8012.820	Hz
FIDRES	0.244532	Hz
AQ	4.0894966	sec
RG	181	
DW	62.400	usec
DE	6.50	usec
TE	298.1	K
D1	1.00000000	sec
TD0	1	
SFO1	400.1724710	MHz
NUC1	1H	
P1	9.88	usec
SI	65536	
SF	400.1699995	MHz
WDW	EM	
SSB	0	
LB	0.30	Hz
GB	0	
PC	1.00	

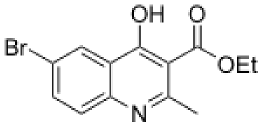

10b

ethyl 6-bromo-4-hydroxy-2-methylquinoline-3-carboxylate

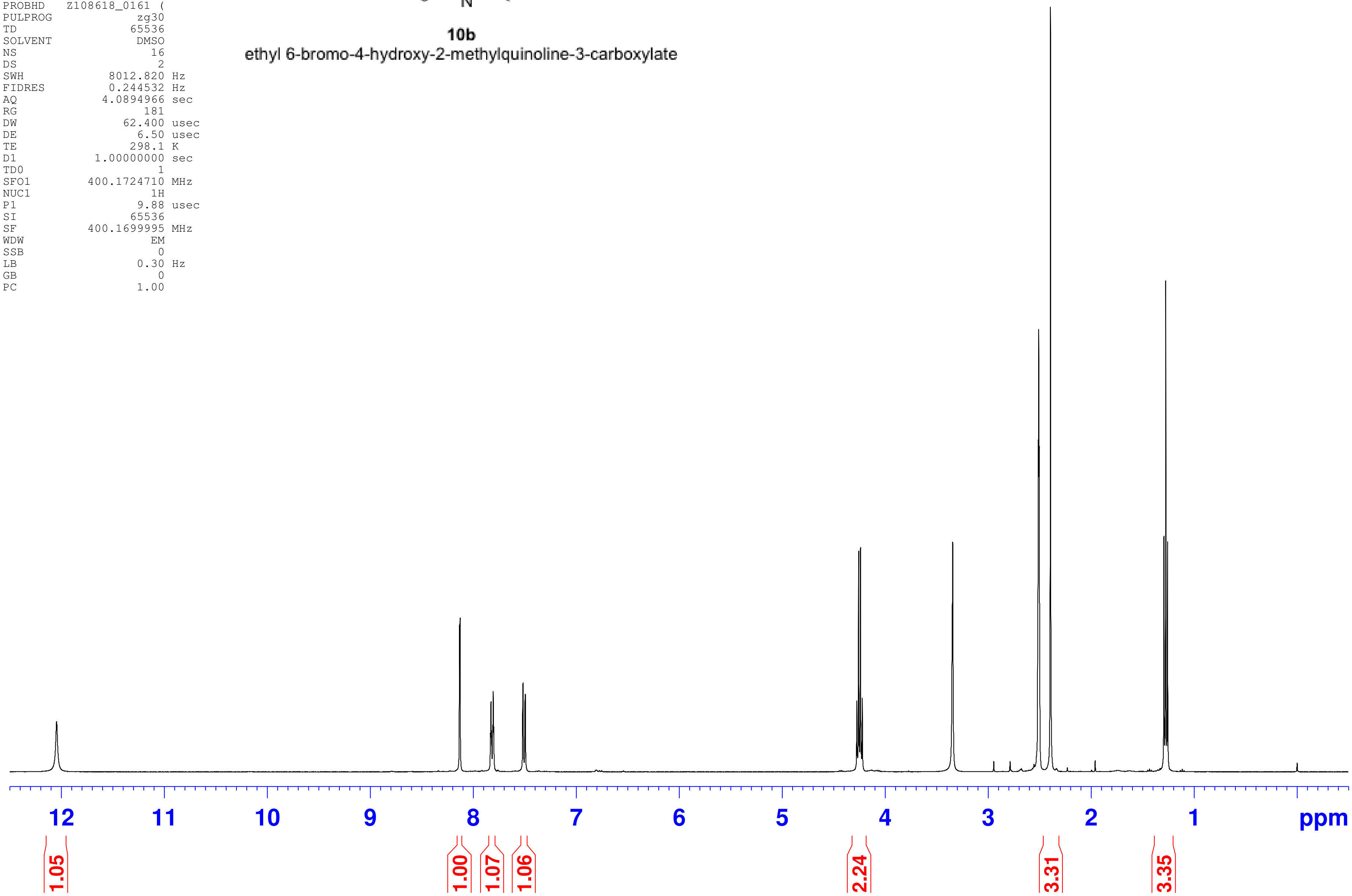

SMB-1-019 (DMSO, 400 MHz)

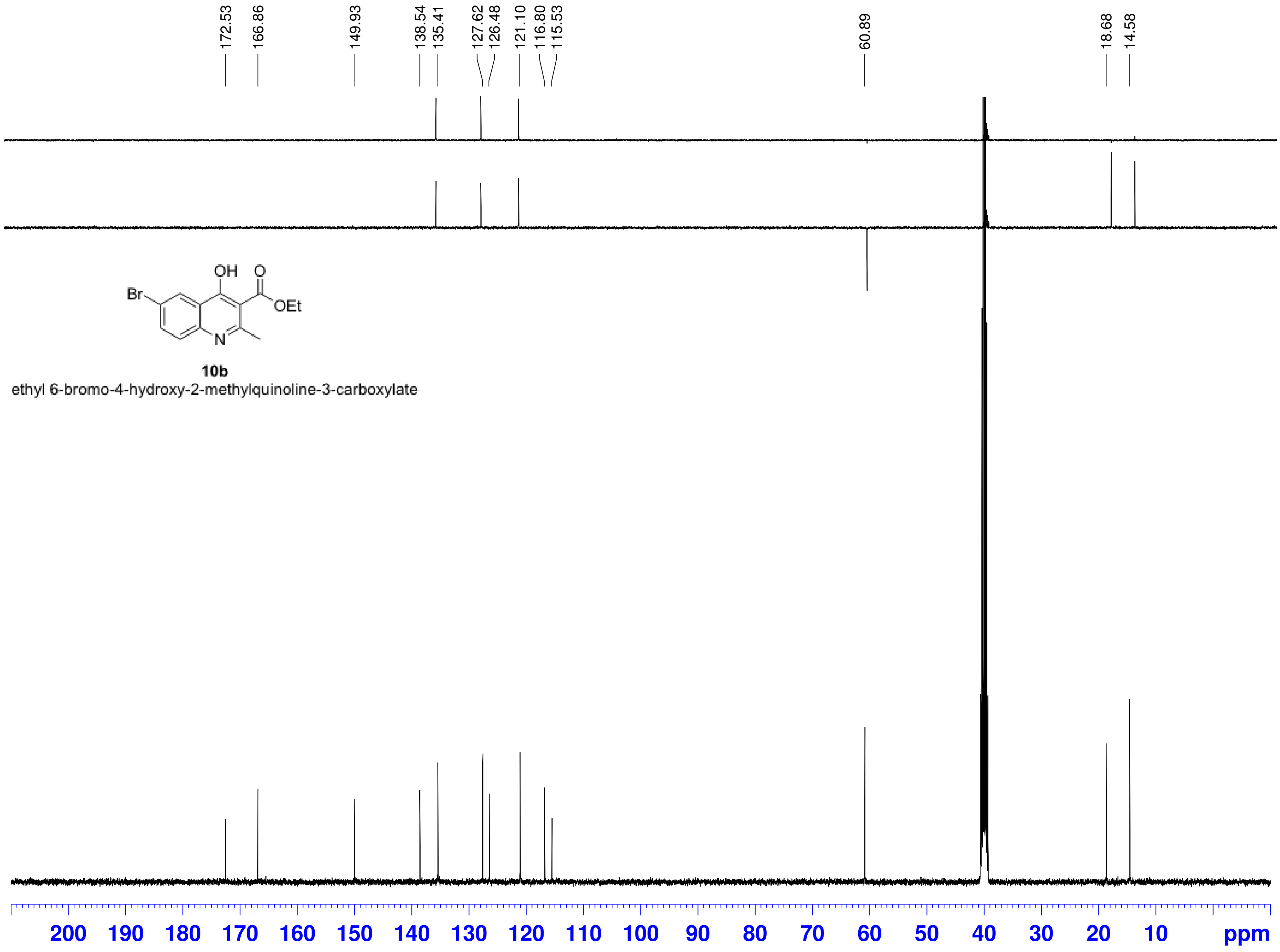

SMB-1-019 (DMSO, 400 MHz)

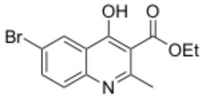

10b

ethyl 6-bromo-4-hydroxy-2-methylquinoline-3-carboxylate

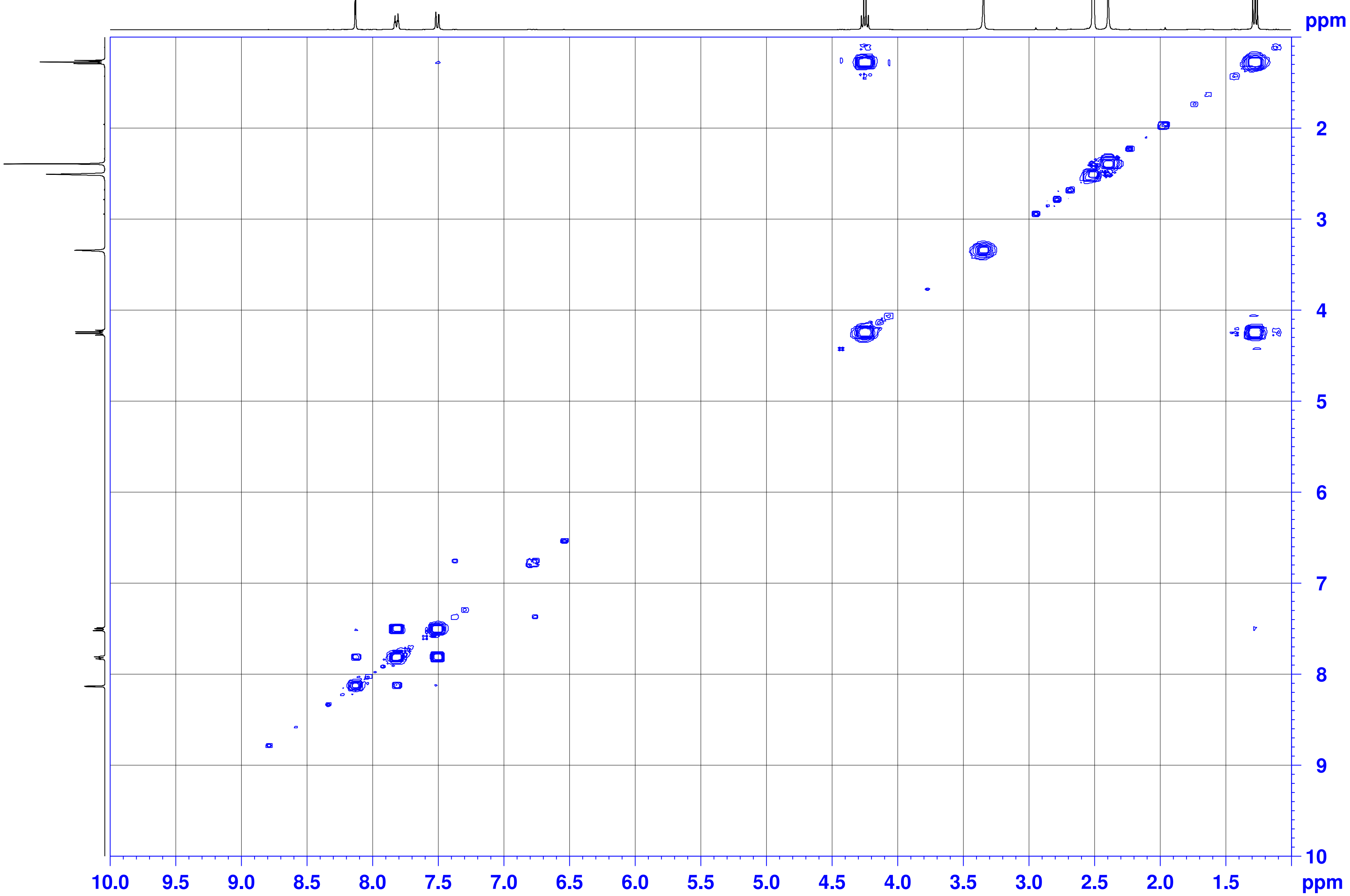

SMB-1-019 (DMSO, 400 MHz)

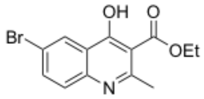

10b

ethyl 6-bromo-4-hydroxy-2-methylquinoline-3-carboxylate

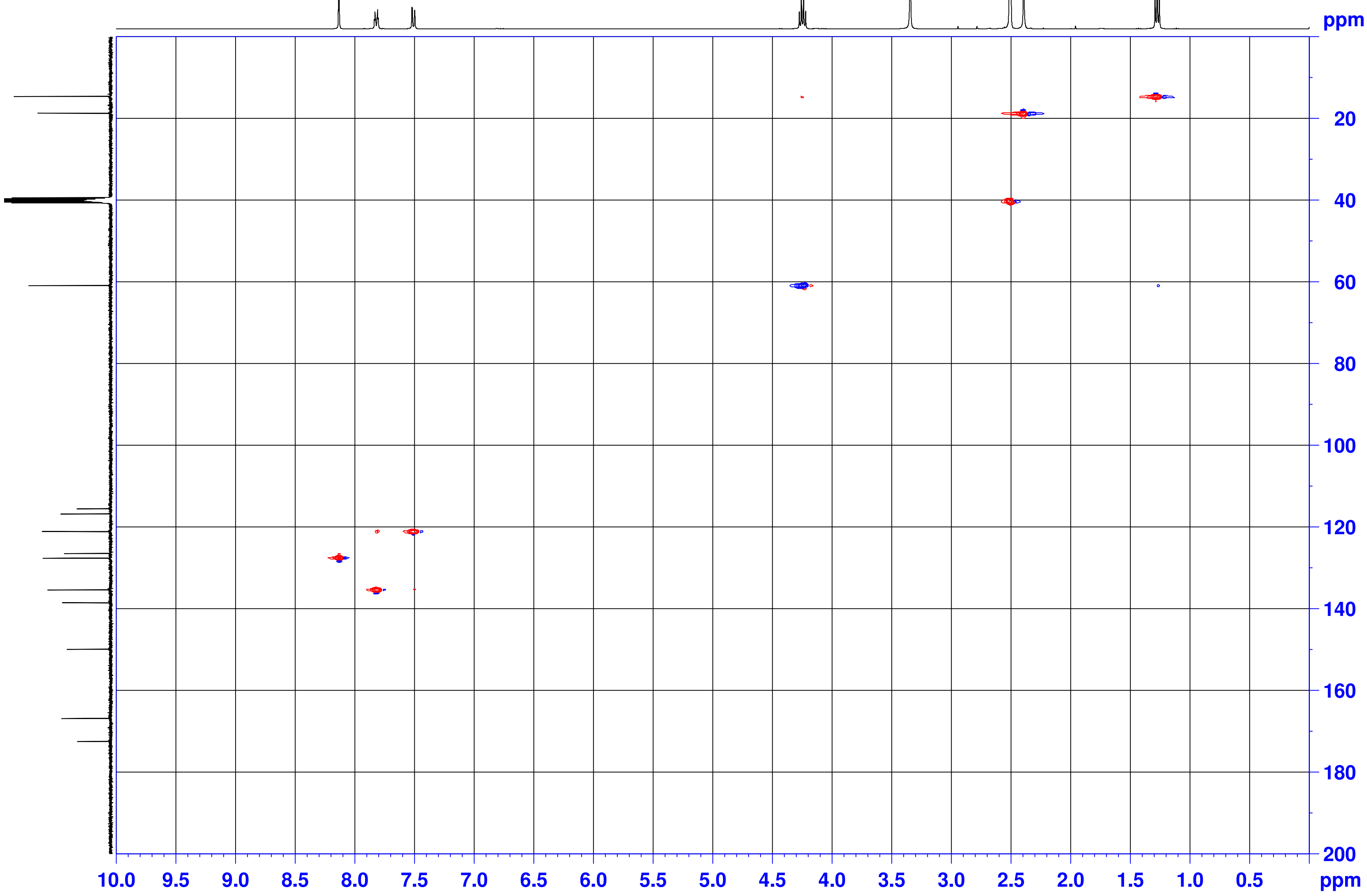

SMB-1-019 (DMSO, 400 MHz)

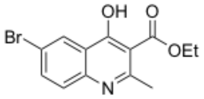

10b

ethyl 6-bromo-4-hydroxy-2-methylquinoline-3-carboxylate

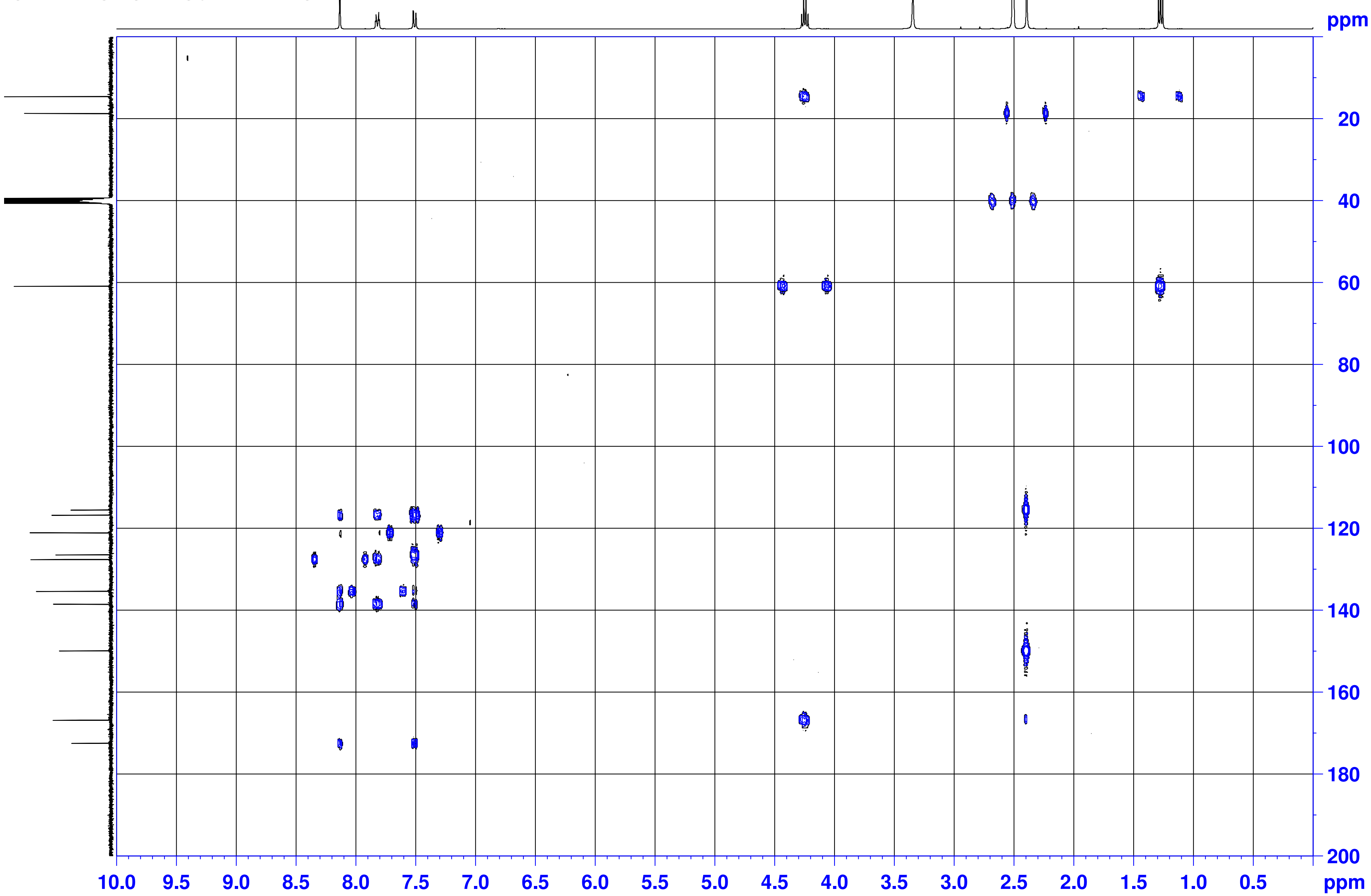

SMB-1-020
Collection time: Fri Jul 28 14:48:50 2017 (GMT-06:00)

Number of sample scans: 8
Number of background scans: 8
Resolution: 2.000
Sample gain: 8.0
Mirror velocity: 0.6329
Aperture: 100.00

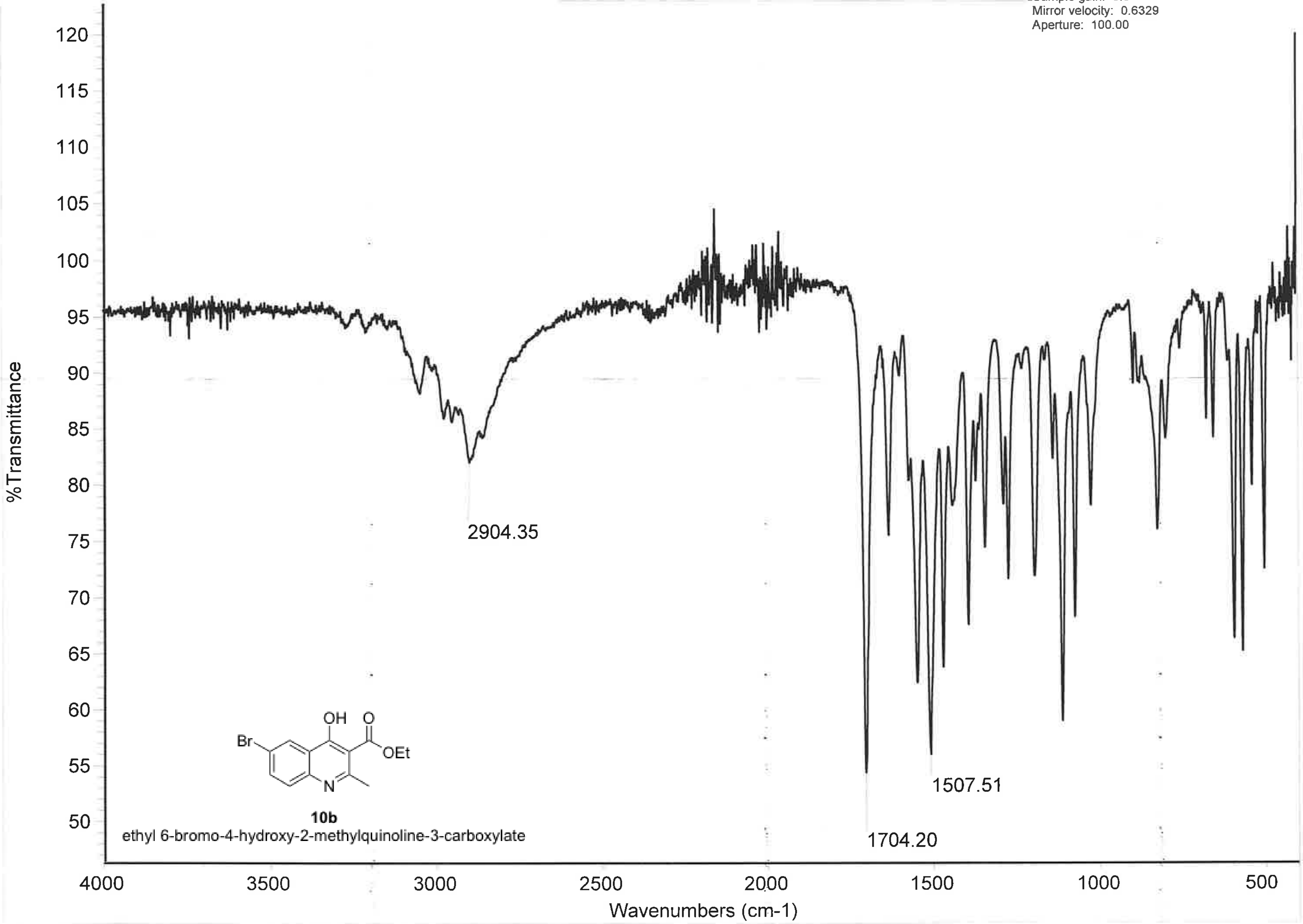

10b

ethyl 6-bromo-4-hydroxy-2-methylquinoline-3-carboxylate

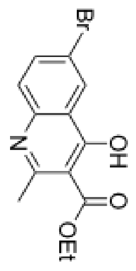

10b

ethyl 6-bromo-4-hydroxy-2-methylquinoline-3-carboxylate

Atlantic Microlab, Inc.

Sample No. SMB-1-020

6180 Atlantic Blvd. Suite M
Norcross, GA 30071
www.atlanticmicrolab.com

Company/School: University of Southern Mississippi
Dept.: Chemistry and Biochemistry
Address: 118 College Dr. #5043
City, State, Zip: Hattiesburg, MS 39406
Name: Matthew G. Donahue Date: 2/25/17
Phone: 614-203-1123

Professor/Supervisor: Matthew G. Donahue
PO# / CC#: Visa 5794)

Element	Theory	Found	
C	50.34	50.10	
H	3.90	3.88	

Single ☒ Duplicate ☐

Elements Present: $C_{13}H_{12}BrNO_3$

Analyze for: C, H

Hygroscopic ☐ Explosive ☐
M.P. ______ B.P. ______

To be dried: Yes ☐ No ☒
Temp. ______ Vac. ______ Time ______

Rush Service ☐ Rush service guarantees analyses will be completed and results available by 5 PM EST on the day the sample is received by 11 AM.

Include Email Address or FAX # Below
matthew.donahue@usm.edu

Date Received: MAR 13 P.M. Date Completed: MAR 14 2017

Remarks:

AHP-1-019

Collection time: Fri Jul 28 14:53:08 2017 (GMT-06:00)

Number of sample scans: 8
Number of background scans: 8
Resolution: 2.000
Sample gain: 8.0
Mirror velocity: 0.6329
Aperture: 100.00

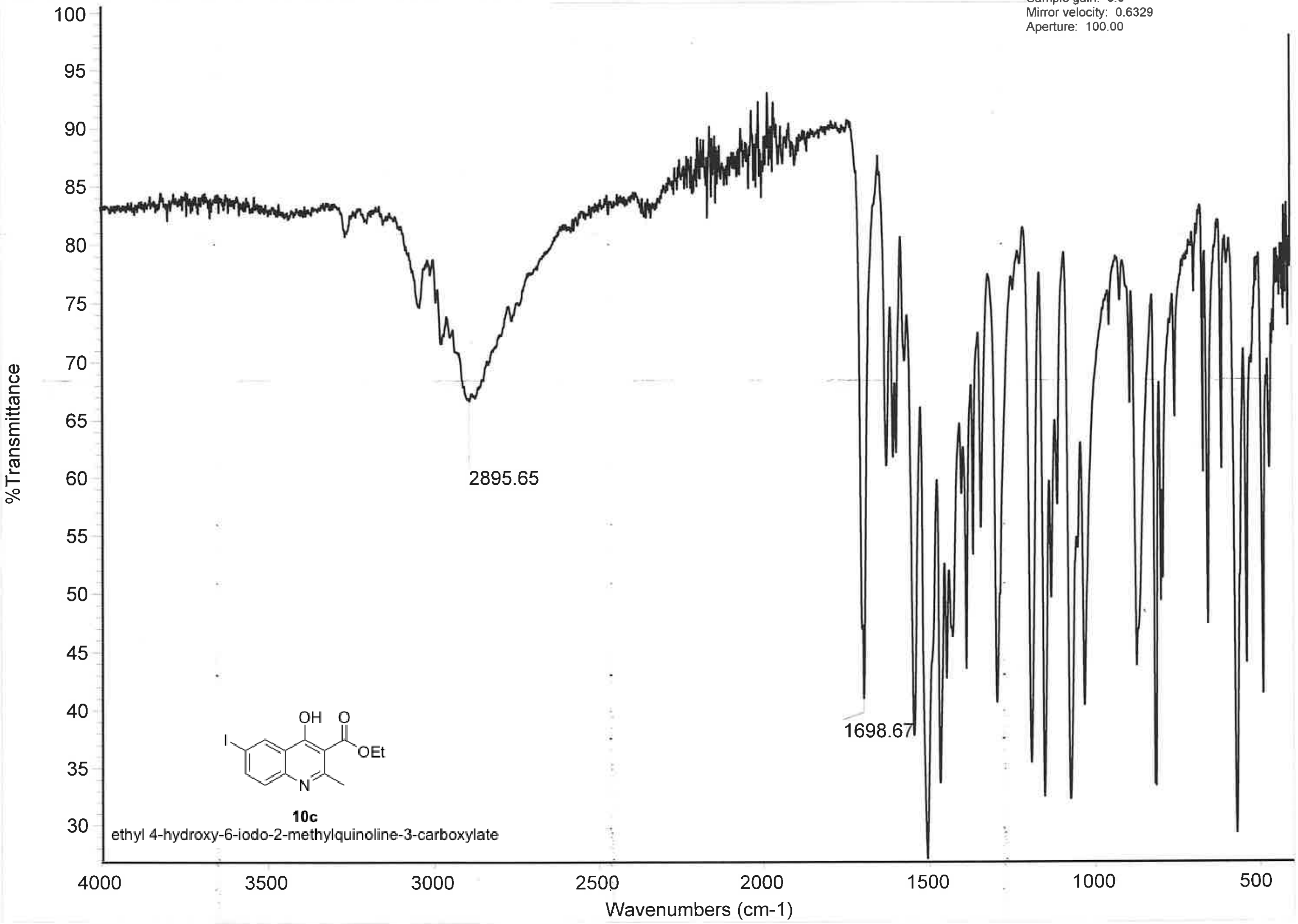

10c

ethyl 4-hydroxy-6-iodo-2-methylquinoline-3-carboxylate

AHP-1-019 (DMSO, 400 MHz) Pure White Solid - Iodo quinoline

NAME	AHP-1-019
EXPNO	20
PROCNO	1
Date_	20170225
Time	13.06 h
INSTRUM	spect
PROBHD	Z108618_0161 (
PULPROG	zg30
TD	65536
SOLVENT	DMSO
NS	16
DS	2
SWH	8012.820 Hz
FIDRES	0.244532 Hz
AQ	4.0894966 sec
RG	161
DW	62.400 usec
DE	6.50 usec
TE	297.3 K
D1	1.00000000 sec
TD0	1
SFO1	400.1724710 MHz
NUC1	1H
P1	9.88 usec
SI	65536
SF	400.1700000 MHz
WDW	EM
SSB	0
LB	0.30 Hz
GB	0
PC	1.00

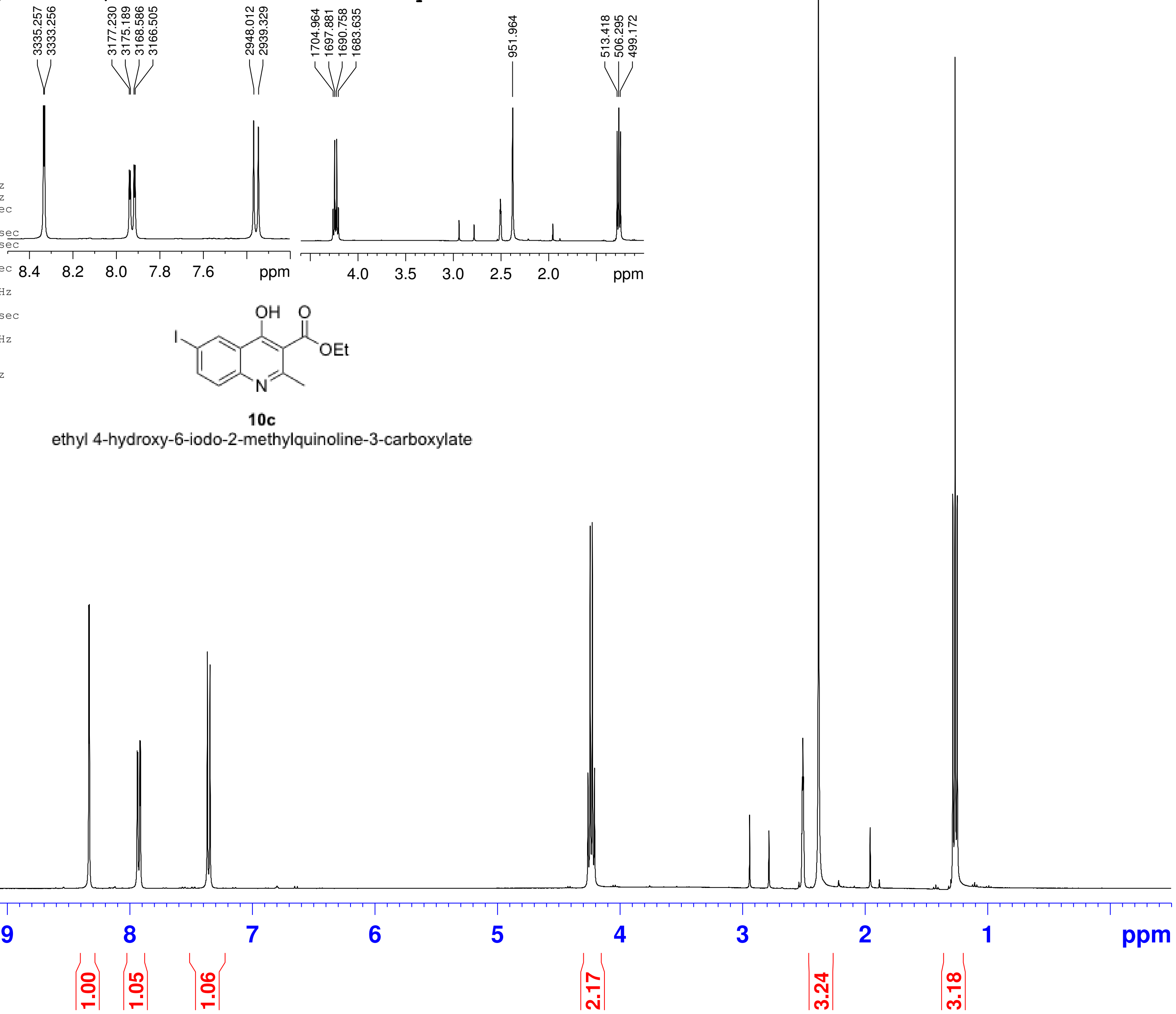

10c

ethyl 4-hydroxy-6-iodo-2-methylquinoline-3-carboxylate

AHP-1-019 (DMSO, 400 MHz) Pure White Solid - Iodo quinoline

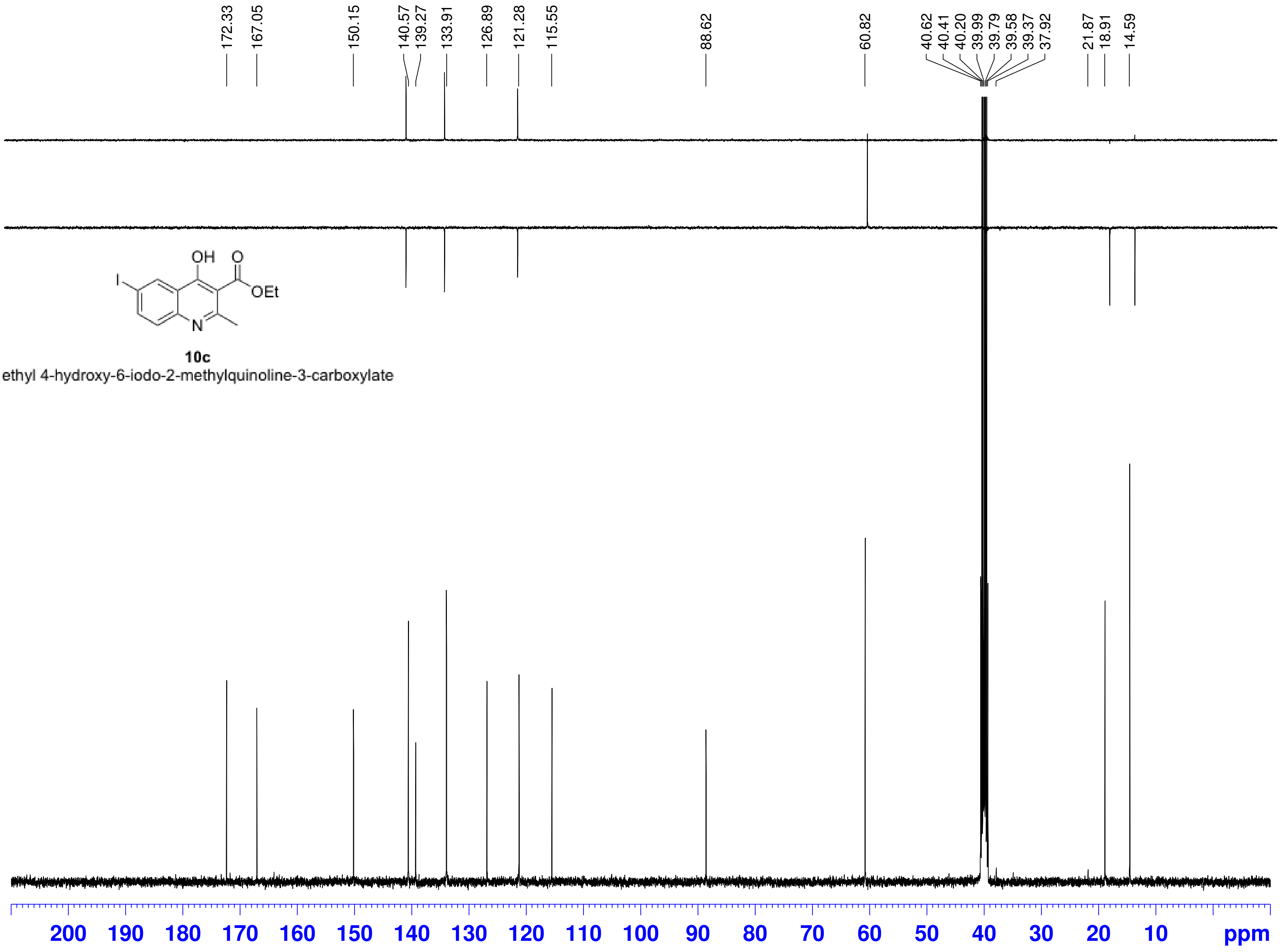

10c

ethyl 4-hydroxy-6-iodo-2-methylquinoline-3-carboxylate

AHP-1-019 (DMSO, 400 MHz)

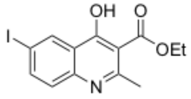

10c

ethyl 4-hydroxy-6-iodo-2-methylquinoline-3-carboxylate

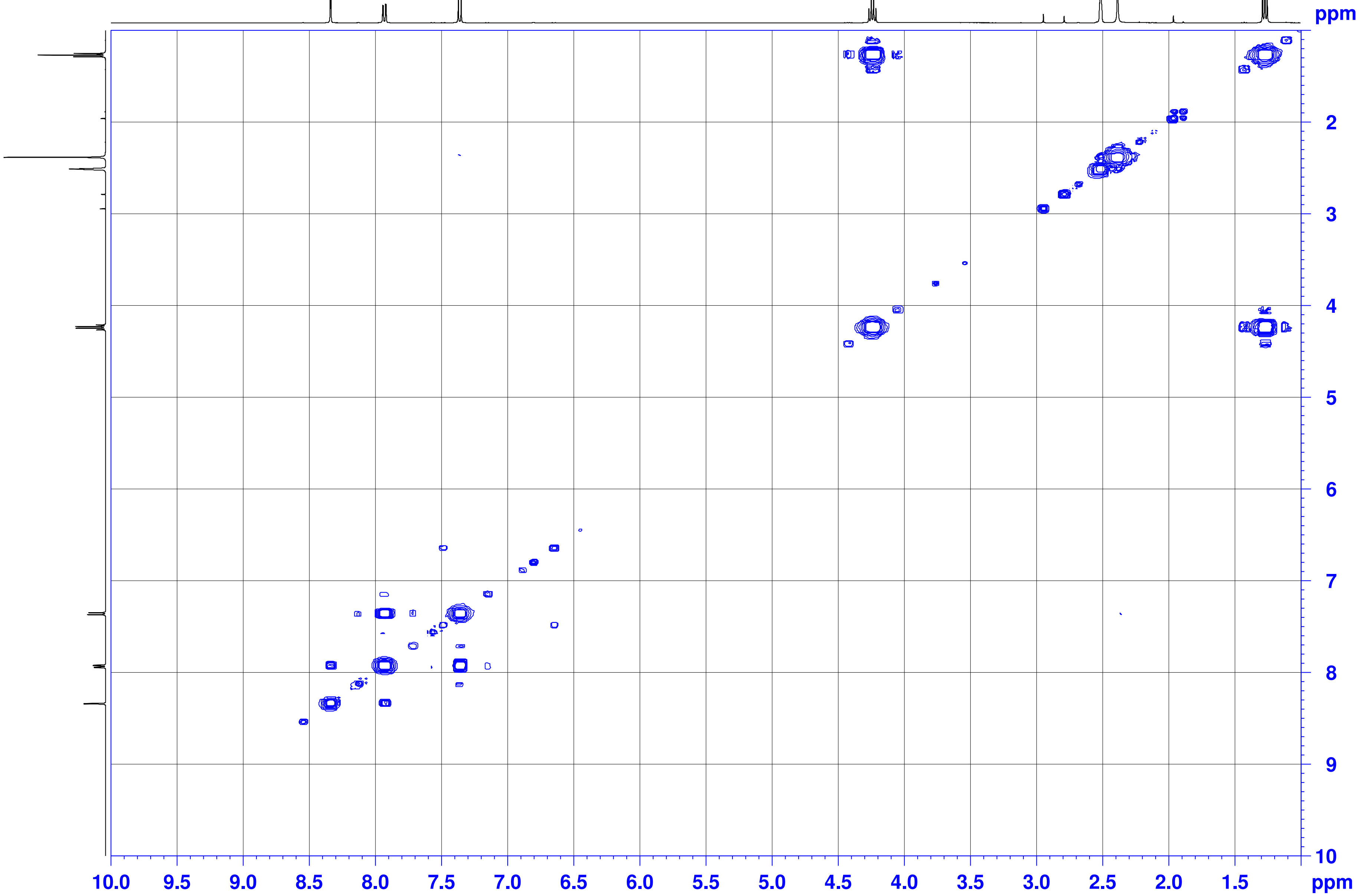

AHP-1-019 (DMSO, 400 MHz)

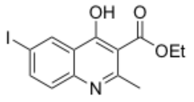

10c

ethyl 4-hydroxy-6-iodo-2-methylquinoline-3-carboxylate

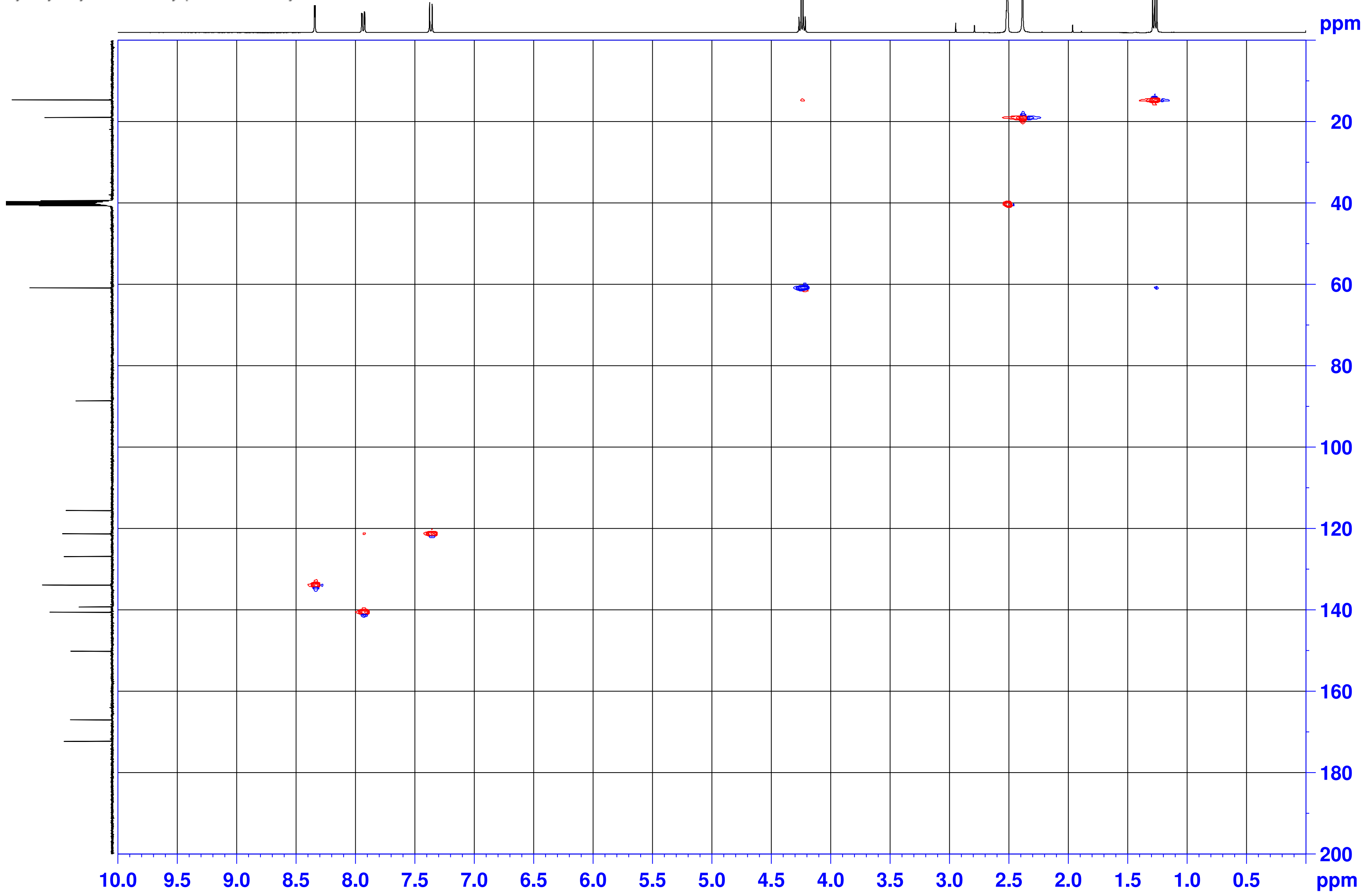

AHP-1-019 (DMSO, 400 MHz)

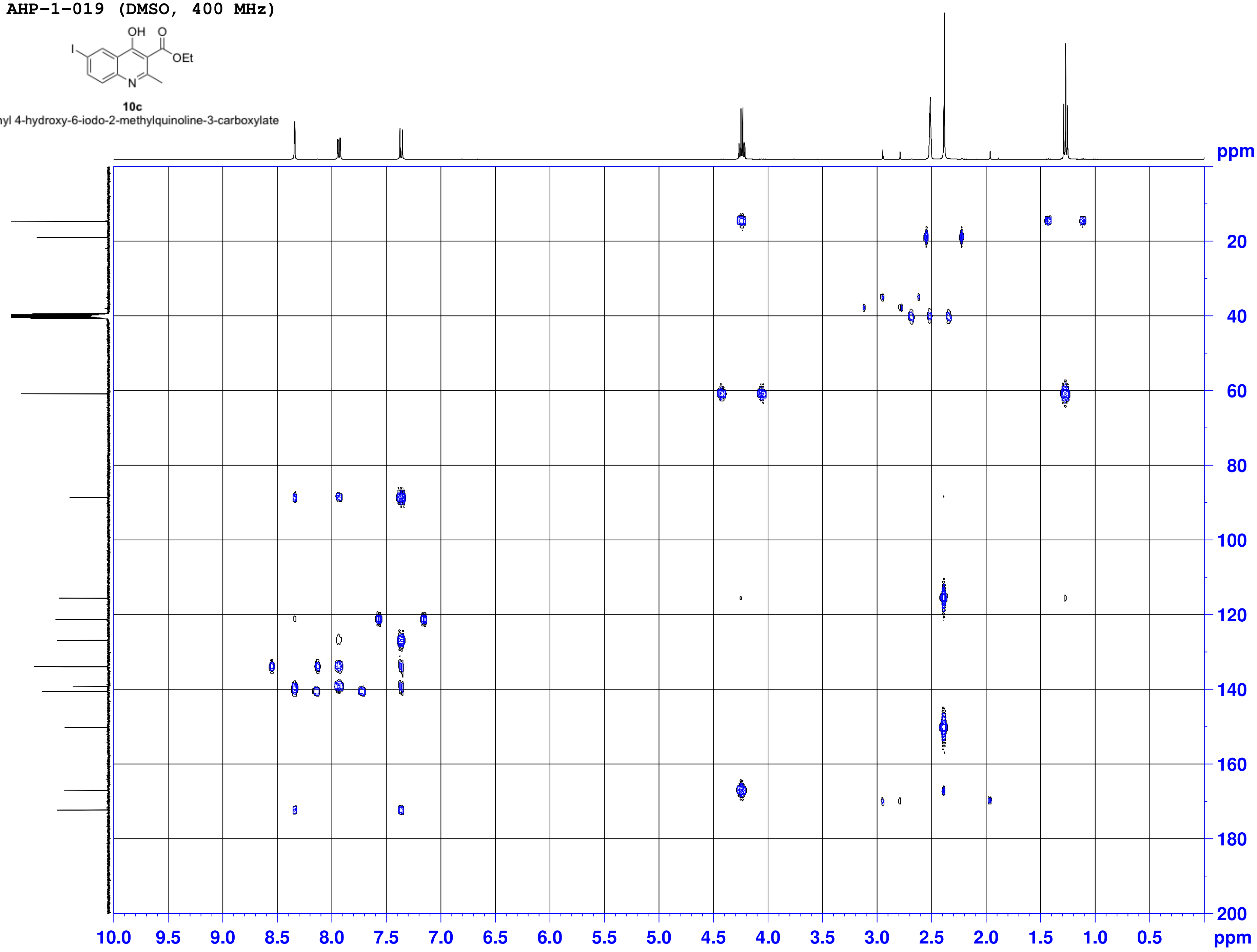

10c

ethyl 4-hydroxy-6-iodo-2-methylquinoline-3-carboxylate

Atlantic Microlab, Inc.

Sample No. AHP-1-019

6180 Atlantic Blvd. Suite M
Norcross, GA 30071
www.atlanticmicrolab.com

Professor/Supervisor: Matthew G. Donahue
PO# / CC#: Visa 5794

Company/School: University of Southern Mississippi
Dept.: Chemistry and Biochemistry
Address: 118 College Dr. #5043
City, State, Zip: Hattiesburg, MS 39406
Name: Matthew G. Donahue
Date: 2/25/17
Phone: 614-203-1123

Single ☒ Duplicate ☐
Elements Present: $C_{13}H_{12}INO_3$
Analyze for: C, H
Hygroscopic ☐ Explosive ☐
M.P. ____ B.P. ____
To be dried: Yes ☐ No ☒
Temp. ____ Vac. ____ Time ____
Rush Service ☐ Rush service guarantees analyses will be completed and results available by 5 PM EST on the day the sample is received by 11 AM.
Include Email Address or FAX # Below
matthew.donahue@usm.edu

Element	Theory	Found	
C	43.72	43.43	
H	3.39	3.29	

Date Received: MAR 13 P.M.
Date Completed: MAR 14 2017
Remarks:

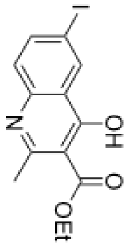

10c
ethyl 4-hydroxy-6-iodo-2-methylquinoline-3-carboxylate

EBC-1-012

Collection time: Fri Jul 28 14:56:59 2017 (GMT-06:00)

Number of sample scans: 8
Number of background scans: 8
Resolution: 2.000
Sample gain: 8.0
Mirror velocity: 0.6329
Aperture: 100.00

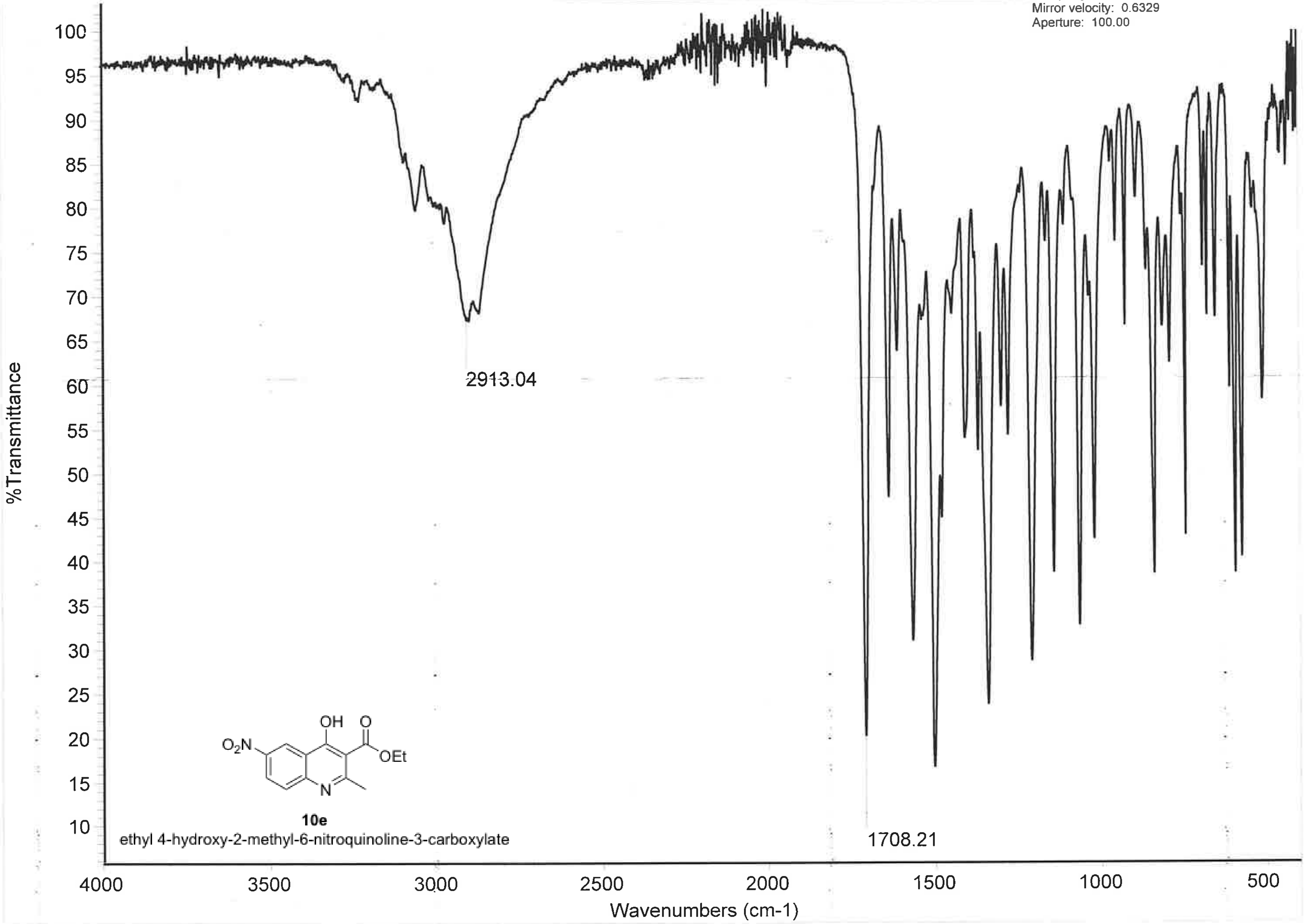

10e

ethyl 4-hydroxy-2-methyl-6-nitroquinoline-3-carboxylate

EBC-1-012 DMSO-d6
pale yellow solid

NAME	EBC-1-012
EXPNO	20
PROCNO	1
Date_	20161212
Time	16.58 h
INSTRUM	spect
PROBHD	Z108618_0161 (
PULPROG	zg30
TD	65536
SOLVENT	DMSO
NS	16
DS	2
SWH	8012.820 Hz
FIDRES	0.244532 Hz
AQ	4.0894966 sec
RG	144
DW	62.400 usec
DE	6.50 usec
TE	297.3 K
D1	1.00000000 sec
TD0	1
SFO1	400.1724710 MHz
NUC1	1H
P1	29.07 usec
SI	65536
SF	400.1699988 MHz
WDW	EM
SSB	0
LB	0.30 Hz
GB	0
PC	1.00

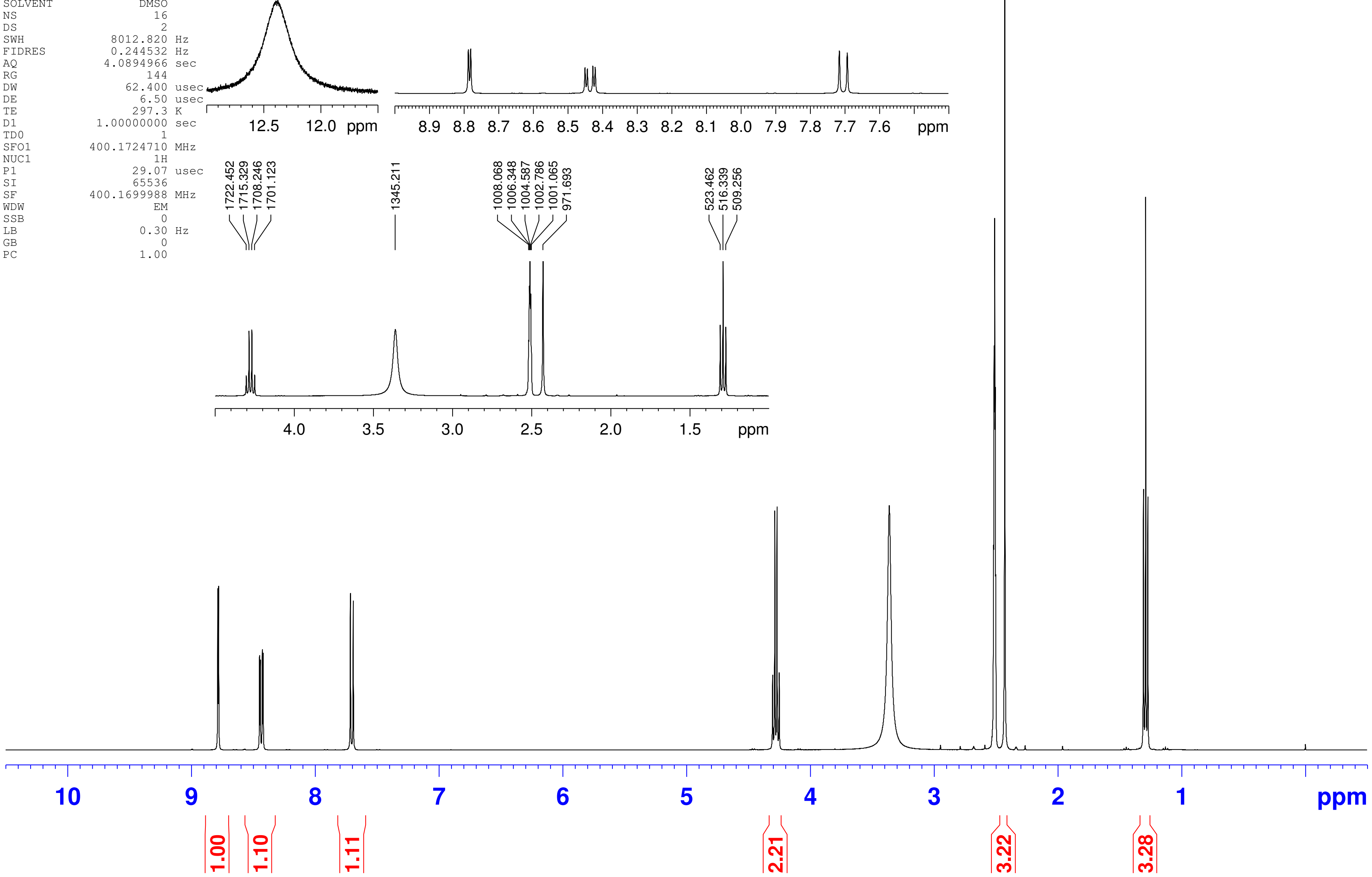

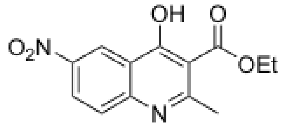

10e

ethyl 4-hydroxy-2-methyl-6-nitroquinoline-3-carboxylate

EBC-1-012 DMSO-d6
old sample
pale yellow solid

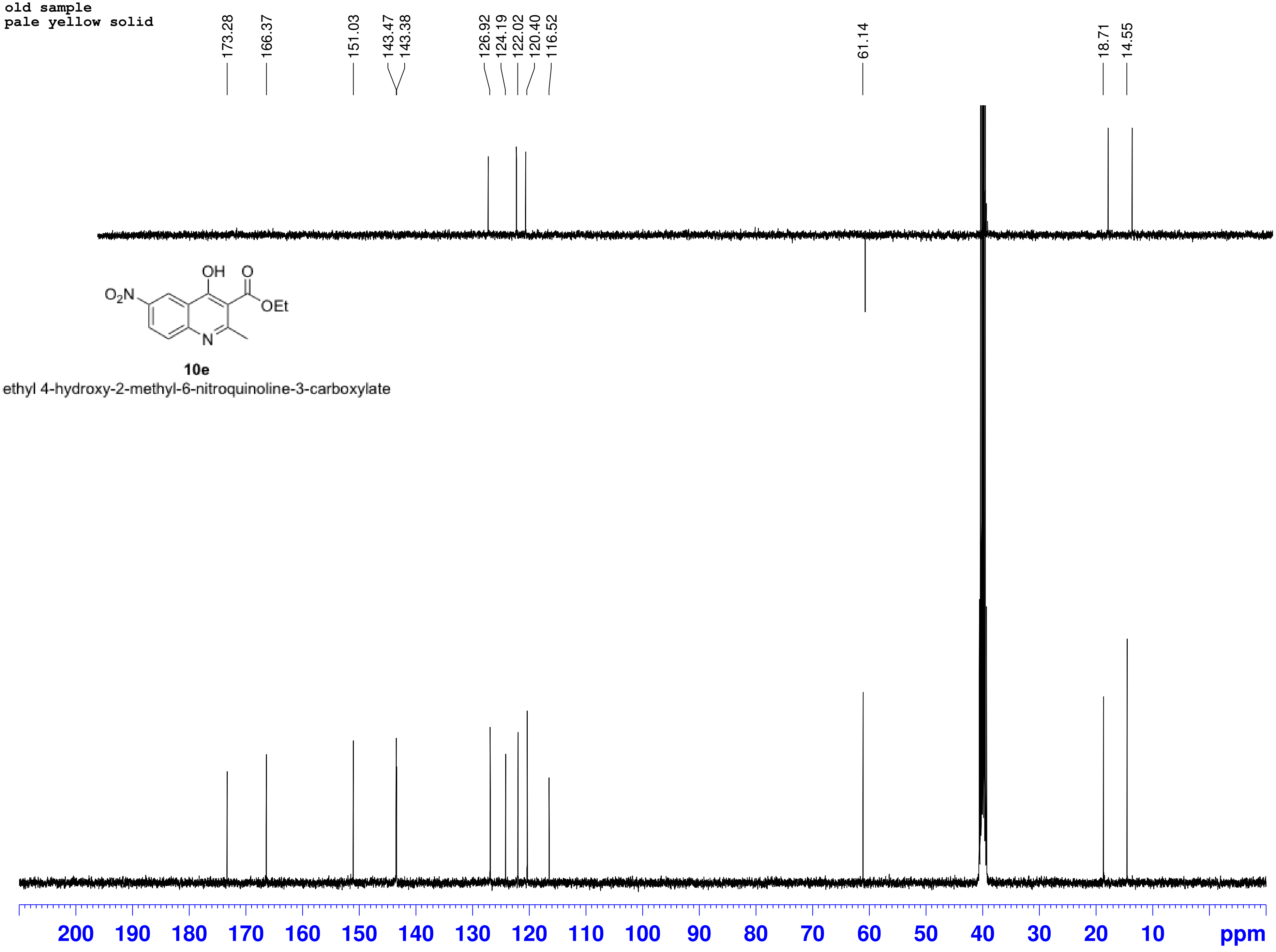

10e
ethyl 4-hydroxy-2-methyl-6-nitroquinoline-3-carboxylate

EBC-1-012 (DMSO, 400 MHz)

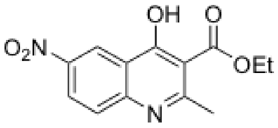

10e

ethyl 4-hydroxy-2-methyl-6-nitroquinoline-3-carboxylate

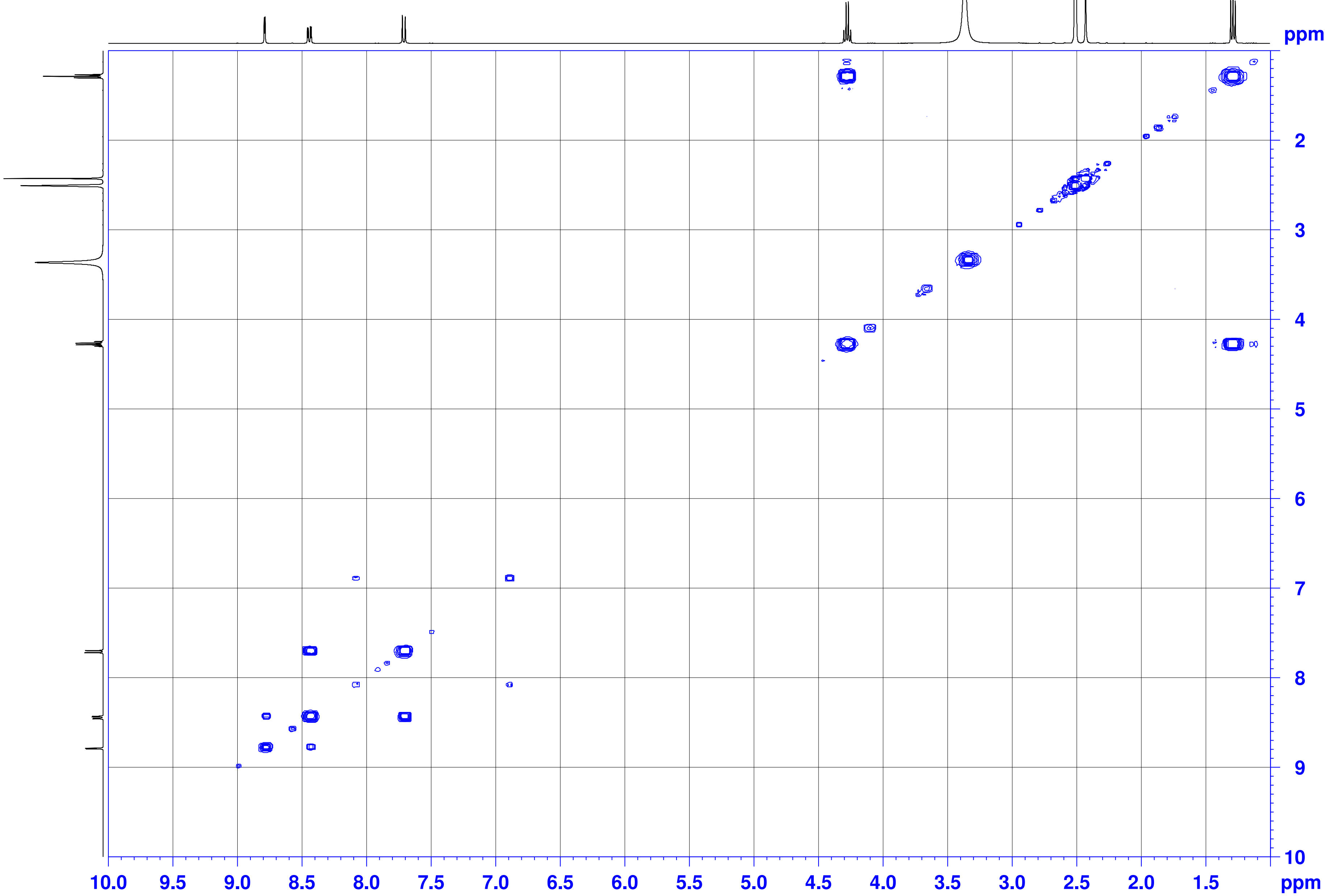

EBC-1-012 (DMSO, 400 MHz)

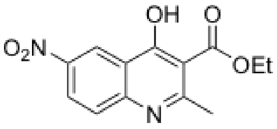

10e

ethyl 4-hydroxy-2-methyl-6-nitroquinoline-3-carboxylate

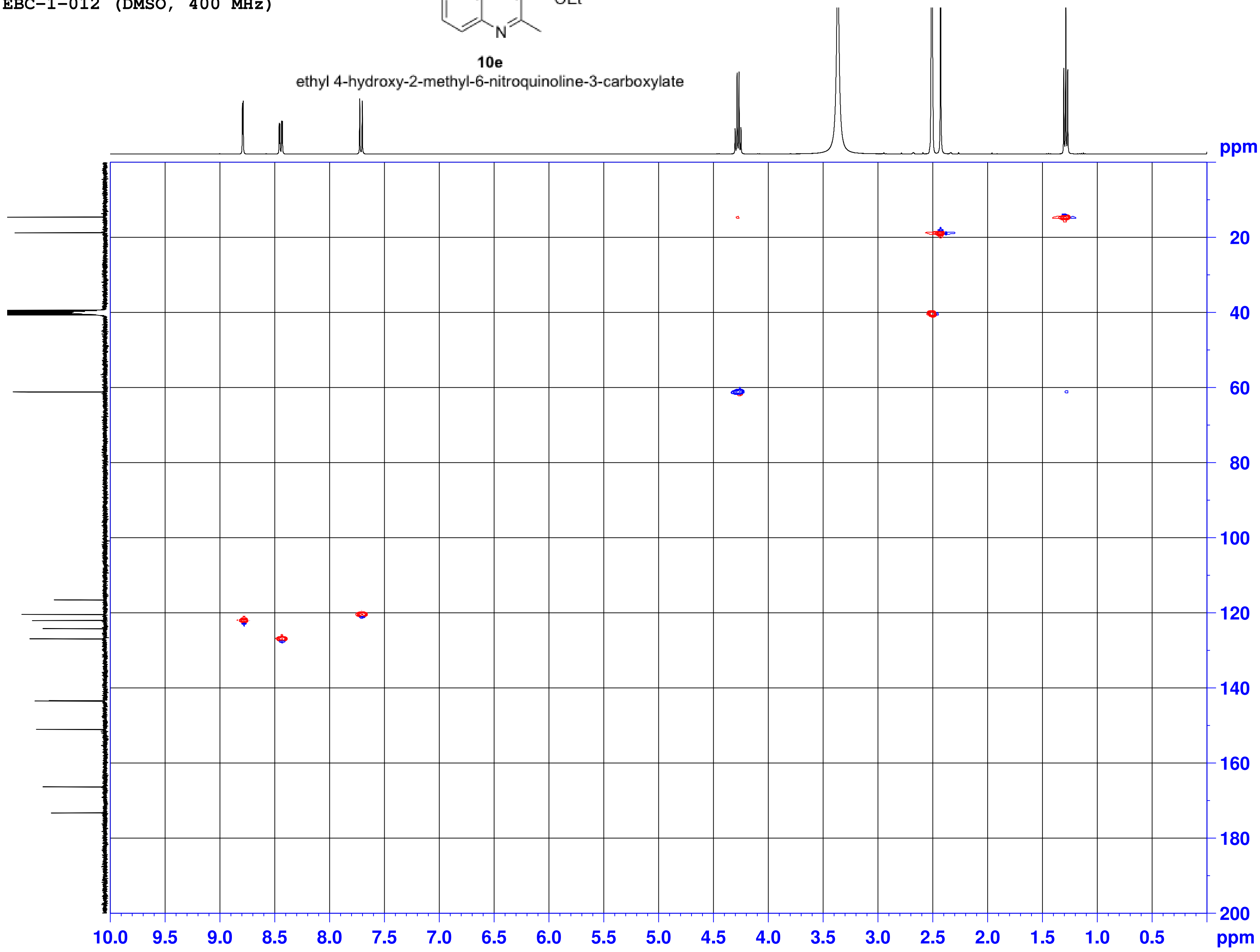

EBC-1-015 DMSO-d6
Red-orange solid

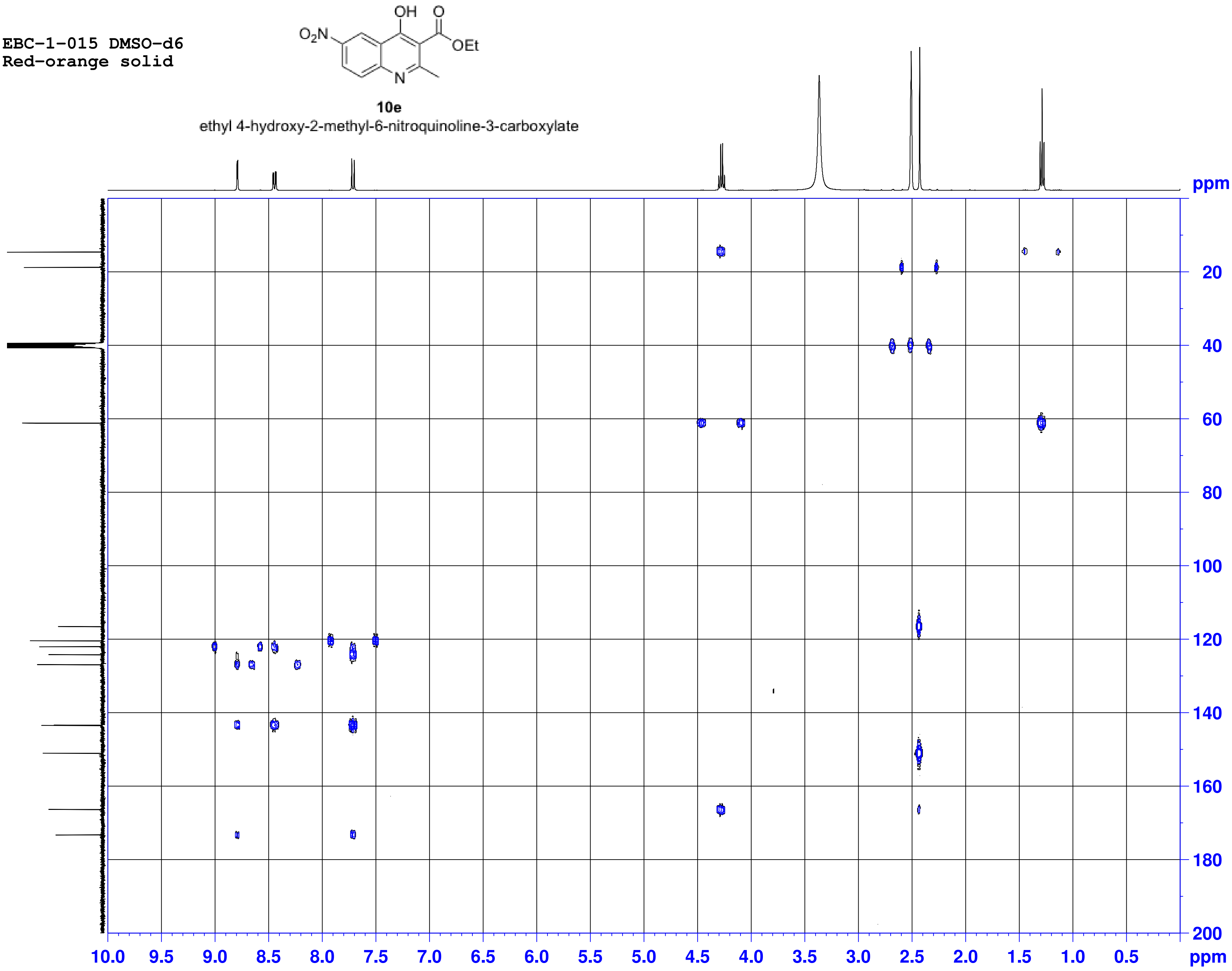

10e
ethyl 4-hydroxy-2-methyl-6-nitroquinoline-3-carboxylate

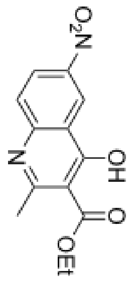

10e

ethyl 4-hydroxy-2-methyl-6-nitroquinoline-3-carboxylate

Atlantic Microlab, Inc.

Sample No. EBC-1-012

6180 Atlantic Blvd. Suite M
Norcross, GA 30071
www.atlanticmicrolab.com

Professor/Supervisor: Matthew G. Donahue

PO# / CC# Visa 5794

Company/School University of Southern Mississippi
Dept. Chemistry and Biochemistry
Address 118 College Dr. #5043
City, State, Zip Hattiesburg, MS 39406
Name Matthew G. Donahue Date 2/25/17
Phone 614-203-1123

Element	Theory	Found	
C	56.52	56.26	
H	4.38	4.43	

Single ☒ Duplicate ☐

Elements Present: $C_{13}H_{12}N_2O_5$

Analyze for: C, H

Hygroscopic ☐ Explosive ☐
M.P. ____ B.P. ____

To be dried: Yes ☐ No ☒
Temp. ____ Vac. ____ Time ____

Rush Service ☐ Rush service guarantees analyses will be completed and results available by 5 PM EST on the day the sample is received by 11 AM.

Include Email Address or FAX # Below

matthew.donahue@usm.edu

Date Received MAR 13 P.M. Date Completed MAR 14 2017

Remarks:

MEW-1-004

Collection time: Fri Jul 28 15:01:21 2017 (GMT-06:00)

Number of sample scans: 8
Number of background scans: 8
Resolution: 2.000
Sample gain: 8.0
Mirror velocity: 0.6329
Aperture: 100.00

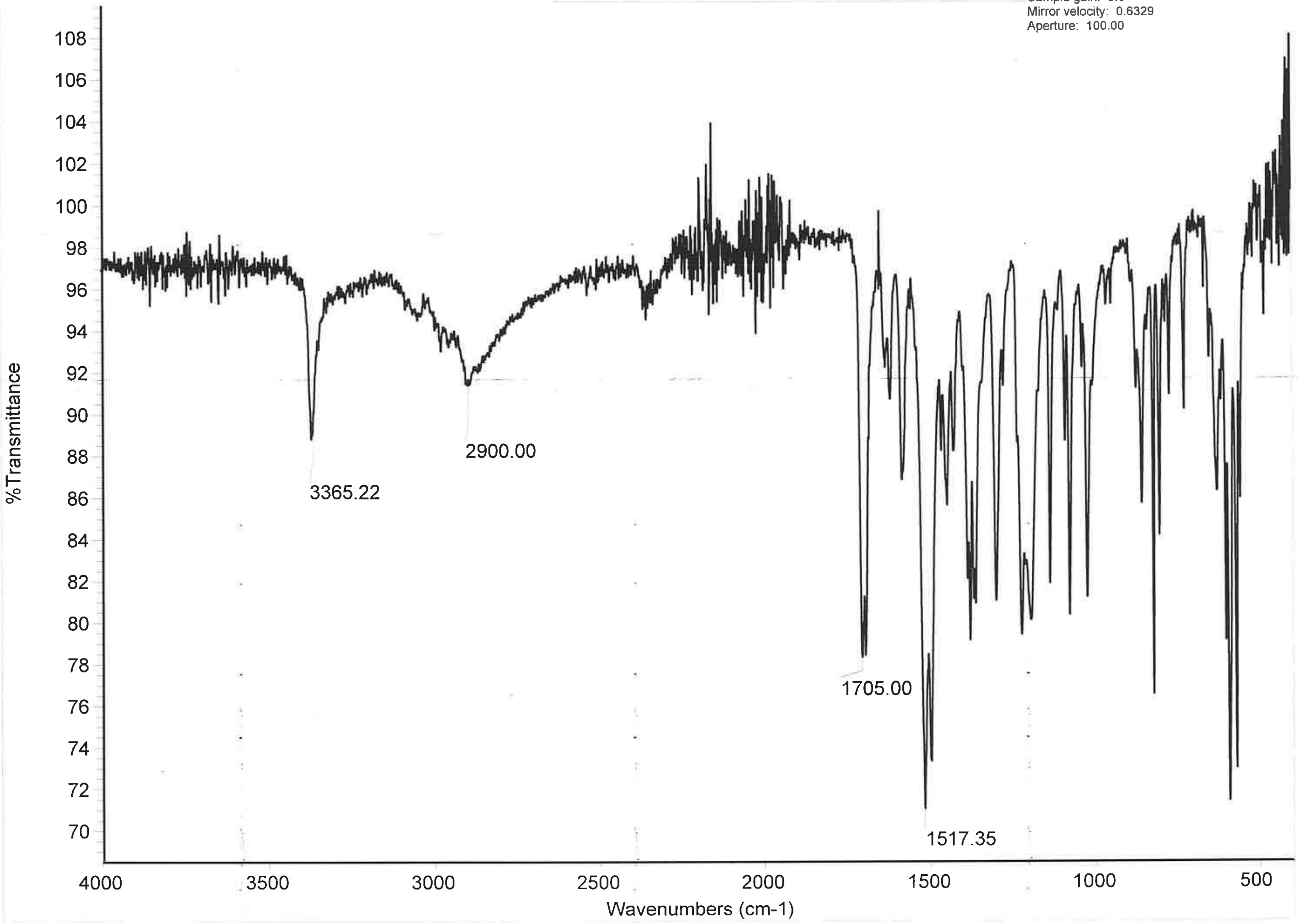

MEW-1-004 (DMSO, 400 MHz) White Solid- synthesis of 4,6-dihydroxyquinoline

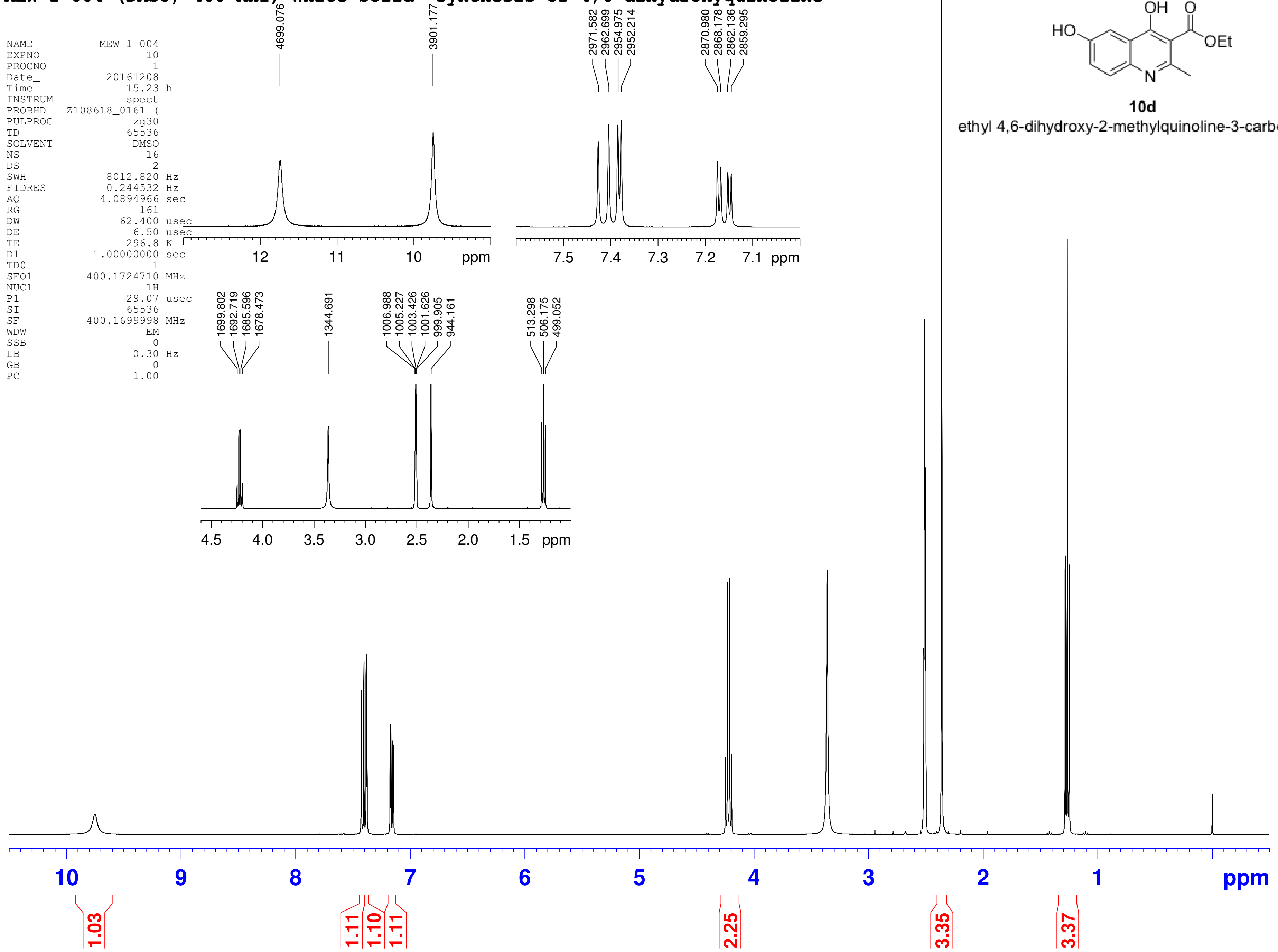

10d

ethyl 4,6-dihydroxy-2-methylquinoline-3-carboxylate

MEW-1-004 (DMSO, 400 MHz) Pure White Solid - dihydroxy quinoli

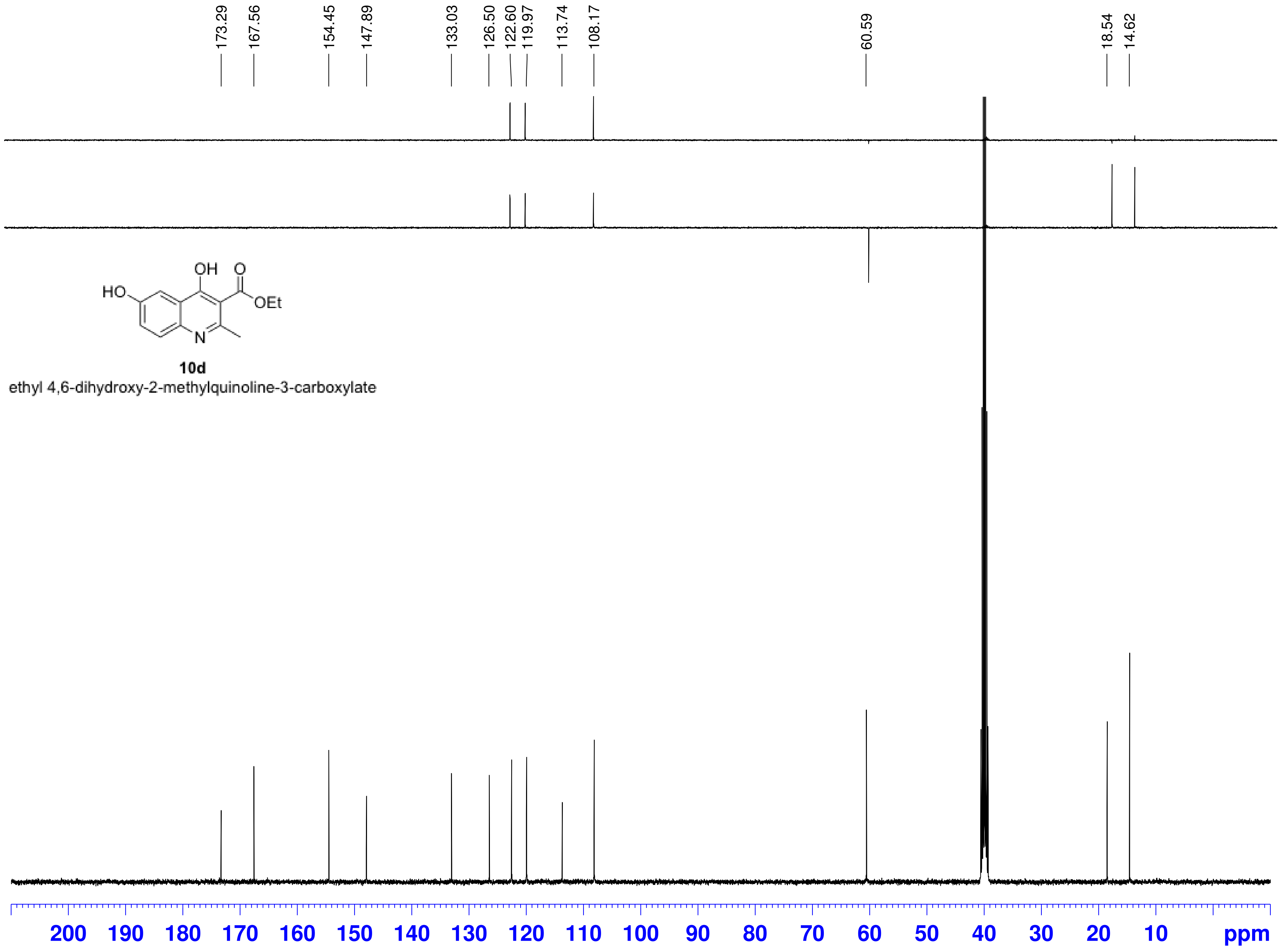

10d
ethyl 4,6-dihydroxy-2-methylquinoline-3-carboxylate

MEW-1-004 (DMSO, 400 MHz)

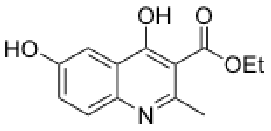

10d
ethyl 4,6-dihydroxy-2-methylquinoline-3-carboxylate

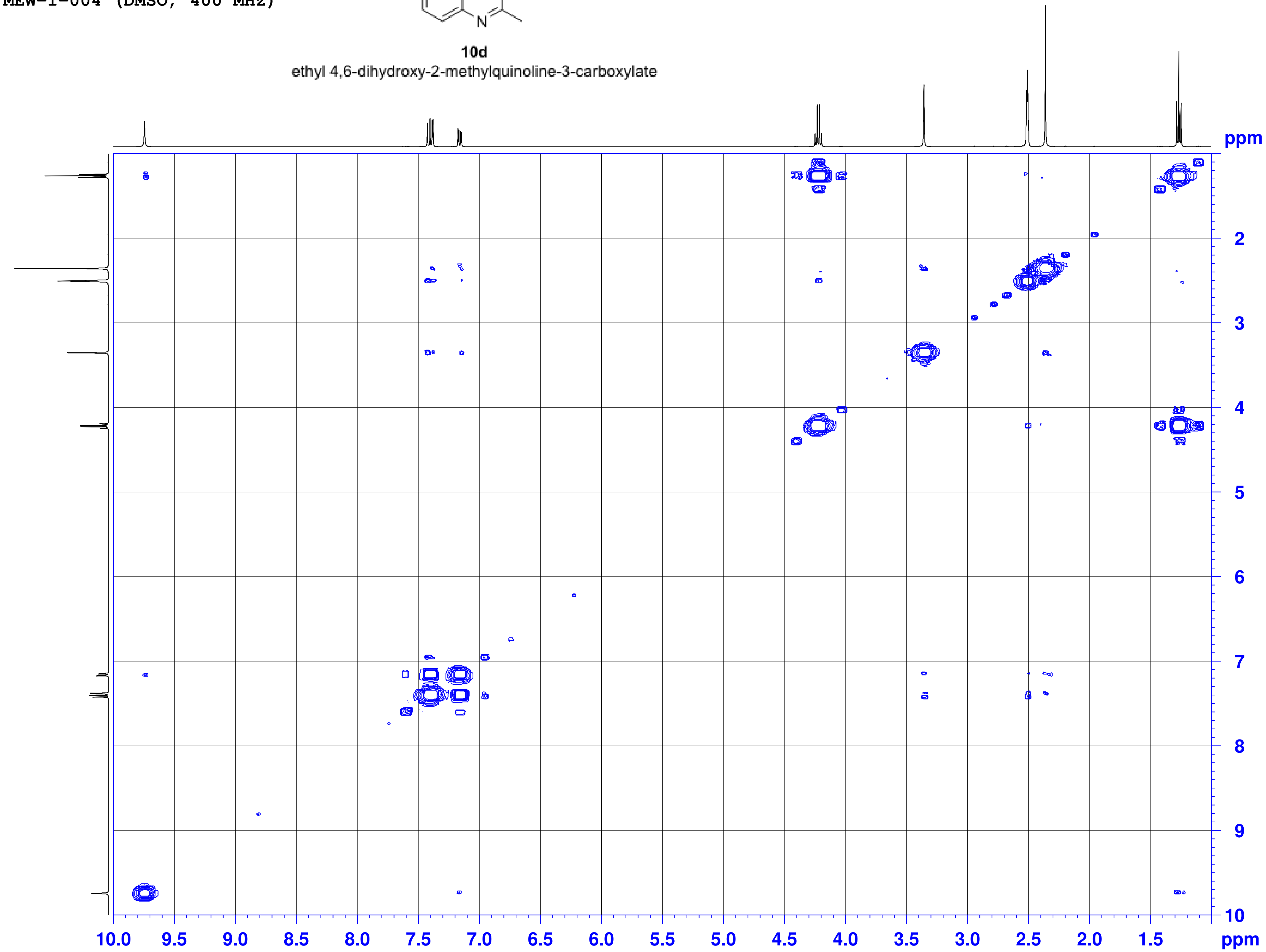

MEW-1-004 (DMSO, 400 MHz)

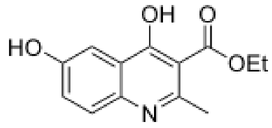

10d
ethyl 4,6-dihydroxy-2-methylquinoline-3-carboxylate

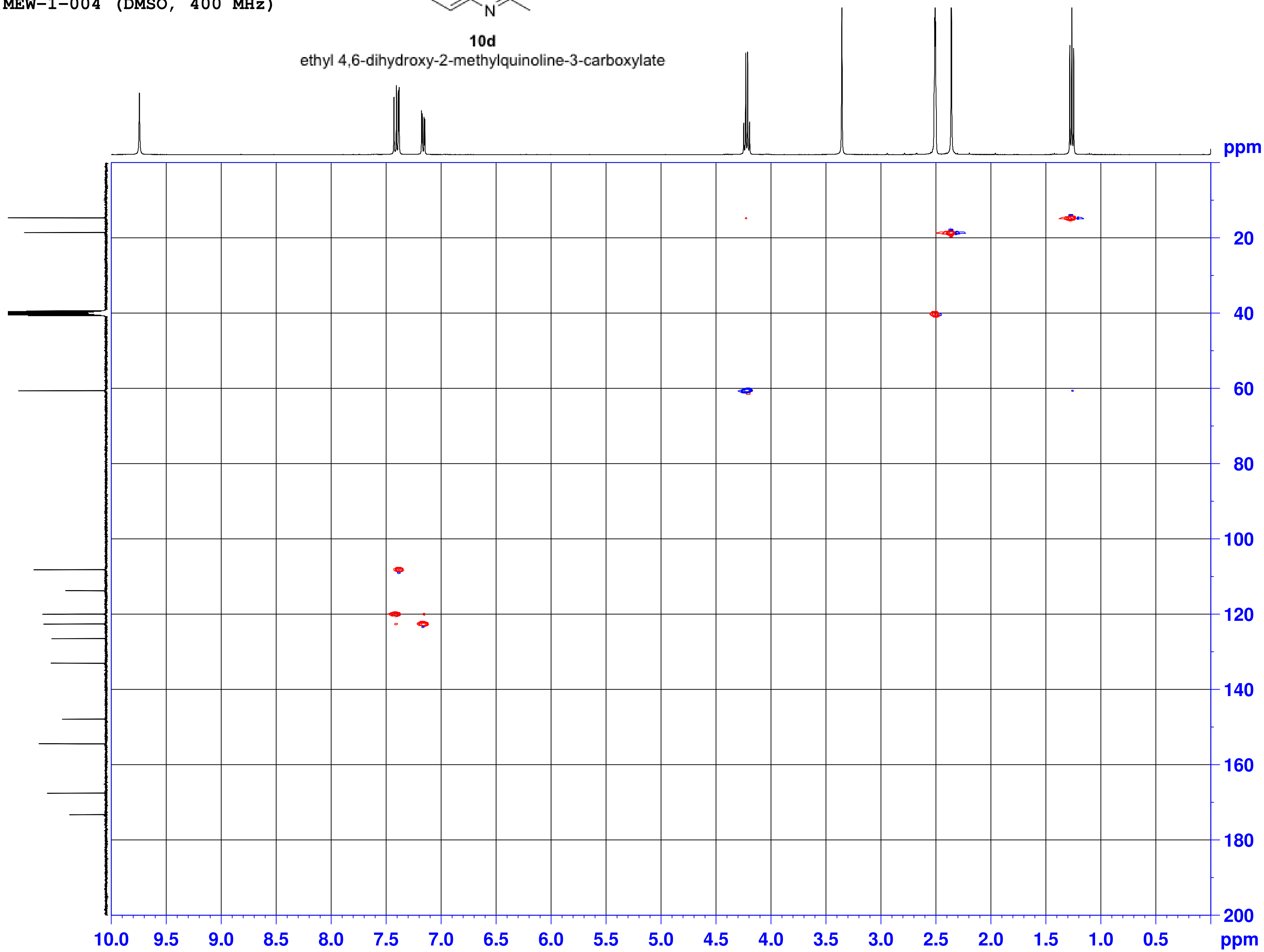

MEW-1-004 (DMSO, 400 MHz)

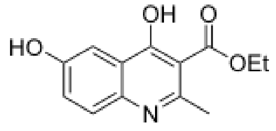

10d

ethyl 4,6-dihydroxy-2-methylquinoline-3-carboxylate

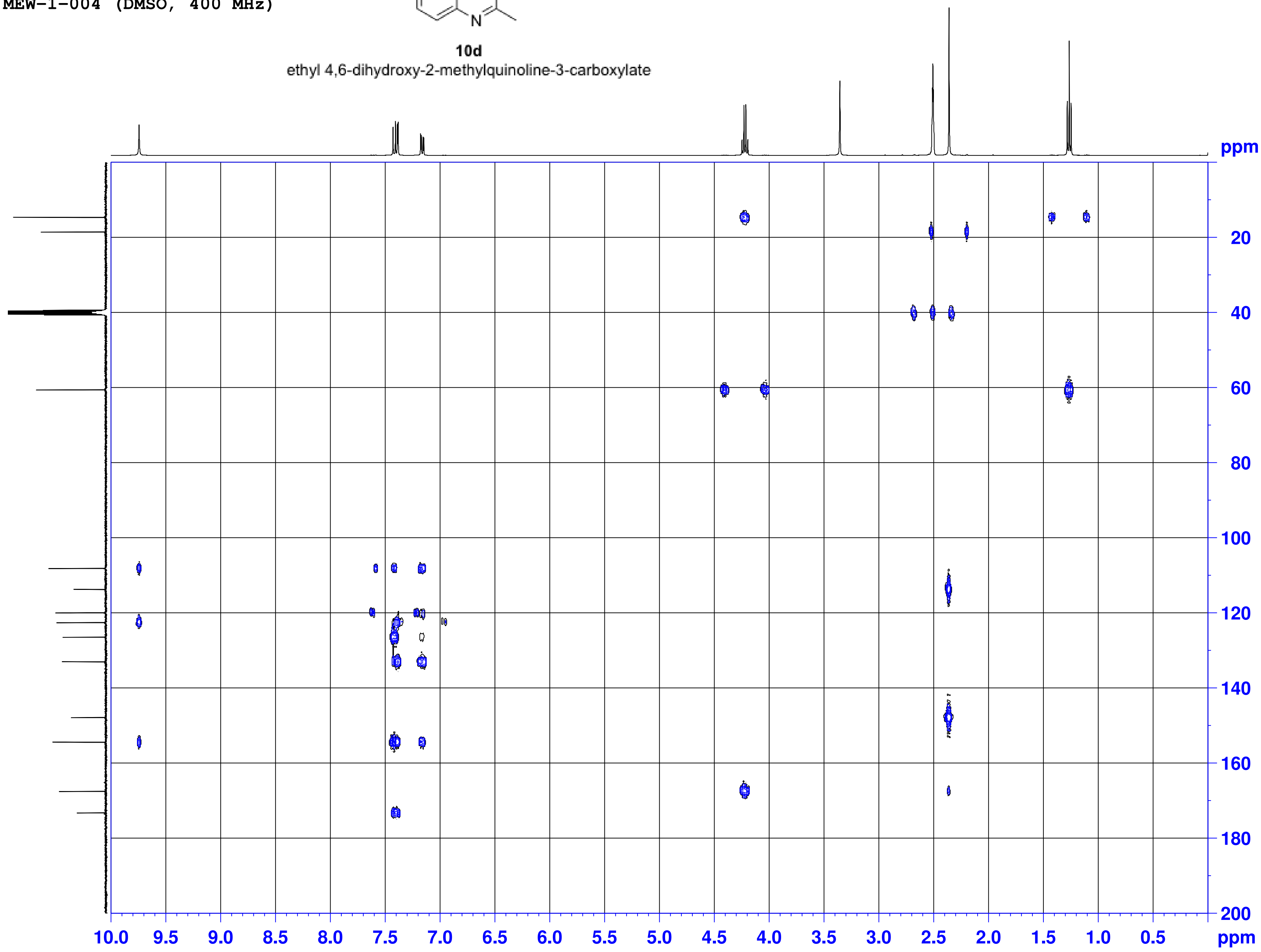

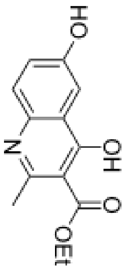

10d

ethyl 4,6-dihydroxy-2-methylquinoline-3-carboxylate

Atlantic Microlab, Inc.

Sample No. MEW-1-009

6180 Atlantic Blvd. Suite M
Norcross, GA 30071
www.atlanticmicrolab.com

Professor/Supervisor: Matthew G. Donahue
PO# / CC# Visa 5794

Company/School University of Southern Mississippi
Dept. Chemistry and Biochemistry
Address 118 College Dr. #5043
City, State, Zip Hattiesburg, MS 39406
Name Matthew G. Donahue Date 2/25/17
Phone 614-203-1123

Element	Theory	Found	
C	63.15	62.89	
H	5.30	5.44	

Single ☒ Duplicate ☐
Elements Present: $C_{13}H_{13}NO_4$
Analyze for: C, H
Hygroscopic ☐ Explosive ☐
M.P. ______ B.P. ______
To be dried: Yes ☐ No ☒
Temp. ______ Vac. ______ Time ______
Rush Service ☐ Rush service guarantees analyses will be completed and results available by 5 PM EST on the day the sample is received by 11 AM.
Include Email Address or FAX # Below
matthew.donahue@usm.edu

Date Received MAR 13 P.M. Date Completed MAR 14 2017
Remarks:

*EBC-1-013

Collection time: Thu Mar 16 12:33:49 2017 (GMT-06:00)

Number of sample scans: 8
Number of background scans: 8
Resolution: 2.000
Sample gain: 8.0
Mirror velocity: 0.6329
Aperture: 100.00

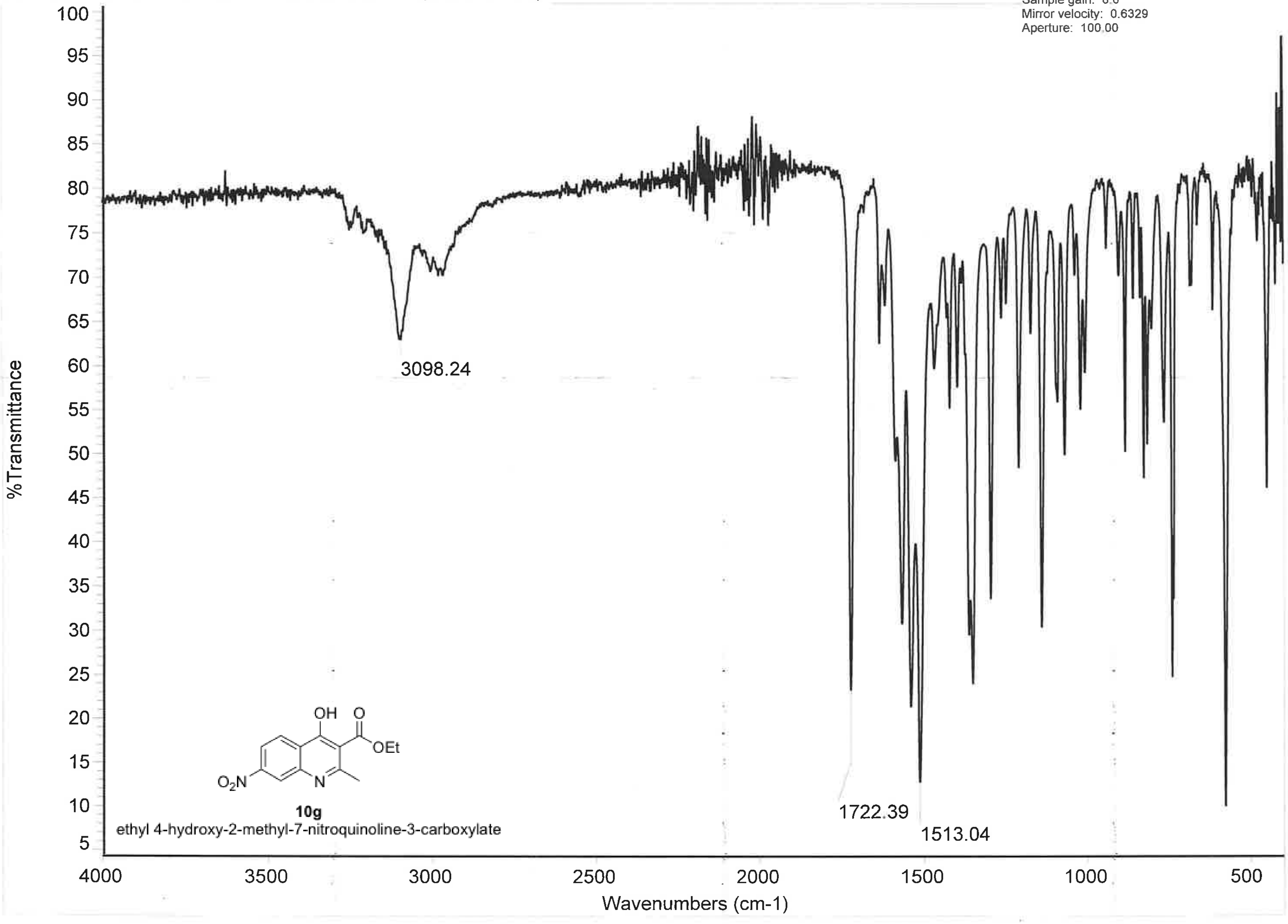

EBC-1-013 DMSO-D6
yellow solid

NAME	EBC-1-013	
EXPNO	20	
PROCNO	1	
Date_	20170228	
Time	17.03	h
INSTRUM	spect	
PROBHD	Z108618_0161 (	
PULPROG	zg30	
TD	65536	
SOLVENT	DMSO	
NS	16	
DS	2	
SWH	8012.820	Hz
FIDRES	0.244532	Hz
AQ	4.0894966	sec
RG	203	
DW	62.400	usec
DE	6.50	usec
TE	297.6	K
D1	1.00000000	sec
TD0	1	
SFO1	400.1724710	MHz
NUC1	1H	
P1	9.88	usec
SI	65536	
SF	400.1700000	MHz
WDW	EM	
SSB	0	
LB	0.30	Hz
GB	0	
PC	1.00	

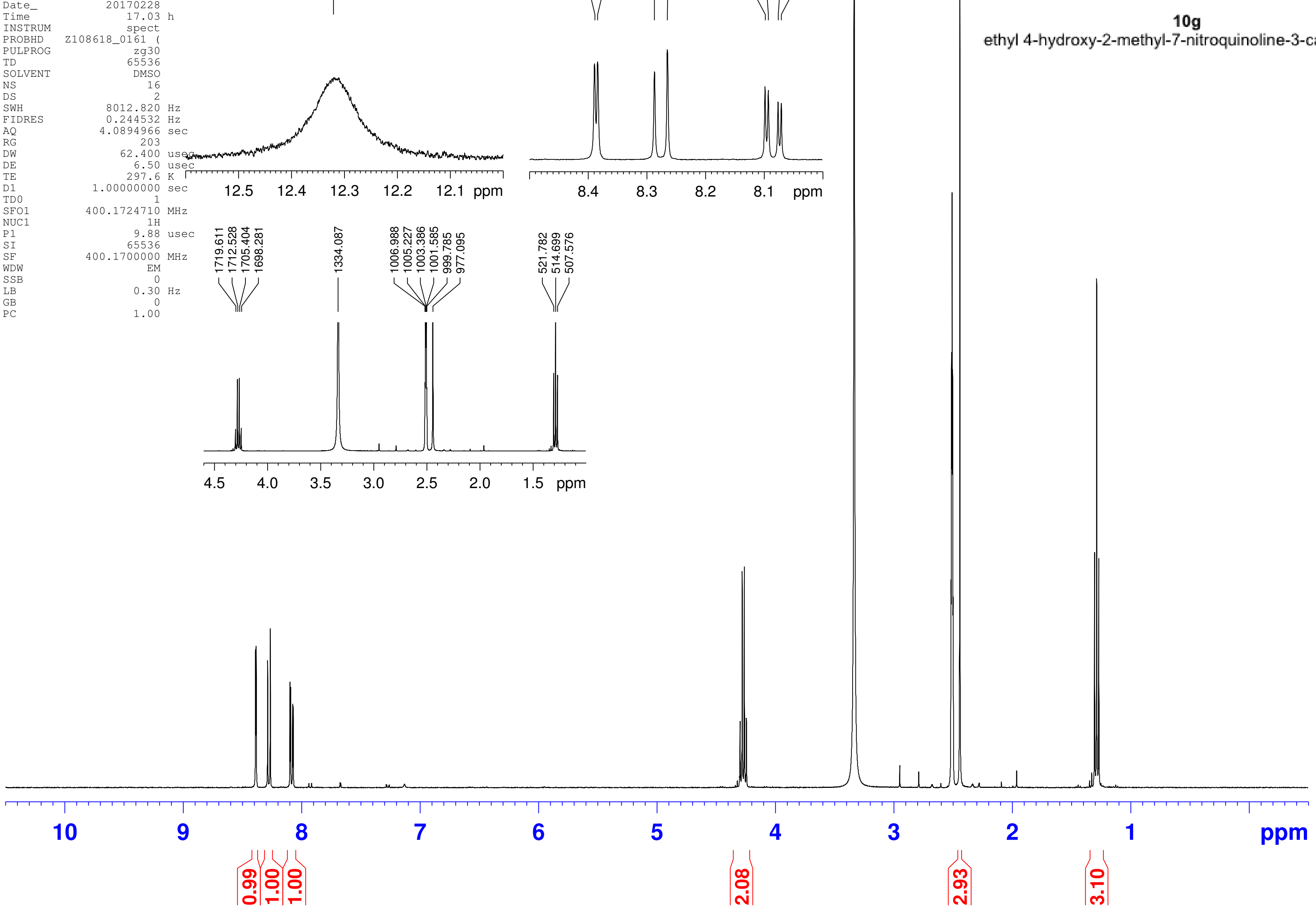

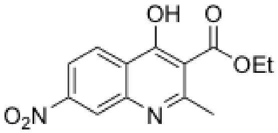

10g

ethyl 4-hydroxy-2-methyl-7-nitroquinoline-3-carboxylate

EBC-1-013 DMSO-D6
yellow solid

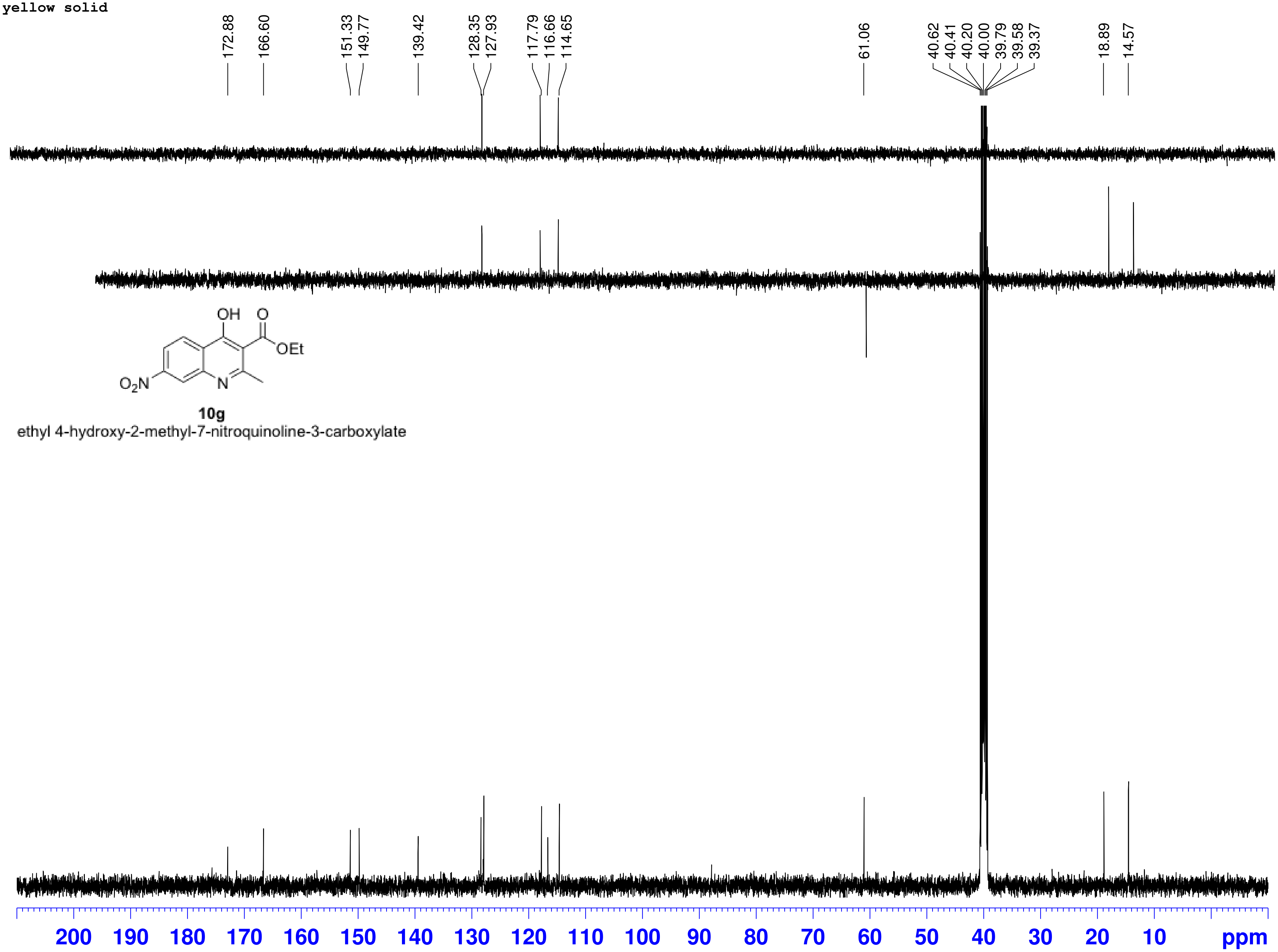

10g

ethyl 4-hydroxy-2-methyl-7-nitroquinoline-3-carboxylate

EBC-1-013 DMSO-D6
yellow solid

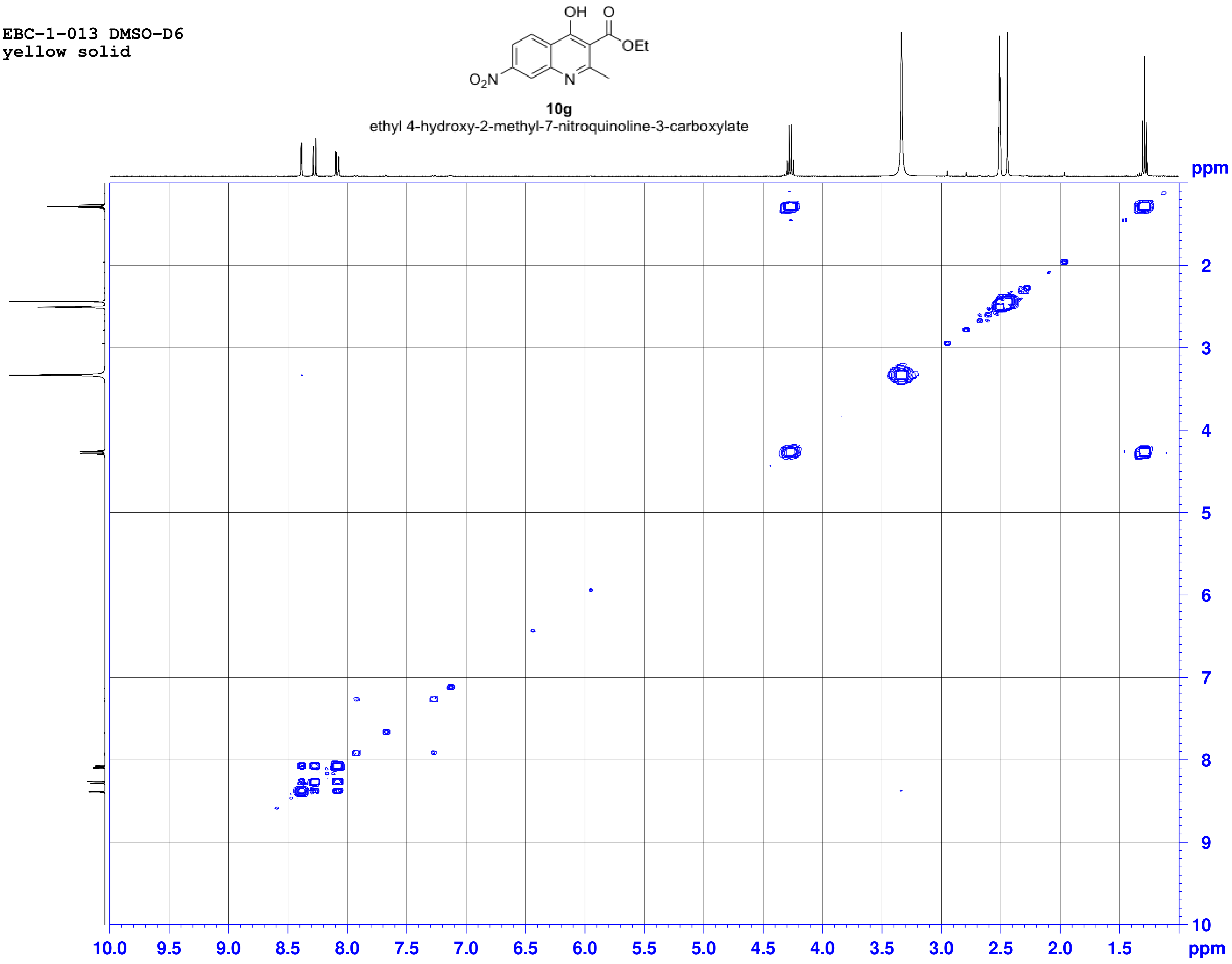

EBC-1-013 DMSO-D6
yellow solid

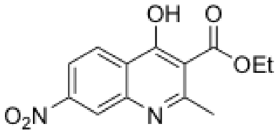

10g

ethyl 4-hydroxy-2-methyl-7-nitroquinoline-3-carboxylate

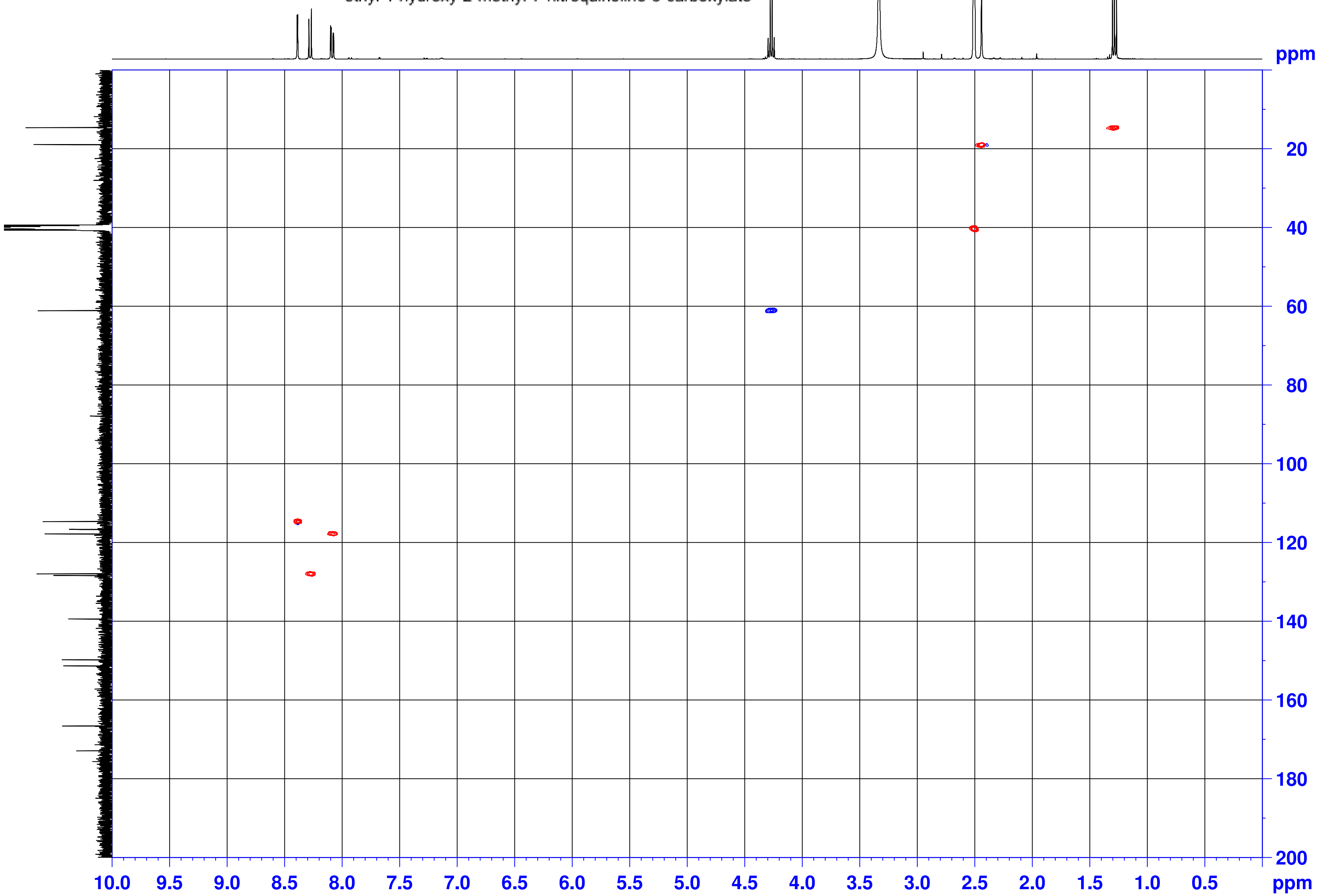

EBC-1-013 DMSO-D6
yellow solid

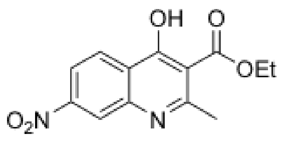

10g

ethyl 4-hydroxy-2-methyl-7-nitroquinoline-3-carboxylate

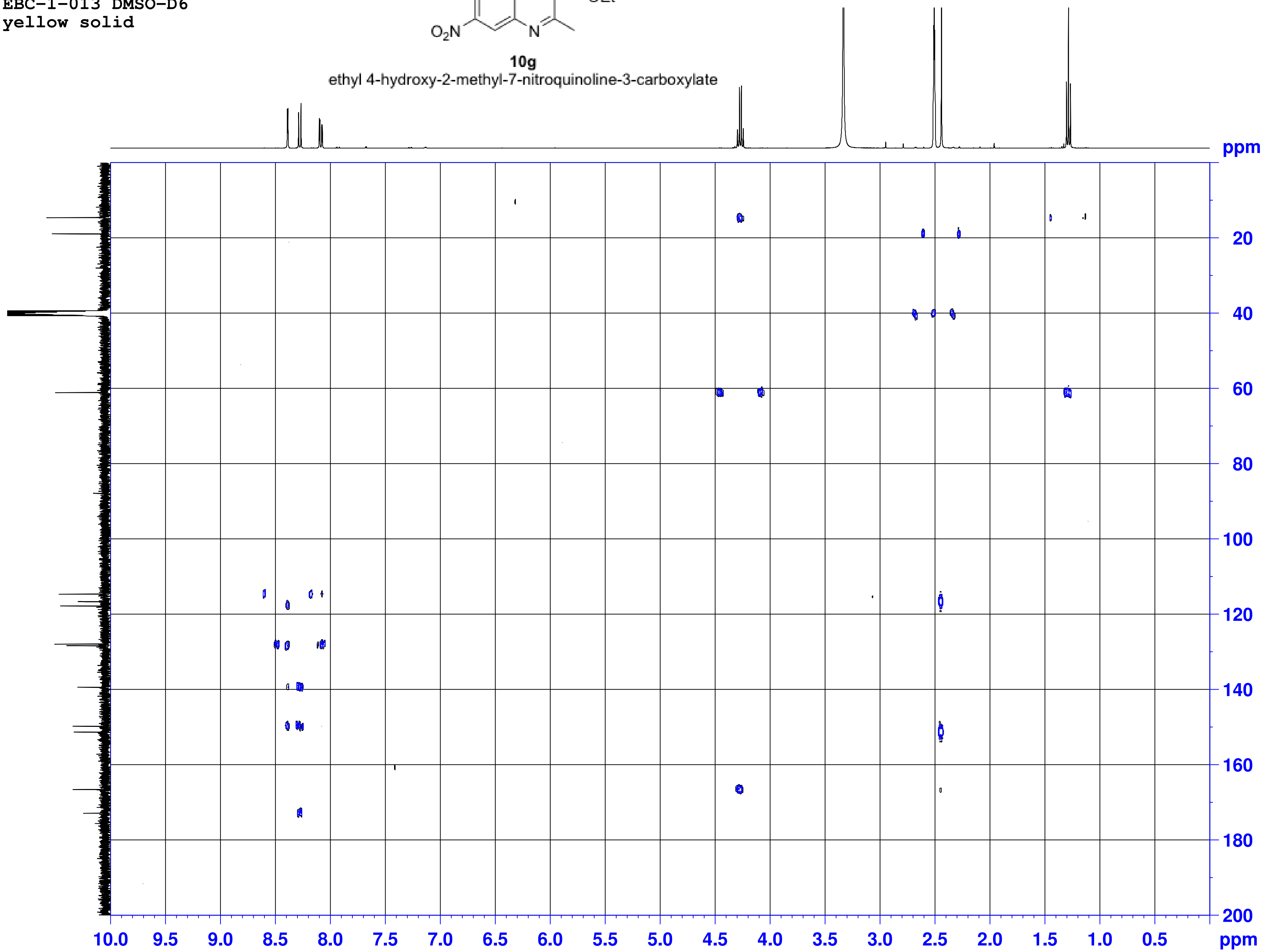

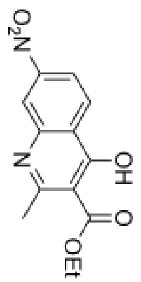

10g
ethyl 4-hydroxy-2-methyl-7-nitroquinoline-3-carboxylate

Atlantic Microlab, Inc.

Sample No. EBC-1-013

6180 Atlantic Blvd. Suite M
Norcross, GA 30071
www.atlanticmicrolab.com

Company/School University of Southern Mississippi
Dept. Chemistry and Biochemistry
Address 118 College Dr. #5043
City, State, Zip Hattiesburg, MS 39406
Name Matthew G. Donahue Date 2/23/17
Phone 614-203-1123

Professor/Supervisor: Matthew G. Donahue
PO# / CC#. Visa 5794

Element	Theory	Found	
C	56.52	56.23	
H	4.38	4.40	

Single ☒ Duplicate ☐
Elements Present: $C_{13}H_{12}N_2O_5$
Analyze for: C, H
Hygroscopic ☐ Explosive ☐
M.P. ______ B.P. ______
To be dried: Yes ☐ No ☒
Temp. ______ Vac. ______ Time ______
Rush Service ☐ Rush service guarantees analyses will be completed and results available by 5 PM EST on the day the sample is received by 11 AM.
Include Email Address or FAX # Below
matthew.donahue@usm.edu

Date Received MAR 13 P.M. Date Completed MAR 14 2017
Remarks:

NGJ-8-030 (DMSO, 400 MHz) Pure White Solid - 7-bromoquinoline synthesis

NAME	NGJ-8-030	
EXPNO	40	
PROCNO	1	
Date_	20170823	
Time	17.45	h
INSTRUM	spect	
PROBHD	Z108618_0161 (	
PULPROG	zg30	
TD	65536	
SOLVENT	DMSO	
NS	16	
DS	2	
SWH	8012.820	Hz
FIDRES	0.244532	Hz
AQ	4.0894966	sec
RG	161	
DW	62.400	usec
DE	6.50	usec
TE	297.7	K
D1	1.00000000	sec
TD0	1	
SFO1	400.1724710	MHz
NUC1	1H	
P1	9.88	usec
SI	65536	
SF	400.1699989	MHz
WDW	EM	
SSB	0	
LB	0.30	Hz
GB	0	
PC	1.00	

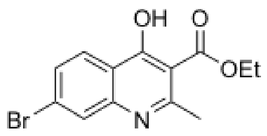

10f

ethyl 7-bromo-4-hydroxy-2-methylquinoline-3-carboxylate

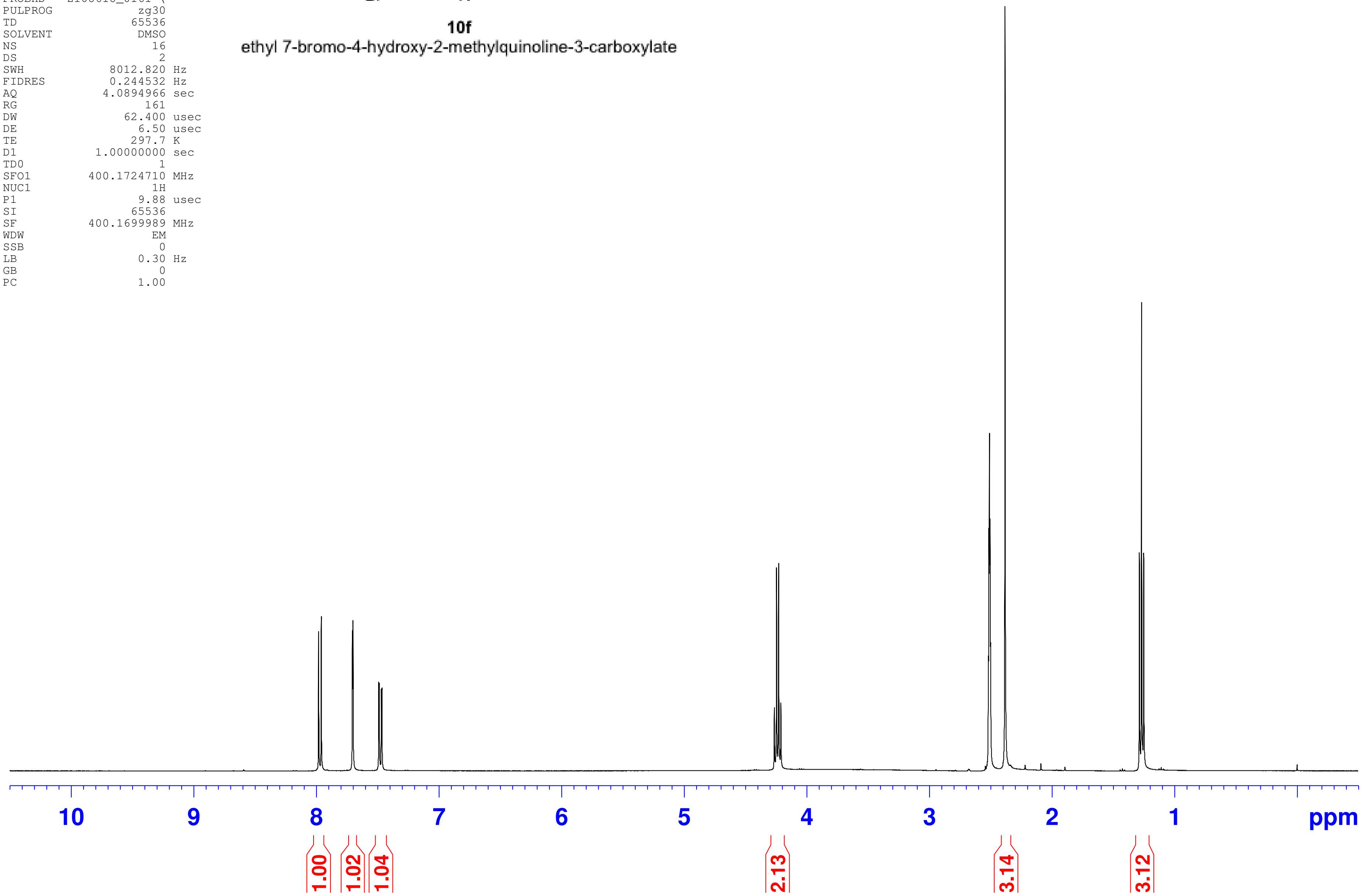

NGJ-8-030 (DMSO, 400 MHz) Pure White Solid - 7-bromoquinoline

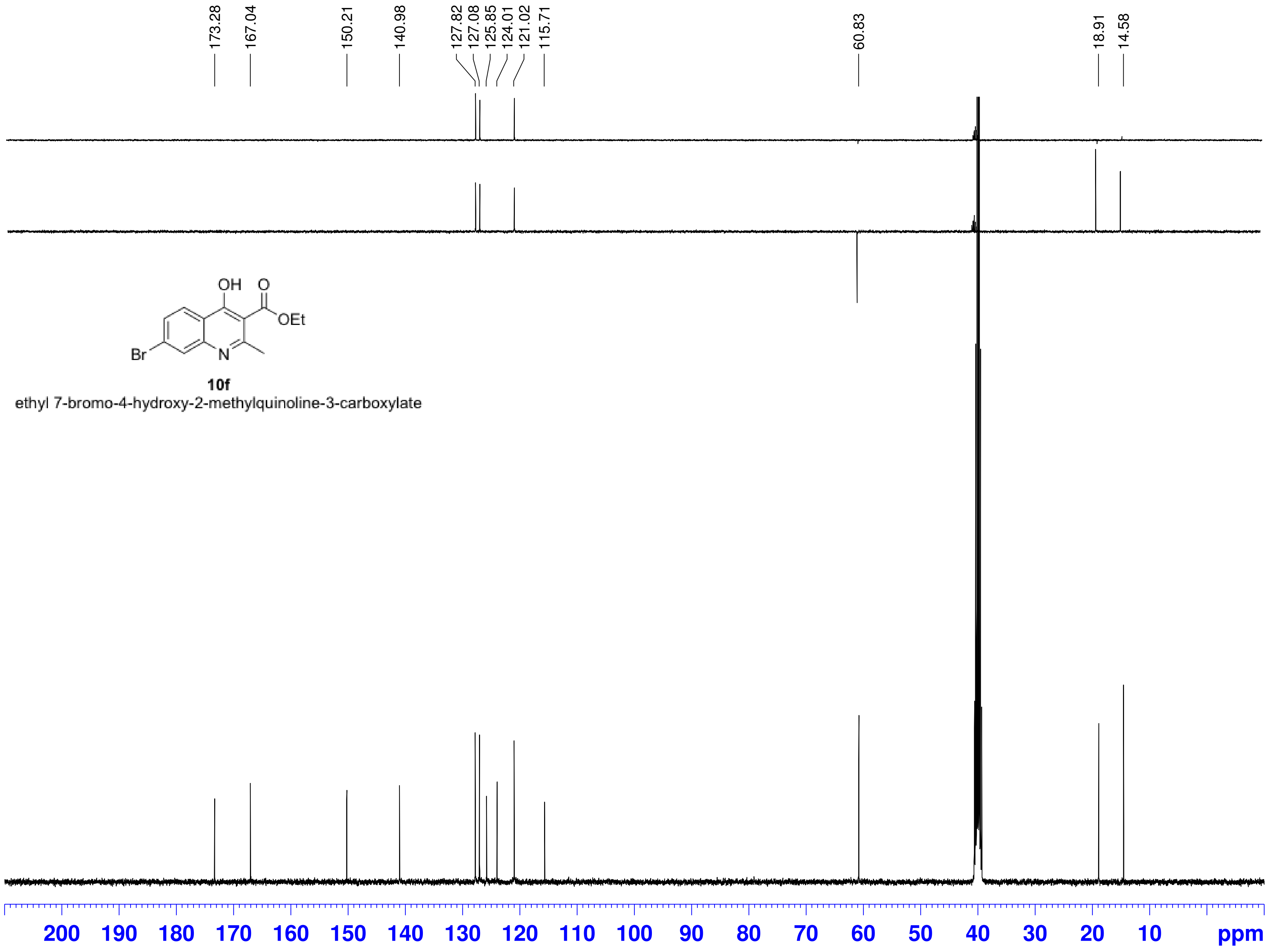

10f
ethyl 7-bromo-4-hydroxy-2-methylquinoline-3-carboxylate

NGJ-8-030 (DMSO, 400 MHz) Pure White Solid - 7-bromoquinoline synthesis

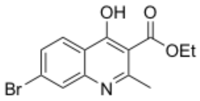

10f

ethyl 7-bromo-4-hydroxy-2-methylquinoline-3-carboxylate

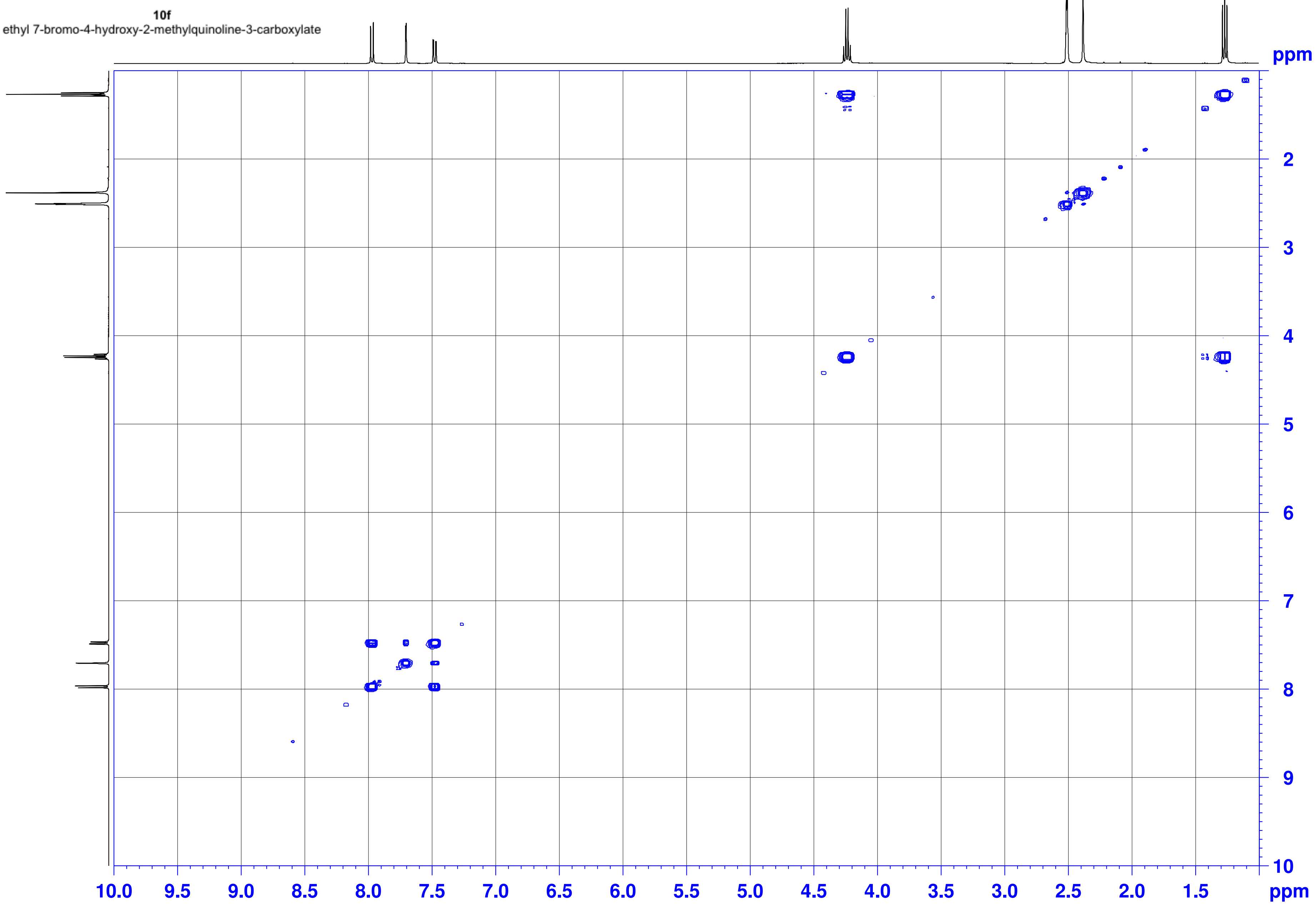

NGJ-8-030 (DMSO, 400 MHz) Pure White Solid - 7-bromoquinoline synthesis

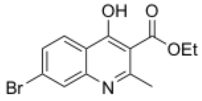

10f

ethyl 7-bromo-4-hydroxy-2-methylquinoline-3-carboxylate

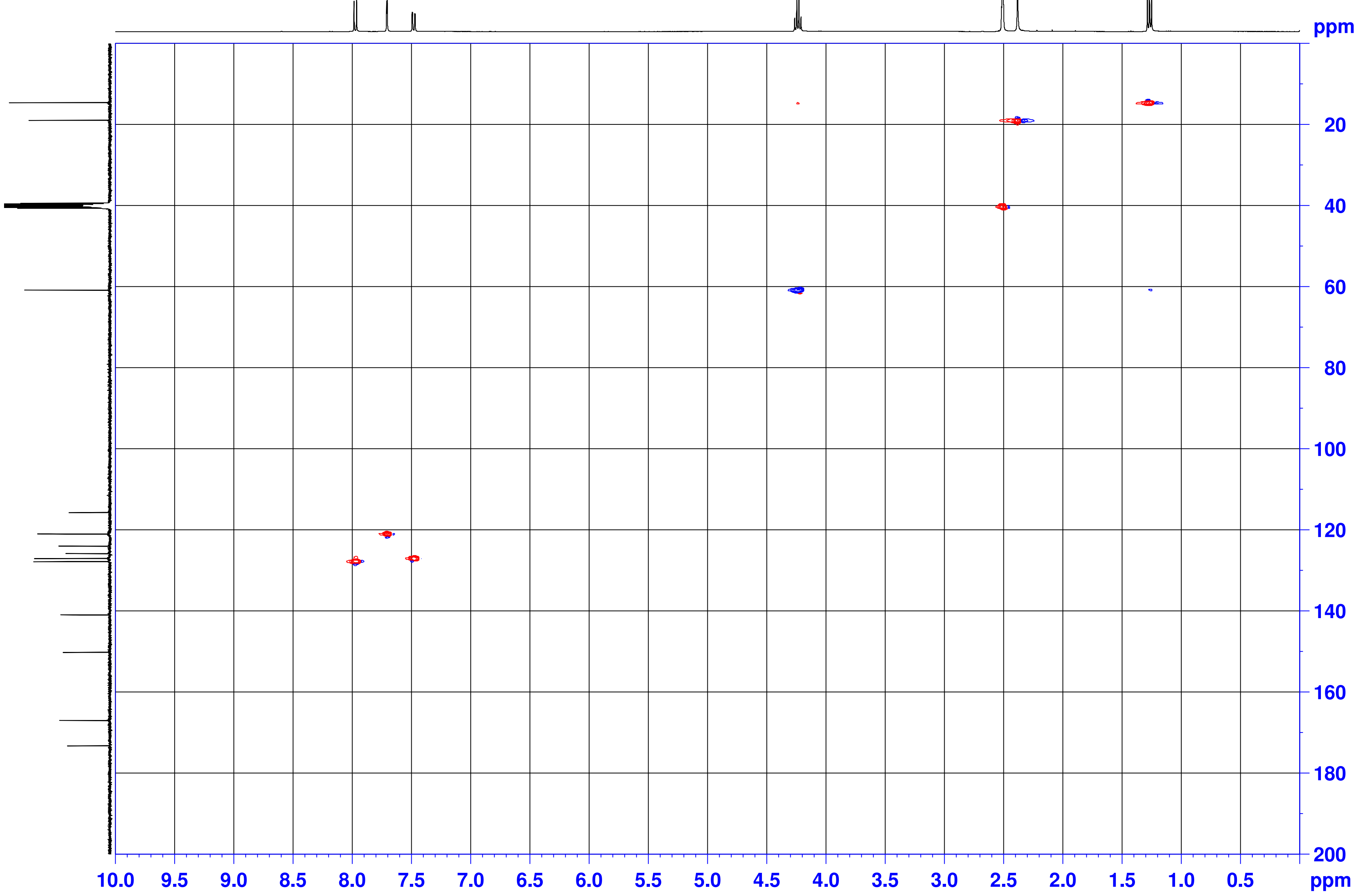

NGJ-8-030 (DMSO, 400 MHz) Pure White Solid - 7-bromoquinoline synthesis

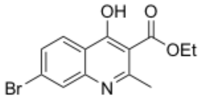

10f

ethyl 7-bromo-4-hydroxy-2-methylquinoline-3-carboxylate

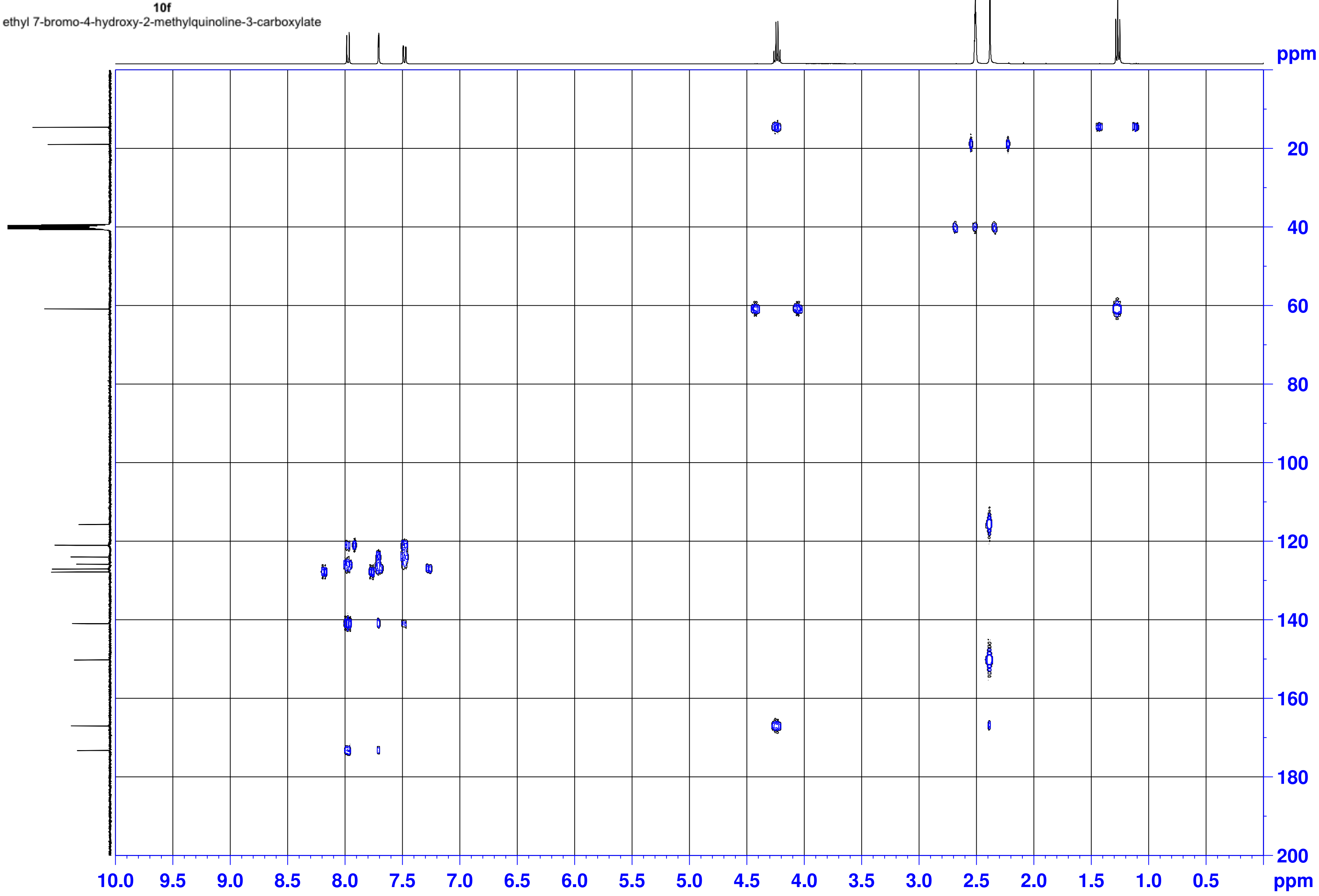

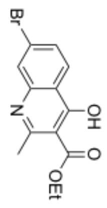

10f

ethyl 7-bromo-4-hydroxy-2-methylquinoline-3-carboxylate

Atlantic Microlab, Inc.

Sample No. NGT-08-030

6180 Atlantic Blvd. Suite M
Norcross, GA 30071
www.atlanticmicrolab.com

Professor/Supervisor: Matthew G. Donahue
PO# / CC# Mastercard 9359 (On file)

Company/School University of Southern Mississippi
Dept. Chemistry and Biochemistry
Address 118 College Dr. #5043
City, State, Zip Hattiesburg, MS 39406
Name Matthew G. Donahue Date 25-July-2018
Phone 614-203-1123

Element	Theory	Found	
C	50.34	49.75	49.85
H	3.90	3.89	3.88
		NO CHARGE FOR DUPLICATES	

Single ☒ Duplicate ☐
Elements Present: $C_{13}H_{12}BrNO_3$
Analyze for: C, H
Hygroscopic ☐ Explosive ☐
M.P. ____ B.P. ____
To be dried: Yes ☐ No ☒
Temp. ____ Vac. ____ Time ____
Rush Service ☐ Rush service guarantees analyses will be completed and results available by 5 PM EST on the day the sample is received by 11 AM.
Include Email Address or FAX # Below
matthew.donahue@usm.edu

Date Received JUL 30 2018 Date Completed JUL 31 2018
Remarks:

NGJ-8-035 (DMSO, 400 MHz) Pure White Solid - Synthesis of 8-bromoquinoline

NAME	NGJ-8-035	
EXPNO	20	
PROCNO	1	
Date_	20170830	
Time	21.24	h
INSTRUM	spect	
PROBHD	Z108618_0161 (	
PULPROG	zg30	
TD	65536	
SOLVENT	DMSO	
NS	16	
DS	2	
SWH	8012.820	Hz
FIDRES	0.244532	Hz
AQ	4.0894966	sec
RG	114	
DW	62.400	usec
DE	6.50	usec
TE	298.2	K
D1	1.00000000	sec
TD0	1	
SFO1	400.1724710	MHz
NUC1	1H	
P1	9.88	usec
SI	65536	
SF	400.1699960	MHz
WDW	EM	
SSB	0	
LB	0.30	Hz
GB	0	
PC	1.00	

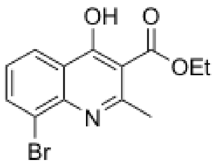

10h

ethyl 8-bromo-4-hydroxy-2-methylquinoline-3-carboxylate

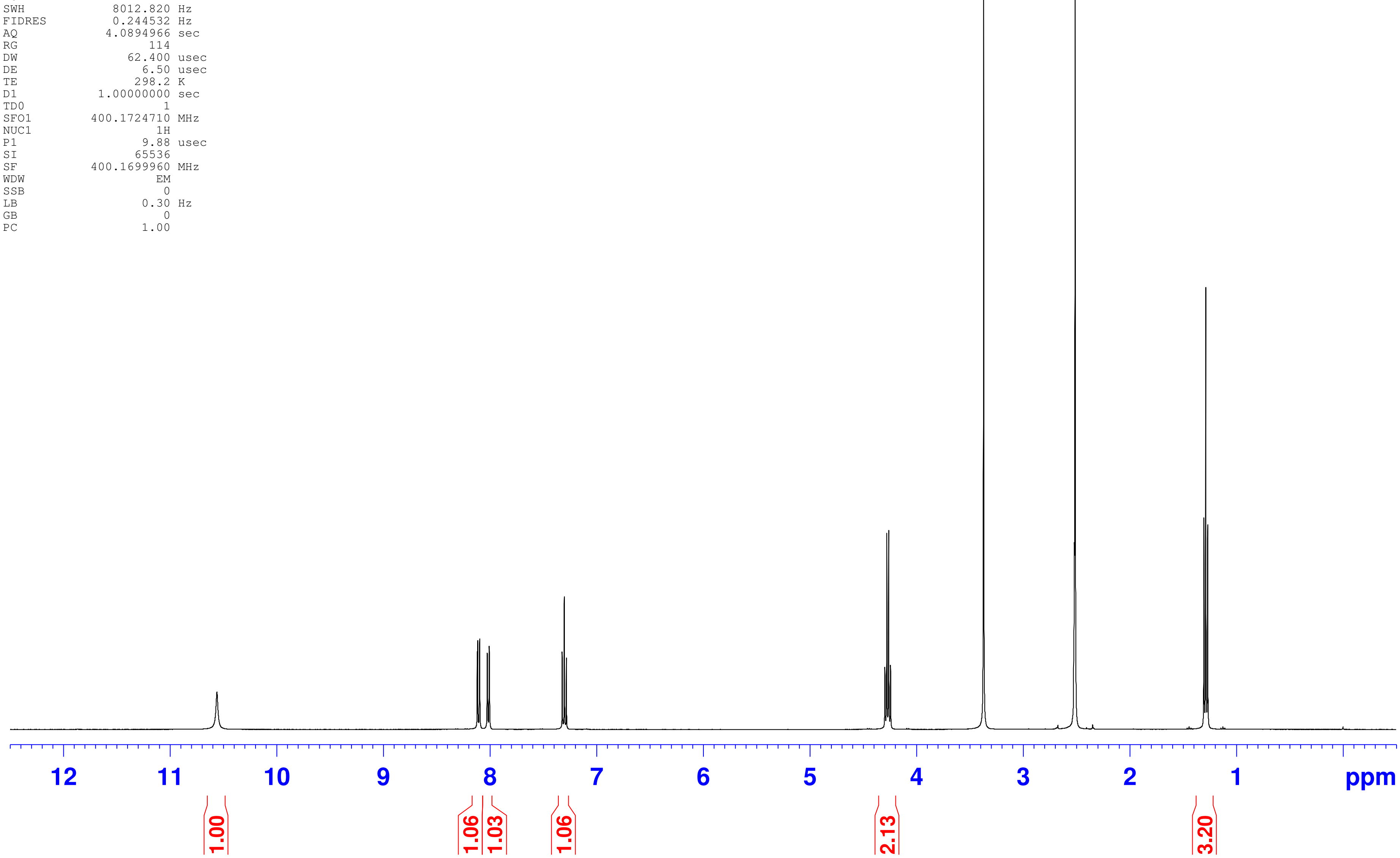

NGJ-8-035 (DMSO, 400 MHz) Pure White Solid - Synthesis of 8-b[illegible]

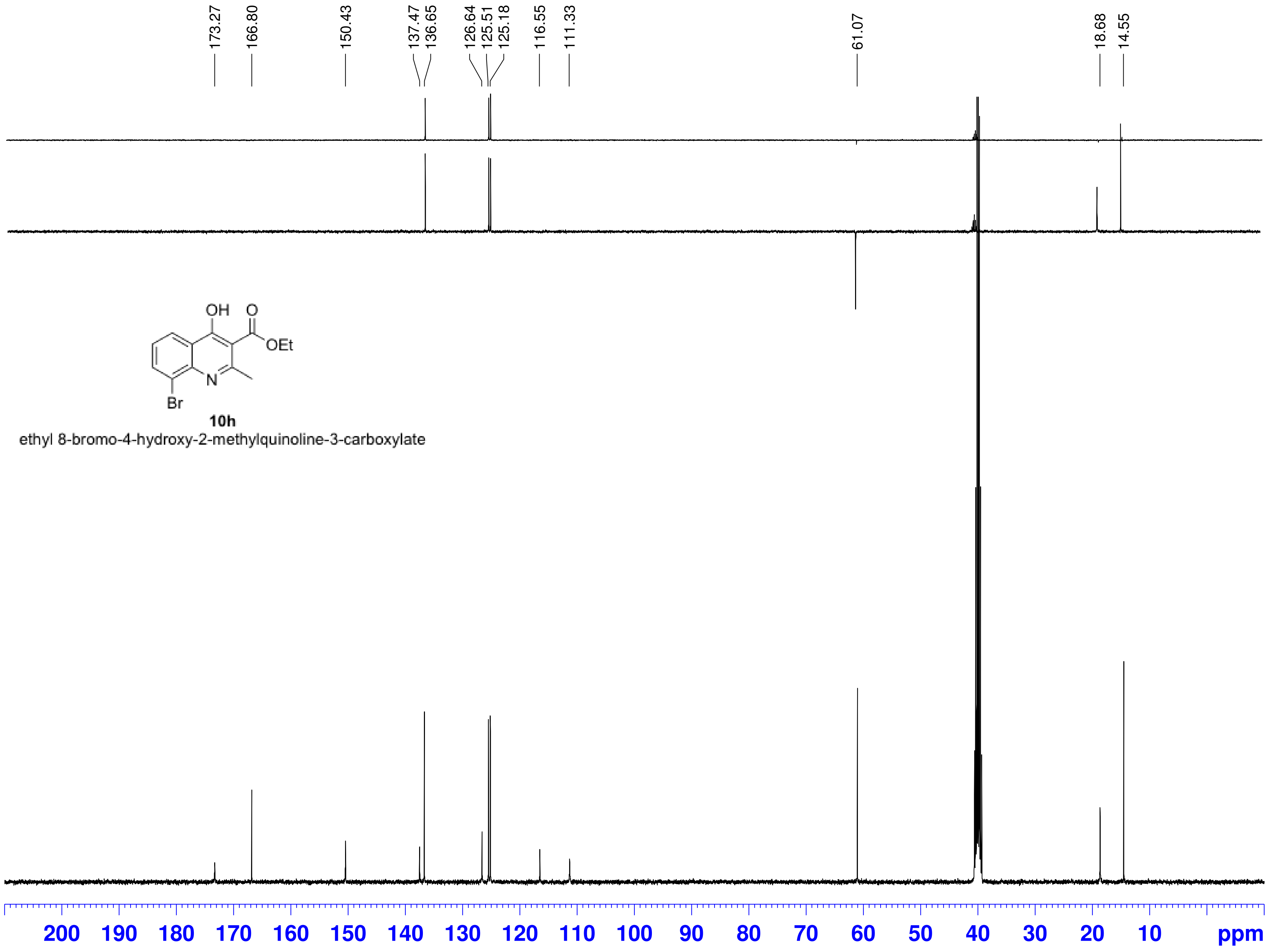

10h

ethyl 8-bromo-4-hydroxy-2-methylquinoline-3-carboxylate

NGJ-8-035 (DMSO, 400 MHz) Pure White Solid - Synthesis of 8-bromoquinoline

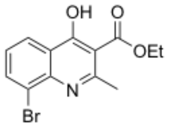

10h

ethyl 8-bromo-4-hydroxy-2-methylquinoline-3-carboxylate

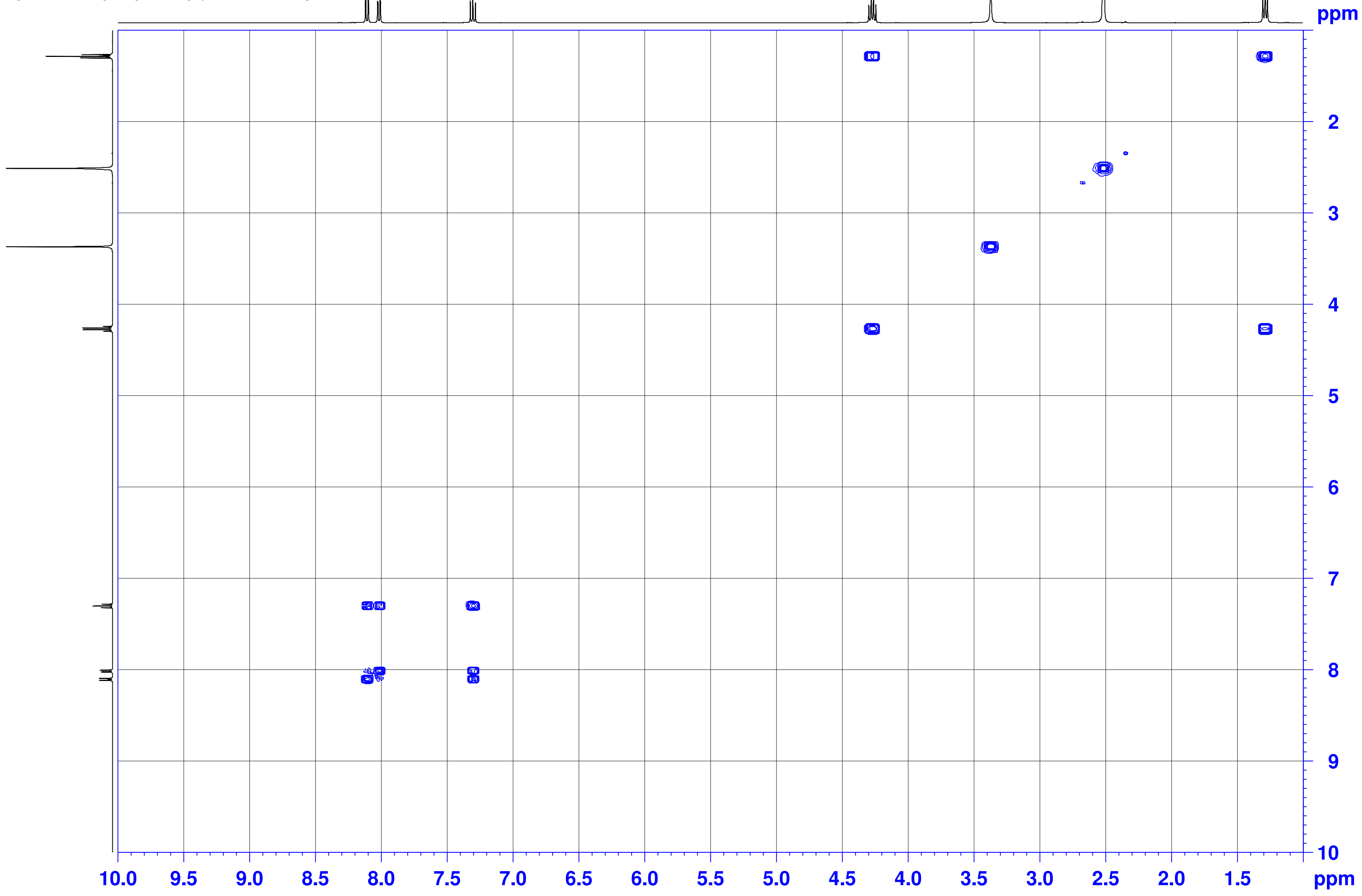

NGJ-8-035 (DMSO, 400 MHz) Pure White Solid - Synthesis of 8-bromoquinoline

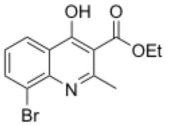

10h

ethyl 8-bromo-4-hydroxy-2-methylquinoline-3-carboxylate

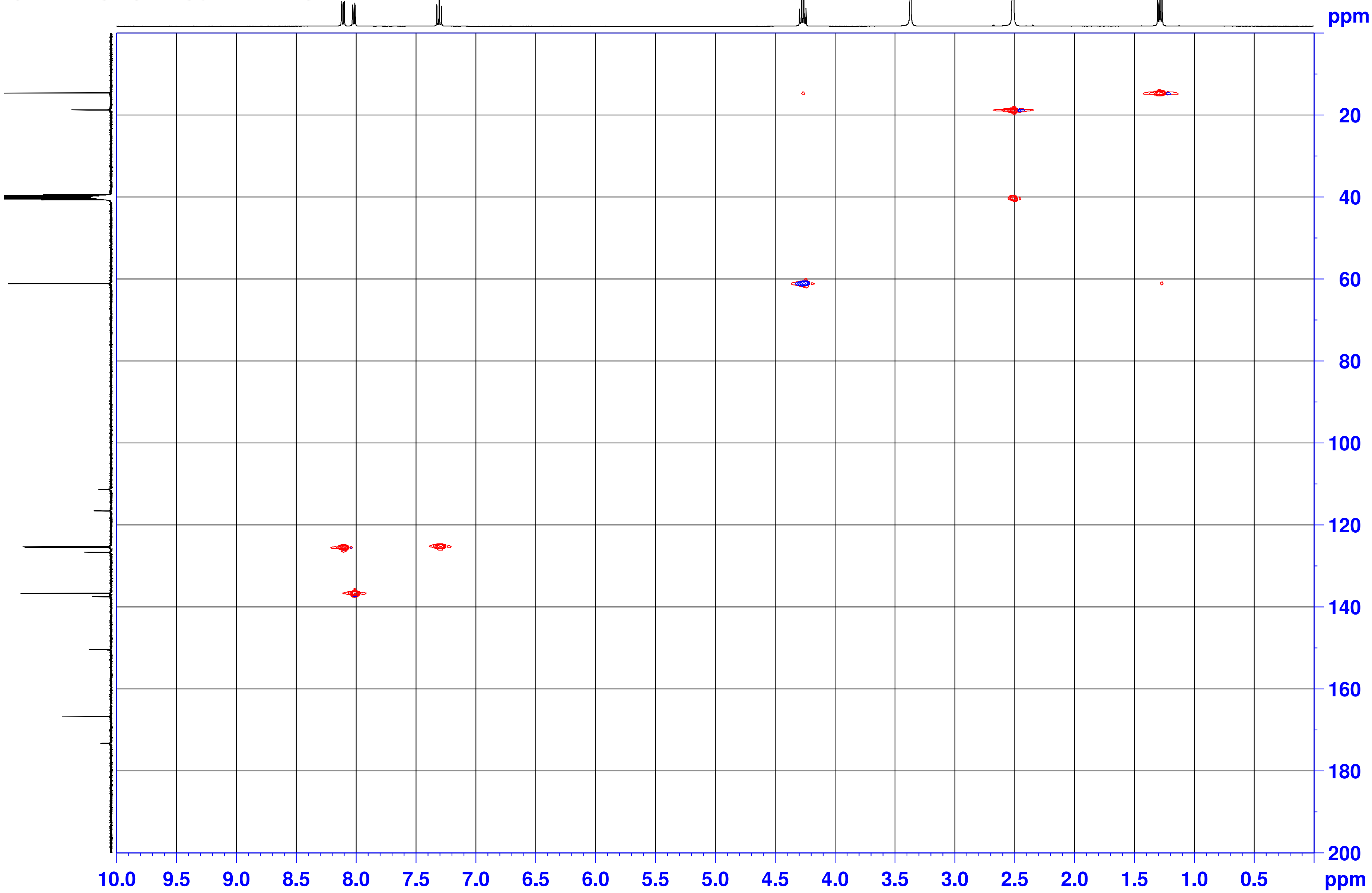

NGJ-8-035 (DMSO, 400 MHz) Pure White Solid - Synthesis of 8-bromoquinoline

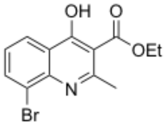

10h

ethyl 8-bromo-4-hydroxy-2-methylquinoline-3-carboxylate

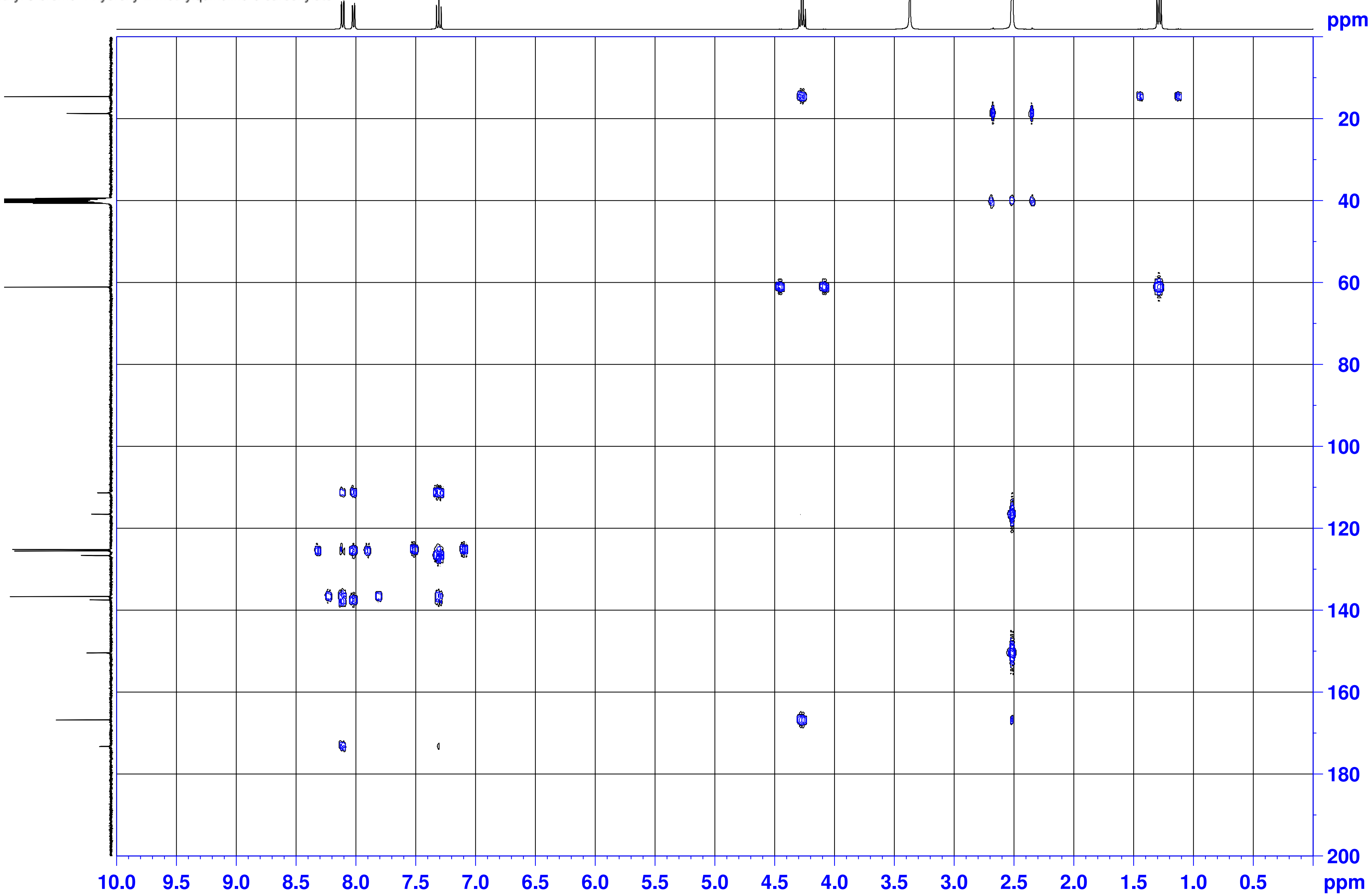

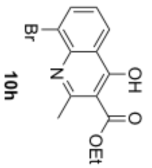

10h

ethyl 8-bromo-4-hydroxy-2-methylquinoline-3-carboxylate

Atlantic Microlab, Inc.

Sample No. NGJ-8-035

6180 Atlantic Blvd. Suite M
Norcross, GA 30071
www.atlanticmicrolab.com

Company/School University of Southern Mississippi
Dept. Chemistry and Biochemistry
Address 118 College Dr. #5043
City, State, Zip Hattiesburg, MS 39406
Name Matthew Donahue Date 31. Aug 2017
Phone 614-203-1123

Professor/Supervisor: Matthew G. Donahue
PO# / CC# Mastercard 9359 (On file)

Element	Theory	Found	
C	50.34	47.63	47.50
H	3.90	4.24	4.34
		NO CHARGE FOR DUPLICATES	

Single ☒ Duplicate ☐
Elements Present: $C_{13}H_{12}BrNO_3$
Analyze for: C, H
Hygroscopic ☐ Explosive ☐
M.P. ______ B.P. ______
To be dried: Yes ☐ No ☒
Temp. ______ Vac. ______ Time ______
Rush Service ☐ Rush service guarantees analyses will be completed and results available by 5 PM EST on the day the sample is received by 11 AM.
Include Email Address or FAX # Below
matthew.donahue@usm.edu

Date Received SEP 06 2017 Date Completed SEP 07 2017
Remarks:

AHP-1-048-3 (DMSO-d6, 400 MHz) Off white filter cake from methanol trituration

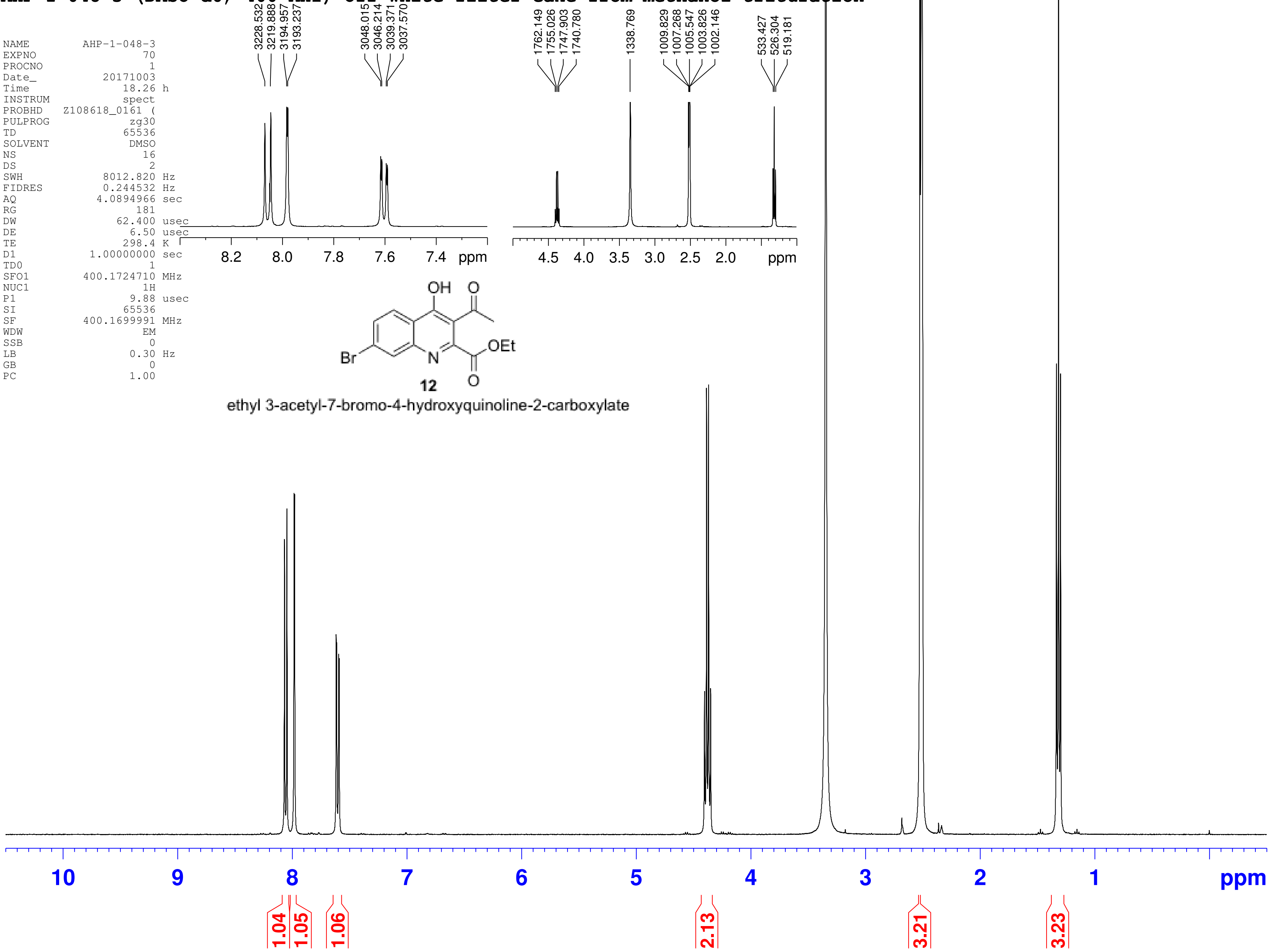

12

ethyl 3-acetyl-7-bromo-4-hydroxyquinoline-2-carboxylate

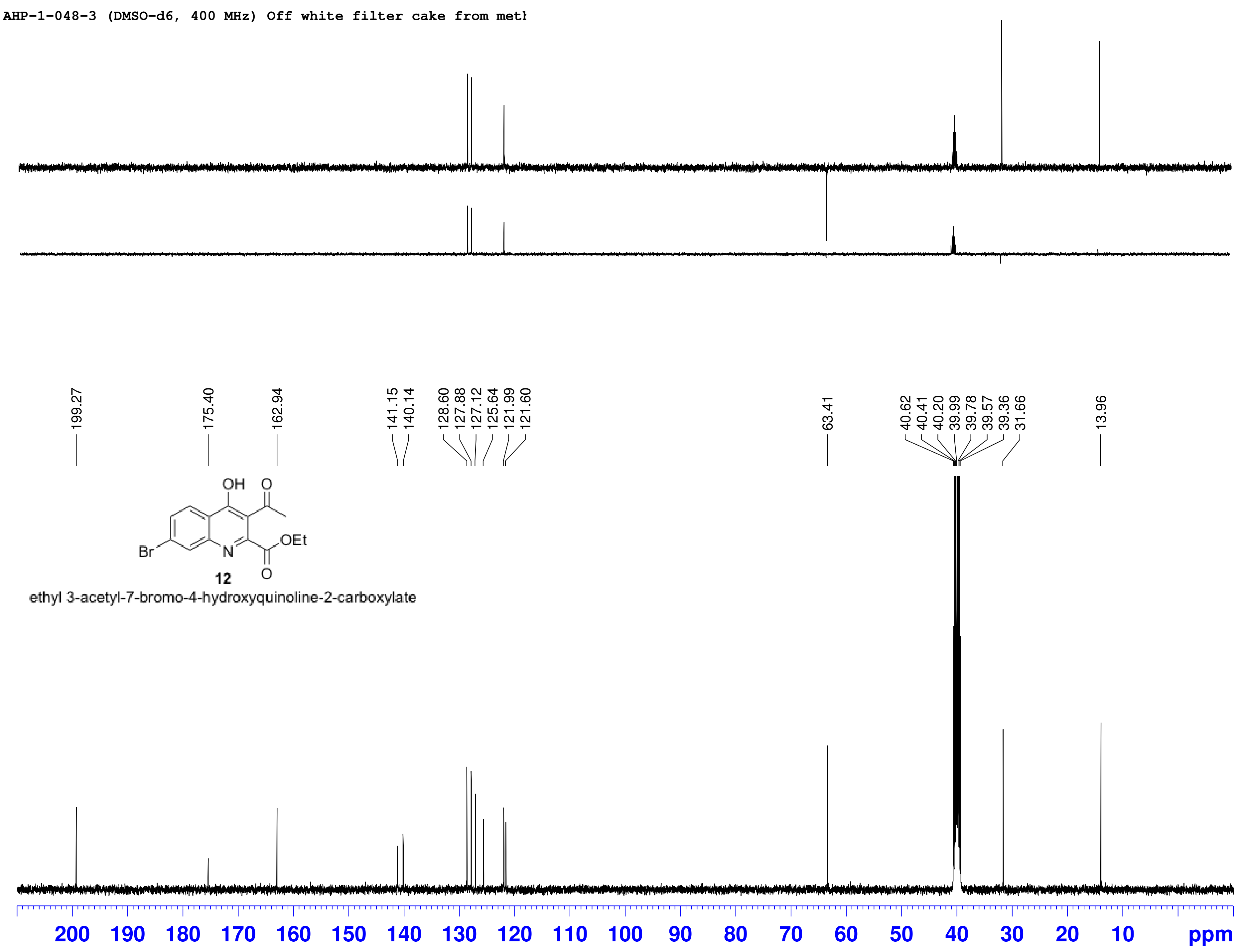
AHP-1-048-3 (DMSO-d6, 400 MHz) Off white filter cake from meth
199.27
175.40
162.94
141.15
140.14
128.60
127.88
127.12
125.64
121.99
121.60
63.41
40.62
40.41
40.20
39.99
39.78
39.57
39.36
31.66
13.96
OH
O
OEt
Br
N
O
12
ethyl 3-acetyl-7-bromo-4-hydroxyquinoline-2-carboxylate
200
190
180
170
160
150
140
130
120
110
100
90
80
70
60
50
40
30
20
10
ppm

AHP-1-048-3 (DMSO-d6, 400 MHz) Off white filter cake from methanol trituration

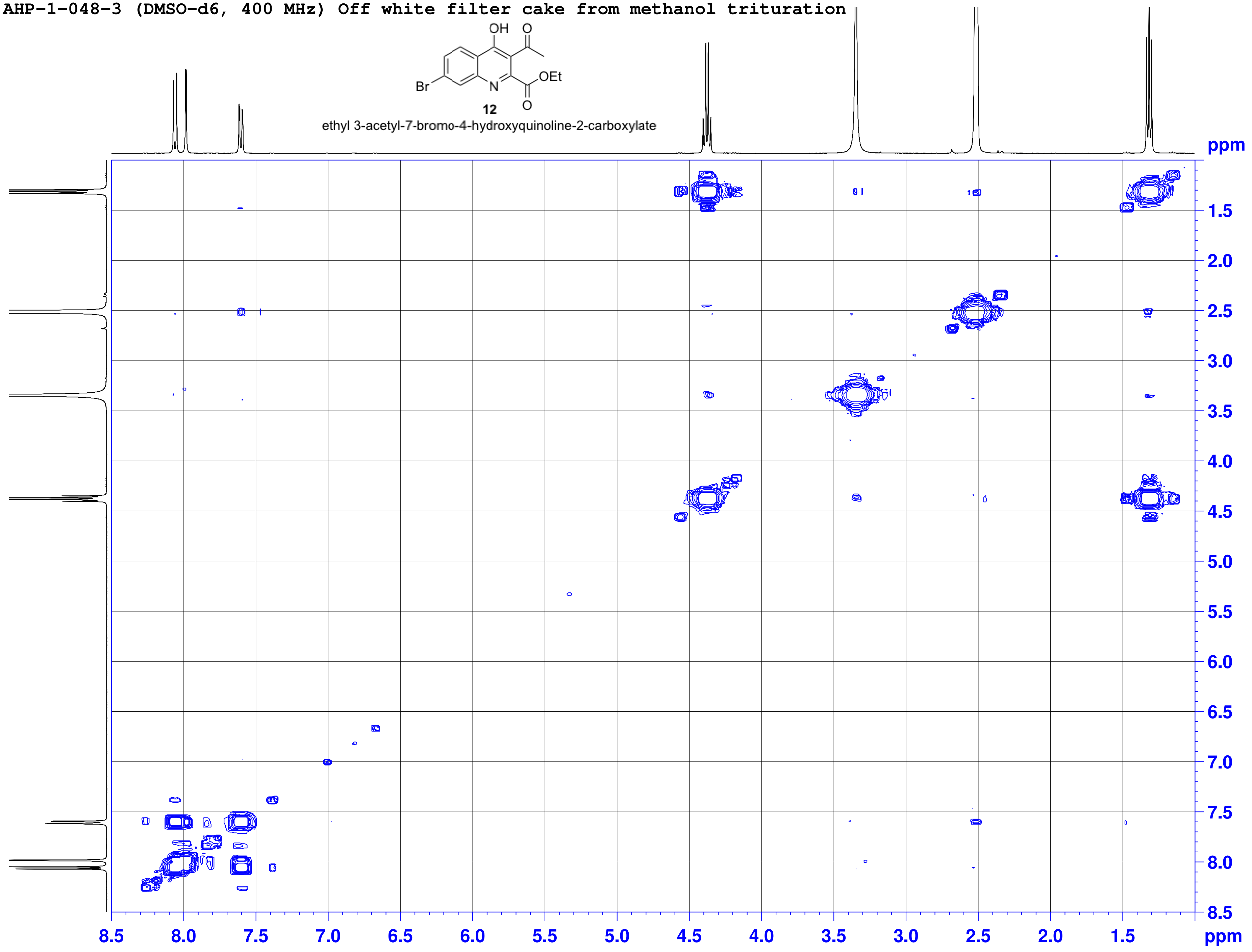

AHP-1-048-3 (DMSO-d6, 400 MHz) Off white filter cake from methanol trituration

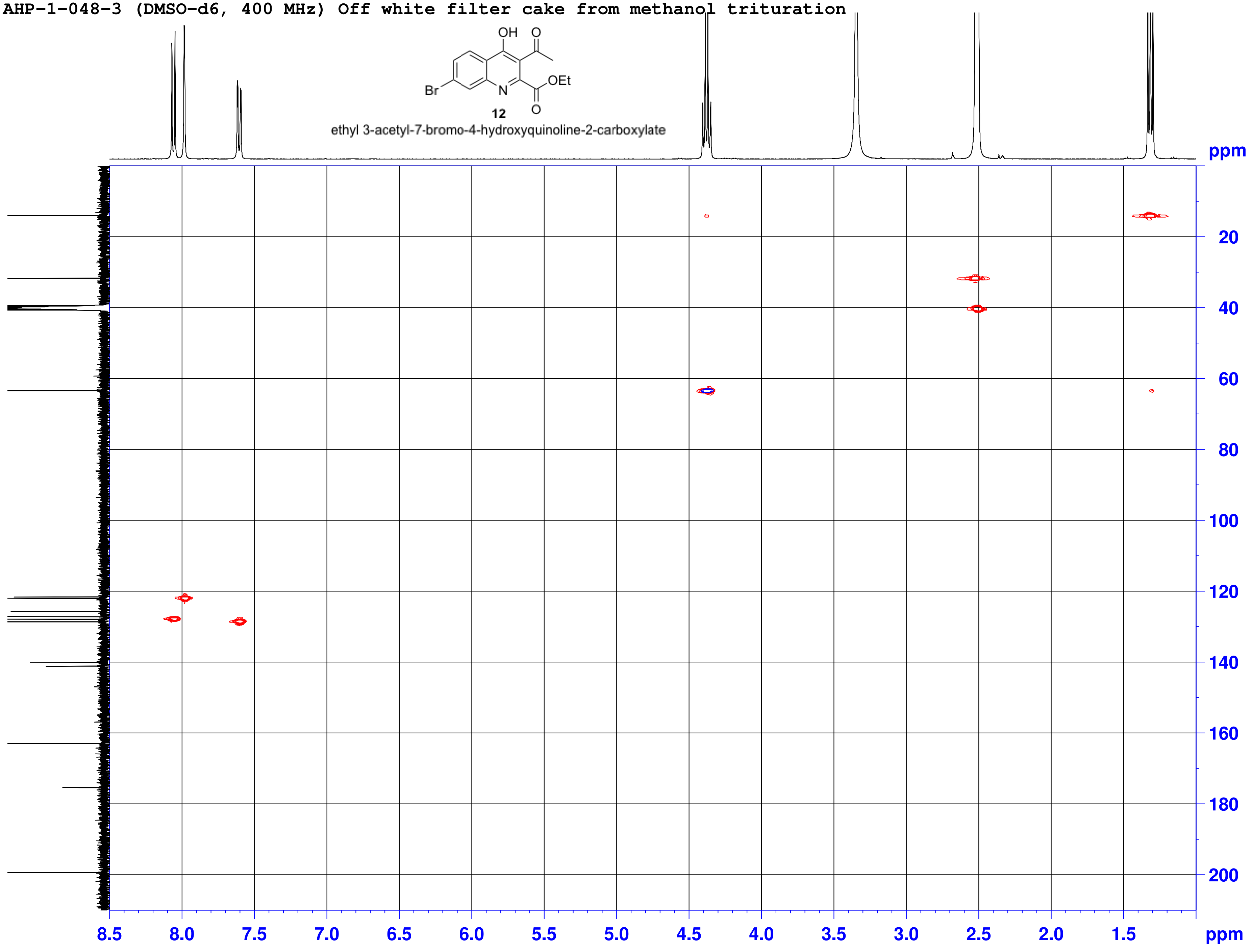

AHP-1-048-3 (DMSO-d6, 400 MHz) Off white filter cake from methanol trituration

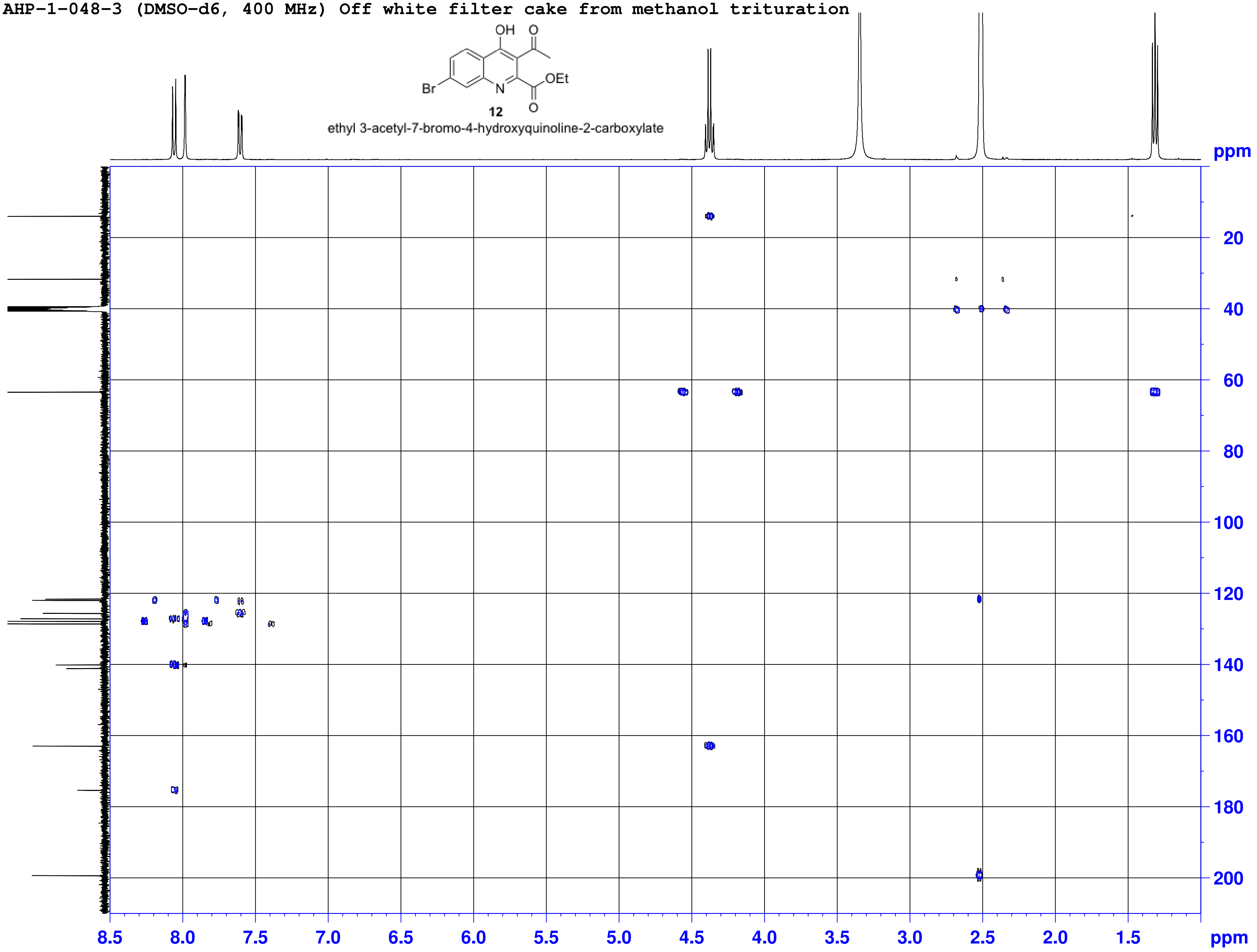

Atlantic Microlab, Inc.

Sample No. AHP-1-048-3

6180 Atlantic Blvd. Suite M
Norcross, GA 30071
www.atlanticmicrolab.com

Company/School University of Southern Mississippi
Dept. Chemistry and Biochemistry
Address 118 College Dr. #5043
City, State, Zip Hattiesburg, MS 39406
Name Matthew Donahue
Phone 614-203-1123
Date 04-OCT-2017

Professor/Supervisor: Matthew G. Donahue
PO# / CC# ! Visa 5794

Element	Theory	Found	
C	49.73	49.81	
H	3.58	3.47	

Single ☒ Duplicate ☐

Elements Present: $C_{14}H_{12}BrNO_4$

Analyze for: C, H

Hygroscopic ☐ Explosive ☐

M.P. 198-201°C B.P.

To be dried: Yes ☐ No ☒

Temp. ____ Vac. ____ Time ____

Rush Service ☐ Rush service guarantees analyses will be completed and results available by 5 PM EST on the day the sample is received by 11 AM.

Include Email Address or FAX # Below

matthew.donahue@usm.edu

Date Received JUL 30 2018 Date Completed JUL 31 2018

Remarks:

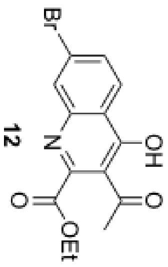

12

ethyl 3-acetyl-7-bromo-4-hydroxyquinoline-2-carboxylate

JDH-1-216 (400MHz, CDCl3) Chlorination of 4-Hydroxyquinoline

NAME	JDH-1-216
EXPNO	40
PROCNO	1
Date_	20171027
Time	10.29 h
INSTRUM	spect
PROBHD	Z108618_0161 (
PULPROG	zg30
TD	65536
SOLVENT	CDCl3
NS	16
DS	2
SWH	8012.820 Hz
FIDRES	0.244532 Hz
AQ	4.0894966 sec
RG	32
DW	62.400 usec
DE	6.50 usec
TE	298.3 K
D1	1.00000000 sec
TD0	1
SFO1	400.1724710 MHz
NUC1	1H
P1	9.88 usec
SI	65536
SF	400.1699764 MHz
WDW	EM
SSB	0
LB	0.30 Hz
GB	0
PC	1.00

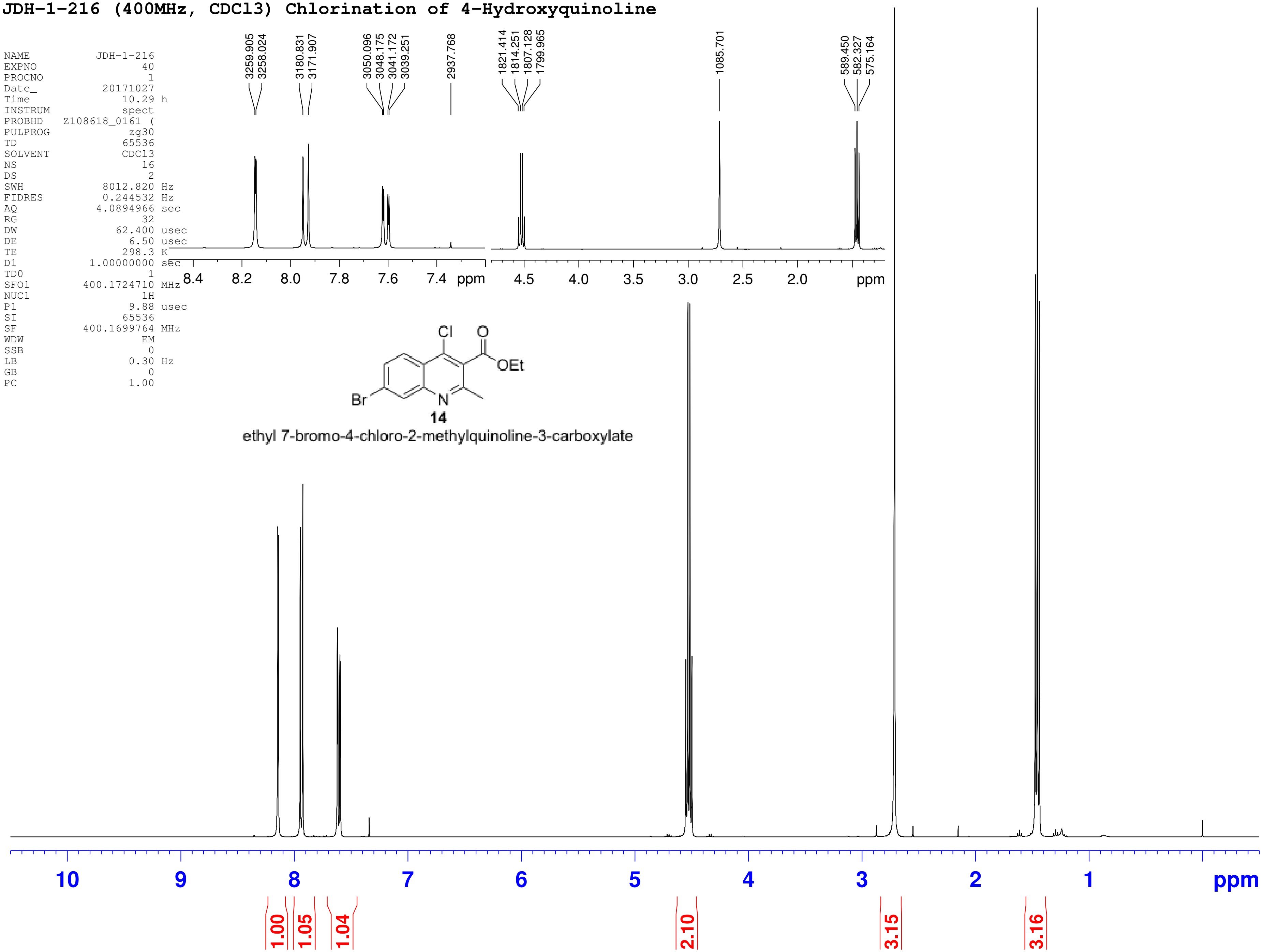

14

ethyl 7-bromo-4-chloro-2-methylquinoline-3-carboxylate

JDH-1-216 (400MHz, CDCl3) Chlorination of 4-hydroxyquinoline

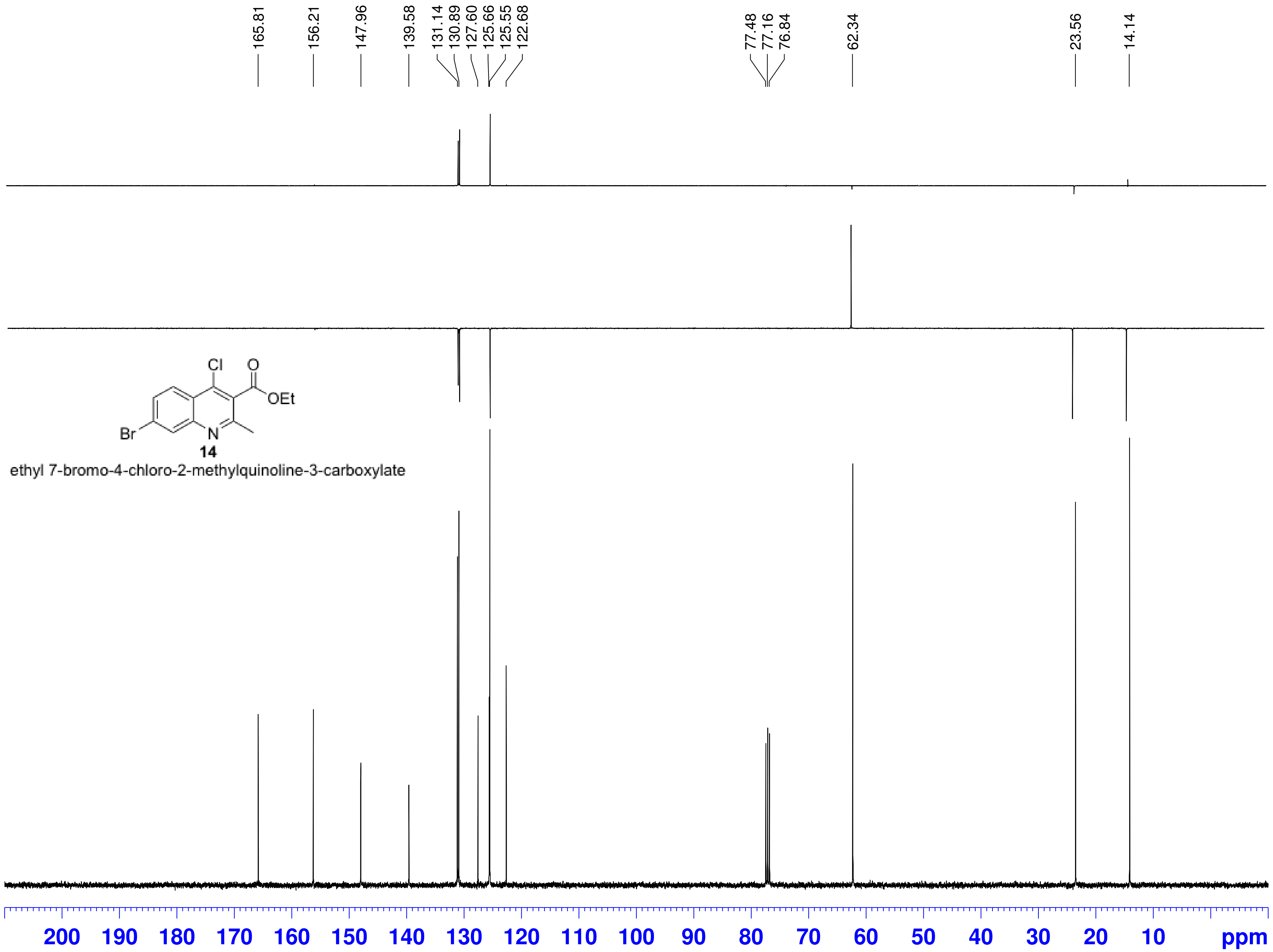

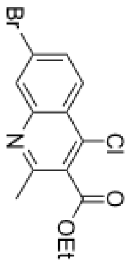

14

ethyl 7-bromo-4-chloro-2-methylquinoline-3-carboxylate

Atlantic Microlab, Inc.

Sample No. SKW-1-085

6180 Atlantic Blvd. Suite M
Norcross, GA 30071
www.atlanticmicrolab.com

Company/School University of Southern Mississippi
Dept. Chemistry and Biochemistry
Address 118 College Dr. #5043
City, State, Zip Hattiesburg, MS 39406
Name Matthew G. Donahue Date 25 July 2018
Phone 614-203-1123

Professor/Supervisor: Matthew G. Donahue
PO# / CC# Mastercard 9359 (On file)

Element	Theory	Found	
C	47.52	44.86	44.72
H	3.37	3.23	3.19
		NO CHARGE FOR DUPLICATES	

Single ☒ Duplicate ☐
Elements Present: $C_{13}H_{11}BrClNO_2$
Analyze for: C, H
Hygroscopic ☐ Explosive ☐
M.P. ______ B.P. ______
To be dried: Yes ☐ No ☒
Temp. ______ Vac. ______ Time ______
Rush Service ☐ Rush service guarantees analyses will be completed and results available by 5 PM EST on the day the sample is received by 11 AM.
Include Email Address or FAX # Below
matthew.donahue@usm.edu

Date Received JUL 30 2018 Date Completed JUL 31 2018
Remarks:

JDH-1-229B DIBAL-H reduction (400MHz, CDCl3)

NAME	JDH-1-229	
EXPNO	40	
PROCNO	1	
Date_	20171114	
Time	18.57	h
INSTRUM	spect	
PROBHD	Z108618_0161 (	
PULPROG	zg30	
TD	65536	
SOLVENT	CDCl3	
NS	16	
DS	2	
SWH	8012.820	Hz
FIDRES	0.244532	Hz
AQ	4.0894966	sec
RG	203	
DW	62.400	usec
DE	6.50	usec
TE	298.2	K
D1	1.00000000	sec
TD0	1	
SFO1	400.1724710	MHz
NUC1	1H	
P1	9.88	usec
SI	65536	
SF	400.1699946	MHz
WDW	EM	
SSB	0	
LB	0.30	Hz
GB	0	
PC	1.00	

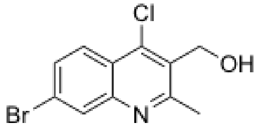

SI-1
(7-bromo-4-chloro-2-methylquinolin-3-yl)methanol

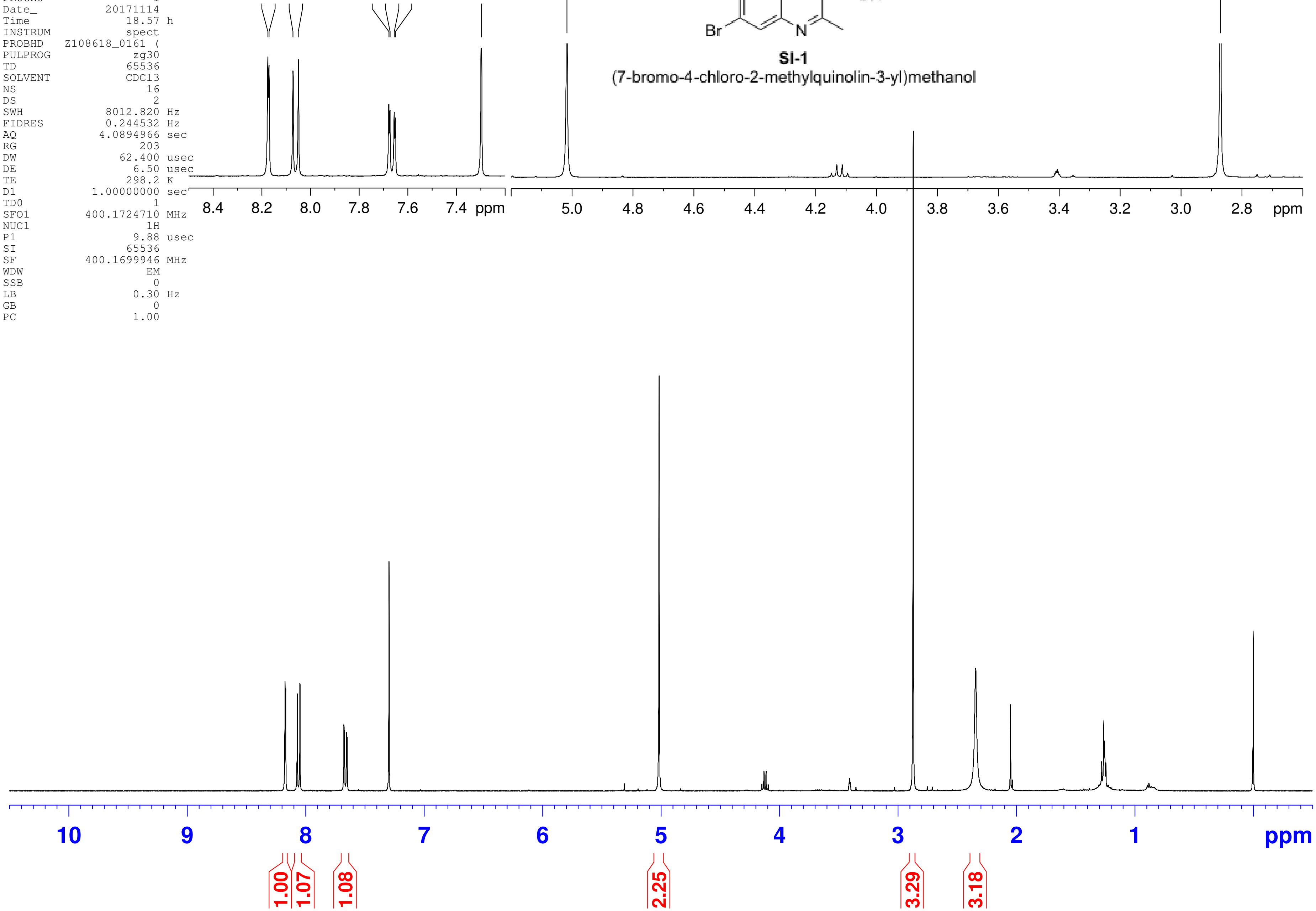

JDH-1-229B DIBAL-H reduction (400MHz, CDCl3)

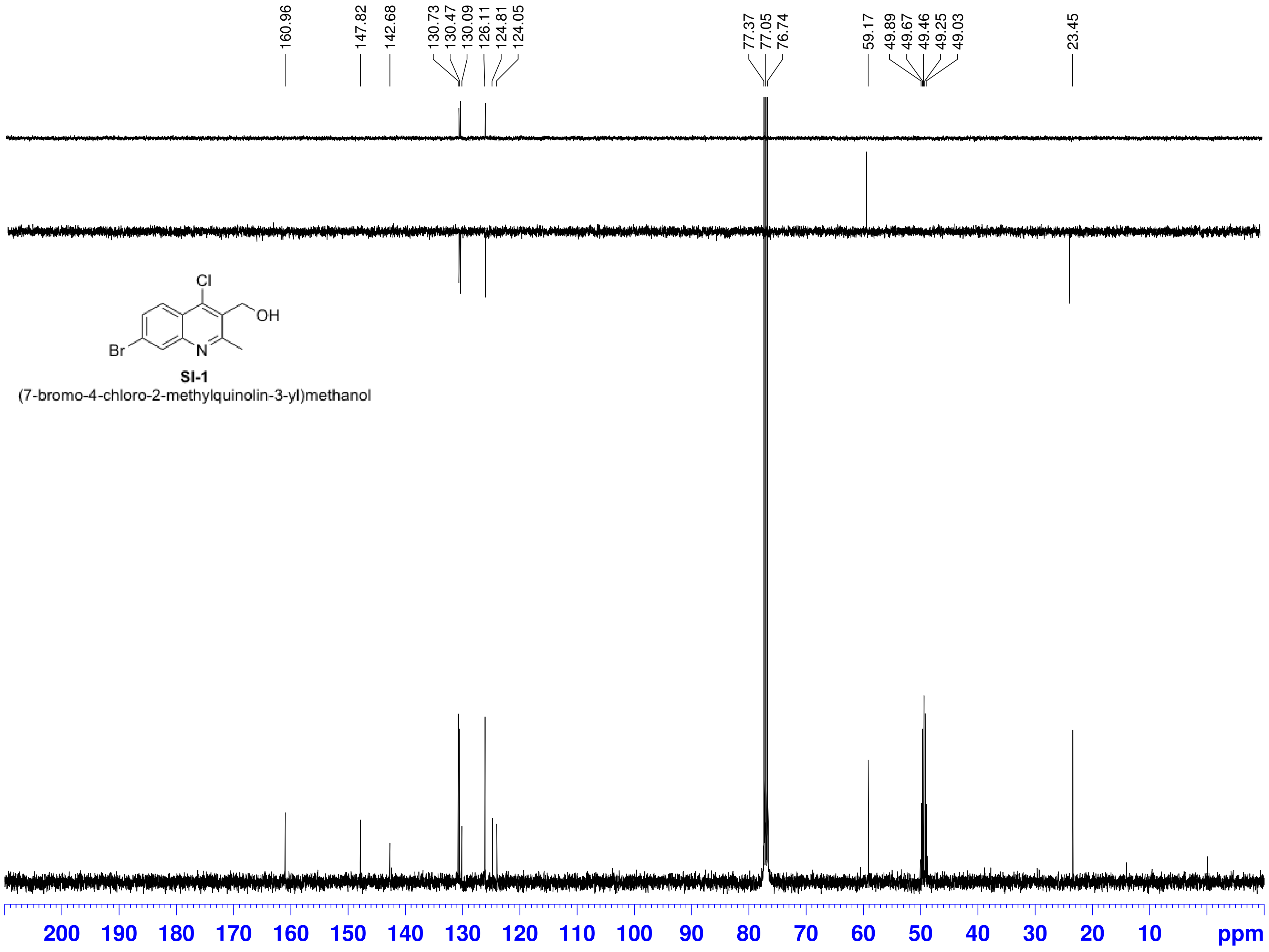

JDH-1-226 Parikh-Doering Oxidation (CDCl3, 400MHz)

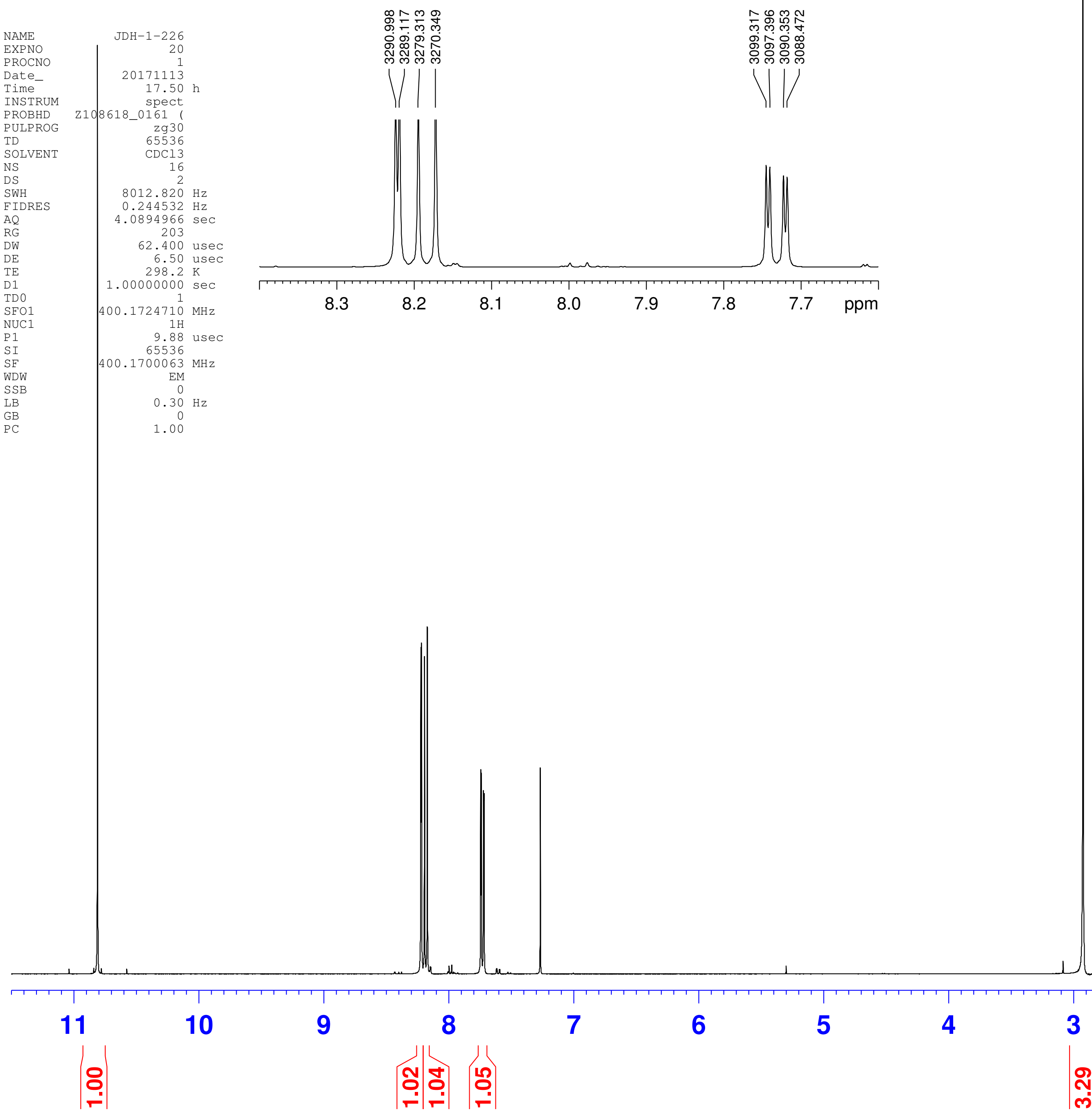

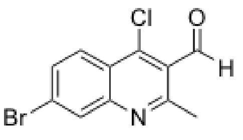

15

7-bromo-4-chloro-2-methylquinoline-3-carbaldehyde

JDH-1-226B Pyr*SO3 oxidation (400MHz, CDCl3)

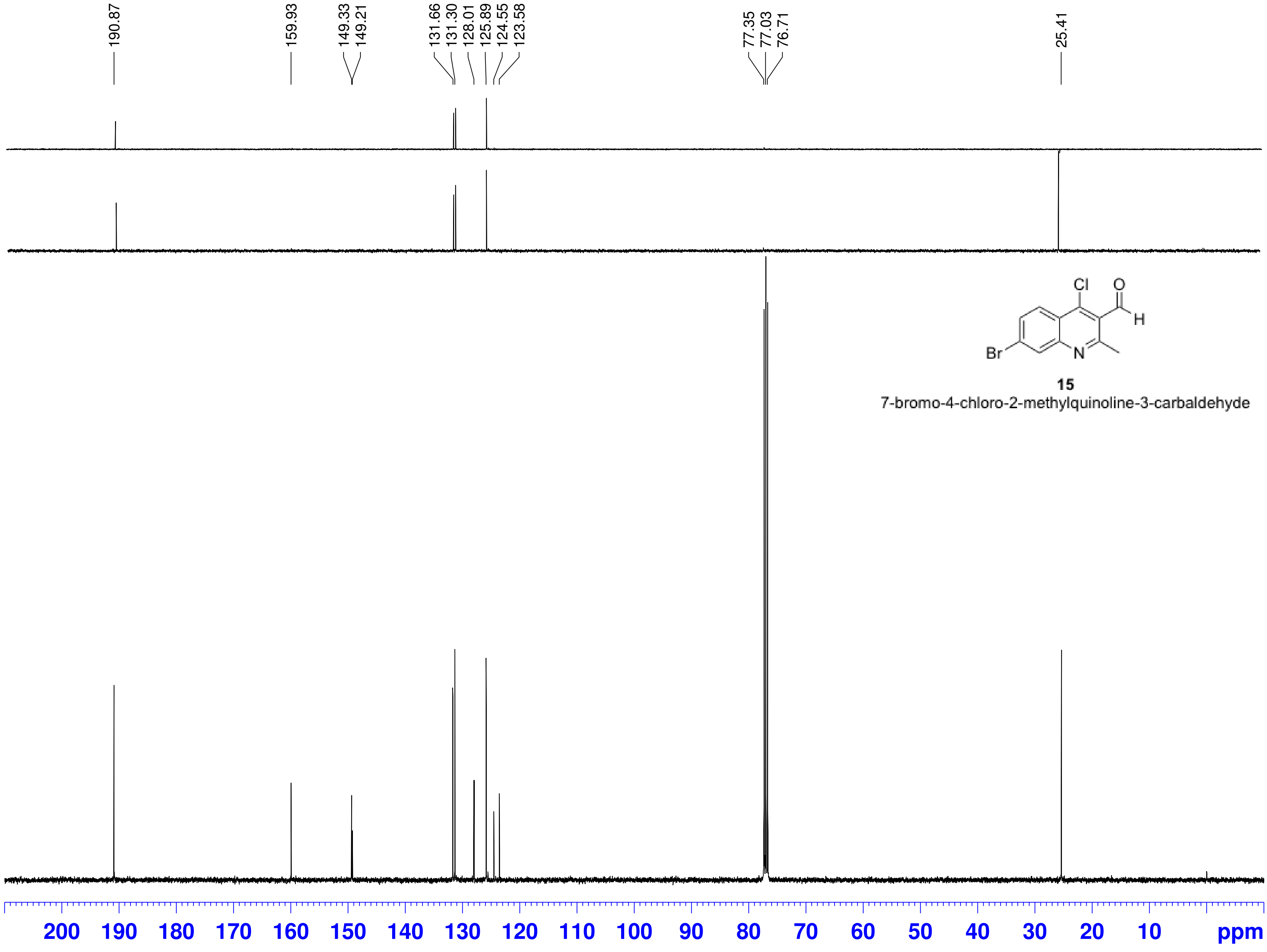

15

7-bromo-4-chloro-2-methylquinoline-3-carbaldehyde

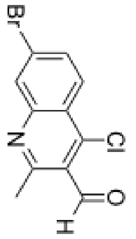

15

7-bromo-4-chloro-2-methylquinoline-3-carbaldehyde

Atlantic Microlab, Inc.

Sample No. NGJ-10-007

6180 Atlantic Blvd. Suite M
Norcross, GA 30071
www.atlanticmicrolab.com

Company/School University of Southern Mississippi
Dept. Chemistry and Biochemistry
Address 118 College Dr. #5043
City, State, Zip Hattiesburg, MS 39406
Name Matthew G. Donahue   Date 25-July-2018
Phone 614-203-1123

Professor/Supervisor: Matthew G. Donahue
PO# / CC# Mastercard 9359 (On file)

Element	Theory	Found	
C	46.43	45.72	45.69
H	2.48	2.41	2.38
		NO CHARGE FOR DUPLICATES	

Single ☒ Duplicate ☐
Elements Present: $C_{11}H_7BrClNO$
Analyze for: C, H
Hygroscopic ☐ Explosive ☐
M.P. ______ B.P. ______
To be dried: Yes ☐ No ☒
Temp. ______ Vac. ______ Time ______
Rush Service ☐ Rush service guarantees analyses will be completed and results available by 5 PM EST on the day the sample is received by 11 AM.
Include Email Address or FAX # Below
matthew.donahue@usm.edu

Date Received JUL 30 2018   Date Completed JUL 31 2018
Remarks:

JDH-1-232 Cyanosilyation (400MHz, CDCl3)

16

2-(7-bromo-4-chloro-2-methylquinolin-3-yl)-2-((trimethylsilyl)oxy)acetonitrile

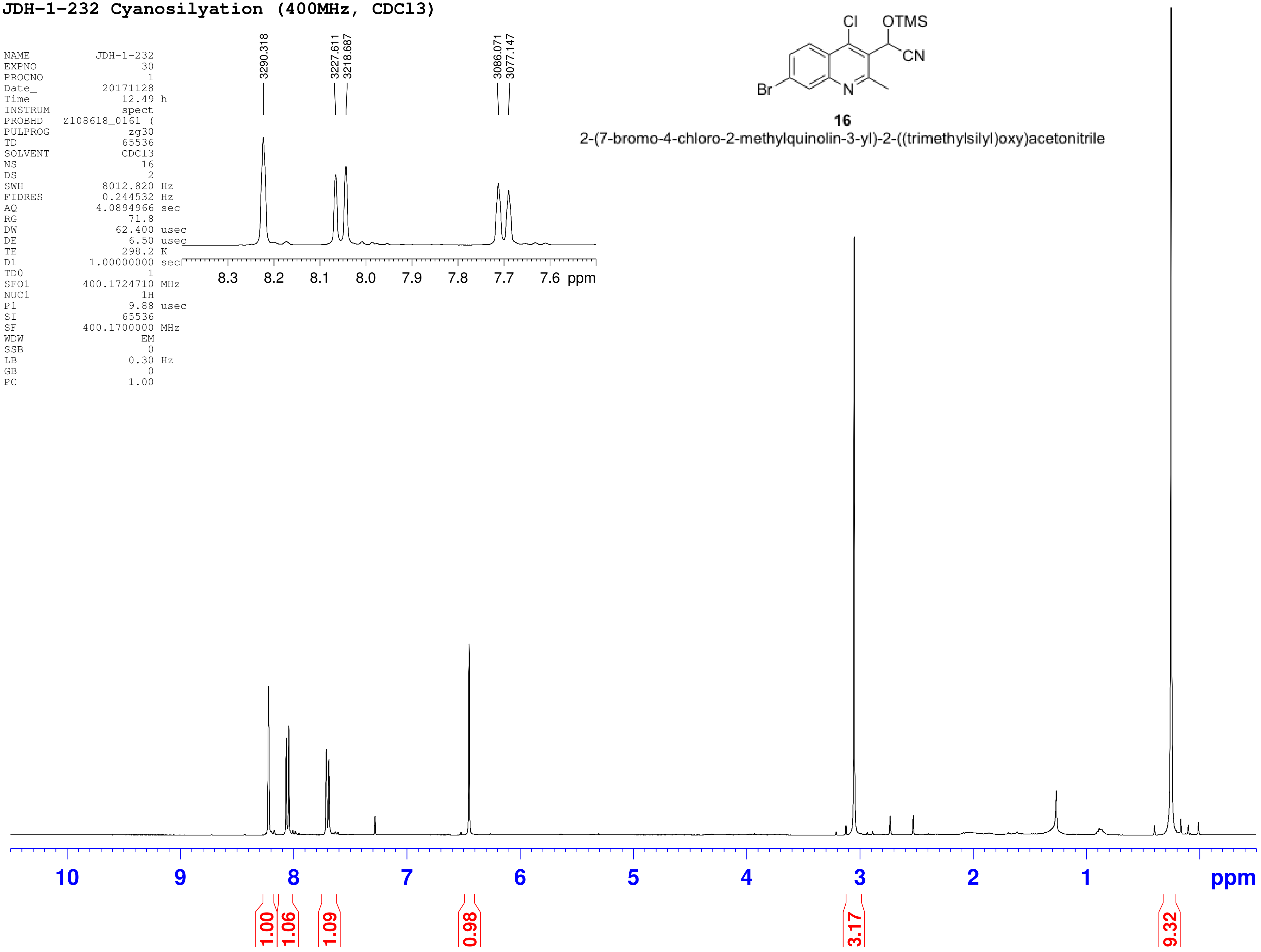

JDH-1-232 Cyanosilyation (400MHz, CDCl3)

NAME	JDH-1-232
EXPNO	31
PROCNO	1
Date_	20171128
Time	13.03 h
INSTRUM	spect
PROBHD	Z108618_0161 (
PULPROG	deptqgpsp
TD	65536
SOLVENT	CDCl3
NS	216
DS	4
SWH	24038.461 Hz
FIDRES	0.733596 Hz
AQ	1.3631988 sec
RG	203
DW	20.800 usec
DE	6.50 usec
TE	298.5 K
CNST2	145.0000000
D1	2.00000000 sec
D2	0.00344828 sec
D12	0.00002000 sec
D16	0.00020000 sec
TD0	1
SFO1	100.6328888 MHz
NUC1	13C
P1	8.40 usec
P13	2000.00 usec
SI	32768
SF	100.6227899 MHz
WDW	EM
SSB	0
LB	1.00 Hz
GB	0
PC	1.40

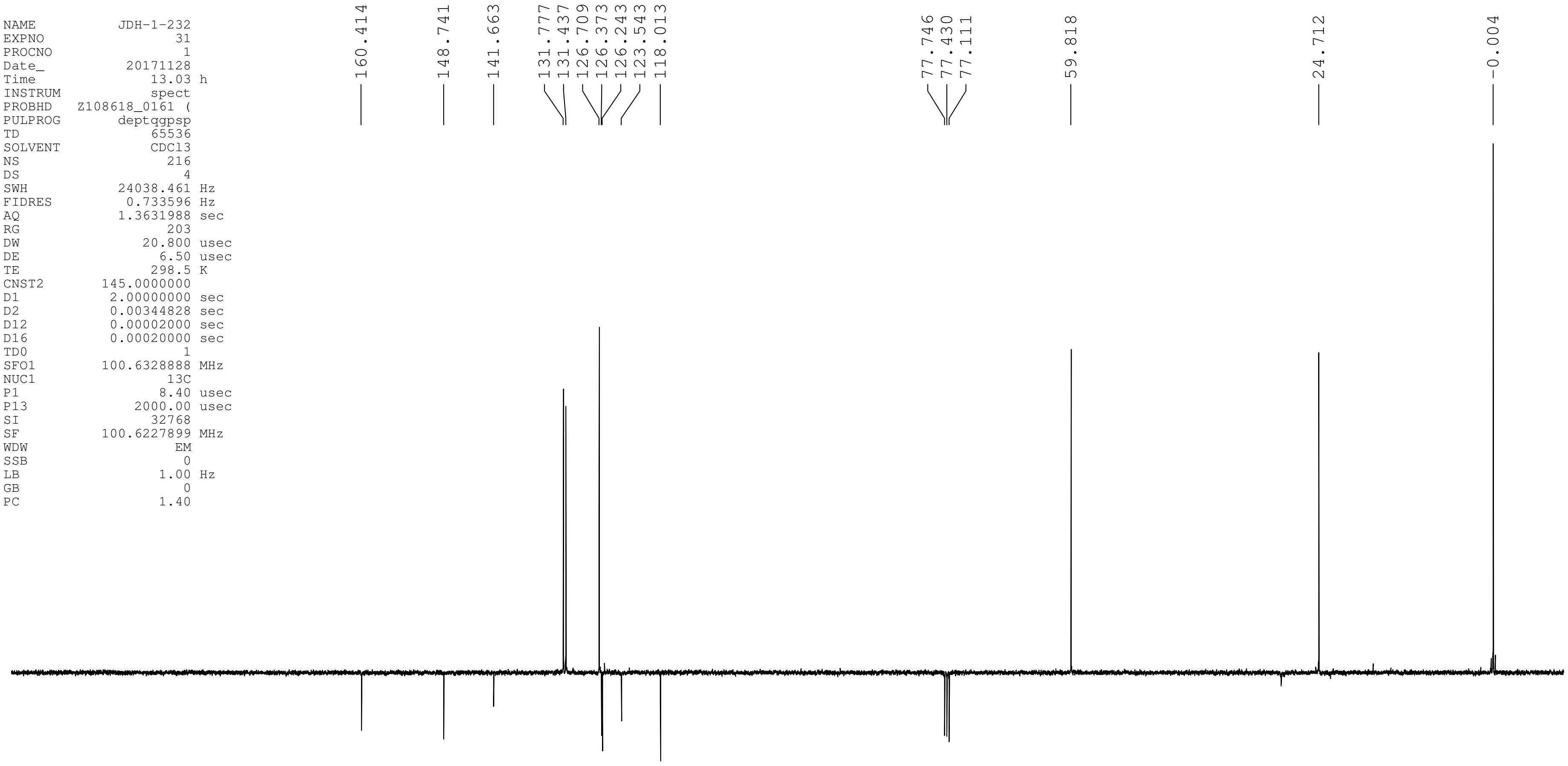

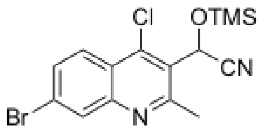

16

2-(7-bromo-4-chloro-2-methylquinolin-3-yl)-2-((trimethylsilyl)oxy)acetonitrile

JDH-1-251 (400MHz, DMSO) Cyanide Hydrolysis

NAME	JDH-1-248
EXPNO	60
PROCNO	1
Date_	20180125
Time	12.53 h
INSTRUM	spect
PROBHD	Z108618_0161 (
PULPROG	zg30
TD	65536
SOLVENT	DMSO
NS	16
DS	2
SWH	8012.820 Hz
FIDRES	0.244532 Hz
AQ	4.0894966 sec
RG	203
DW	62.400 usec
DE	6.50 usec
TE	298.3 K
D1	1.00000000 sec
TD0	1
SFO1	400.1724710 MHz
NUC1	1H
P1	9.88 usec
SI	65536
SF	400.1699993 MHz
WDW	EM
SSB	0
LB	0.30 Hz
GB	0
PC	1.00

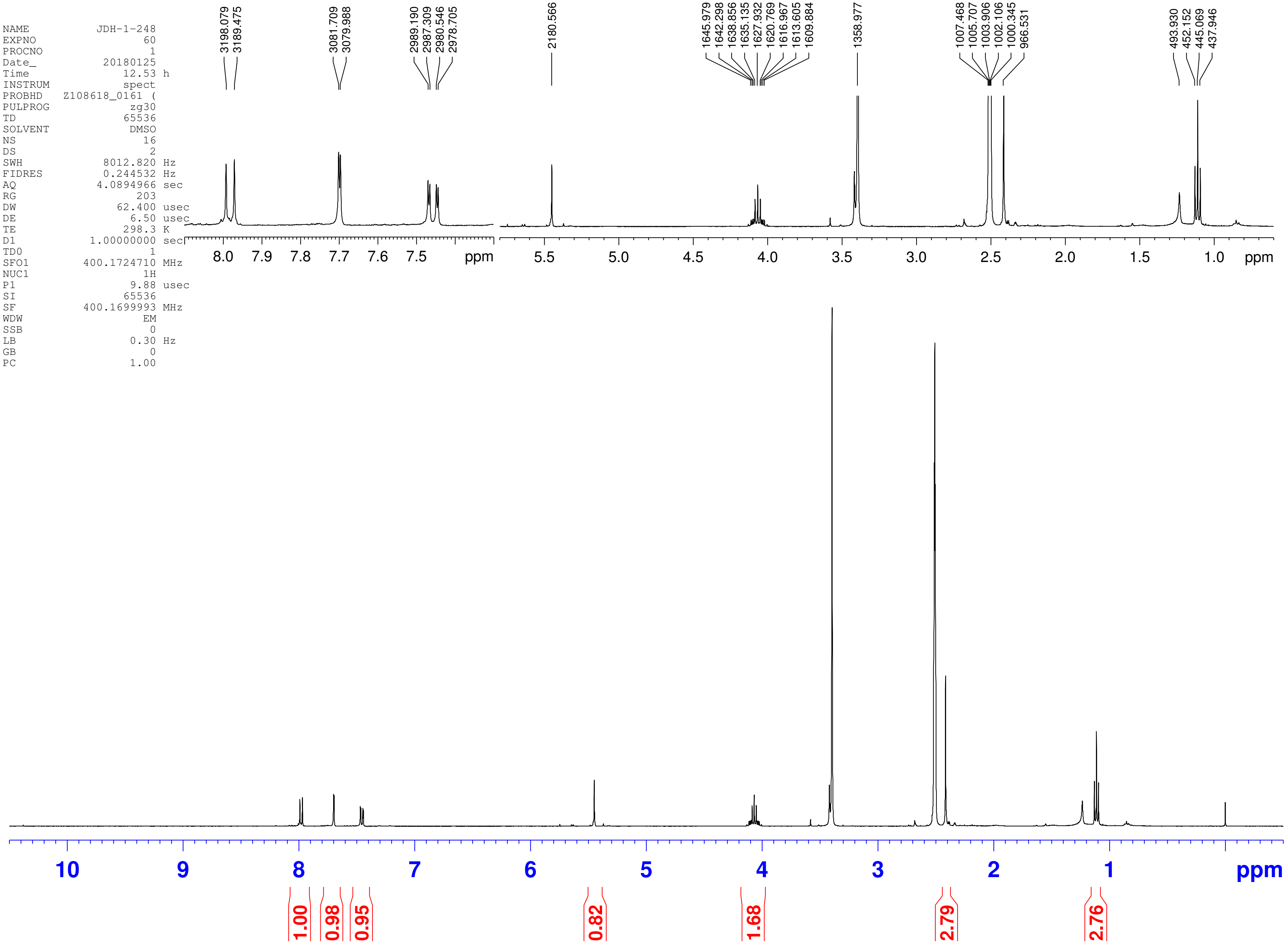

JDH-1-248 (400Mhz, DMSO)
1 drop D2O

NAME	JDH-1-248
EXPNO	71
PROCNO	1
Date_	20180125
Time	21.50 h
INSTRUM	spect
PROBHD	Z108618_0161 (
PULPROG	deptqgpsp
TD	65536
SOLVENT	DMSO
NS	1024
DS	4
SWH	24038.461 Hz
FIDRES	0.733596 Hz
AQ	1.3631988 sec
RG	203
DW	20.800 usec
DE	6.50 usec
TE	298.2 K
CNST2	145.0000000
D1	2.00000000 sec
D2	0.00344828 sec
D12	0.00002000 sec
D16	0.00020000 sec
TD0	1
SFO1	100.6328888 MHz
NUC1	13C
P1	8.40 usec
P13	2000.00 usec
SI	32768
SF	100.6228270 MHz
WDW	EM
SSB	0
LB	1.00 Hz
GB	0
PC	1.40

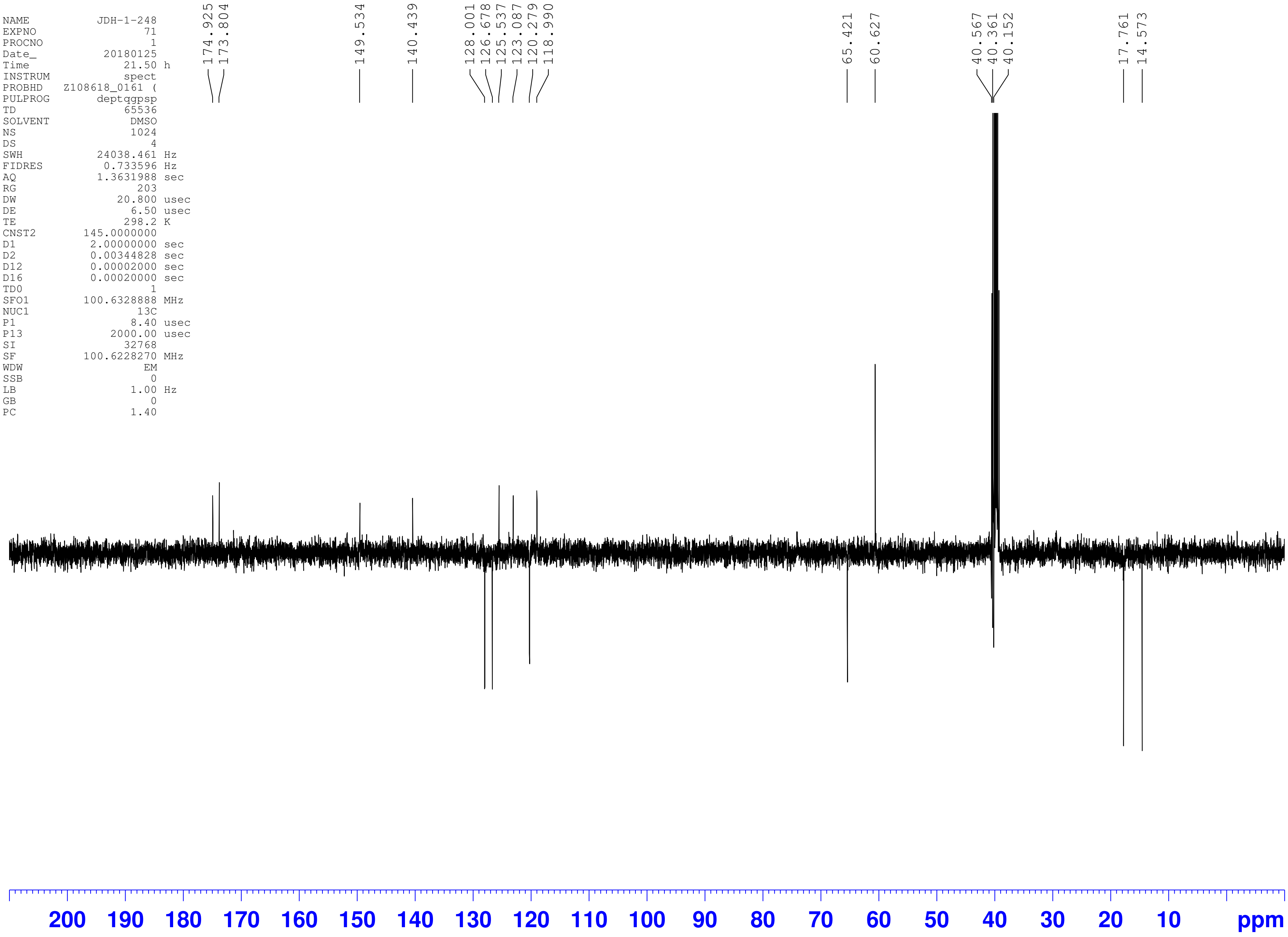

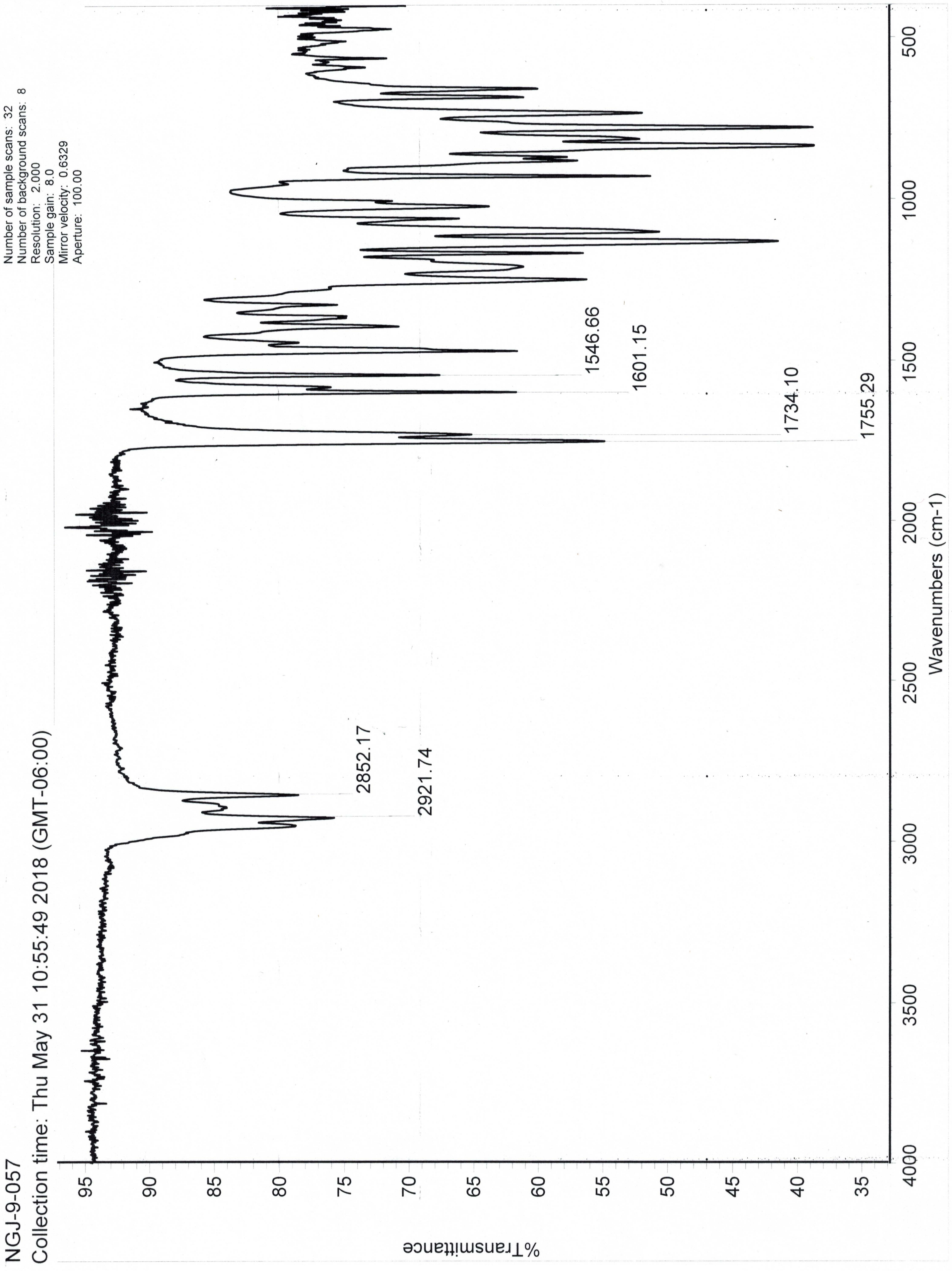

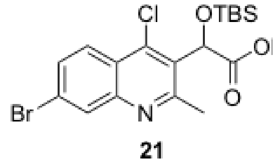

21

ethyl 2-(7-bromo-4-chloro-2-methylquinolin-3-yl)-2-((*tert*-butyldimethylsilyl)oxy)acetate

NGJ-9-057-1 (CDCl3, 400 MHz) Crude Yellow Oil - MAC acylation of 7-bromoquinoline-3-carbaldehy
fraction 12

NAME	NGJ-9-057
EXPNO	40
PROCNO	1
Date_	20180417
Time	17.47 h
INSTRUM	spect
PROBHD	Z108618_0161 (
PULPROG	zg30
TD	65536
SOLVENT	CDCl3
NS	16
DS	2
SWH	8012.820 Hz
FIDRES	0.244532 Hz
AQ	4.0894966 sec
RG	203
DW	62.400 usec
DE	6.50 usec
TE	298.1 K
D1	1.00000000 sec
TD0	1
SFO1	400.1724710 MHz
NUC1	1H
P1	9.88 usec
SI	65536
SF	400.1700096 MHz
WDW	EM
SSB	0
LB	0.30 Hz
GB	0
PC	1.00

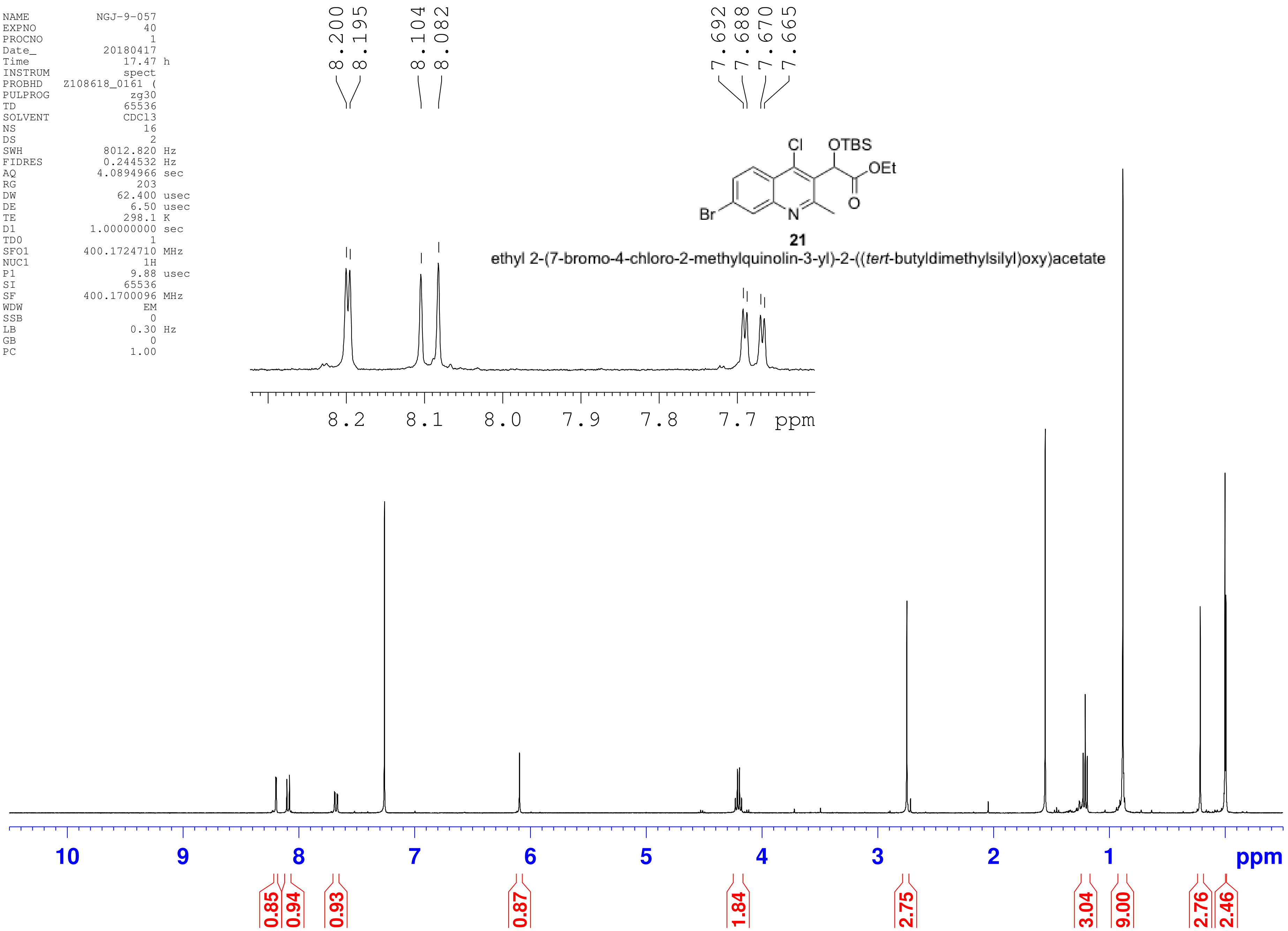

21

ethyl 2-(7-bromo-4-chloro-2-methylquinolin-3-yl)-2-((*tert*-butyldimethylsilyl)oxy)acetate

NGJ-9-057-2 (CDCl3, 400 MHz) Crude Yellow Oil - MAC acylation
fraction 13

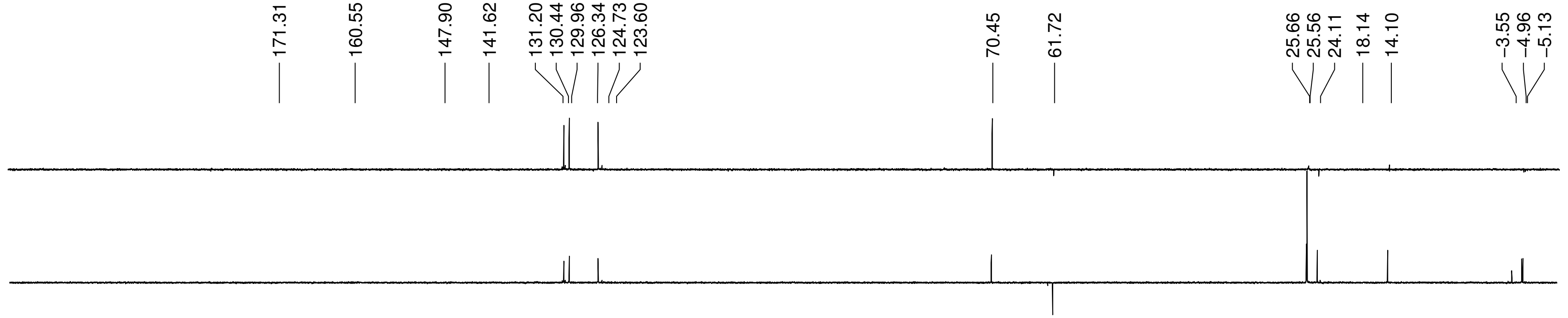

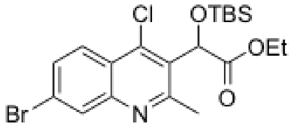

21

ethyl 2-(7-bromo-4-chloro-2-methylquinolin-3-yl)-2-((*tert*-butyldimethylsilyl)oxy)acetate

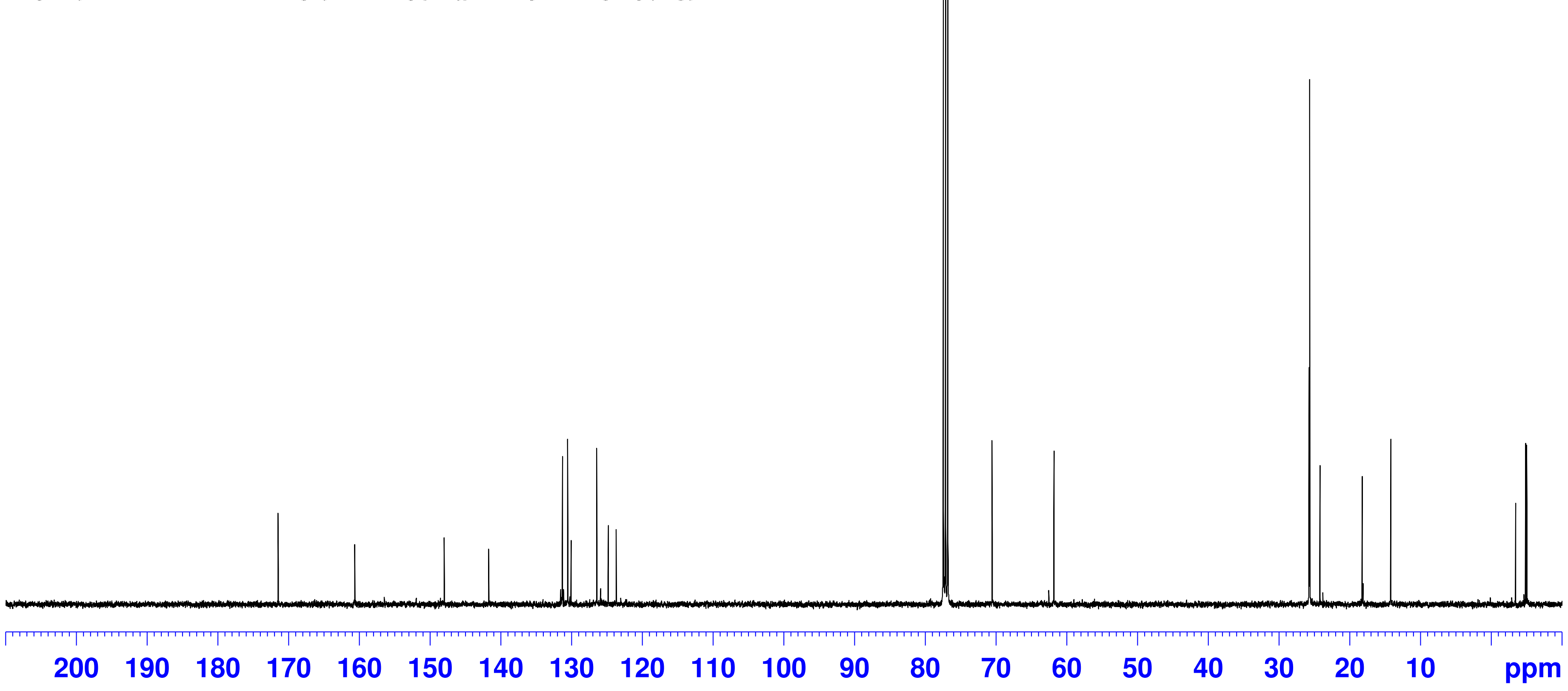

NGJ-9-057-2 (CDCl3, 400 MHz) Crude Yellow Oil - MAC acylation of 7-bromoquinoline-3-carbaldehy
fraction 13

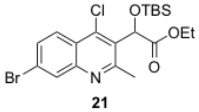

21

ethyl 2-(7-bromo-4-chloro-2-methylquinolin-3-yl)-2-((*tert*-butyldimethylsilyl)oxy)acetate

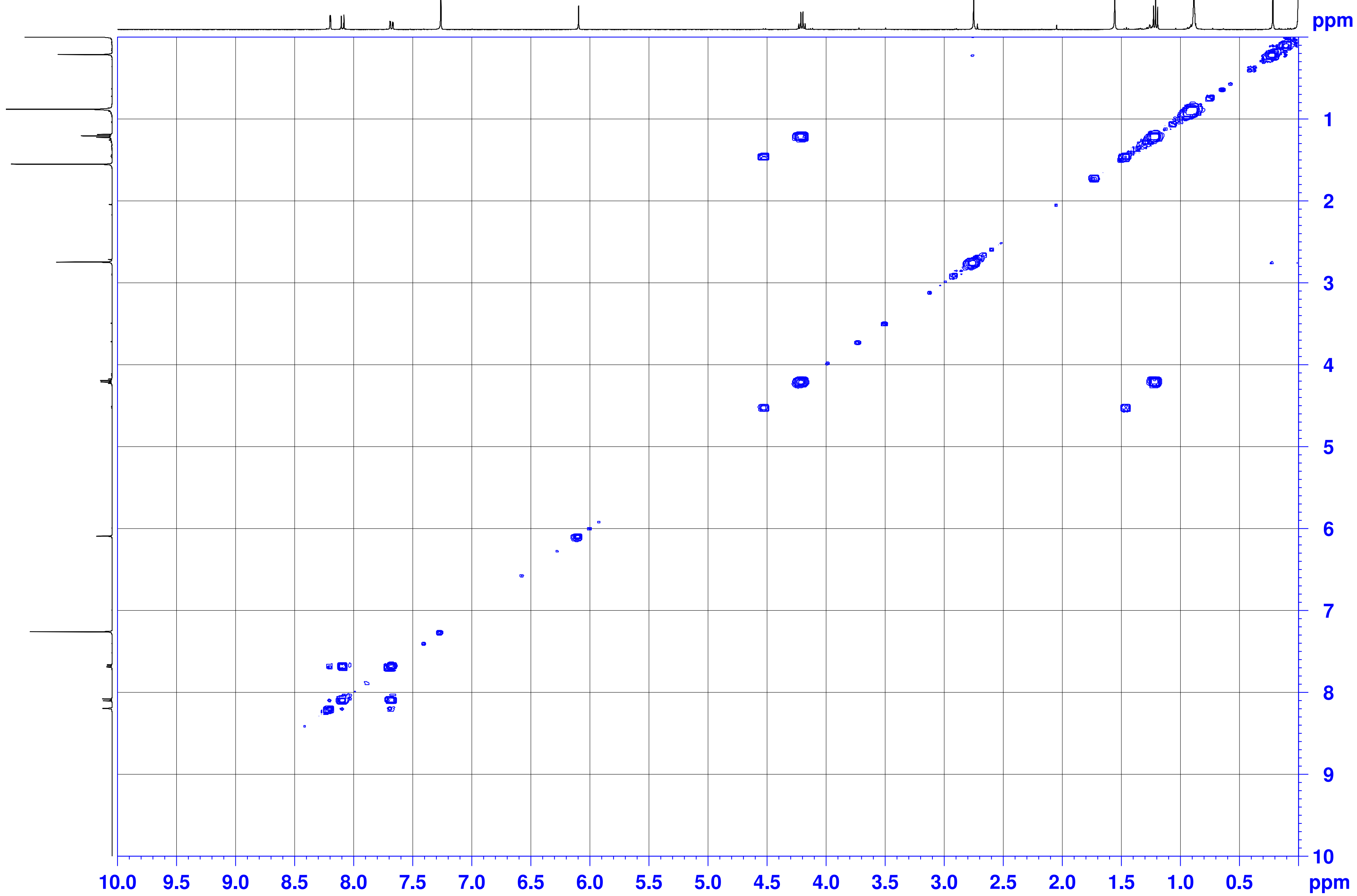

NGJ-9-057-2 (CDCl3, 400 MHz) Crude Yellow Oil - MAC acylation of 7-bromoquinoline-3-carbaldehy
fraction 13

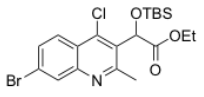

21

ethyl 2-(7-bromo-4-chloro-2-methylquinolin-3-yl)-2-((*tert*-butyldimethylsilyl)oxy)acetate

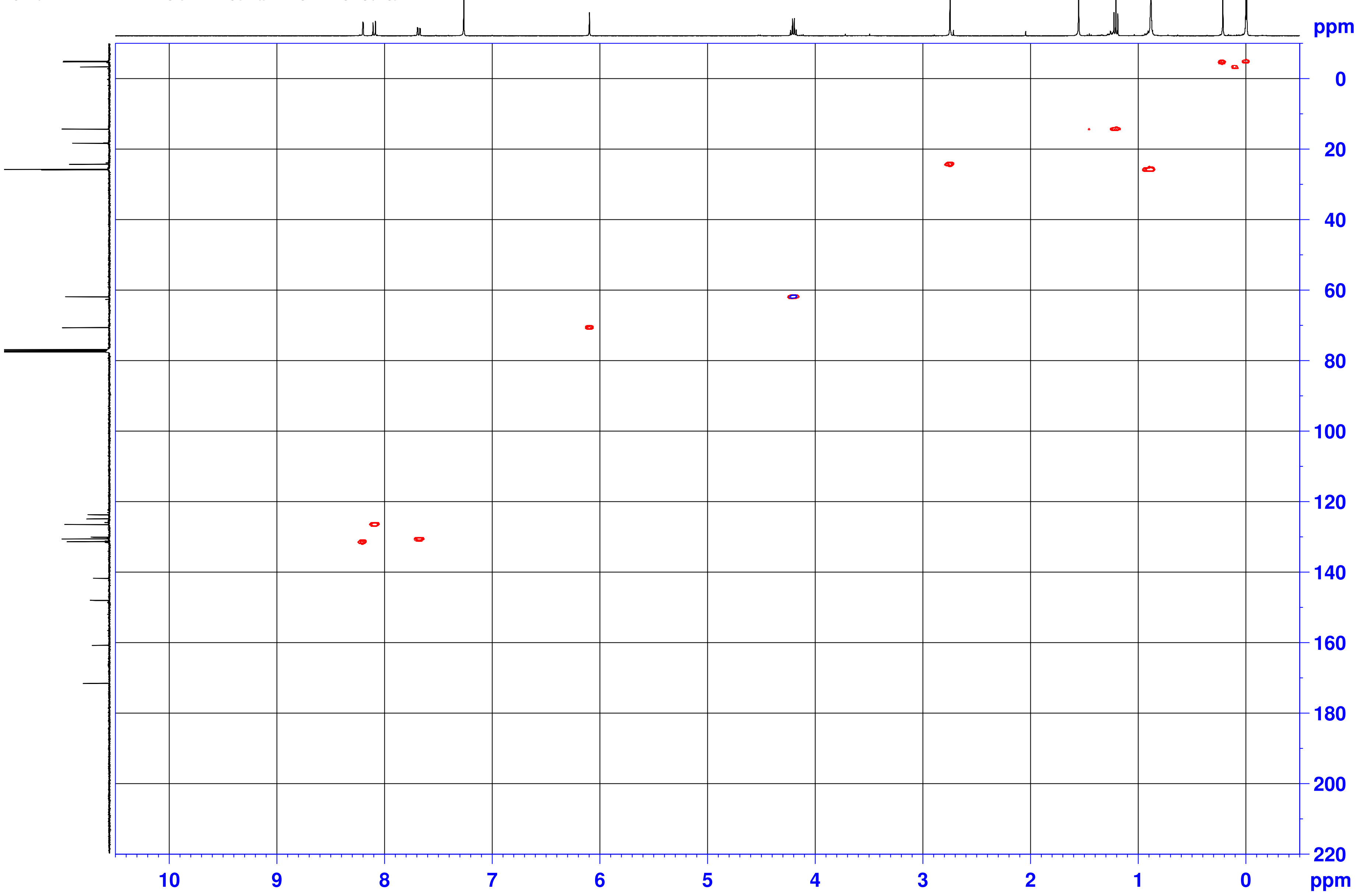

NGJ-9-057-2 (CDCl3, 400 MHz) Crude Yellow Oil - MAC acylation of 7-bromoquinoline-3-carbaldeh[illegible]
fraction 13

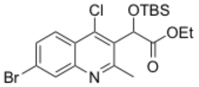

21

ethyl 2-(7-bromo-4-chloro-2-methylquinolin-3-yl)-2-((*tert*-butyldimethylsilyl)oxy)acetate

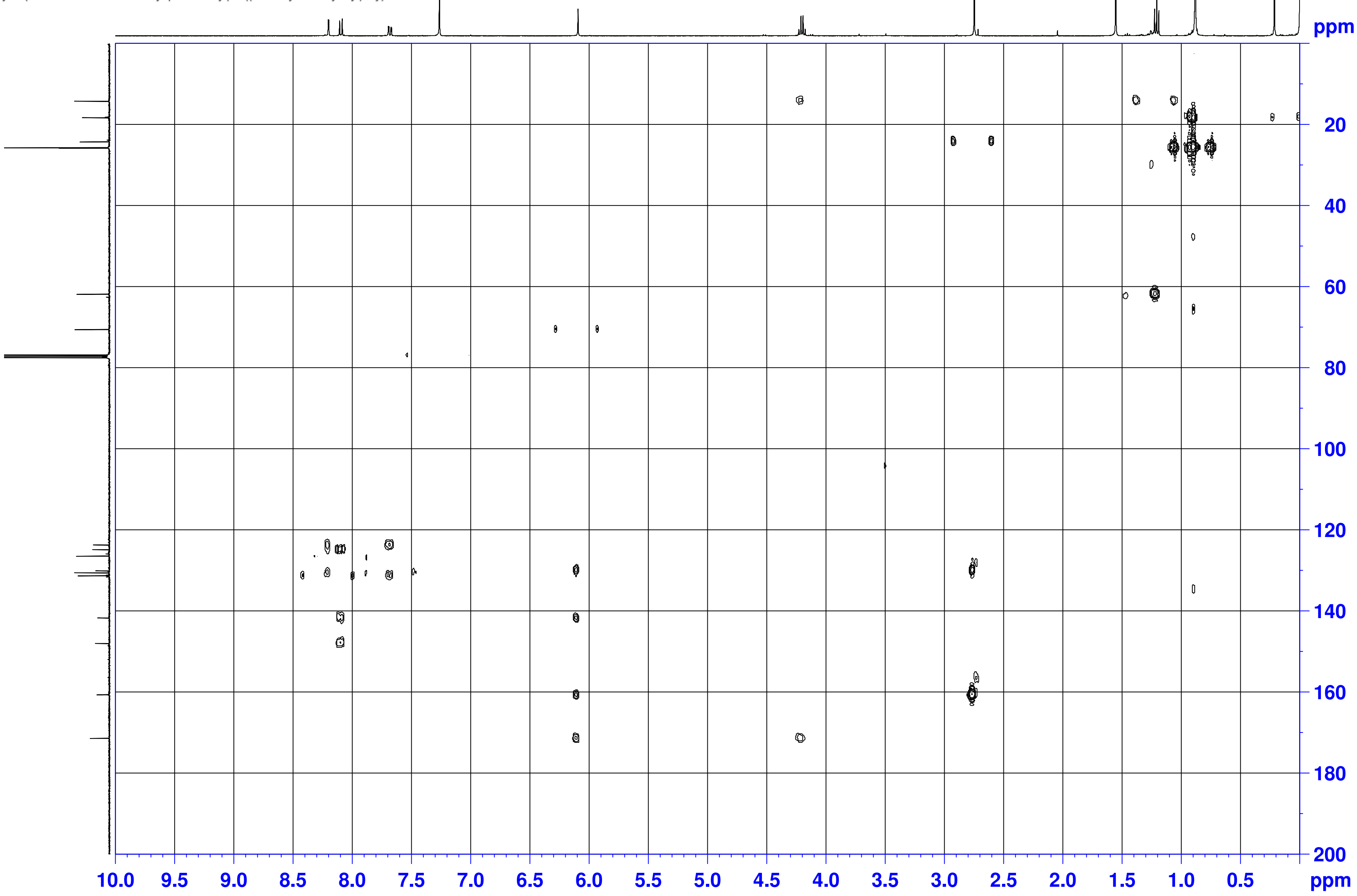

Mass Spectrum List Report

Analysis Info

Analysis Name	Y:\FTICR-MS\MS-1\Data\apexdata080818\NGJ-9-057_pos_000001.d	Acquisition Date	8/8/2018 3:00:04 PM
Method		Operator	FTMS_USER
Sample Name	NGJ-9-057_pos	Instrument	apex-Qe
Comment	NGJ-9-057 in THF:MeOH C20H27BrClNO3Si H+		

Sample Name NGJ-9-057 in THF:MeOH

Exact Mass of C20H27BrClNO3Si H+ = 472.070487 m/z

Mass Observed with signature = 472.070663 m/z

Difference < 1.0 ppm

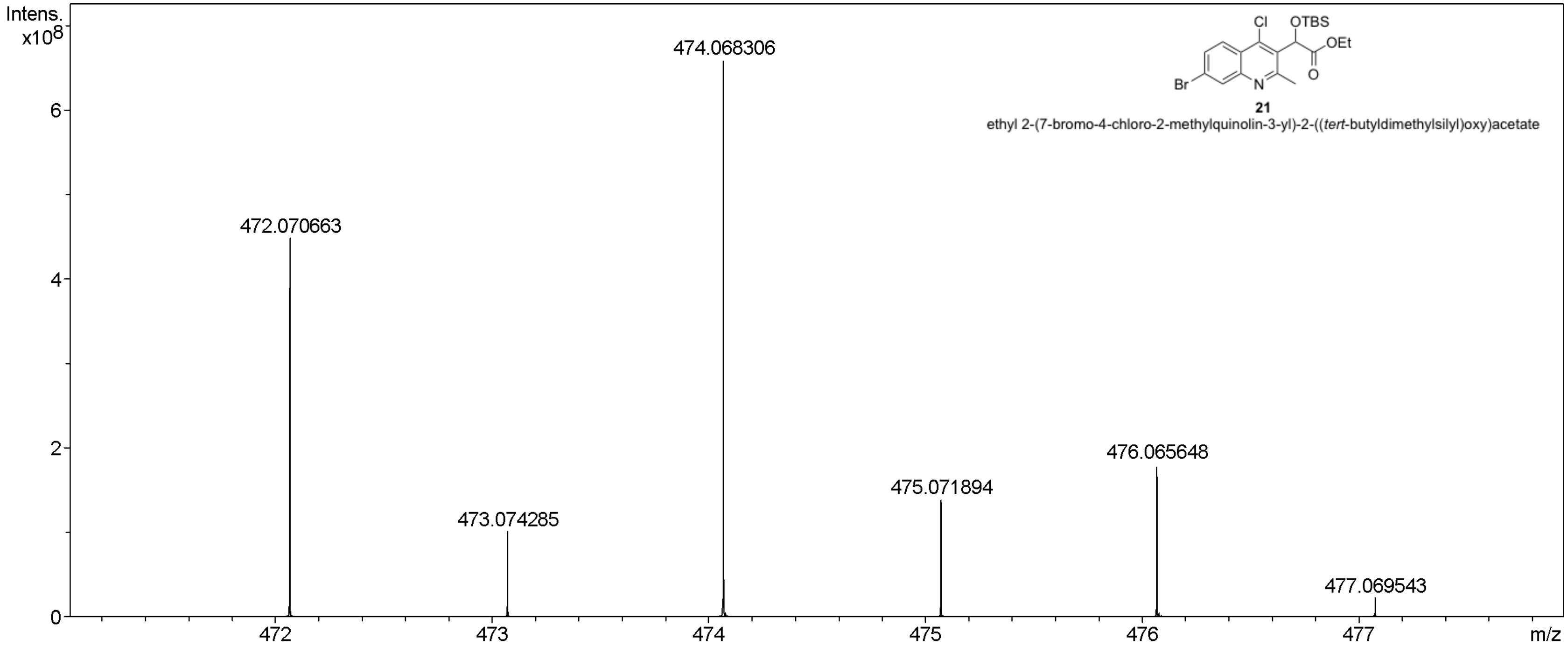

#	m/z	I
1	190.060842	559930
2	199.093957	1361850

Mass Spectrum List Report

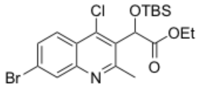

21

ethyl 2-(7-bromo-4-chloro-2-methylquinolin-3-yl)-2-((*tert*-butyldimethylsilyl)oxy)acetate

#	m/z	I
3	199.094212	3484556
4	201.201209	762026
5	208.146237	2155854
6	209.211102	1475350
7	209.441439	1005758
8	213.073487	477454
9	213.109836	3077182
10	214.113235	648390
11	215.089047	50210399
12	216.092507	1136879
13	219.759178	2820643
14	220.397648	2124596
15	221.048875	7251621
16	221.050375	481513
17	221.700567	1991494
18	222.365244	6137447
19	229.104807	803274
20	231.084096	419570
21	231.120410	8383106
22	232.123797	467182
23	236.039870	3146520
24	236.538299	435675
25	237.038775	3110174
26	237.540539	966848
27	238.037416	842051
28	239.125565	689625
29	245.099737	1460158
30	245.136147	2323790
31	247.031340	722875
32	269.136181	1520211
33	271.151875	472870
34	278.175336	468873
35	283.151873	428920
36	284.331432	588899
37	285.131206	479627
38	301.162448	1070012
39	323.019626	760351
40	326.136668	1061107
41	329.989509	638795
42	331.987481	568599
43	340.394218	908874
44	341.971794	587604
45	342.728085	464024
46	354.409874	453281
47	359.984598	1530053

Mass Spectrum List Report

#	m/z	I
48	360.324155	1177032
49	361.982626	1422547
50	362.000289	544275
51	363.998262	649441
52	365.136403	1060073
53	365.266652	447018
54	371.163231	486934
55	381.152493	479018
56	383.205014	444477
57	384.181826	2376198
58	392.029701	446806
59	393.298018	1859939
60	394.301396	496430
61	398.241932	5926299
62	399.042148	1461412
63	399.245342	1205158
64	401.040083	1467323
65	403.037058	484499
66	413.212507	1172817
67	413.266908	897233
68	423.199586	704463
69	424.231606	17888989
70	425.231223	1375977
71	425.235006	3136745
72	425.288053	1975274
73	426.228444	1125366
74	428.121617	469070
75	435.345217	3290984
76	436.348501	742900
77	438.110137	539017
78	440.108106	545953
79	441.298184	735791
80	458.055551	2978402
81	459.059020	505067
82	460.053680	2303604
83	462.050630	702086
84	463.303924	1078160
85	472.068324	15427793
86	472.070663	448440529
87	472.071810	29419729
88	472.072365	14076114
89	472.072942	8612050
90	472.073507	5642450
91	472.074093	3969234
92	472.074683	3024082

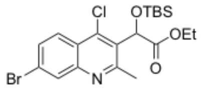

21

ethyl 2-(7-bromo-4-chloro-2-methylquinolin-3-yl)-2-((*tert*-butyldimethylsilyl)oxy)acetate

Mass Spectrum List Report

#	m/z	I
93	472.075262	2381010
94	472.076428	1576146
95	473.068077	1559512
96	473.071018	12174552
97	473.074285	101574872
98	473.075429	7198936
99	473.075987	3331288
100	473.077784	1314008
101	474.064775	15997150
102	474.066169	19015902
103	474.068306	658811102
104	474.069897	22878430
105	474.070523	13576414
106	474.071093	9314526
107	474.071671	6454494
108	474.072277	4903134
109	474.073999	3498206
110	474.075342	2494686
111	474.077713	4693214
112	475.064961	1516772
113	475.066063	2351332
114	475.067809	3633380
115	475.069252	10743524
116	475.071894	139733220
117	475.073022	9525476
118	475.075595	1458148
119	476.063477	10916074
120	476.065648	180889834
121	476.072556	1646442
122	476.074629	1400298
123	476.075935	4472042
124	476.083110	502890
125	476.087165	1318122
126	477.063076	620783
127	477.066617	3275503
128	477.069543	22653167
129	478.063289	1979124
130	478.073163	1268724
131	478.085223	1158900
132	480.551254	791169
133	481.253391	1180164
134	487.423976	587037
135	494.050312	9177393
136	494.052782	101443889
137	494.053901	8522033

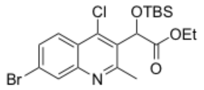

21
ethyl 2-(7-bromo-4-chloro-2-methylquinolin-3-yl)-2-((*tert*-butyldimethylsilyl)oxy)acetate

Mass Spectrum List Report

#	m/z	I
138	494.055185	2493745
139	494.566873	507954
140	495.049883	601651
141	495.053671	2520371
142	495.056642	11864371
143	496.047236	7064885
144	496.048232	11149621
145	496.050410	126200117
146	496.051633	10324277
147	496.052227	5118261
148	496.052856	3142965
149	496.056659	800565
150	496.060317	721845
151	497.050642	1680695
152	497.051710	2572087
153	497.053575	3496247
154	497.054654	10318135
155	498.045372	4625721
156	498.048217	21186873
157	498.058161	912697
158	499.048776	1106491
159	499.051774	2846523
160	508.152452	458054
161	508.579519	1792327
162	508.582629	4036935
163	509.582956	660423
164	509.586053	1250631
165	512.046366	448264
166	513.082743	572873
167	515.080551	654793
168	519.439393	1396553
169	520.442694	519881
170	522.598248	1467720
171	523.325163	5754183
172	523.601507	542151
173	524.060491	1121863
174	524.063883	20699463
175	524.328562	1574727
176	524.577632	1171271
177	525.067457	3069255
178	525.377154	778822
179	526.061700	18196806
180	527.065436	2342213
181	528.059063	4463941
182	529.062481	840900

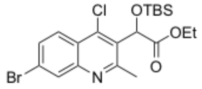

21

ethyl 2-(7-bromo-4-chloro-2-methylquinolin-3-yl)-2-((*tert*-butyldimethylsilyl)oxy)acetate

Mass Spectrum List Report

#	m/z	I
183	533.455165	1561153
184	534.458586	589632
185	534.598326	860480
186	536.610934	3767102
187	536.613689	27150654
188	537.396008	584381
189	537.614656	1655805
190	537.617306	6846781
191	538.620697	1272380
192	542.488666	494520
193	542.491980	1251896
194	543.495203	478136
195	544.507616	603831
196	550.629599	1304882
197	551.632953	580529
198	552.605312	706992
199	552.608938	3200304
200	553.612311	1204015
201	564.645325	11942187
202	565.648687	4206891
203	566.652038	649643
204	568.109349	463404
205	569.403639	1946412
206	570.407105	570412
207	578.661166	841267
208	580.640377	1391925
209	581.643790	504759
210	590.661064	1014602
211	591.664603	452556
212	592.676665	20035919
213	593.680136	6785362
214	594.683452	1090390
215	599.549729	529639
216	604.630326	585342
217	608.671843	2534292
218	609.675231	993177
219	611.414454	911780
220	613.430033	3255729
221	614.433405	1094071
222	620.708318	1355494
223	621.711654	458095
224	642.631228	855247
225	645.359324	1992946
226	646.362830	504448
227	648.739761	758815

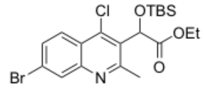

21

ethyl 2-(7-bromo-4-chloro-2-methylquinolin-3-yl)-2-((*tert*-butyldimethylsilyl)oxy)acetate

Mass Spectrum List Report

#	m/z	I
228	655.441070	1293179
229	657.456549	4540310
230	658.459843	1618852
231	685.431008	1606904
232	685.436979	77376760
233	685.439376	4721912
234	686.440652	30280962
235	687.444219	4273420
236	688.448147	486806
237	699.467193	1097581
238	701.482851	3390840
239	702.486283	1196413
240	732.211387	474199
241	734.209248	566987
242	743.493609	762632
243	745.509300	1919225
244	746.512785	766961
245	789.535706	934728
246	790.539495	574412
247	804.989930	477763
248	806.987375	1088987
249	808.985156	690037
250	875.661084	507747
251	877.676876	655345
252	919.688181	963063
253	920.691435	489844
254	921.703072	1207026
255	963.713587	1094487
256	964.717620	673249
257	965.730236	1302891
258	966.733747	949622
259	1007.741254	1265104
260	1008.744665	772576
261	1009.756928	1146608
262	1010.759627	646911
263	1051.767502	711863
264	1052.771124	566204
265	1053.783609	1036480
266	1054.786882	569668

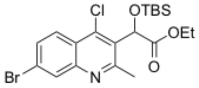

21

ethyl 2-(7-bromo-4-chloro-2-methylquinolin-3-yl)-2-((*tert*-butyldimethylsilyl)oxy)acetate
